# Mechanochemical Synthesis of 2‑Amino-1,4-naphthoquinones and Telescopic Synthesis of Lawsone

**DOI:** 10.1021/acsomega.4c11349

**Published:** 2025-08-27

**Authors:** Igor Sande, Talita Santana Nascimento, Sabrina Martinez, Sâmia Rocha Lima, Silvio Cunha

**Affiliations:** † Instituto de Química, 28111 Universidade Federal da Bahia, Campus de Ondina, Salvador, Bahia 40170-115, Brazil; ‡ Instituto Nacional de Ciência e Tecnologia - INCT em Energia e Ambiente, Universidade Federal da Bahia, Campus de Ondina, Salvador, Bahia 40170-290, Brazil

## Abstract

A mechanochemical solvent-free synthesis of 2-amino-1,4-naphthoquinones was developed by the reaction of aromatic/aliphatic amines with naphthoquinone, and better yields were obtained with aromatic amines. The combination of sodium acetate as a base and silica as solid auxiliary grinding was crucial in most cases. The simplest 2-amino-1,4-naphthoquinone was prepared via mechanochemistry and under microwave heating (high yield) by applying the known combination of sodium azide/acetic acid. In the mechanochemical reaction of binucleophile 2-amino-4-methylphenol, a selectivity dependence was observed with applied frequency, and chemotherapeutic 10-methyl-benzo­[a]­phenoxazine-5-one was isolated as a minor product at a high frequency, which was alternatively synthesized via microwave from Lawsone and from naphthoquinone, being an innovation to this class of bioactive compound. Mechanochemistry had the advantage of multigram preparation of 2-amino-1,4-naphthoquinone and 2-(phenyl)­amino-1,4-naphthoquinone, which were applied in the two-step synthesis of natural product Lawsone via acid hydrolysis. A more sustainable telescopic synthesis of Lawsone was accomplished and is the first straightforward total synthesis involving mechanochemistry.

## Graphical abstract

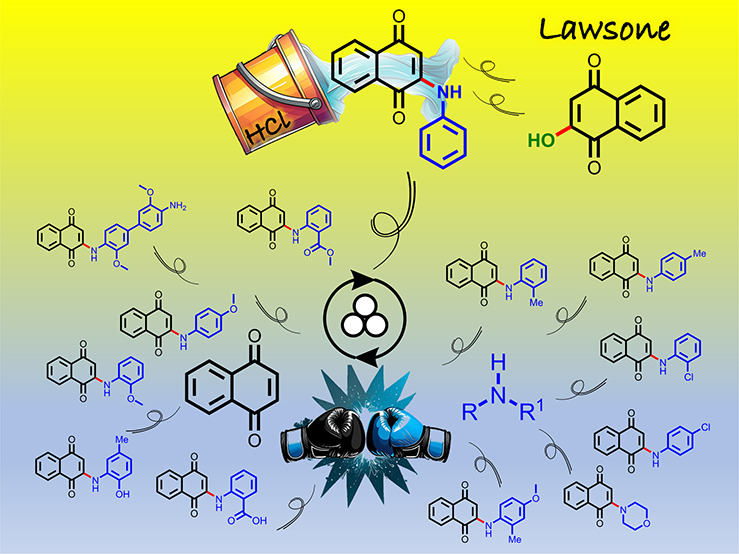

## Introduction

Enabling technologies are essential to achieve sustainable chemistry,^1^ and the International Union of Pure and Applied Chemistry (IUPAC) identified mechanochemistry as one of the top ten chemical innovations for a more sustainable future with the potential to change our world.^2^ Since the beginning of the new century, mechanochemistry has been continuously and intensively applied in organic synthesis,^3–11^ and mechanochemistry for sustainable industry is the next frontier.^12^

Compounds containing a 2-amino-1,4-naphthoquinone moiety in their structure have demonstrated activities such as anticancer,^13^ antibacterial,^14^ and antiseizure,^15^ and are synthetic intermediates to access functionalized quinone derivatives.^16–25^ Besides, 2-amino-1,4-naphthoquinones are important in materials and technological fields, for example, in the composition of redox-polymer-based proton battery in aqueous system,^26^ and selective multichannel chemosensor to detect and quantify Hg^2+^ ions.^27^

Since the pioneering contribution of Fieser in the synthesis of 2-amino-1,4-naphthoquinones **3**,^28^ the simplest synthetic methodology to access this versatile compound involves the reaction of 1,4-naphthoquinone **1** with amines **2** to afford **3** by oxidative coupling in which the C­(sp^2^)–H is converted to C­(sp^2^)–N bond, Figure 1.^29–42^ Metal-free and metal-promoted strategies are available, being these oxidative additions more recurrent in the literature in the presence of metal catalysts such as NaAuCl_4_·2H_2_O,^29^ Cu­(OAc)_2_,^30,31^ FeCl_3_,^31^ CeCl_3_·7H_2_O,^31–33^ Zn­(OAc)_2_·2H_2_O,^34^ AgOAc,^35^ and BiCl_3_.^36^ In some cases, equimolar amounts of oxidant are necessary. Another strategy is based on the nucleophilic substitution of 2-hydroxy,^43,44^ 2-methoxy^45^ and 2-halo-1,4-naphthoquinones with amines.^15,46–52^

**Figure 1:**
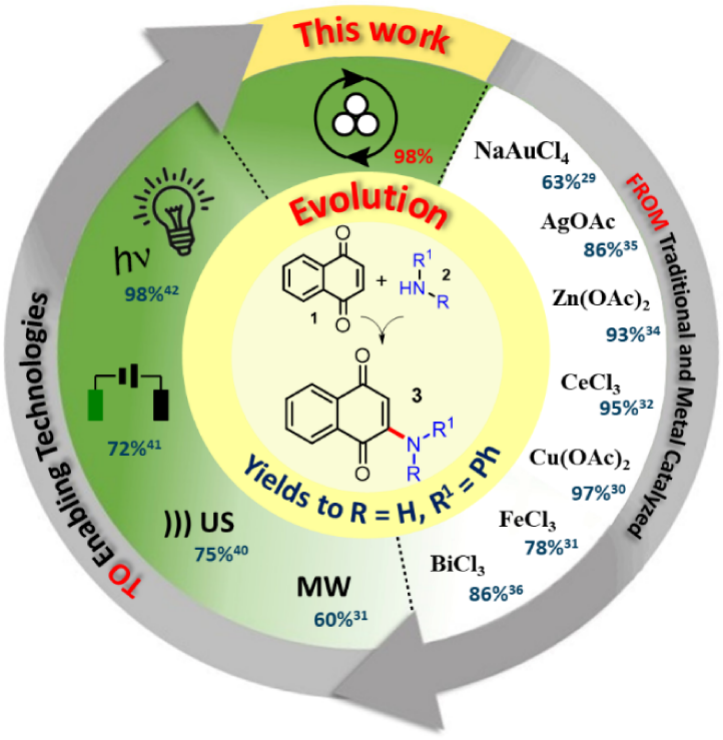
Evolution of the synthesis approach to 2-amino-1,4-naphthoquinones from amines and 1,4-naphthoquinone.

To increase the sustainable aspect, others approaches combine enabling technologies and Lewis acid to access 2-amino-1,4-naphthoquinones **3**, such as I_2_,^37,38^ HClO_4_–SiO_2_ ^39^ with ultrasound irradiation (US), FeCl_3_, CeCl_3_, and Cu­(OAc)_2_ catalysis under microwave heating (MW).^33^ In a greener approach, these enabling technologies were employed without Lewis acid, so that MW^14^ and US^40^ afforded 2-amino-1,4-naphthoquinones without requiring any metal catalyst or additional oxidant, achieving short reaction times. In addition, synthesis of **3** was achieved by electrooxidative coupling between **1** and **2** employing platinum electrochemical cell in the presence of NH_4_I and LiClO_4_.^41^ More recently, it has been reported the photochemical oxidative C­(sp^2^)–H/C­(sp^2^)–N coupling between 1,4-naphthoquinone and amines to obtain 2-amino-1,4-naphthoquinones, Figure 1.^42^

Despite there being several advantages in the use of US, MW, electrochemistry, and photochemistry in the synthesis of 2-amino-1,4-naphthoquinones, all these methodologies are solvent-dependent. Therefore, the mechanochemistry approach should be an excellent opportunity due to its inherent solvent-free nature and the possibility of carrying out gram-scale reactions. There is only one mechanically grinded syntheses of **3** in solid phase by Mortar and Pestle, limited in scope and low yields, with much of the starting material being not consumed.^53^

Herein, we developed the mechanosynthesis of 2-amino-1,4-naphthoquinones **3** under solvent-, metal-, and oxidant-free conditions, including multigram-scale preparations. As an example of application, two mechanochemical prepared 2-amino-1,4-naphthoquinones were converted into Lawsone, providing a new route to this synthetic useful natural product.^54–56^

## Results and Discussion

The mechanochemical route of the title compounds was investigated in a planetary ball mill reactor with different jar volumes. Due to the vertically positioned jar in this equipment during milling, it is suitable for accommodating significant amounts of liquid reagents without overflowing, enabling reactions without solid support and facilitating scale-up.^57^ A model reaction for the mechanosynthesis of 2-(phenyl)­amino-1,4-naphthoquinone **3a** was investigated by the reaction of 1,4-naphthoquinone **1a** with aniline **2a**. Aniline was selected for the optimization because **3a** was previously prepared by diverse known methodologies^30,53^ and thus a direct comparison concerning the reaction time and yield can be easily done. Several reaction variables can be investigated under mechanochemical conditions. In the present study, the planetary ball milling reactor was coupled to a 12 mL stainless-steel jar with four stainless-steel balls (*N*_MB_ = 4) of 10 mm diameter (*d*_MB_ = 10 mm) with a milling-ball (MB) filling degree of 17% (Φ_MB_ = 0.173). These mechanochemical parameters were initially fixed, and the other reaction conditions were optimized based on TLC analysis and posteriorly by isolated reaction yield, Table 1.

**Table 1:**
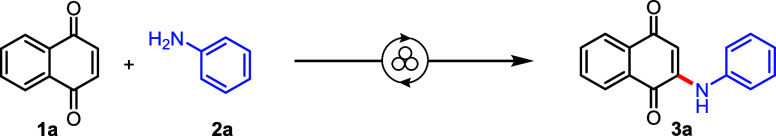
Optimization of the Mechanochemical Synthesis for 2-(Phenyl)­amino-1,4-naphthoquinone **3a** ^a^ ^b^ ^c^

Entry^a^	Time (min)	**2a** (eq )	Frequency (rpm)	Solid support	Base (eq )	Yield^c^ (%)
1	10	1.1	400	—	—	**1a**+**3a**
2	30	1.1	400	—	—	**1a**+**3a**
3	30	1.1	500	—	—	**1a**+**3a+**PB^d^
4	30	5.0	500	—	—	**1a**+**3a**
5	60	5.0	500	—	—	**1a**+**3a**
6^b^	15	1.1	500	SiO_2_	—	**1a**+**3a+**PB^d^
7^b^	15–60	1.1	400	SiO_2_	—	77
8^b^	15	1.1	400	SiO_2_	K_2_CO_3_ (0.5)	73
9^b^	15	1.1	400	SiO_2_	NaHCO_3_ (0.5)	76
10^b^	15	1.1	400	SiO_2_	NaOAc·3H_2_O (0.5)	84
11^b^	15	1.1	400	SiO_2_	NaOAc·3H_2_O (1.0)	98

a Reactions performed in a 12 mL stainless-steel vessel with stainless-steel balls (N_MB_ = 4; d_MB_ = 10 mm; Φ_MB_ = 0.173), **1a** (0.32 mmol).

b SiO_2_:**1a** (10:1) m/m.

c Product **3a** detected but not isolated (in mixture with **1a**) or isolated yield.

d PB: polar byproduct.

With the selected mechanochemical parameters (*N*_MB_, *d*_MB_, and Φ_MB_), the reaction of 1,4-naphthoquinone **1a** and aniline **2a** was investigated of 400 rpm for 10 min, and TLC analysis revealed the formation of **3a**, but **1a** was not completely consumed (Table 1, entry 1). Increasing the reaction time to 30 min did not show a significant alteration (entry 2). Thus, the milling frequency was changed to 500 rpm and an increase in the consumption of **1a** was observed by a decrease in its TLC spot diameter, along with the formation of a polar byproduct (Table 1, entry 3). Subsequently, a large excess of aniline and an increase in reaction time were evaluated, but no significant alteration in the TLC profile occurred (Table 1, entries 4 and 5).

Incomplete reactions under the conditions of entries 1–5 of Table 1 were indicated by the presence of **1a** and should be associated with the accumulation of solid aggregates on the wall of the flask, which decreases the contact surface between the reactants. The solid aggregates form when paste or gum formation is observed during milling, which makes adequate mixing and energy transfer difficult. To minimize or avoid their formation, a mechanochemical strategy relies on the use of a solid auxiliary grinding (SAG) agent, which disperses reagents. Therefore, silica gel (70–230 mesh) was selected as the SAG in a proportion of 10:1 m/m in relation to **1a** (Table 1, entry 6), and an increase in the consumption of **1a** occurred with the formation of the polar byproduct at 500 rpm. It was rationalized that byproduct formation is associated with more energy supply with the increase of frequency from 400 to 500 rpm (compare entries 1–2 with 3–6 of Table 1). Changing the frequency back to 400 rpm, **3a** was isolated in 77% yield, despite unreacted **1a** still being detected by TLC (Table 1, entry 7). The initial hypothesis proved to be assertive because no polar byproduct was detected via TLC under a frequency of 400 rpm, entries 7–11.^53^

The use of silica has the bonus of facilities sample handling by inserting the reaction mixture directly into the column for purification by chromatography. However, in addition to the intrinsic SAG effect, the choice of silica was based on the known proposed reaction mechanism for the oxidative coupling of 1,4-naphthoquinone **1a** with amines **2**, which involves Lewis acid carbonyl activation. Besides, an external source of base should be necessary to drive the reaction progress to product **3** by acid–base reaction with the intermediate protonated aniline (see below in Scheme 2C). Furthermore, the association of silica and inorganic bases was investigated, and potassium carbonate (p*K*_a_ 10.33) was tested in 0.5 equiv (Table 1, entry 8), with a smaller decrease in yield, with total consumption of **1a** for the first time. Since the yield of compound **3a** in the same reaction without base was comparable (73% with and 77% without potassium carbonate) and compound **1a** could be detected by TLC (compare entries 7 and 8), the consumption of reagent **1a** in the presence of base without any increase in yield is likely associated with a possible base-induced redox transformation of compound **1a**. To preclude this, the weaker base sodium bicarbonate (p*K*_a_ 6.35) was tested under the same conditions (Table 1, entry 9), but the TLC showed the same pattern with 76% yield. Subsequently, weaker sodium acetate trihydrate (p*K*_a_ 4.76) was used (Table 1, entry 10), the yield increased to 84%, and the presence of **1a** was still observed. Using twice the initial amount of this base improved the yield of **3a** to 98% without detection of **1a** (Table 1, entry 11). Once a high yield was achieved, the initial mechanochemical parameters (*N*_MB_ = 4, *d*_MB_ = 10 mm, Φ_MB_ = 0.173) did not need further optimization.

**Scheme 1:**
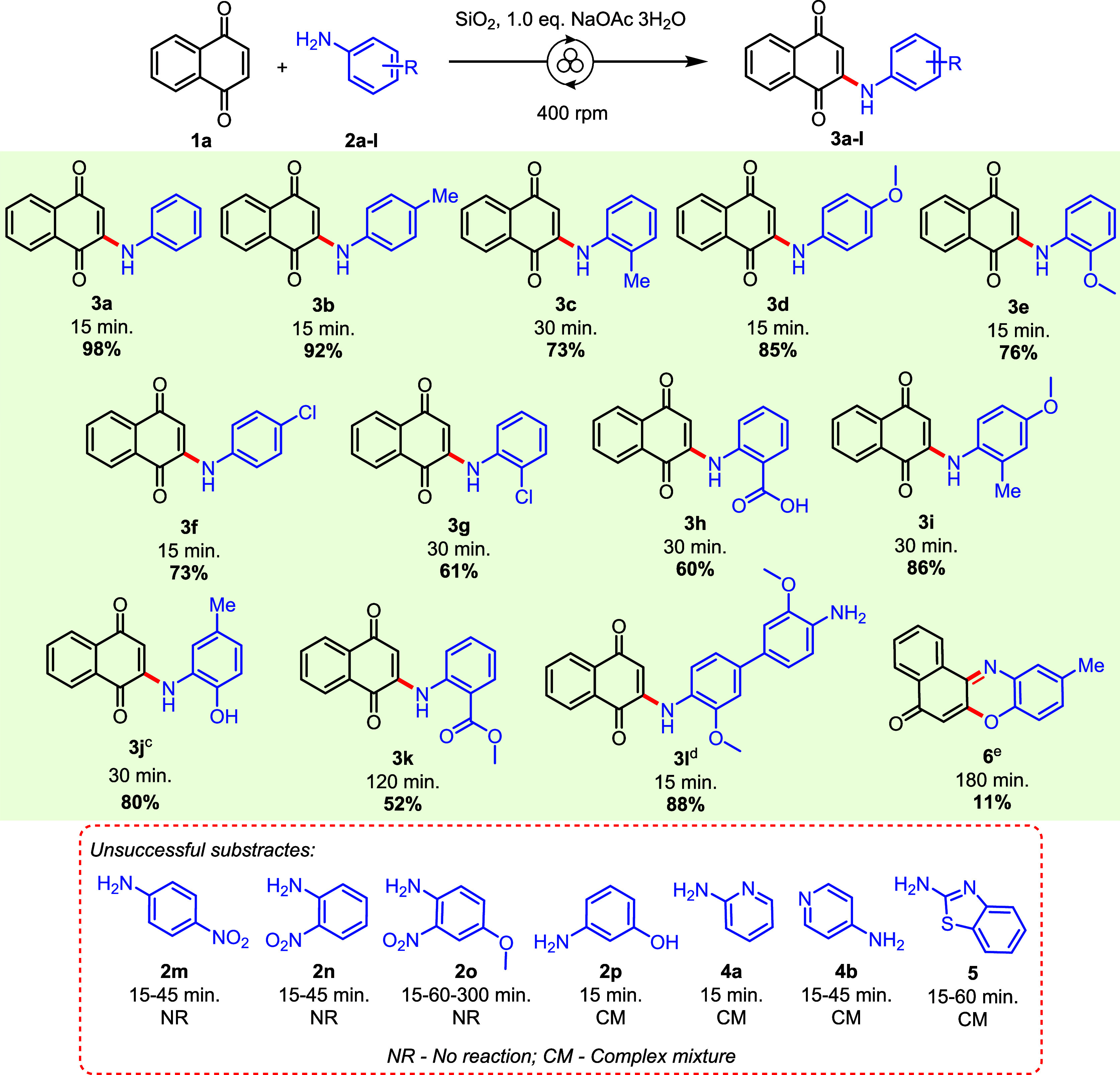
Phenylamine Scope for 2-Arylamino-1,4-naphthoquinones^a,b,c,d,e^ ^a^ ^b^ ^c^ ^d^ ^e^

a Reactions were carried out in a 12 mL stainless-steel vessel with four stainless-steel balls (*N*_MB_ = 4; *d*_MB_ = 10 mm; Φ_MB_ = 0.173) at 400 rpm, **1a** (0.32 mmol), **2** (0.35 mmol), NaOAc.3H_2_O (0.32 mmol), and SiO_2_:**1a** (10:1 m/m).
b Isolated yields.
c Without NaOAc·3H_2_O.
d Reaction conditions: **1a** (0.64 mmol), **2j** (0.32 mmol).
e Reaction was carried out at 500 rpm, **1a** (1.00 mmol), **2j** (1.00 mmol), SiO_2_:**1a** (10:1 m/m), **3j** was also formed in 69%.

**Scheme 2:**
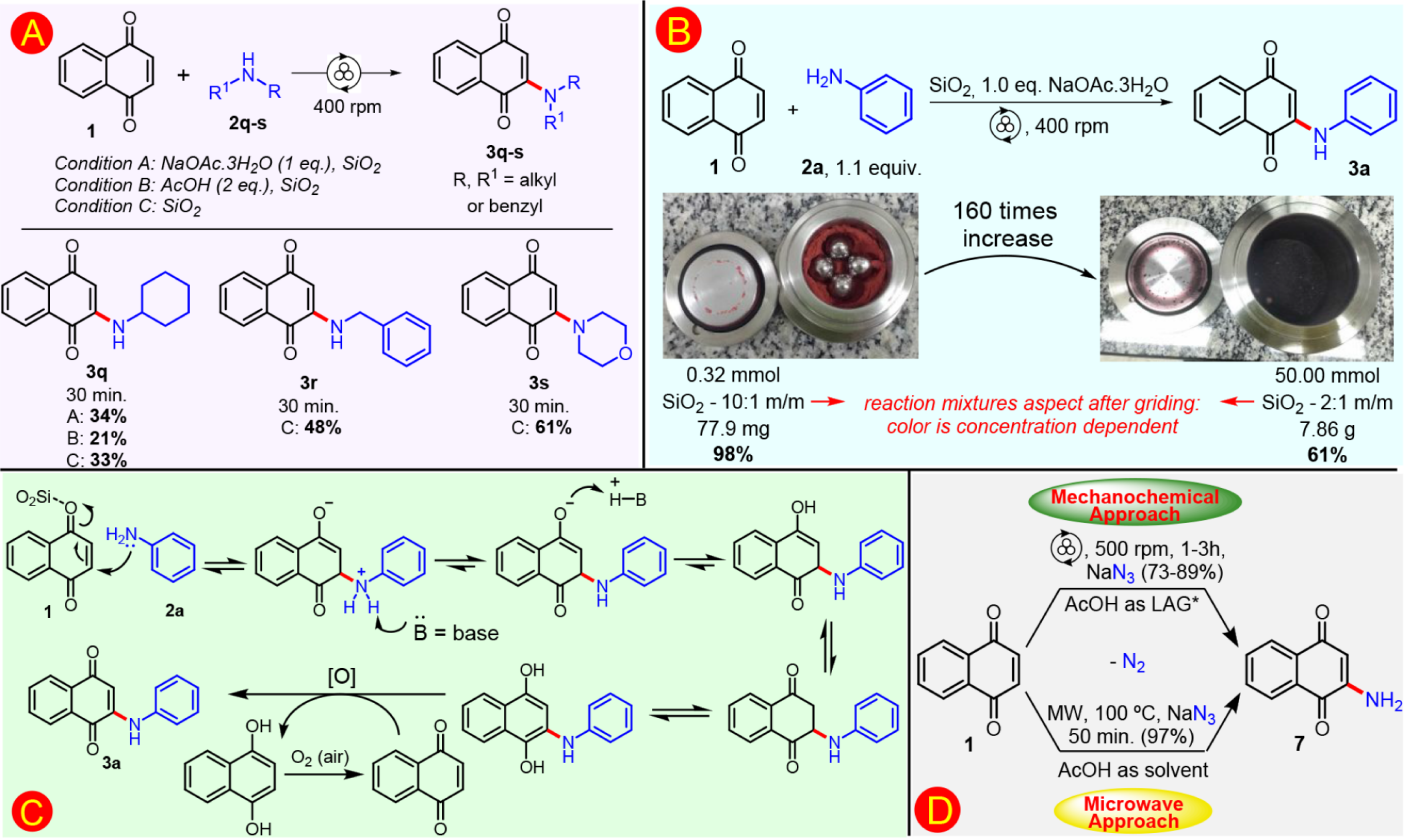
(A) Substrate Scope for 2-Alkylamino-1,4-naphthoquinones;^a,b^ (B) Mechanosynthesis of **3a** in Multigram Scale;^c^ (C) Proposed Mechanism for the Formation of **3a**; (D) Synthesis of 2-Amino-1,4-naphthoquinone **7** under Mechanochemistry;^d^ and Microwave Heating[Fig sch2] ^a^ ^b^ ^c^ ^d^

a The reactions were placed in a 12 mL stainless-steel vessel with four stainless-steel balls (*N*_MB_ = 4; *d*_MB_ = 10 mm; Φ_MB_ = 0.173) at 400 rpm, **1a** (1.00 mmol), **2** (1.00 mmol), and SiO_2_:**1a** (6:1 m/m).
b Isolated yields.
c The reactions were placed in a 125 mL stainless-steel vessel with ten stainless-steel balls (*N*_MB_ = 10, *d*_MB_ = 10 mm, Φ_MB_ = 0.042) at 400 rpm, **1a** (50.0 mmol), **2a** (55.0 mmol), SiO_2_:**1a** (2:1 m/m).
d For 1 mmol: the reaction was placed in a 12 mL stainless-steel vessel with four stainless-steel balls (*N*_MB_ = 4; *d*_MB_ = 10 mm; Φ_MB_ = 0.173) at 400 rpm, **1a** (1.00 mmol), NaN_3_ (2.00 mmol), and AcOH (1.30 mmol). For 50 mmol: the reaction was placed in a 250 mL stainless-steel vessel with 20 stainless-steel balls (*N*_MB_ = 20; *d*_MB_ = 10 mm; Φ_MB_ = 0.042) at 400 rpm, **1a** (50.0 mmol), NaN_3_ (77.0 mmol), and AcOH (260.0 mmol).

The substrate scope of aromatic amines was evaluated, and the results are presented in Scheme 3. For pairs of products **3b–3c**/**3d–3e**/**3f–3g** with the same substituent at *para* and *ortho* positions, there is a decrease in the yields for *ortho-*substituted products. It could be explained by the steric effect of the *ortho* substituent, which makes these anilines less nucleophilic, inhibiting approximation to the electrophilic site of **1**. Anilines with weak electron-withdrawing groups such as carboxylic acid **2h** and carboxylic methyl ester **2k** provide the 1,4-addition products **3h** and **3k** in moderate 60% and 52% yields, respectively. In attempts to synthesize bis-2-amino-1,4-naphthoquinones by a bidirectional reaction, *o*-dianisidine **2l** was employed with 2 equiv of **1** in optimized conditions. However, only the monosubstituted product **3l** was isolated in 88% yield.

**Scheme 3:**
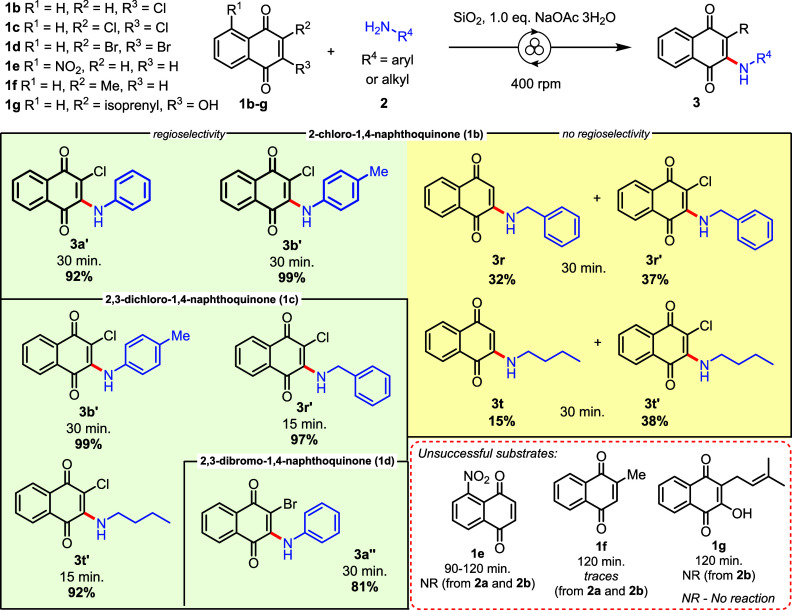
Naphthoquinone Scope for 2-Amino-1,4-naphthoquinones^a^ ^b^

a The reactions were placed in a 12 mL stainless-steel vessel with four stainless-steel balls (*N*_MB_ = 4; *d*_MB_ = 10 mm; Φ_MB_ = 0.173) at 400 rpm, **1** (0.32 mmol), **2** (0.35 mmol), NaOAc·3H_2_O (0.32 mmol), and 500 mg of SiO_2_ (SAG).
b Isolated yields.

Analysis of ^1^H NMR of **3l** confirmed product formation, which showed two signals for methoxy groups in 3.94 and 3.88 ppm, NH and NH_2_ signals in 8.64 and 4.90 ppm, respectively, and one olefinic hydrogen from the quinone moiety in 5.86 ppm. ^13^C NMR is consistent with ^1^H NMR, showing 24 distinct carbons in the structure, including the carbonyl at 182.7 and 182.1 ppm and the methoxy groups at 56.4 and 56.0 ppm (see the Supporting Information for **3l**, Figures S46–S49). In general, the mechanochemical approach to 2-(aryl)­amino-1,4-naphthoquinones afforded satisfactory yield in comparison to other reported methods.^29–42,53^

Limitations were observed in this methodology, such as the reaction with *m*-aminophenol **2p** and heteroaromatic amines **4a**, **4b**, and **5** whose reactions formed many byproducts (Scheme 1). Besides, anilines with strong electron-withdrawing groups in the aromatic ring, such as nitro (**2m**, **2n**, and **2o**), did not react. This fact is in accordance with the literature, wherein weak nucleophilic amines such as nitroanilines and fluoranilines give very low yields, long time reactions, or there is no reaction.^40,58^

In an attempt to expand the scope, a few representative alkylamines were evaluated, whose products are known by other synthetic methods. In this way, product **3q** from cyclohexylamine was isolated in low 34% yield, even with reactant **1a** being consumed (Scheme 2A, Condition A). Therefore, a new condition was evaluated employing 2 equiv of acetic acid; however, the yield decreased to 21% (Scheme 2A, Condition B). A condition without base or acid demonstrated comparable results, obtaining **3q** with 33% yield (Scheme 2, see Conditions A and C). Thus, this simplest condition was extended to investigate other alkylamines. Benzylamine **2r** and morpholine **2s** were selected to expand the scope (Scheme 2, Condition C). In addition, glycine and ß-alanine were tested, but a complex mixture had formed. As a general trend to the mechanochemical synthesis of compounds **3**, aryl amines were more successfully applied than alkyl ones in the tested condition. As a consequence, to access 2-alkylamino-1,4-naphthoquinones, earlier methodologies are synthetically more adequate and should be considered as an alternative.^14,41,42,46^

The potential of solvent-free multigram synthesis of mechanochemistry is a great attractive aspect toward sustainability. Thus, the reaction scale-up was evaluated to model compound **3a** from 0.32 to 50 mmol of reagents, which corresponded to 160 times increase, Scheme 2B. This needed a new reaction design conducted in a 125 mL stainless-steel jar with *N*_MB_ = 10, *d*_MB_ = 10 mm, Φ_MB_ = 0.042, and proportion of SAG SiO_2_:**1a** (2:1 m/m). However, mechanochemical parameters were unoptimized, such as the milling-ball filling degree and SAG proportion. Yield of **3a** decreased from 98% to 61%, and still represents a scale-up of 100 times in terms of obtained product mass, with imposed limitation of purification by chromatography column, Figure S1.

Besides unoptimized mechanochemical conditions, yield decrease in scale-up should be associated with the influence of atmospheric oxygen also, because it was demonstrated that the mass transfer of oxygen during the reaction influences the outcome of the reaction yield of scale-up for **3a**. When the reaction was performed without oxygen atmosphere, the yield decreased, even in the presence of Cu­(OAc)_2_·H_2_O as an oxidant.^30^ Thus, the efficient oxygen diffusion into the reaction mass is important, and the yield decrease possible explanation in the multigram mechanosynthesis of **3a** there is the mass compaction of the reaction mixture in the jar, which can hinder the insertion and diffusion of oxygen from the air. The oxygen participation is indicated in the proposed mechanism, Scheme 2C.

The plausible mechanism pathway to the reaction of 1,4-naphthoquinone **1a** with amines **2** to access 2-amino-1,4-naphthoquinone **3** is well known by oxidative coupling in which the C­(sp^2^)–H is converted into C­(sp^2^)–N bond by a Michael addition and subsequent oxidation, Scheme 2C. In the considered mechanochemical pathway with silica as SAG, the carbonyl moiety of **1a** may coordinate with acid centers present in the SAG, favoring the attack by the nucleophilic amine. Subsequently, the addition of a weak base should shift the chemical equilibrium to the product, by removing the acid hydrogen of the protonated aniline, precluding the retro-Michael due to the suppression of the good leaving group. In addition, dihydro intermediate oxidation can be realized by atmospheric oxygen or by **1a** itself, and restored **1a** return to reaction, Scheme 2C.

Beyond the aryl and alkylamines investigated herein, the synthesis of the simplest unsubstituted 2-amino-1,4-naphthoquinone **7** was elaborated. Unlike amines as nucleophiles in the reaction with **1a** to give substituted **3**, synthesis of **7** employs NaN_3_ in acid medium as NH_2_ source.^59^ In the described mechanism of **1a** and sodium azide, an acid font is necessary to generated *in situ* hydrazoic acid by a reaction with azide, which attacks the olefinic bond by Michael addition followed by nitrogen gas liberation to obtain **7**.^60^ Considering this mechanistic aspects and under the same mechanochemical condition of **3**, naphthoquinone **1a** and NaN_3_ was reacted with in the presence of excess acetic acid, affording **7** with 73%. Herein again, the scale-up to multigram preparation was elaborated, and **7** could be obtained in 89% yield, representing an increase of 64 times, Scheme 2D. An attempt to using NH_4_OAc (3 equiv) instead of NaN_3_ as NH_3_ source was undertaken in a mechanochemical reaction with **1a**,^57^ under the same parameters of small-scale reaction of Scheme 2D, but no reaction occurred.

Alternatively, the synthesis of N-unsubstituted 2-amino-1,4-naphthoquinone **7** was done under microwave heating with 97% yield, Scheme 2D. Both mechanochemical and MW synthesis were chromatography-free, and each approach has its advantages. While the MW route allows shorter reaction time with a slightly better yield, the mechanochemical synthesis could be done on a higher scale.

The substrate scope with respect to the naphthoquinone component was studied, and the results are described in Scheme 3. Under mechanochemically optimized conditions for **1a**, naphthoquinones **1b**–**g** were evaluated. For 2-chloro-1,4-naphthoquinone **1b** and aromatic amines **2a**–**b**, 2-chloro-3-amino-1,4-naphthoquinones **3a’** and **3b’** were obtained in high yields, while a mixture of compounds **3r–3r’** and **3t–3t’** was formed under modest yields from benzylamine **2r** and butylamine **2t**, respectively. Formation of products **3** *r* **/3t** from aliphatic amines can be rationalized by a path involving Michael addition/HCl elimination, while formation of 3-chloro derivatives **3r’/3t’** comes from the proposed mechanism of Scheme 2C. However, when 2,3-dihalogenated naphthoquinones **1c**–**1d** were the starting material, both aromatic and aliphatic amines afforded a single product (**3b’**/**3r’**/**3t’**/**3a″**) in excellent yields, Scheme 3. On the other hand, under mechanochemical optimized conditions, no reaction was detected for naphthoquinones **1e**, **1f**, and **1g**, and aromatic or aliphatic amines.

Lawsone is a natural product with huge applications in organic synthesis. Inspired by previously known synthesis of hydroxy-juglone from dimethylamino-juglone under hydrolysis condition,^61^ and with a representative set of 2-amino-1,4-naphthoquinones in hand (aryl **3a**–**l**, alkyl **3q**–**s** and **7**), Lawsone **8** was selected as a synthetic target. To this end, 2-amino-1,4-naphthoquinones **3a** and **7** were preferable precursors owing to their availability via multigram preparations herein developed. Stepwise and telescopic syntheses were planned. In the two-step route, **3a** and **7** were submitted to hydrolysis with concentrated HCl under reflux affording Lawsone **8**. The reaction time (6, 8, and 15h) was optimized for both compounds, and 8 h afforded better yield for both, with 81% and 88% from **3a** and **7**, respectively. Once the ideal reaction time was determined, the scale was increased to 25 mmol, and yields decreased to 73% and 47% from **3a** and **7** as starting material, respectively, Scheme 4 (see inserted graphic).

**Scheme 4:**
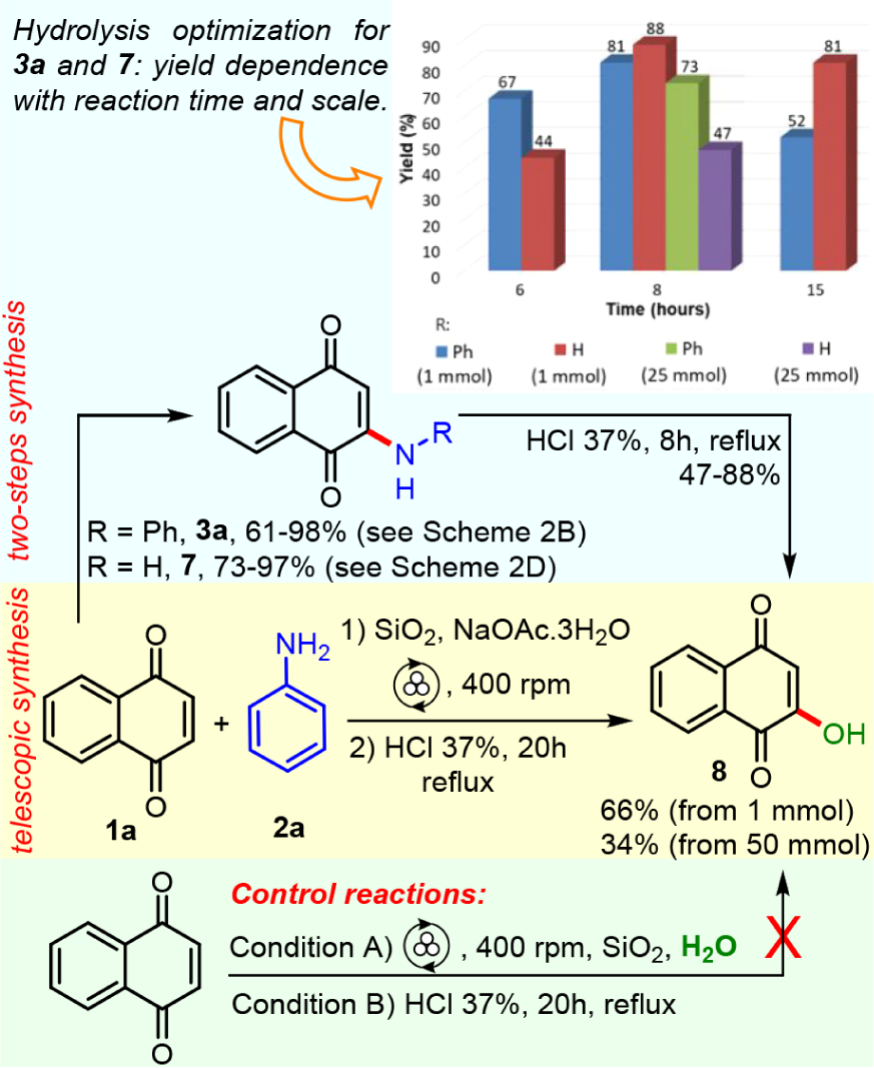
Comparison between Telescopic and Two-Step Syntheses of Lawsone **8** via 2-Amino-1,4-naphthoquinones, Control Reactions, and Hydrolysis Studies for **3a** and **7**

Further applications of Lawsone were envisioned, and gram amounts were necessary. A telescopic approach to Lawsone was elaborated to avoid the isolation and purification of intermediate 2-amino-1,4-naphthoquinones. Safety considerations and higher yield in the hydrolysis step at 25 mmol scale favor **3a** over **7**, because NaN_3_ is employed in excess and dangerous HN_3_ is formed during gram-scale synthesis of **7**, while intermediate **3a** avoids such a scenario. Thus, 1,4-naphthoquinone **1a** and aniline **2a** were submitted to mechanochemical reaction, and all jar content at the end of mechanosynthesis was submitted directly to hydrolysis condition without chromatographic purification of intermediate **3a**, Scheme 4. This hydrolysis reaction was carried out for 20 h, a time somewhat longer than the stepwise synthesis, affording **8** with 66% yield from 1 mmol of **1a**, and 34% from 50 mmol, Scheme 4.

Control reactions with water as nucleophile were tested under mechanochemical and hydrolysis acid conditions with naphthoquinone **1a**, and no trace of Lawsone **8** could be observed, Scheme 4, conditions A–B. Therefore, water as a nucleophile is not able to transform **1a** directly into **8** through the Michael addition reaction, being intermediate 2-amino-1,4-naphthoquinones essential to the synthesis of Lawsone.

In the evaluation of global yields of stepwise and telescopic total syntheses of Lawsone, the reaction scale of 50 mmol of **1a** was common to the three syntheses and taking into consideration, making comparison more realistic: 45% via intermediate **3a** (61%/73% to each step), 42% via **7** (89%/47% to each step), and 34% to the telescopic approach, smaller yield. It should be emphasized that the telescopic synthesis of **8** is preferable because it is more sustainable, avoiding the use of NaN_3_ and is chromatography-free. Although Lawsone is a simple natural product, to the best of our knowledge, this is the first total synthesis involving mechanochemistry in the synthetic route, paving the way to incorporate this enabling technology in the synthetic plans of more complex targets.

In the synthesis of 2-(aryl)­amino-1,4-naphthoquinone we identified an effect of the frequency in the reaction profile. When mechanosynthesis of **3j** was performed at 400 rpm with naphthoquinone **1a** and amine **2j**, a trace amount of a fluorescent byproduct was detected via TLC. Upon changing the frequency to 500 rpm, the 1,4-adduct **3j** yield decreased from 80% to 69% and fluorescent minor product 10-methyl-benzo­[a]­phenoxazine-5-one **6** was isolated in 11% yield, Scheme 1. Furthermore, the synthesis of benzo­[a]­phenoxazines was described in the literature from Lawsone and aminophenol derivatives,^44,62,63^ where **6** was formed with 10% yield. Thus, Lawsone **8** reacted with aniline **2j** under mechanochemical condition and compound **6** was isolated in 26% yield, being **3j** isolated in 74% yield, Scheme 5 (condition A). As mentioned, the selectivity of reaction between **1a** and **2j** was modified by a frequency increase from 400 to 500 rpm, which suggests that yield should be optimized. Studies to maximize the formation of **6** from **8**, and the straightforward synthesis of **6** inverting the selectivity of reaction between **1a** and **2j** via mechanochemistry, are under investigation and will be reported in due course. However, we developed two MW new conditions to synthesize **6** because it was described as promising chemotherapeutics for BRAF V600E COLO205 cells, a refractory mutated cancer.^44^

**Scheme 5:**
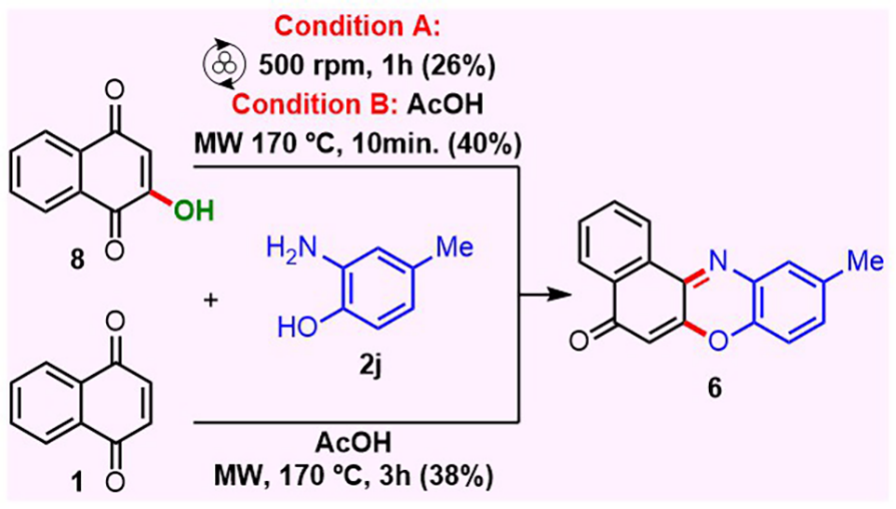
Synthesis of **6** from Lawsone **8** and Naphthoquinone **1a** under Mechanochemical and MW Heating

In the first MW approach, Lawsone **8** reacted with aniline **2j** and compound **6** was isolated as the major product in 40% yield, Scheme 5 (condition B). In the second MW route, an innovative strategy was done by direct reaction of naphthoquinone **1a** with **2j** affording **6** in 38% yield, Scheme 5. Interestingly, TLC analysis indicated several spots in the synthesis from naphthoquinone **1a** and only three spots (**6** major) in the reaction from Lawsone **8**, suggesting that this synthesis is more selective.

## Conclusions

A solvent-free synthesis of 2-amino-1,4-naphthoquinones was developed via mechanochemistry by the reaction of amines with naphthoquinones **1**, and aromatic amines afforded better yields than aliphatic ones. The combination of sodium acetate as base and silica as solid grinding auxiliary was crucial to most cases. To the simplest N-unsubstituted 2-amino-1,4-naphthoquinone **7**, the combination of sodium azide and acetic acid was used to generate HN_3_ *in situ*, affording the NH_2_ derivative. Besides, the same compound could be accessed in high yield via microwave heating. However, the mechanochemistry route has the advantage of multigram (50 mmol) preparation of 2-amino-1,4-naphthoquinone **7** and 2-(phenyl)­amino-1,4-naphthoquinone **3a**. These two compounds were applied in the two-step synthesis of natural product Lawsone via acid hydrolysis. In search of a more sustainable route, the telescopic synthesis of Lawsone was accomplished, being the first and most straightforward total synthesis involving mechanochemistry. In addition, a selectivity dependence was observed with applied frequency in the mechanochemistry reaction of 2-amino-4-methylphenol **2j** with naphthoquinone **1a**, whereby the promising chemotherapeutics 10-methyl-benzo­[a]­phenoxazine-5-one **6** was isolated as a minor product at a high frequency.^44^ This observation led to MW synthesis of **6** from Lawsone and starting material naphthoquinone **1a**, which represents a synthetic innovation to this class of bioactive compound.

## Experimental Section

### General Experimental Information

Commercially available chemicals and solvents were acquired from local suppliers and used without further purification, unless otherwise noted. Melting points were determined on a Microqumica MQAPF 301 hot plate apparatus and were uncorrected. Infrared spectra were recorded as KBr discs or attenuated total reflectance (ATR) on a Shimadzu IR Affinity-1 instrument. ^1^H NMR and ^13^C NMR spectra were recorded on a Bruker Avance III NMR spectrometer (500 MHz for ^1^H and 125 MHz for ^13^C) with tetramethylsilane as an internal standard. Chemical shifts (δ) are reported in parts per million relative to the residual solvent signals, and coupling constants (J) are reported in hertz. The multiplicities are described as brs = broad signal, s = singlet, d = doublet, *t* = triplet, q = quartet, qnt = quintet, sx = sextet, dd = doublet of doublets, dt = doublet of triplets, and *m* = multiplet. High-resolution mass spectra (HRMS) were recorded using electrospray ionization (ESI) (hybrid linear ion trap–orbitrap FT-MS/MS and QqTOF Microtof-QII models). Reagents and materials were of the highest commercially available grade and used without further purification. Mechanochemical reactions were performed in a planetary ball mill Retsch PM100. Microwave heating reactions were performed in a CEM Discover SP using the 10 or 30 mL Pyrex pressure vial for closed-vessel reactions, under the indicated power automatically to reach and maintain the set temperature, specified in each case, with infrared (IR) temperature control and medium stirring speed using stir bars (cylindrical 10 × 3 mm for 10 mL and egg-shaped 19 × 9.5 mm for 30 mL Pyrex pressure vial), default ramp time of 1.5 min. Chromatography columns and reactions were carried out using Merck 70–230 mesh silica gel. Analytical thin-layer chromatography (TLC) was performed using Merck silica gel 60F_254_, 0.2 mm precoated TLC plates. TLC plates were visualized using UV (254 and 366 nm). Spectral data of compounds **3a**–**j**, **3q**–**t**, **3a’**, **3b’**, **3r’**, **3t’**, **3a″**, **6**, **7**, and **8** are in accordance with reported literature and are indicated in each case.

**CAUTION 1** *! Sodium azide is potentially explosive. In the first 30 min of the mechanochemical synthesis of 2-amino-1,4-naphthoquinone* **7** *with sodium azide, the stainless-steel vessel must be opened every 5 min to alleviate the internal pressure by N*_2_ *formation.*

**CAUTION 2** *! In the synthesis of 2-amino-1,4-naphthoquinone* **7** *under microwave heating in a CEM Discover SP reactor, the Pyrex microwave tube must be 30 mL, and do not increase the scale of the reaction. Even to a reaction on a lower scale than that herein described, Pyrex microwave tube must be of 30 mL (never use the 10 mL tube).*

### General Procedure for 2-Arylamino-1,4-naphthoquinones

In a 15 mL beaker, 500 mg of silica gel (Merck 70–230 mesh) as a auxiliary grinding solid, 50 mg (0.32 mmol) of 1,4-naphthoquinone **1a**, 43 mg (0.32 mmol) of sodium acetate trihydrate, and 1.1 equiv of the respective aniline **2a**–**l** were added. The mixture was transferred to a 12 mL stainless-steel vessel with four balls of 10 mm of the same material. The mechanochemical apparatus was configured with intervals every 7 min and 30 s, interval time of 1 s, inversion active, and 400 rpm. The time of milling was varied according to reaction.

#### 2-(Phenyl)­amino-1,4-naphthoquinone **3a** ^30,35,41^

**1a** = 51.6 mg (0.33 mmol), **2a** = 29 μL (0.32 mmol), NaOAc·3H_2_O = 47.8 mg (0.35 mmol), Silica = 501.0 mg. Reaction time −15 min. Purified by silica gel column chromatography eluting with CH_2_Cl_2_. Red solid (77.9 mg, 0.31 mmol, 98%), mp 189–190 °C (Lit.^30^ 190–191 °C). ^1^H NMR (DMSO-*d*_6_, 500 MHz) *δ* 9.22 (1H, s), 8.06 (1H, d, J = 7.5 Hz), 7.95 (1H, d, J = 7.5 Hz), 7.86 (1H, t, J = 7.5 Hz), 7.78 (1H, t, J = 7.5 Hz), 7.45 (2H, t, J = 7.5 Hz), 7.40 (2H, d, J = 7.5 Hz), 7.23 (1H, t, J = 7.5 Hz), 6.11 (1H, s). ^13^C­{^1^H} NMR (DMSO-*d*_6_, 125 MHz) *δ* 183.0, 182.0, 146.6, 138.6, 135.3, 133.1, 130.9, 129.8, 126.6, 125.7, 125.7, 124.2, 102.4. IR (KBr) *v*/cm^–1^ 3317, 1666, 1639,1608, 1597, 1573, 1527, 1446, 1354, 1303, 1246, 991, 775, 725, 709, 686, 624, 551.

#### 2-(4-Methylphenyl)­amino-1,4-naphthoquinone **3b** ^30,41^

**1a** = 49.5 mg (0.32 mmol), **2b** = 37.1 mg (0.35 mmol), NaOAc.3H_2_O = 48.0 mg (0.36 mmol), Silica = 507.7 mg. Reaction time −15 min. Purified by silica gel column chromatography eluting with hexane:AcOEt (9:1) until removes **1a** followed by CH_2_Cl_2_ to obtain **3b**. Red solid (75.8 mg, 0.29 mmol, 92%), mp 198–200 °C (Lit.^30^ 199–200 °C). ^1^H NMR (DMSO-*d*_6_, 500 MHz) δ 9.15 (1H, s), 8.06 (1H, dd, J = 7.5 Hz, 1.0 Hz), 7.94 (1H, dd, J = 7.5 Hz, 1.0 Hz), 7.85 (1H, dt, J = 7.5 Hz, 1.0 Hz), 7.78 (1H, dt, J = 7.5 Hz, 1.0 Hz), 7.27 (2H, d, J = 8.5 Hz), 7.25 (2H, d, J = 8.5 Hz), 6.04 (1H, s), 2.32 (3H, s). ^13^C­{^1^H} RMN (DMSO-*d*_6_, 125 MHz) δ 182.9, 182.1, 146.9, 135.9, 135.3, 135.1, 133.1, 133.0, 130.9, 130.2, 126.6, 125.7, 124.2, 102.1, 21.0. IR (KBr) *v*/cm^–1^ 3325, 1666, 1635, 1604, 1570, 1527, 1350, 1303, 991, 810, 775, 725, 613.

#### 2-(2-Methylphenyl)­amino-1,4-naphthoquinone **3c** ^35^

**1a** = 50.2 mg (0.32 mmol), **2c** = 38 μL (0.35 mmol), NaOAc.3H_2_O = 44.0 mg (0.33 mmol), Silica = 500.3 mg. Reaction time −30 min. Purified by silica gel column chromatography eluting with hexane:AcOEt (98:2) until removes **1a** followed by hexane:CH_2_Cl_2_ (6:4) and CH_2_Cl_2_ to obtain **3c**. Orange solid (60.8 mg, 0.23 mmol, 73%), mp 150–152 °C (Lit.^39^ 146–148 °C). ^1^H NMR (DMSO-*d*_6_, 500 MHz) δ 9.01 (1H, s), 8.07 (1H, d, J = 7.5 Hz), 7.93 (1H, d, J = 7.5 Hz), 7.85 (1H, dt, J = 7.5 Hz, 1.0 Hz), 7.78 (1H, dt, J = 7.5 Hz, 1.0 Hz), 7.37–7.24 (4H, m), 5.32 (1H, s), 2.20 (3H, s), ^13^C­{^1^H} NMR (DMSO-*d*_6_, 125 MHz) δ 182.5, 181.9, 148.3, 136.6, 135.3, 135.0, 133.3, 133.0, 131.5, 131.0, 127.7, 127.4, 127.3, 126.5, 125.8, 101.7, 17.8. IR (KBr) *v*/cm^–1^ 3290, 1678, 1612, 1597, 1562, 1508, 1477, 1462, 1354, 1296, 1157, 1107, 911, 779, 759, 721.

#### 2-(4-Methoxyphenyl)­amino-1,4-naphthoquinone **3d** ^30^

**1a** = 50.1 mg (0.32 mmol), **2d** = 50.6 mg (0.41 mmol), NaOAc.3H_2_O = 46.0 mg (0.34 mmol), Silica = 500.7 mg. Reaction time −15 min. Purified by silica gel column chromatography eluting with hexane:AcOEt (9:1) until removes **1a** followed by CH_2_Cl_2_ to obtain **3d**. Dark red solid (74.7 mg, 0.27 mmol, 85%), mp 156–157 °C (Lit.^30^ 154–155 °C). ^1^H NMR (DMSO-*d*_6_, 500 MHz) δ 9.13 (1H, s), 8.05 (1H, dd, J = 7.5 Hz, 1.0 Hz), 7.94 (1H, dd, J = 7.5 Hz, 1.0 Hz), 7.85 (1H, dt, J = 7.5 Hz, 1.5 Hz), 7.77 (1H, dt, J = 7.5 Hz, 1.5 Hz), 7.30 (2H, d, J = 9.0 Hz), 7.02 (2H, d, J = 9.0 Hz), 5.93 (1H, s), 3.31 (3H, s). ^13^C­{^1^H} NMR (DMSO-*d*_6_, 125 MHz) δ 182.7, 182.1, 157.4, 147.4, 135.3, 133.2, 132.9, 131.1, 130.9, 126.5, 126.07, 125.7, 115.0, 101.5, 55.8. IR (KBr) *v*/cm^–1^ 3224, 1678, 1620, 1604, 1570, 1512, 1354, 1296, 1238, 1176, 1037, 991, 829, 752.

#### 2-(2-Methoxyphenyl)­amino-1,4-naphthoquinone **3e** ^31^

**1a** = 50.0 mg (0.32 mmol), **2e** = 39 μL (0.35 mmol), NaOAc·3H_2_O = 43.8 mg (0.33 mmol), Silica = 502.9 mg. Reaction time −15 min. Purified by silica gel column chromatography eluting with hexane:AcOEt (98:2) until removes **1a** followed by hexane:CH_2_Cl_2_ (1:1) to obtain **3e**. Dark red solid (67.0 mg, 0.24 mmol, 76%) mp 146–148 °C (Lit.^30^ 147–148 °C). ^1^H NMR (DMSO-*d*_6_, 500 MHz) δ 8.63 (1H, s), 8.06 (1H, dd, J = 7.5 Hz, 1.0 Hz), 7.95 (1H, dd, J = 7.5 Hz, 1.0 Hz), 7.86 (1H, dt, J = 7.5 Hz, 1.5 Hz), 7.79 (1H, dt, J = 7.5 Hz, 1.5 Hz), 7.37 (1H, dd, J = 8.0 Hz, 1.0 Hz), 7.27 (1H, dt, J = 8.0 Hz, 1.0 Hz), 7.17 (1H, d, J = 7.5 Hz), 7.05 (1H, dt, J = 8.0 Hz, 1.0 Hz), 5.80 (1H, s), 3.85 (3H, s). ^13^C­{^1^H} RMN (DMSO-*d*_6_, 125 MHz) δ 182.8, 182.0, 152.7, 145.9, 135.4, 133.13, 133.09, 130.7, 127.3, 126.6, 125.8, 124.6, 121.3, 112.6, 103.0, 56.2. IR (KBr) *v*/cm^–1^ 3305, 1670, 1616, 1597, 1573, 1531, 1462, 1262, 1111, 752, 725.

#### 2-(4-Chlorophenyl)­amino-1,4-naphthoquinone **3f** ^30^

**1a** = 50.1 mg (0.32 mmol), **2f** = 45.8 mg (0.36 mmol), NaOAc.3H_2_O = 45.3 mg (0.34 mmol), Silica = 500.8 mg. Reaction time −15 min. Purified by silica gel column chromatography eluting with hexane:AcOEt (9:1) gradually until (6:1) removing three fractions and CH_2_Cl_2_ to obtain **3f**. Red solid (64.7 mg, 0.23 mmol, 73%), mp 266–268 °C (Lit.^30^ 252 °C, 264 °C). ^1^H NMR (DMSO-*d*_6_, 500 MHz) δ 9.28 (1H, s), 8.08 (1H, d, J = 7.0 Hz), 7.96 (1H, d, J = 7.0 Hz), 7.87 (1H, t, J = 7.0 Hz), 7.80 (1H, t, J = 7.0 Hz), 7.50 (2H, d, J = 8.5 Hz), 7.44 (2H, d, J = 8.5 Hz), 6.14 (1H, s). ^13^C­{^1^H} RMN (DMSO-*d*_6_, 125 MHz) δ 183.1, 181.9, 146.3, 137.6, 135.4, 133.2, 133.0, 130.9, 129.7, 129.4, 126.6, 125.8, 125.6, 103.0. IR (KBr) *v*/cm^–1^ 3197, 1678, 1620, 1604, 1570, 1519, 1492, 1404, 1357, 1288, 1122, 1095, 991, 860, 775, 721, 570.

#### 2-(2-Chlorophenyl)­amino-1,4-naphthoquinone **3g** ^52^

**1a** = 50.9 mg (0.33 mmol), **2g** = 51.6 mg (0.41 mmol), NaOAc·3H_2_O = 44.6 mg (0.33 mmol), Silica = 504.5 mg. Reaction time −30 min. Purified by silica gel column chromatography eluting CH_2_Cl_2_ to obtain **3f**. Orange solid (60.7 mg, 0.21 mmol, 65%), mp 146–148 °C (Lit.^37^ 149–151 °C). ^1^H NMR (DMSO-*d*_6_, 500 MHz) δ 9.07 (1H, s), 8.08 (1H, dd, J = 7.5 Hz, 0.5 Hz), 7.94 (1H, dd, J = 7.5 Hz, 0.5 Hz), 7.86 (1H, dt, J = 7.5 Hz, 1.0 Hz), 7.79 (1H, dt, J = 7.5 Hz, 1.0 Hz), 7.63 (1H, dd, J = 7.5 Hz, 1.0 Hz), 7.50–7.36 (3H, m), 5.49 (1H, s). ^13^C­{^1^H} NMR (DMSO-*d*_6_, 125 MHz) δ 182.8, 181.6, 147.0, 135.5, 135.3, 133.2, 133.0, 130.8, 130.7, 130.2, 128.9, 128.8, 128.4, 126.6, 125.9, 103.5. IR (KBr) *v*/cm^–1^ 3332, 1674, 1639, 1620, 1589, 1531, 1465, 1446, 1346, 1296, 1149, 1111, 1095, 991, 775, 740, 721.

#### 2-(2-Amino-1,4-naphthoquinone) Benzoic Acid **3h** ^30^

**1a** = 60.4 mg (0.39 mmol), **2h** = 48.9 mg (0.36 mmol), NaOAc·3H_2_O = 41.7 mg (0.32 mmol), Silica = 514.8 mg. Reaction time −30 min. The solid in the vessel was transferred to an Erlenmeyer flask, 15 mL of EtOH was added, and then submitted to gentle warming (∼70 °C) and stirring. The mixture was filtered and washed with warm EtOH. The solution was evaporated and solubilized in a small volume of water and 8 mol/L HCl was added dropwise until a red solid precipitate was formed (**3h**) that was filtered and washed with H_2_O:EtOH (1:1). Red solid (69.3 mg, 0.24 mmol, 60%), mp 245–251^dec.^ °C (Lit.^30^ 237–240^dec.^ °C). ^1^H NMR (DMSO-*d*_6_, 500 MHz) δ 10.82 (1H, s), 8.09 (1H, d, J = 7.5 Hz), 8.05 (1H, d, J = 7.5 Hz), 7.98 (1H, d, J = 7.5 Hz), 7.89 (1H, t, J = 7.5 Hz), 7.82 (1H, t, J = 7.5 Hz), 7.70–7.69 (2H, m), 7.25–7.22 (1H, m), 6.57 (1H, s). IR (KBr) *v*/cm^–1^ 3074, 3012, 1684, 1612, 1585, 1570, 1531, 1357, 1292, 1211, 1063, 991, 775, 748, 721.

#### 2-(2-Methyl-4-methoxyphenyl)­amino-1,4-naphthoquinone **3i** ^30^

**1a** = 53.7 mg (0.34 mmol), **2i** = 48.9 mg (0.36 mmol), NaOAc·3H_2_O = 41.3 mg (0.31 mmol), Silica = 520.7 mg. Reaction time −30 min. Purified by silica gel column chromatography eluting with CH_2_Cl_2_, removing **2i** residues first. Dark red solid (85.9 mg, 0.29 mmol, 86%), mp 114–117 °C (Lit.^30^ 121–124 °C). ^1^H NMR (DMSO-*d*_6_, 500 MHz) δ 8.91 (1H, s), 8.06 (1H, dd, J = 7.5 Hz, 1.0 Hz), 7.93 (1H, dd, J = 7.5 Hz, 1.0 Hz), 7.85 (1H, dt, J = 7.5 Hz, 1.0 Hz), 7.77 (1H, dt, J = 7.5 Hz, 1.0 Hz), 7.14 (1H, d, J = 8.5 Hz), 6.94 (1H, d, J = 3.0 Hz), 6.87 (1H, dd, J = 8.5 Hz, 3.0 Hz), 5.25 (1H, s), 3.78 (3H, s), 2.16 (3H, s). ^13^C­{^1^H} RMN (DMSO-*d*_6_, 125 MHz) δ 182.4, 182.0, 158.6, 148.9, 136.6, 135.3, 133.4, 132.9, 129.2, 128.6, 126.4, 125.7, 116.4, 112.7, 101.4, 55.7, 18.1. IR (KBr) *v*/cm^–1^ 3284, 1730, 1678, 1612, 1595, 1564, 1512, 1489, 1456, 1354, 1305, 1276, 1242, 1222, 1159, 1122, 1041, 989, 846, 827, 777, 725.

#### 2-(2-Hydroxy-5-methylphenyl)­amino-1,4-naphthoquinone **3j** ^62^

**1a** = 61.4 mg (0.39 mmol), **2j** = 44.7 mg (0.36 mmol), Silica = 518.0 mg. Reaction time −30 min. Purified by silica gel column chromatography eluting with hexane:AcOEt (9:1) gradually until (2:1). The dark red solution was evaporated and solubilized in a small amount of CH_2_Cl_2_ and washed with 4 × 5 mL of 2 mol/L HCl. The organic layer was dried with MgSO_4_ and evaporated to obtain **3j.** Dark red solid (81.4 mg, 0.29 mmol, 80%), mp 186–188 °C (Lit.^62^ 206 °C). ^1^H NMR (DMSO-*d*_6_, 500 MHz) δ 9.71 (1H, s), 8.63 (1H, s), 8.05 (1H, dd, J = 7.5 Hz, 1.0 Hz), 7.95 (1H, dd, J = 7.5 Hz, 1.0 Hz), 7.85 (1H, dt, J = 7.5 Hz, 1.0 Hz), 7.78 (1H, dt, J = 7.5 Hz, 1.0 Hz), 7.08 (1H, d, J = 1.5 Hz), 6.92 (1H, dd, J = 8.0 Hz, 1.5 Hz), 6.87 (1H, d, J = 8.0 Hz), 5.77 (1H, s), 2.25 (3H, s). ^13^C­{^1^H} NMR (DMSO-*d*_6_, 125 MHz) δ 182.7, 182.0, 148.7, 146.0, 135.4, 133.2, 133.0, 130.8, 128.8, 127.7, 126.5, 125.8, 125.1, 125.0, 116.5, 102.9, 20.6. IR (KBr) *v*/cm^–1^ 3292, 1680, 1664, 1616, 1595, 1570, 1516, 1496, 1363, 1330, 1296, 1255, 1224, 1190, 1126, 1116, 991, 835, 817, 779, 725, 669, 582.

#### Methyl 2-(2-Amino-1,4-naphthoquinone) Benzoate **3k**

**1a** = 48.2 mg (0.30 mmol), **2k** = 51.4 mg (0.34 mmol), Silica = 496.1 mg. Reaction time −120 min. Purified by silica gel column chromatography eluting with hexane:AcOEt (9:1) gradually until (8:1). Red solid (48.8 mg, 0.16 mmol, 52%), mp 191–195 °C. ^1^H NMR (CDCl_3_, 500 MHz) δ 10.71 (1H, s), 8.17 (1H, dd, J = 8.0 Hz, 1.0 Hz), 8.12–8.10 (2H, m), 7.76 (1H, dt, J = 7.5 Hz, 1.0 Hz), 7.69 (1H, dt, J = 7.5 Hz, 1.0 Hz), 7.63 (1H, d, J = 8.0 Hz), 7.59 (1H, dt, J = 8.0 Hz, 1.5 Hz), 7.15 (1H, dt, J = 8.0 Hz, 1.0 Hz), 6.72 (1H, s), 3.98 (3H, s). ^13^C­{^1^H} NMR (CDCl_3_, 125 MHz) δ 184.5, 181.9, 167.6, 143.7, 140.8, 134.7, 134.0, 132.9, 132.6, 132.1, 130.6, 126.8, 126.0, 123.1, 120.4, 119.0, 105.6, 52.6. IR (KBr) *v*/cm^–1^ 3233, 3171, 3082, 1707, 1678, 1643, 1612, 1587, 1578, 1535, 1452, 1433, 1348, 1296, 1265, 1220, 1188, 1147, 1120, 1082, 991, 954, 853, 825, 773, 748, 721, 692, 675, 567. HRMS (ESI) *m*/*z* calc for C_18_H_14_NO_4_ [M + H]^+^ 308.0917, found 308.0917.

#### 2-(4’-Amino-3,3′-dimethoxy-[1,1’-biphenyl])­amino-1,4-naphthoquinone **3l**

**1a** = 101.7 mg (0.64 mmol), **2l** = 78.0 mg (0.32 mmol), NaOAc·3H_2_O = 78.0 mg (0.58 mmol), Silica = 1012 mg. Reaction time −15 min. Purified by silica gel column chromatography eluting with hexane:AcOEt (9:1) gradually until (5:1) to obtain **3l**. Dark purple solid (112.2 mg, 0.28 mmol, 82%), mp 171–174 °C. ^1^H NMR (DMSO-*d*_6_, 500 MHz) δ 8.64 (1H, s), 8.07 (1H, d, J = 7.5 Hz), 7.96 (1H, d, J = 7.5 Hz), 7.87 (1H, dt, J = 7.5 Hz, 1.0 Hz), 7.79 (1H, dt, J = 7.5 Hz, 1.0 Hz), 7.37 (1H, d, J = 8.5 Hz), 7.30 (1H, d, J = 1.5 Hz), 7.25 (1H, dd, J = 8.0 Hz, 1.5 Hz), 7.15 (1H, d, J = 1.5 Hz), 7.10 (1H, dd, J = 8.5 Hz, 1.5 Hz), 6.72 (1H, d, J = 8.0 Hz), 5.86 (1H, s), 4.90 (2H, s), 3.94 (3H, s), 3.88 (3H, s). ^13^C­{^1^H} NMR (DMSO-*d*_6_, 125 MHz) δ 182.7, 182.1, 152.9, 147.1, 145.8, 140.3, 138.2, 135.5, 133.2, 133.1, 130.8, 128.1, 126.6, 125.8, 124.6, 124.5, 119.9, 118.6, 114.3, 109.9, 109.6, 103.0, 56.4, 56.0. IR (KBr) *v*/cm^–1^ 3437, 3329, 3275, 3012, 1674, 1620, 1573, 1543, 1523, 1492, 1458, 1446, 1411, 1350, 1296, 1238, 1203, 1114, 1033, 987, 840, 786, 752, 721. HRMS (ESI) *m*/*z* calc for C_24_H_21_N_2_O_4_ [M + H]^+^ 401.1529, found 401.1496.

### Synthesis of 10-Methyl-benzo­[a]­phenoxazine-5-one **6**

In a 15 mL beaker, 1529 mg of silica gel (Merck 70–230 mesh) as an auxiliary grinding solid, 163.3 mg (1.03 mmol) of **1a**, and 126.9 mg (1.03 mmol) of **2j** were added. The mixture was transferred to a 12 mL stainless-steel jar with four balls of 10 mm of the same material. The mechanochemical apparatus was configured with interval every 7 min and 30 s, interval time of 1 s, inversion actived, and 500 rpm for 180 min. The fluorescent yellow spot was separated by silica gel column chromatography eluting with hexane:AcOEt (9:1) to obtain **6** and a dark red band was separated eluting gradually until hexane:AcOEt (4:1) to obtain **3j** with 69% yield (192.1 mg, 0.69 mmol).

#### 10-Methyl-benzo­[a]­phenoxazine-5-one **6** ^44^

Yellow solid (29.9 mg, 0.11 mmol, 11%), mp 194–195 °C (Lit.^44^ 201 °C). ^1^H NMR (CDCl_3_, 500 MHz) δ 8.70 (1H, d, J = 7.0 Hz), 8.30 (1H, d, J = 7.0 Hz), 7.79–7.73 (2H, m), 7.61 (1H, s), 7.28 (1H, d, J = 8.0 Hz), 7.19 (1H, d, J = 8.0 Hz), 6.41 (1H, s), 2.46 (3H, s). ^13^C­{^1^H} NMR (CDCl_3_, 125 MHz) δ 183.8, 151.4, 147.2, 142.0, 135.1, 132.6, 132.4, 132.2, 131.9, 131.7, 131.3, 129.8, 125.8, 124.6, 115.4, 107.1, 20.8. IR (KBr) *v*/cm^–1^ 1693, 1663, 1616, 1578, 1474, 1454, 1354, 1331, 1300, 1269, 1223, 1107, 1053, 980, 906, 840, 787, 717, 694.

### Synthesis of 2-(Phenyl)­amino-1,4-naphthoquinone 3a in 50 mmol Scale

In a 250 mL beaker were added 15.16 g of silica gel (Merck 70–230 mesh) as an auxiliary grinding solid, 7.96 g (50.4 mmol) of **1a**, 6.55 g (48.2 mmol) of sodium acetate trihydrate, both previously pulverized, and 4.8 mL (52.6 mmol) of **2a**. The mixture was transferred to a 125 mL stainless-steel jar with ten balls of 10 mm of the same material. The mechanochemical apparatus was configured with interval every 7 min and 30 s, interval time of 2 min, inversion active and 400 rpm for 45 min. The solid was transferred to column chromatography eluting with CH_2_Cl_2_ to obtain 7.86 g (31.6 mmol, 61%) of **3a**.

### General Procedure for 2-Alkylamino-1,4-naphthoquinones

To a 15 mL beaker, 1000 mg of silica gel (Merck 70–230 mesh) as an auxiliary grinding solid, 1.0 mmol of **1a**, and 1.0 mmol of amine **2q**–**s** were added. The mixture was transferred to a 12 mL stainless-steel jar with four balls of 10 mm of the same material. The mechanochemical apparatus was configured with intervals every 7 min and 30 s, interval time of 1 s, inversion active and 400 rpm. The milling time of 30 min.

#### 2-(Cyclohexyl)­amino-1,4-naphthoquinone **3q** ^41^

**1a** = 183.4 mg (1.16 mmol), **2q** = 126 μL (1.10 mmol), Silica = 1029.3 mg. Purified by silica gel column chromatography eluting with hexane:AcOEt (9:1) to obtain **3q**. Red solid (93.9 mg, 0.34 mmol, 33%), mp 83–85 °C (Lit.^39^ 90 °C). ^1^H NMR (CDCl_3_, 500 MHz) δ 8.10 (1H, dd, J = 8.0 Hz, 1.0 Hz), 8.04 (1H, dd, J = 8.0 Hz, 1.0 Hz), 7.72 (1H, dt, J = 8.0 Hz, 1.0 Hz), 7.61 (1H, dt, J = 8.0 Hz, 1.0 Hz), 5.86 (1H, m), 5.77 (1H, s), 3.33–3.27 (1H, m), 2.06–1.23 (10H, m). ^13^C­{^1^H} NMR (CDCl_3_, 125 MHz) δ 182.7, 182.1, 146.7, 134.7, 133.7, 131.8, 130.6, 126.2, 126.1, 100.7, 51.1, 31.9, 25.5, 24.5. IR (KBr) *v*/cm^–1^ 3340, 3062, 3039, 2927, 2854, 1697, 1670, 1620, 1597, 1570, 1519, 1446, 1350, 1303, 1265, 1249, 1215, 1122, 1099, 1002, 952, 891, 860, 779, 725.

#### 2-(Benzyl)­amino-1,4-naphthoquinone **3r** ^30,41^

**1a** = 154.1 mg (0.97 mmol), **2r** = 108 μL (0.99 mmol), Silica = 1026.8 mg. Purified by silica gel column chromatography eluting with hexane:AcOEt (9:1) to obtain **3r.** Orange solid (121.5 mg, 0.46 mmol, 48%), mp 146–148 °C (Lit.^30^ 155 °C). ^1^H NMR (DMSO-*d*_6_, 500 MHz) δ 8.20 (1H, t, J = 6.5 Hz), 7.99 (1H, d, J = 7.5 Hz), 7.91 (1H, d, J = 7.5 Hz), 7.81 (1H, t, J = 7.5 Hz), 7.73 (1H, t, J = 7.5 Hz), 7.36–7.25 (5H, m), 5.57 (1H, s), 4.45 (2H, d, J = 6.5 Hz). ^13^C­{^1^H} NMR (DMSO-*d*_6_, 125 MHz) δ 182.1, 181.9, 148.9, 137.9, 135.3, 133.5, 132.7, 130.9, 129.0, 127.6, 126.4, 125.8, 100.9, 45.5. IR (KBr) *v*/cm^–1^ 3333, 3059, 3032, 2920, 2850, 1681, 1597, 1562, 1504, 1438, 1361, 1338, 1303, 1257, 1122, 1029, 1006, 945, 844, 783, 729.

#### 2-(4-Morpholinyl)­amino-1,4-naphthoquinone **3s** ^35^

**1a** = 163.2 mg (1.03 mmol), **2s** = 93 μL (1.07 mmol), Silica = 1008.7 mg. Purified by silica gel column chromatography eluting with hexane:AcOEt (9:1) to remove **1a** and hexane:AcOEt (7:1) to obtain **3s**. Orange solid (147.4 mg, 0.61 mmol, 61%), mp 152–153 °C (Lit.^35^ 155–157 °C). ^1^H NMR (CDCl_3_, 500 MHz) δ 8.06 (1H, dd, J = 7.5 Hz, 1.0 Hz), 8.02 (1H, dd, J = 7.5 Hz, 1.0 Hz), 7.72 (1H, dt, J = 7.5 Hz, 1.5 Hz), 7.67 (1H, dt, J = 7.5 Hz, 1.5 Hz), 6.05 (1H, s), 3.88 (4H, m), 3.55 (4H, m). ^13^C­{^1^H} NMR (CDCl_3_, 125 MHz) δ 183.8, 182.9, 153.7, 134.0, 132.7, 132.6, 132.2, 126.7, 125.6, 111.9, 66.4, 49.2. IR (KBr) *v*/cm^–1^ 2974, 2927, 2873, 1674, 1643, 1593, 1566, 1438, 1342, 1303, 1273, 1246, 1211, 1118, 983, 840, 786, 729.

### Mechanochemical Synthesis of 2-Amino-1,4-naphthoquinone 7

In a 15 mL beaker were added 152.4 mg (0.96 mmol) of **1a** and 133.6 mg (2.05 mmol) of sodium azide. The mixture was transferred to a 12 mL stainless-steel vessel with four balls of 10 mm of the same material and was added 750 μL (13.11 mmol) of acetic acid. The mechanochemical apparatus was configured with intervals every 7 min and 30 s, interval time of 1 s, inversion active and 500 rpm for 60 min. The pasty mixture in the vessel was transferred with the aid of 3 mL of acetic acid and a pipet to a beaker with 20 mL of ice water. The solid was filtered and washed with ice water to obtain 121.1 mg, (0.70 mmol, 73%) of **7**.

### Mechanochemical Synthesis of 2-Amino-1,4-naphthoquinone 7 in 50 mmol Scale

In a 250 mL beaker were added 7.89 g (49.9 mmol) of **1a** and 5.01 g (77.1 mmol) of sodium azide. The mixture was transferred to a 250 mL stainless-steel vessel with 20 balls of 10 mm of the same material and 15 mL (262.3 mmol) of acetic acid was added. The mechanochemical apparatus was configured with interval every 30 min, interval time of 1 s, inversion active and 500 rpm for 3 h. In the first 30 min, the vessel was opened every 5 min to alleviate the internal pressure by the N_2_ formation. The pasty mixture in the reactor was transferred with the aid of 15 mL of acetic acid and a pipet to a beaker with 500 mL of ice water. The solid was filtered and washed with ice water to obtain 7.74 g (44.7 mmol, 89%) of **7**.

### Synthesis 2-Amino-1,4-naphthoquinone 7 under Microwave Heating

A solution of 1.77 g (11.2 mmol) of **1a** and 1.23 g (18.9 mmol) of sodium azide in 20 mL of acetic acid at microwave tube of 35 mL was subjected to microwave heating at 100 °C, 250 psi and 300 W for 50 min. After this time, the reaction mixture was poured into 100 mL of cold water and the precipitate was filtered and washed with cold water to obtain 1.90 g (11.0 mmol, 98%) of **7**.

#### 2-Amino-1,4-naphthoquinone **7** ^64^

Brown solid, mp 194–195 °C (Lit.^59^ 202–204 °C). ^1^H NMR (500 MHz, CDCl_3_) δ 8.08 (1H, d, J = 8.0 Hz), 8.06 (1H, d, J = 8.0 Hz), 7.72 (1H, dt, J = 7.5 Hz, 1.0 Hz), 7.64 (1H, dt, J = 7.5 Hz, 1.0 Hz), 6.00 (1H, s), 5.16 (2H, brs). ^13^C­{^1^H} NMR (125 MHz, CDCl_3_) δ 183.9, 182.0, 148.4, 134.7, 133.5, 132.4, 130.7, 126.6, 126.3, 105.3. IR (KBr) *v*/cm^–1^ 3387, 1685, 1616, 1562, 1473, 1485, 1419, 1365, 1273, 1219, 1126, 987, 833, 779, 725, 659.

### General Procedure for Naphthoquinone Scope Ampliation

In a 15 mL beaker, 500 mg of silica gel (Merck 70–230 mesh) as an auxiliary grinding solid, 0.32 mmol of respective 1,4-naphthoquinone **1b**–**1g**, 0.32 mmol of sodium acetate trihydrate, and 1.1 equiv of the amine **2** were added. The mixture was transferred to a 12 mL stainless-steel vessel with four balls of 10 mm of the same material. The mechanochemical apparatus was configured with intervals every 7 min and 30 s, interval time of 1 s, inversion active and 400 rpm. The time of milling was varied according to the reaction.

#### 2-Chloro-3-(phenylamino)-1,4-naphthoquinone **3a** ^19^ (from 1b)

**1b** = 61.7 mg (0.32 mmol), **2a** = 33 μL (0.35 mmol), NaOAc·3H_2_O = 53.9 mg (0.40 mmol), Silica = 503.2 mg. Reaction time −30 min. The solid in the vessel was transferred to an Erlenmeyer flask, 20 mL of ethyl acetate was added, and the mixture was stirred for 5 min. The mixture was filtered, washed with ethyl acetate, and the solution was evaporated to obtain **3a’**. Red solid (83.2 mg, 0.29 mmol, 92%), mp 197–198 °C (Lit.^19^ 206–207 °C). ^1^H NMR (CDCl_3_, 500 MHz) δ 8.19 (1H, d, *J* = 7.5 Hz), 8.12 (1H, d, *J* = 7.5 Hz), 7.77 (1H, t, *J* = 7.5 Hz), 7.69 [2H: N–H (s), C–H (t, *J* = 7.5 Hz)], 7.35 (2H, t, *J* = 7.5 Hz), 7.22 (1H, t, *J* = 7.5 Hz), 7.09 (2H, d, *J* = 7.5 Hz). ^13^C­{^1^H} NMR (CDCl_3_, 125 MHz) δ 180.7, 177.6, 141.7, 137.6, 135.2, 133.1, 130.0, 128.6, 127.3, 127.1, 125.8, 124.4. IR (ATR): *v*/cm^–1^ 3233, 1674, 1593, 1562, 1508, 1489, 1443, 1331, 1288, 1238, 1138, 1076, 1045, 1018, 922, 802, 787, 756, 718, 691.

#### 2-Chloro-3-(4-methylphenylamino)-1,4-naphthoquinone **3b**’^19^ (from 1b)

**1b** = 62.2 mg (0.32 mmol), **2b** = 39.0 mg (0.36 mmol), NaOAc·3H_2_O = 44.1 mg (0.32 mmol), Silica = 502.2 mg. Reaction time −30 min. The solid in the vessel was transferred to an Erlenmeyer flask, 20 mL of ethyl acetate was added and stirred for 5 min. The mixture was filtered and washed with ethyl acetate, and the solution was evaporated to obtain **3b’**. Purple solid (93.3 mg, 0.31 mmol, 99%), mp 182–187 °C (Lit.^19^ 189–190 °C). ^1^H NMR (CDCl_3_, 500 MHz) δ: 8.18 (1H, d, J = 7.5 Hz), 8.10 (1H, d, J = 7.5 Hz), 7.76 (1H, t, J = 7.5 Hz), 7.69–7.65 (2H, m), 7.15 (2H, d, J = 8.0 Hz), 6.98 (2H, d, J = 8.0 Hz), 2.36 (3H, s). ^13^C­{^1^H} NMR (CDCl_3_, 125 MHz) δ: 180.7, 177.5, 141.8, 135.8, 135.1, 135.0, 134.8, 133.0, 132.8, 130.0, 129.1, 128.0, 127.2, 127.1, 124.5, 114.4, 21.2. IR (ATR): *v*/cm^–1^ 3221, 2959, 2920, 2855, 1674, 1631, 1593, 1562, 1516, 1497, 1327, 1285, 1242, 1138, 1111, 1018, 914, 848, 817, 717.

#### 2-(Benzylamino)-3-chloro-1,4-naphthoquinone 3r’^65^ and 2-(Benzylamino)-1,4-naphthoquinone 3r (from 1b)

**1b** = 67.6 mg (0.35 mmol), **2r** = 40 μL (0.35 mmol), NaOAc.3H_2_O = 65.8 mg (0.48 mmol), Silica = 504.5 mg. Reaction time −30 min. Purified by silica gel column chromatography, eluting with hexane:AcOEt (9:1) to remove **3r’** (37%) and **3r** (29.4 mg, 0.11 mmol, 32%) at last. Red solid (38.9 mg, 0.13 mmol, 37%), mp 112–113 °C (Lit.^65^ 112 °C). ^1^H NMR (CDCl_3_, 500 MHz) δ: 8.17 (1H, d, J = 7.5 Hz), 8.05 (1H, d, J = 7.5 Hz), 7.75 (1H, t, J = 7.5 Hz), 7.65 (1H, t, J = 7.5 Hz), 7.42 – 7.34 (5H, m), 6.25 (1H, brs), 5.08 (2H, d, J = 6.0 Hz). ^13^C­{^1^H} NMR (CDCl_3_, 125 MHz) δ 180.5, 177.0, 144.2, 138.0, 135.1, 132.8, 132.7, 129.9, 129.2, 128.2, 127.8, 127.01, 126.99, 49.1. IR (ATR): *v*/cm^–1^ 3314, 3275, 1674, 1639, 1593, 1566, 1516, 1454, 1443, 1331, 1292, 1250, 1134, 1061, 1026, 818, 802, 791, 752, 718, 698, 679.

#### 2-(Butylamino)-3-chloro-1,4-naphthoquinone 3t’^65^ and 2-(Butylamino)-1,4-naphthoquinone 3t^41,42^ (from 1b)

**1b** = 61.4 mg (0.32 mmol), **2r** = 35 μL (0.35 mmol), NaOAc·3H_2_O = 43.1 mg (0.32 mmol), Silica = 500.7 mg. Reaction time −30 min. Purified by silica gel column chromatography, eluting with hexane:AcOEt (9:1) to remove **3t’** (31.8 mg, 0.12 mmol, 38%) and **3t** (10.6 mg, 0.04 mmol, 15%) at last.

**3t’:** Red solid (31.8 mg, 0.12 mmol, 38%), mp 111–113 °C (Lit.^65^ 110 °C). ^1^H NMR (CDCl_3_, 500 MHz) δ 8.14 (1H, d, J = 7.5 Hz), 8.02 (1H, d, J = 7.5 Hz), 7.71 (1H, t, J = 7.5 Hz), 7.61 (1H, t, J = 7.5 Hz), 6.06 (1H, brs), 3.85 (2H, q, J = 7.0 Hz), 1.67 (2H, qnt, J = 7.5 Hz), 1.44 (2H, sx, J = 7.5 Hz), 0.97 (3H, t, J = 7.5 Hz). ^13^C­{^1^H} NMR (CDCl_3_, 125 MHz) δ 180.7, 177.0, 144.4, 135.1, 134.8, 133.0, 132.5, 129.90, 128.0, 127.0, 126.9, 44.8, 33.2, 20.0, 13.9. IR (ATR): *v*/cm^–1^ 3310, 3275, 2959, 2932, 2870, 1674, 1643, 1597, 1566, 1508, 1454, 1438, 1331, 1292, 1257, 1165, 1130, 1111, 1069, 1007, 821, 806, 791, 718, 679.

**3t:** Red solid (10.6 mg, 0.04 mmol, 15%), mp 102–104 °C. ^1^H NMR (CDCl_3_, 500 MHz) δ 8.10 (1H, d, J = 7.5 Hz), 8.04 (1H, d, J = 7.5 Hz), 7.72 (1H, t, J = 7.5 Hz), 7.61 (1H, t, J = 7.5 Hz), 5.88 (1H, brs), 5.73 (1H, s), 3.18 (2H, q, J = 7.0 Hz), 1.68 (2H, qnt, J = 7.5 Hz), 1.44 (2H, sx, J = 7.5 Hz), 0.97 (3H, t, J = 7.5 Hz). ^13^C­{^1^H} NMR (CDCl_3_, 125 MHz) δ 183.1, 182.1, 148.1, 134.9, 133.9, 132.0, 130.7, 126.4, 126.3, 100.9, 42.4, 30.4, 20.3, 13.8. IR (ATR): *v*/cm^–1^ 3348, 2951, 2924, 2855, 1674, 1628, 1593, 1570, 1512, 1462, 1385, 1346, 1323, 1308, 1273, 1242, 1215, 1153, 1134, 1119, 1091, 976, 829, 775, 729.

#### 2-Chloro-3-(4-methylphenylamino)-1,4-naphthoquinone **3b** ^19^ (from 1c)

**1c** = 77.8 mg (0.34 mmol), **2b** = 41.8 mg (0.39 mmol), NaOAc.3H_2_O = 46.4 mg (0.35 mmol), Silica = 508.5 mg. Reaction time −30 min. The solid in the vessel was transferred to an Erlenmeyer flask, 20 mL of ethyl acetate was added, and stirred for 5 min. The mixture was filtered and washed with ethyl acetate, and the solution was evaporated to obtain 100.8 mg (0.34 mmol, 99%) of **3b’**.

#### 2-(Benzylamino)-3-chloro-1,4-naphthoquinone 3r’^65^ (from 1c)

**1c** = 72.6 mg (0.32 mmol), **2r** = 40 μL (0.35 mmol), NaOAc.3H_2_O = 51.4 mg (0.38 mmol), Silica = 506.8 mg. Reaction time −15 min. The solid in the vessel was transferred to an Erlenmeyer flask, 20 mL of ethyl acetate was added and stirred for 5 min. The mixture was filtered and washed with ethyl acetate, and the solution was evaporated to obtain 92.5 mg (0.31 mmol, 97%) of **3r’**.

#### 2-(Butylamino)-3-chloro-1,4-naphthoquinone 3t’^65^ (from 1c)

**1c** = 74.7 mg (0.33 mmol), **2r** = 35 μL (0.35 mmol), NaOAc·3H_2_O = 48.1 mg (0.36 mmol), Silica = 495.2 mg. Reaction time −15 min. The solid in the vessel was transferred to an Erlenmeyer flask, 20 mL of ethyl acetate was added and stirred for 5 min. The mixture was filtered and washed with ethyl acetate, and the solution was evaporated to obtain 80.0 mg (0.30 mmol, 92%) of **3t’**.

#### 2-Bromo-3-(phenylamino)-1,4-naphthoquinone 3a″ ^19^ (from 1d)

**1d** = 102.1 mg (0.32 mmol), **2a** = 35 μL (0.35 mmol), NaOAc·3H_2_O = 44.8 mg (0.33 mmol), Silica = 501.8 mg. Reaction time −30 min. Purified by silica gel column chromatography, eluting with hexane:AcOEt (9:1) followed by (8:2) to obtain **3a″**. Red solid (86.2 mg, 0.26 mmol, 81%), mp 185–188 °C (Lit.^19^ 193–194 °C). ^1^H NMR (CDCl_3_, 500 MHz) δ: 8.12 (1H, d, J = 7.5 Hz), 8.04 (1H, d, J = 7.5 Hz), 7.71–7.66 (2H, m), 7.62 (1H, t, J = 7.5 Hz), 7.28 (2H, t, J = 7.5 Hz), 7.16 (1H, t, J = 7.5 Hz), 7.04 (2H, d, J = 7.5 Hz). ^13^C­{^1^H} NMR (CDCl_3_, 125 MHz) δ: 180.2, 177.5, 144.3, 137.6, 135.1, 133.1, 132.6, 130.0, 128.7, 127.6, 127.2, 125.9, 124.9, 107.8. IR (ATR): *v*/cm^–1^ 3229, 2955, 2924, 2855, 1670, 1632, 1589, 1555, 1504, 1485, 1443, 1327, 1285, 1249, 1234, 1130, 1076, 1111, 918, 825, 775, 756, 718, 691, 679.

### Synthesis of Lawsone 8 from 2-(Phenyl)­amino-1,4-naphthoquinone 3a

A solution of 244.1 mg (0.98 mmol) of **3a** in 12 mL of concentrated HCl was stirred and reflux for 8 h. After this time, the reaction mixture was poured into 100 mL of cold water and extracted with 4 × 15 mL of CH_2_Cl_2_. The combined organic layer was extracted with small amount of saturated solution of Na_2_CO_3_ until the aqueous layer became colorless. The combined aqueous layer was neutralized with concentrated HCl and extracted with 2 × 15 mL of AcOEt. The new organic layer was dried with MgSO_4_ and evaporated to obtain 138.0 mg (0.78 mmol, 81%) of **8**.

### Synthesis of Lawsone from 2-(Phenyl)­amino-1,4-naphthoquinone 3a in 25 mmol Scale

A solution of 6.27 g (25.2 mmol) of **3a** in 250 mL of concentrated HCl was left under stirring and refluxed for 8 h. After this time, the reaction mixture was poured into 2.5 L of cold water. The solution was separated in two equal amounts, and each one of them was extracted with 5 × 100 mL CH_2_Cl_2_. The combined organic layer was extracted with saturated solution of Na_2_CO_3_ until the aqueous layer became colorless. The solid formed in the aqueous layer was collected together this one. The combined aqueous layer was washed with a small amount of CH_2_Cl_2_, neutralized with concentrated HCl, and extracted with portions of 70 mL of AcOEt until it became colorless. The new organic layer was dried with MgSO_4_ and evaporated to obtain 3.18 g (18.3 mmol, 73%) of **8**.

### Synthesis of Lawsone 8 from 2-Amino-1,4-naphthoquinone 7

A solution of 169.5 mg (0.98 mmol) of **7** in 12 mL of concentrated HCl stirred and refluxed for 8 h. After this time, the reaction mixture was poured into 100 mL of cold water and extracted with 4 × 15 mL CH_2_Cl_2_. The combined organic layer was extracted with a small amount of a saturated solution of Na_2_CO_3_ until the aqueous layer became colorless. The combined aqueous layer was neutralized with concentrated HCl and extracted with 2 × 15 mL of AcOEt. The new organic layer was dried with MgSO_4_ and evaporated to obtain 153.0 mg (0.88 mmol, 88%) of **8**.

### Synthesis of Lawsone 8 from 2-Amino-1,4-naphthoquinone 7 in 25 mmol Scale

A solution of 4.38 g (25.3 mmol) of **7** in 250 mL of concentrated HCl was left under stirring and refluxed for 8 h. After this time, the reaction mixture was poured into 2.5 L of cold water. The solution was separated in two equal amounts, and each one of them was extracted with 5 × 100 mL CH_2_Cl_2_. The combined organic layer was extracted with saturated solution of Na_2_CO_3_ until the aqueous layer became colorless. The solid formed in aqueous layer was collected together with this layer . The combined aqueous layer was washed with a small amount of CH_2_Cl_2_, neutralized with concentrated HCl, and extracted with portions of 70 mL of AcOEt until this one became colorless. The new organic layer was dried with MgSO_4_ and evaporated to obtain 2.06 g (11.8 mmol, 47%) of **8**.

### Telescopic Synthesis of Lawsone 8 via 2-(Phenyl)­amino-1,4-naphthoquinone 3a

In a 15 mL beaker, 1053.8 mg of silica gel (Merck 70–230 mesh) as a solid auxiliary grinding, 161.0 mg (1.02 mmol) of **1a**, 138.1 mg (1.01 mmol) of sodium acetate trihydrate, and 96.0 μL (1.05 mmol) of aniline **2a** were added. The mixture was transferred to a 12 mL stainless-steel vessel with 4 balls of 10 mm of the same material. The mechanochemical apparatus was configured with interval every 7 min and 30 s, interval time of 1 s, inversion active, and 400 rpm for 15 min. The solid in the vessel was transferred to a round-bottom flask with 12 mL of concentrated HCl under stirring and refluxed for 20 h. After this time, silica was removed by filtration and the filtrate was collected over 20 mL of cold water. The cake was washed with 80 mL of water. The combined aqueous solution was extracted with 4 × 15 mL CH_2_Cl_2_. The combined organic layer was extracted with a small amount of saturated solution of Na_2_CO_3_ until the aqueous layer became colorless. The silica cake was washed with a saturated solution of Na_2_CO_3_ and the aqueous layer was combined, neutralized with concentrated HCl, and extracted with 2 × 15 mL of AcOEt. The new organic layer was dried with MgSO_4_ and evaporated to obtain 116.7 mg (0.67 mmol, 66%) of **8**.

### Telescopic Synthesis of Lawsone 8 via 2-(Phenyl)­amino-1,4-naphthoquinone 3a in 50 mmol Scale

In a 250 mL beaker, 15.48 g of silica gel (Merck 70–230 mesh) as solid auxiliary grinding, 8.07 g (51.1 mmol) of **1a**, 6.75 g (49.6 mmol) of sodium acetate trihydrate, and 4.8 mL (52.6 mmol) of aniline **2a** were added. The mixture was transferred to a 125 mL stainless-steel vessel with 10 balls of 10 mm of the same material. The mechanochemical apparatus was configured with interval every 7 min and 30 s, interval time of 1 s, inversion active, and 400 rpm for 45 min. The solid in the vessel was transferred to a round-bottom flask with 350 mL of concentrated HCl under stirring and refluxed for 20 h. After this time, silica was removed by filtration and the filtrate was collected over 2.5 L of cold water. The cake was washed with 1.0 L of water. The combined aqueous solution was fractioned in 5 × 800 mL, and each one was extracted with 3 × 50 mL CH_2_Cl_2_. The combined organic layer was extracted with a small amount of saturated solution of Na_2_CO_3_ until the aqueous layer became colorless. The silica cake was washed with a saturated solution of Na_2_CO_3_ and the aqueous layer was grouped, neutralized with concentrated HCl, and extracted with portions of 50 mL of AcOEt until it became colorless. The organic phase was dried with MgSO_4_ and evaporated to obtain 3.05 g (17.5 mmol, 34%) of **8**.

#### 2-Hydroxy-1,4-naphthoquinone (Lawsone) **8** ^56^

Yellow/orange solid. mp 186–188^dec.^ °C (Lit.^55,56^ 193–195 °C). ^1^H NMR (CDCl_3_, 500 MHz) δ 8.14 (2H, m), 7.82 (1H, t, J = 7.0 Hz), 7.74 (1H, t, J = 7.0 Hz), 6.39 (1H, s). ^13^C­{^1^H} NMR (CDCl_3_, 125 MHz) δ 184.9, 181.9, 156.3, 135.3, 133.1, 132.9, 129.4, 126.7, 126.5, 110.7. IR (KBr): *v*/cm^–1^ 3178, 3074, 2924, 1678, 1639, 1593, 1577, 1458, 1384, 1346, 1284, 1253, 1222, 1176, 1118, 983, 875, 806, 767, 725.

### Synthesis of 10-Methyl-benzo­[a]­phenoxazine-5-one 6 from Lawsone 8 via Microwave Heating

A solution of 225.9 mg (1.30 mmol) of **8** and 135.7 mg (1.10 mmol) of aminophenol **2k** in 5 mL of acetic acid in a vessel of 12 mL was left under microwave irradiation at 170 °C and 300 W for 10 min. After this time, the reaction mixture was poured into 25 mL of ethyl acetate and washed with water (2 × 10 mL) and saturated solution of Na_2_CO_3_ (1 × 10 mL). The organic layer was dried with MgSO_4_, evaporated, and purified by silica gel column chromatography eluting with hexane:AcOEt (9:1) to obtain 116.5 mg (0.45 mmol, 40%) of **6**.

### Synthesis of 10-Methyl-benzo­[a]­phenoxazine-5-one 6 from Lawsone 8 via Mechanochemistry

In a 15 mL beaker, 1501 mg of silica gel (Merck 70–230 mesh) as an auxiliary grinding solid, 174.1 mg (1.00 mmol) of **8**, and 124.7 mg (1.01 mmol) of **2j** were added. The mixture was transferred to a 12 mL stainless-steel jar with four balls of 10 mm of the same material. The mechanochemical apparatus was configured with interval every 7 min and 30 s, interval time of 1 s, inversion actived and 500 rpm for 60 min. The fluorescent yellow spot was separated by silica gel column chromatography eluting with hexane:AcOEt (9:1) to obtain **6** with 26% yield (67.6 mg, 0.26 mmol) and a dark red band was separated by gradually until hexane:AcOEt (4:1) to obtain **3j** with 74% yield (207.4 mg, 0.74 mmol).

### Synthesis of 10-Methyl-benzo­[a]­phenoxazine-5-one 6 from 1,4-Naphthoquinone 1a via Microwave Heating

A solution of 156.4 mg (0.99 mmol) of **1a** and 129.3 mg (1.05 mmol) of aminophenol **2k** in 5 mL of acetic acid in a vessel of 12 mL was left under microwave irradiation at 170 °C and 300 W for 3 h. After this time, the reaction mixture was poured into 100 mL of cold water and extracted with CH_2_Cl_2_ (4 × 10 mL or until the aqueous layer became colorless). The organic layer was evaporated and purified by silica gel column chromatography eluting with hexane:AcOEt (9:1) to obtain 99.5 mg (0.38 mmol, 38%) of **6**.

## Supplementary Material



## Data Availability

The data underlying this study are available in the published article and its online Supporting Information.

## Notes

Selected pictures of experimental procedures and copies of NMR, HRMS, and IV spectra (PDF)
