# Supplementary material for: Mechanochemical Synthesis of 2‑Amino-1,4-naphthoquinones and Telescopic Synthesis of Lawsone
Source: ACS Omega. 2025 Aug 27;10(40):46369–83. doi: 10.1021/acsomega.4c11349 (PMC12529405; doi:10.1021/acsomega.4c11349)
Supplement: Supplementary file 1 [file ao4c11349_si_001.pdf]

SUPPLEMENTARY MATERIAL

Mechanochemical Synthesis of 2-Amino-1,4-naphthoquinones and Telescopic Synthesis of Lawsone

Igor Sande[1], Talita Santana Nascimento[1], Sabrina Martinez[1,2], Sâmia Rocha Lima[1] and Silvio Cunha*[1,2]

[1]Instituto de Química, Universidade Federal da Bahia, Campus de Ondina, 40170-115, Salvador, BA.

[2]Instituto Nacional de Ciência e Tecnologia - INCT em Energia e Ambiente, Universidade Federal da Bahia, Campus de Ondina, Salvador, BA, 40170-290, Brazil.

* To whom correspondence should be addressed. E-mail: silviodc@ufba.br

Table of Contents

Pictures of selected experimental procedures 2

FT-IR, NMR, and HRMS Spectra...4

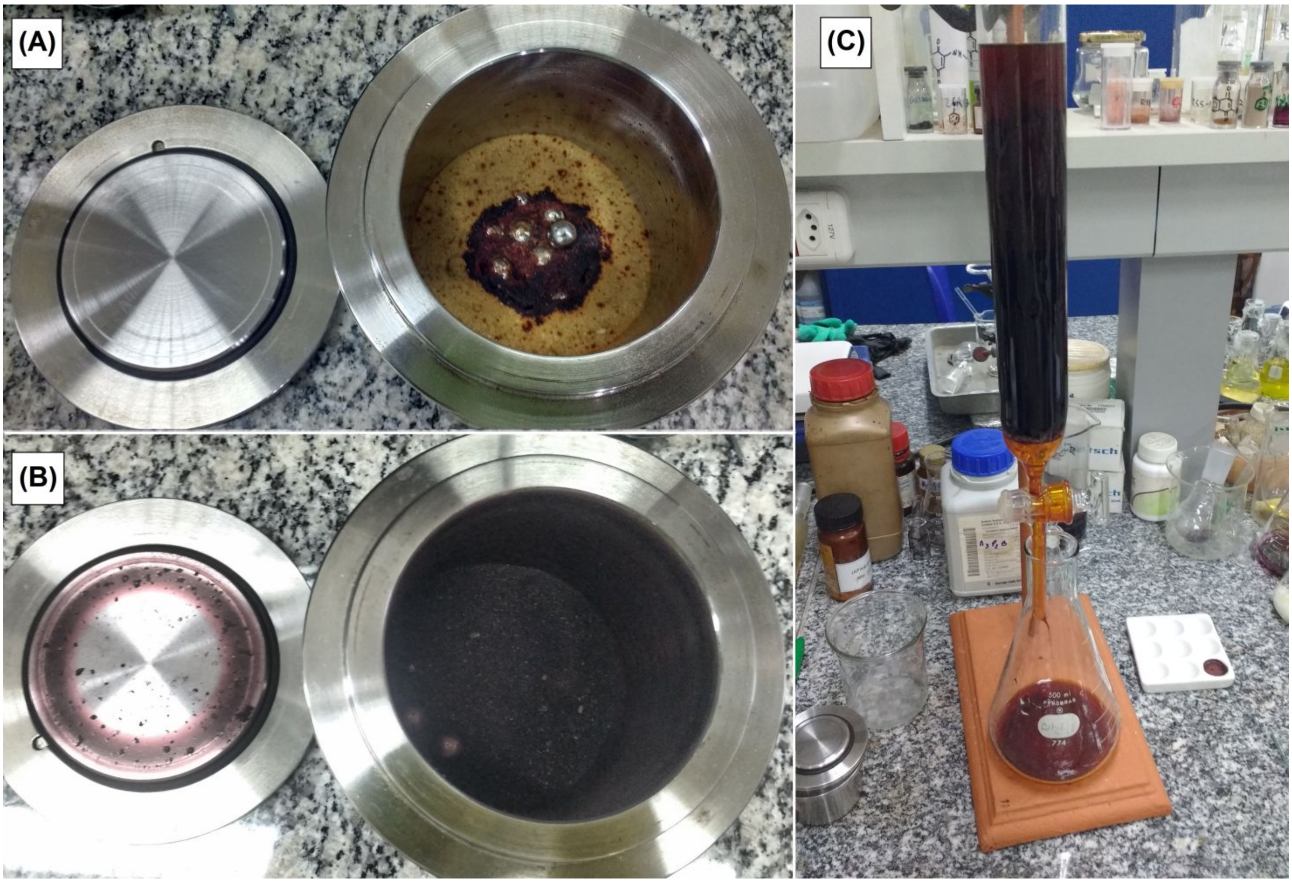

Figure S1. (A) Reagents for multigram synthesis of 2-phenylamino-1,4-naphthoquinone in vessel before milling, (B) visual aspect of content in vessel after milling and (C) purification by column chromatography.

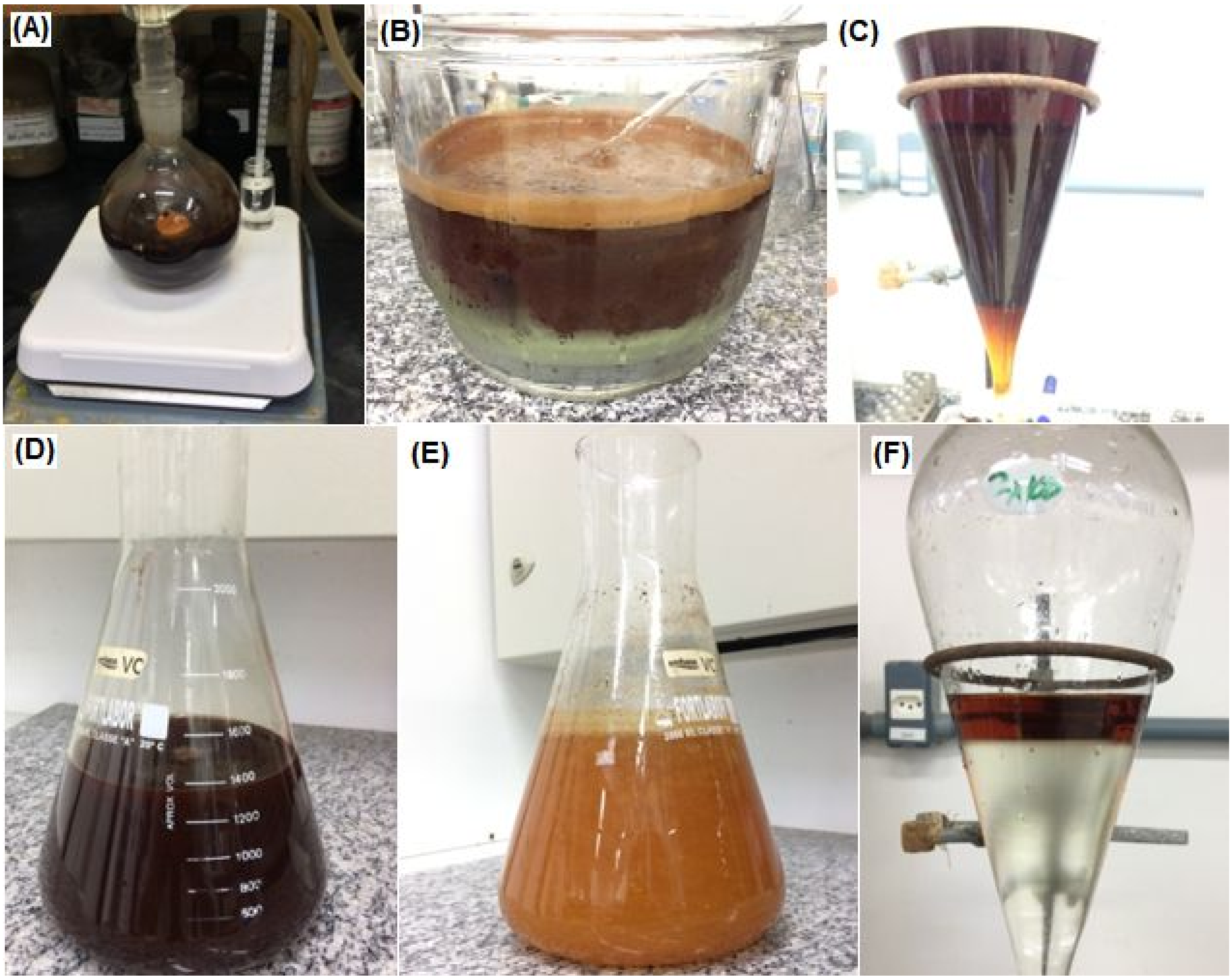

Figure S2. (A) Acid hydrolysis of 2-amino-1,4-naphthoquinone under concentrated HCl (37%), (B) pH adjustment by dilution, (C) extraction with saturated solution of Na_2CO_3, (D) aqueous layer with the lausonate, (E) neutralization with concentrated HCl and (F) extraction with AcOEt.

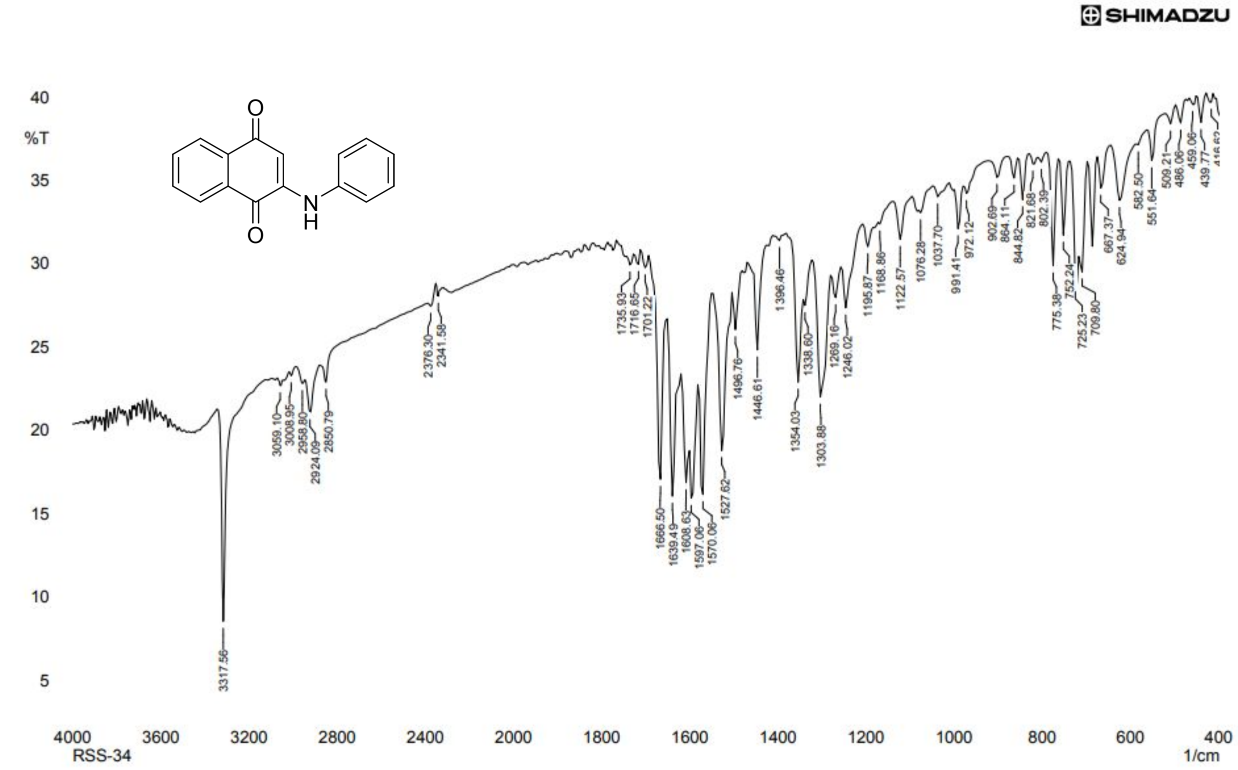

Figure S3. IR (KBr) spectrum of **3a**

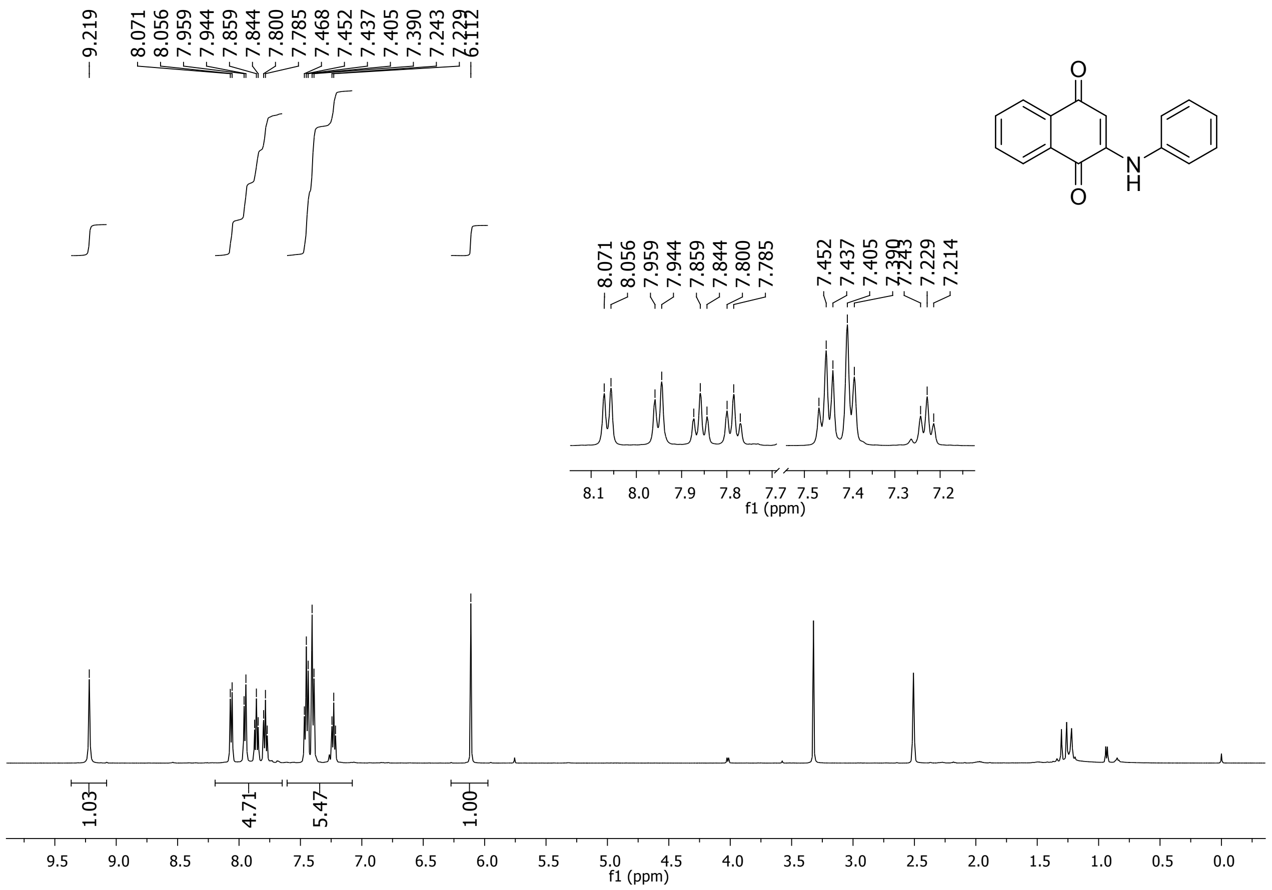

Figure S4. Full ^{1}H NMR DMSO-d_6 spectrum of **3a**

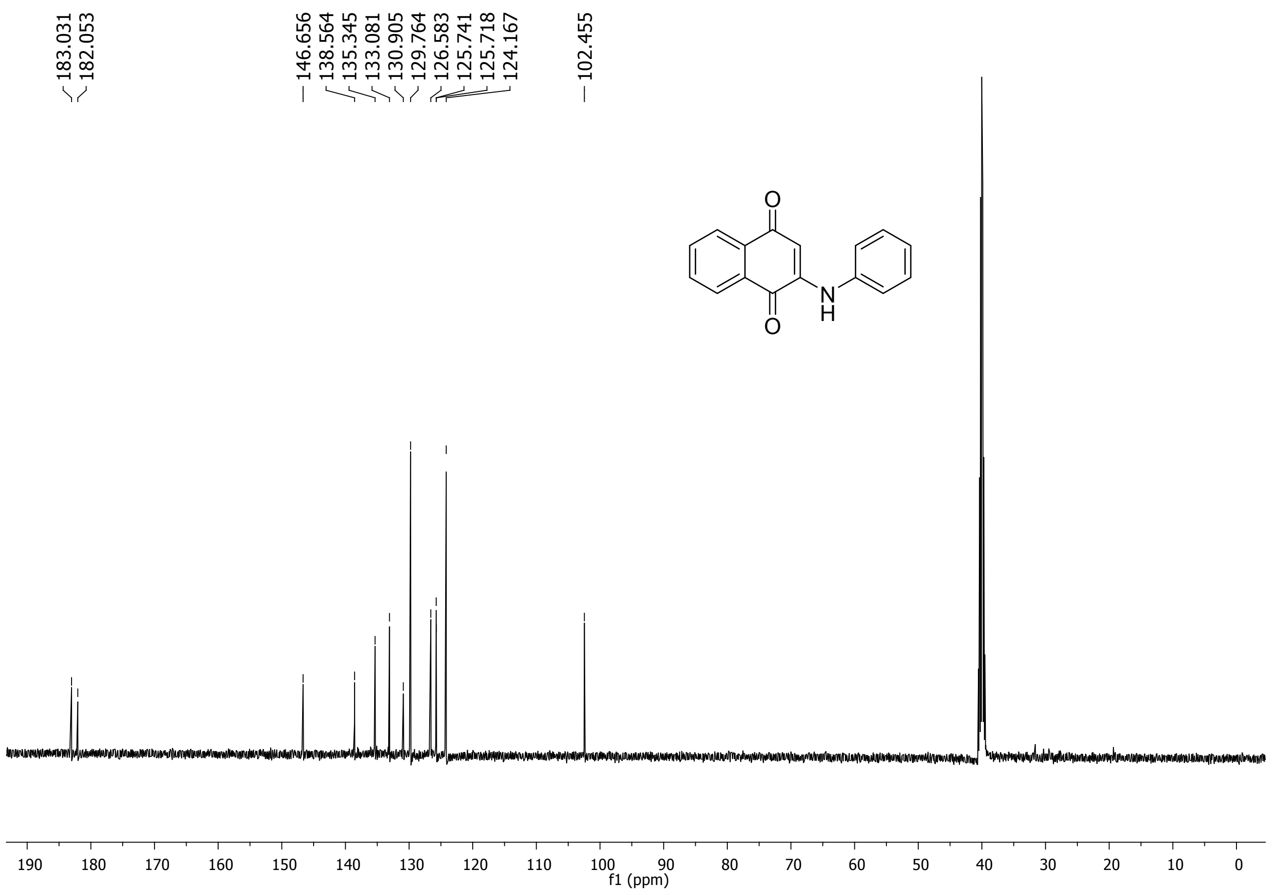

Figure S5. Full $^{13}C\{^{1}H\}$ NMR DMSO-d_6 spectrum of **3a**

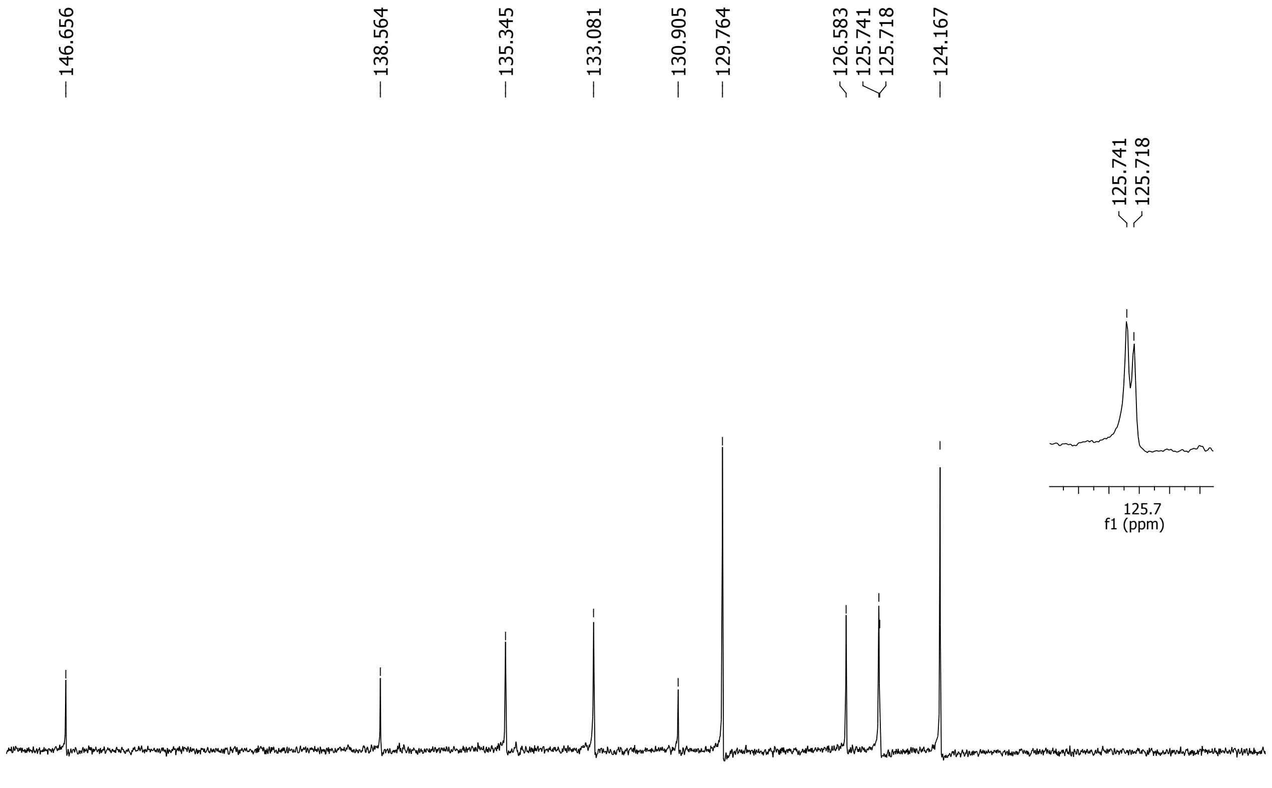

Figure S6. Expanded $^{13}C\{^{1}H\}$ NMR DMSO-d_6 spectrum of **3a**

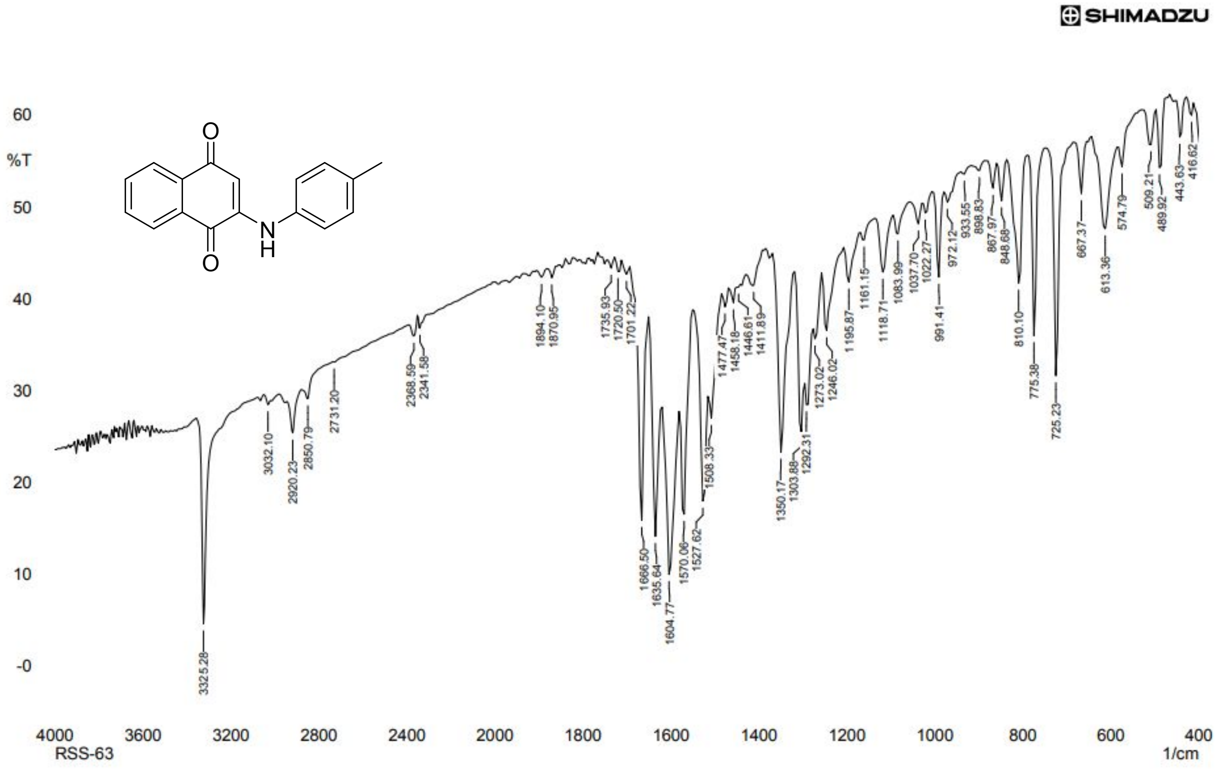

Figure S7. IR (KBr) spectrum of **3b**

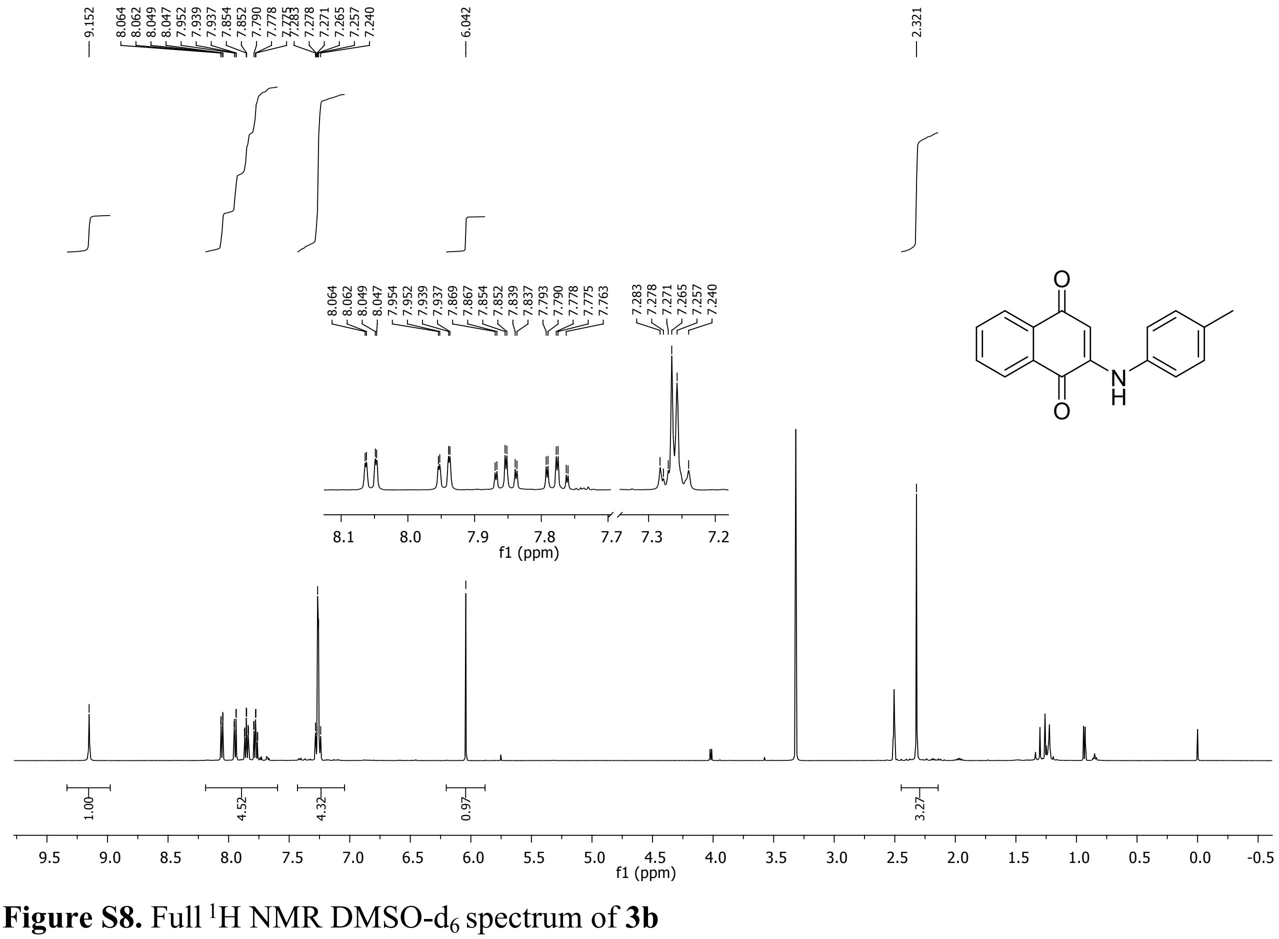

Figure S8. Full ^{1}H NMR DMSO-d_6 spectrum of **3b**

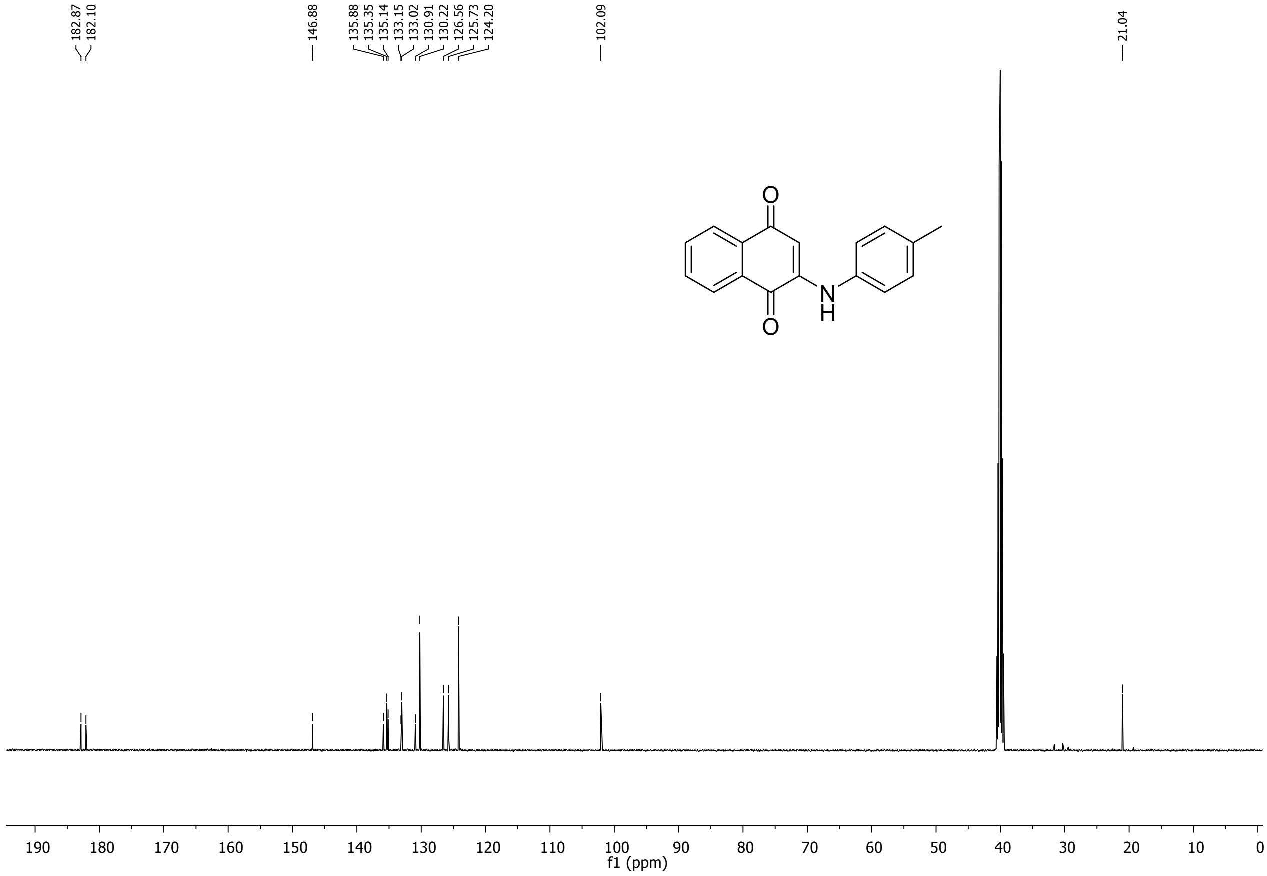

Figure S9. Full $^{13}C\{^{1}H\}$ NMR DMSO-d_6 spectrum of **3b**

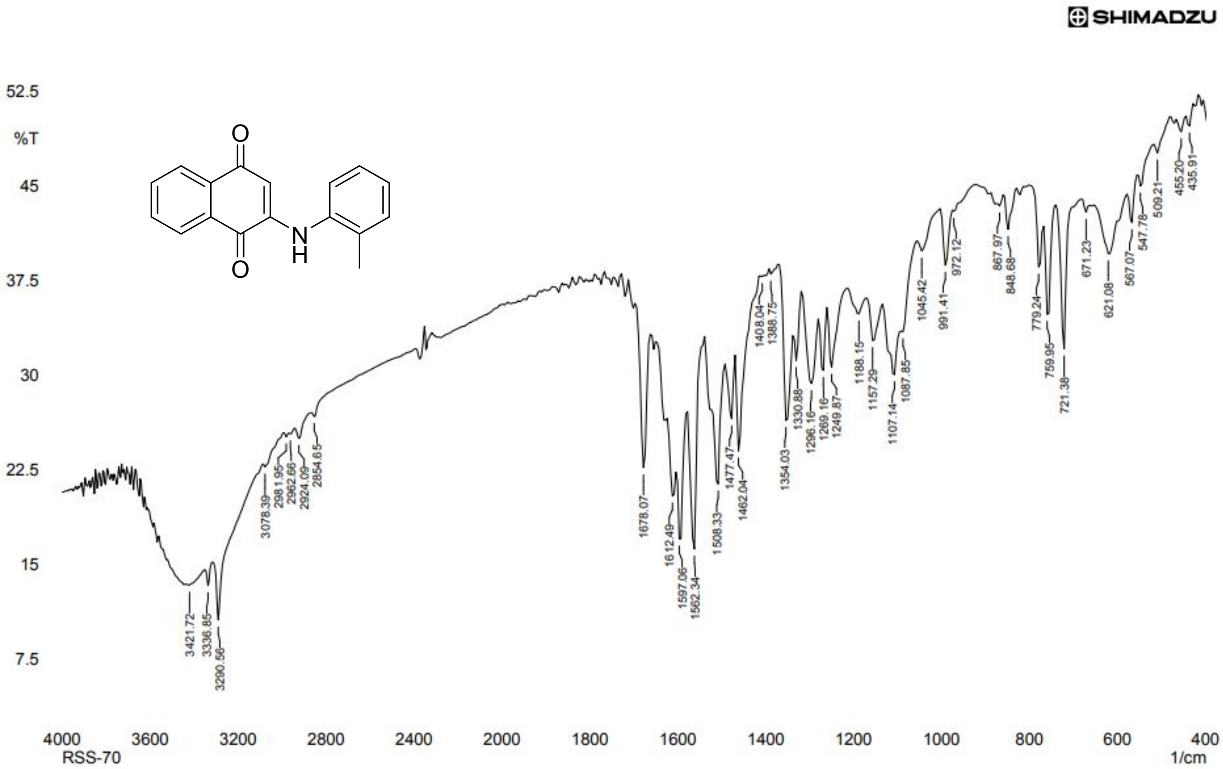

Figure S10. IR (KBr) spectrum of **3c**

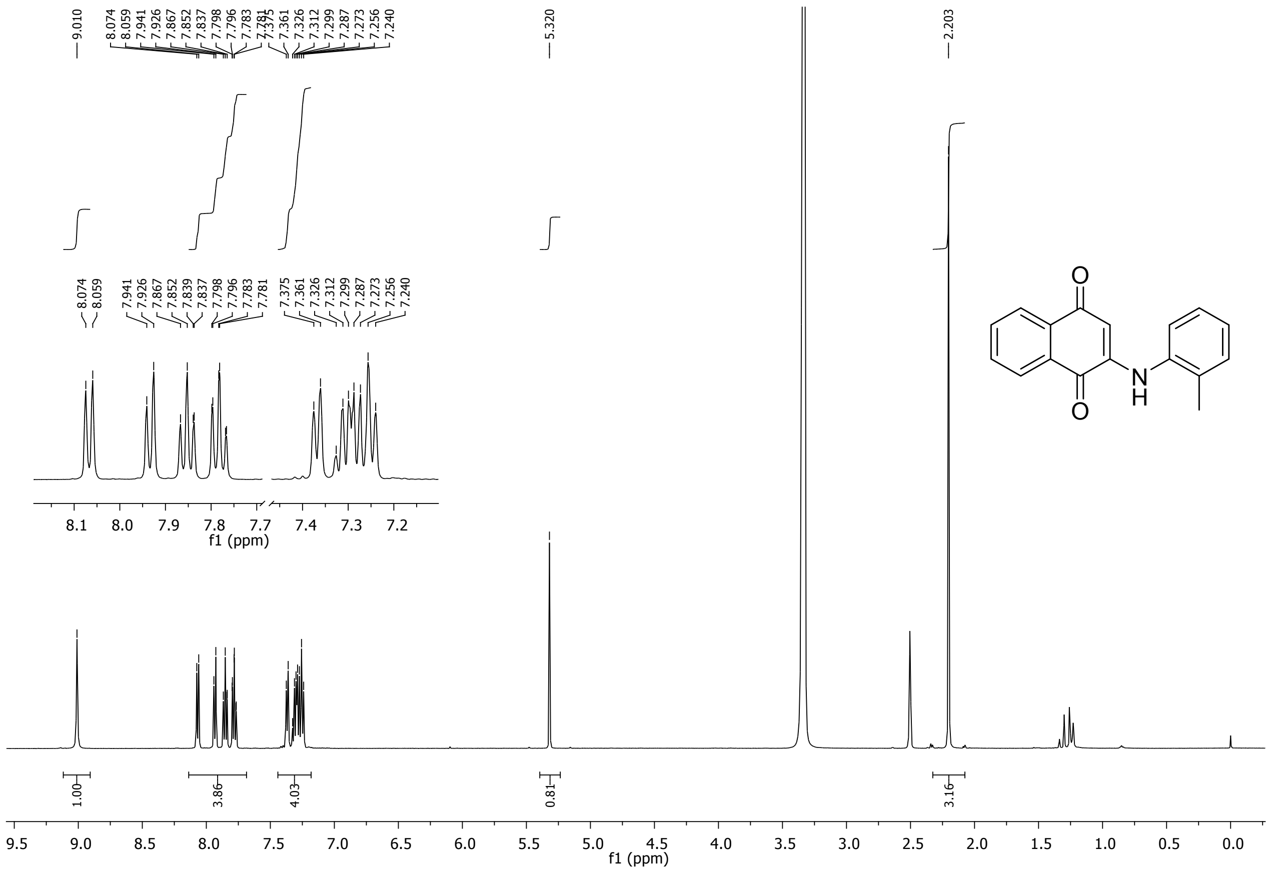

Figure S11. Full ^{1}H NMR DMSO-d_6 spectrum of **3c**

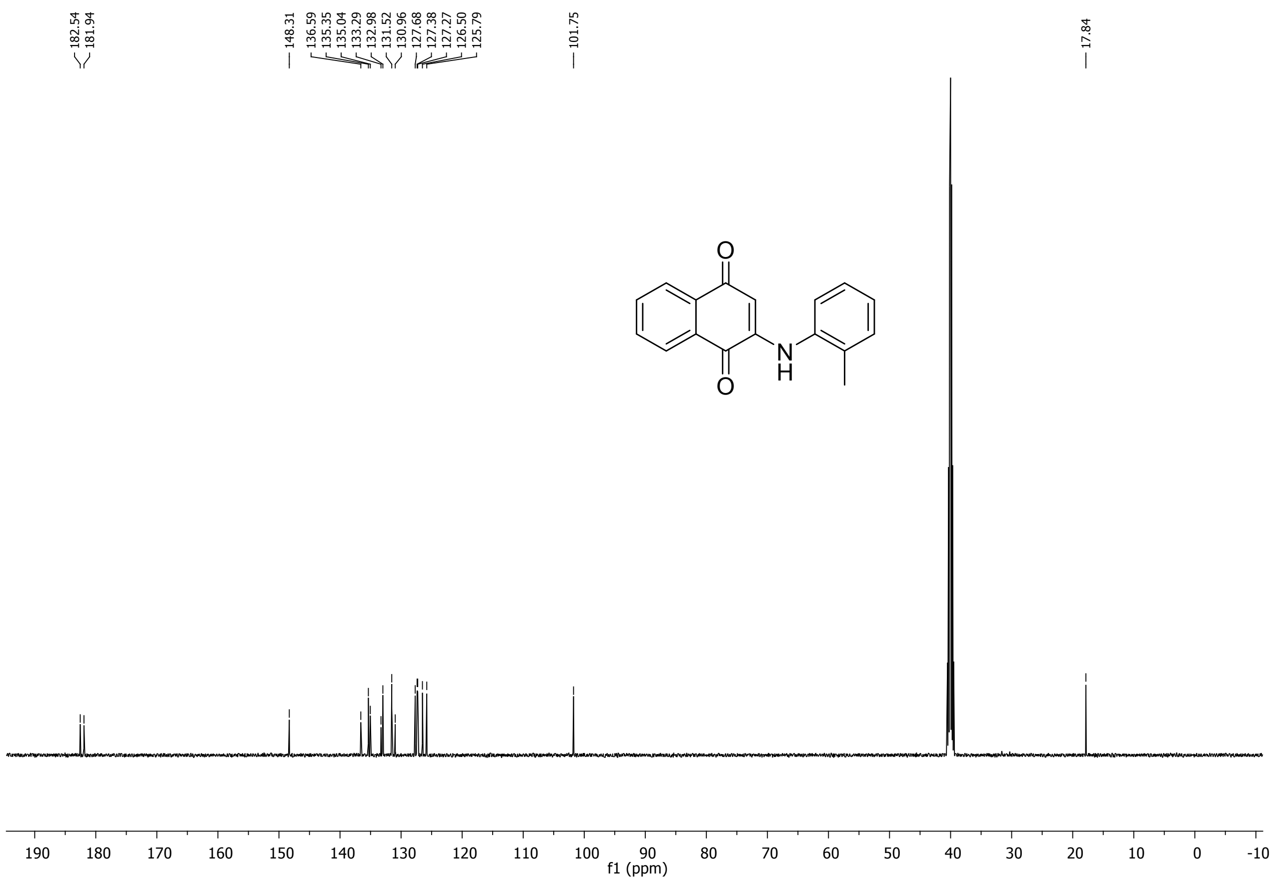

Figure S12. Full $^{13}C\{^{1}H\}$ NMR DMSO-d_6 spectrum of **3c**

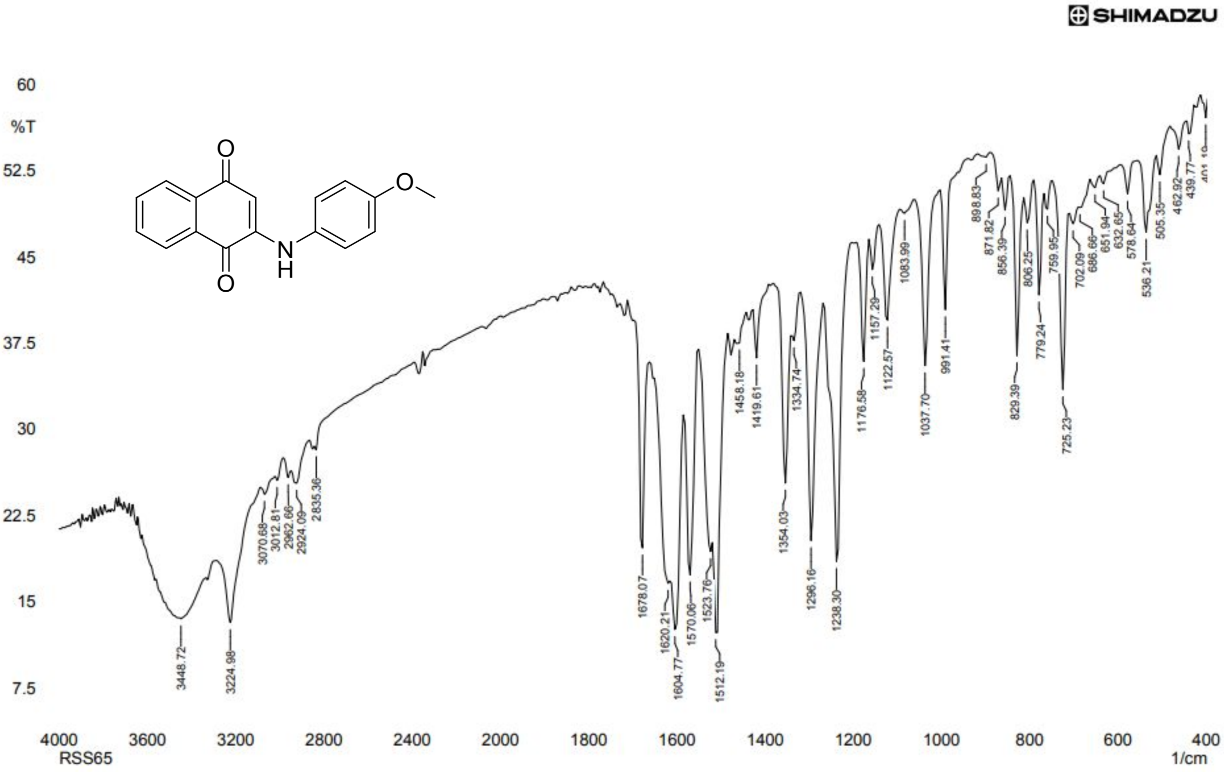

Figure S13. IR (KBr) spectrum of **3d**

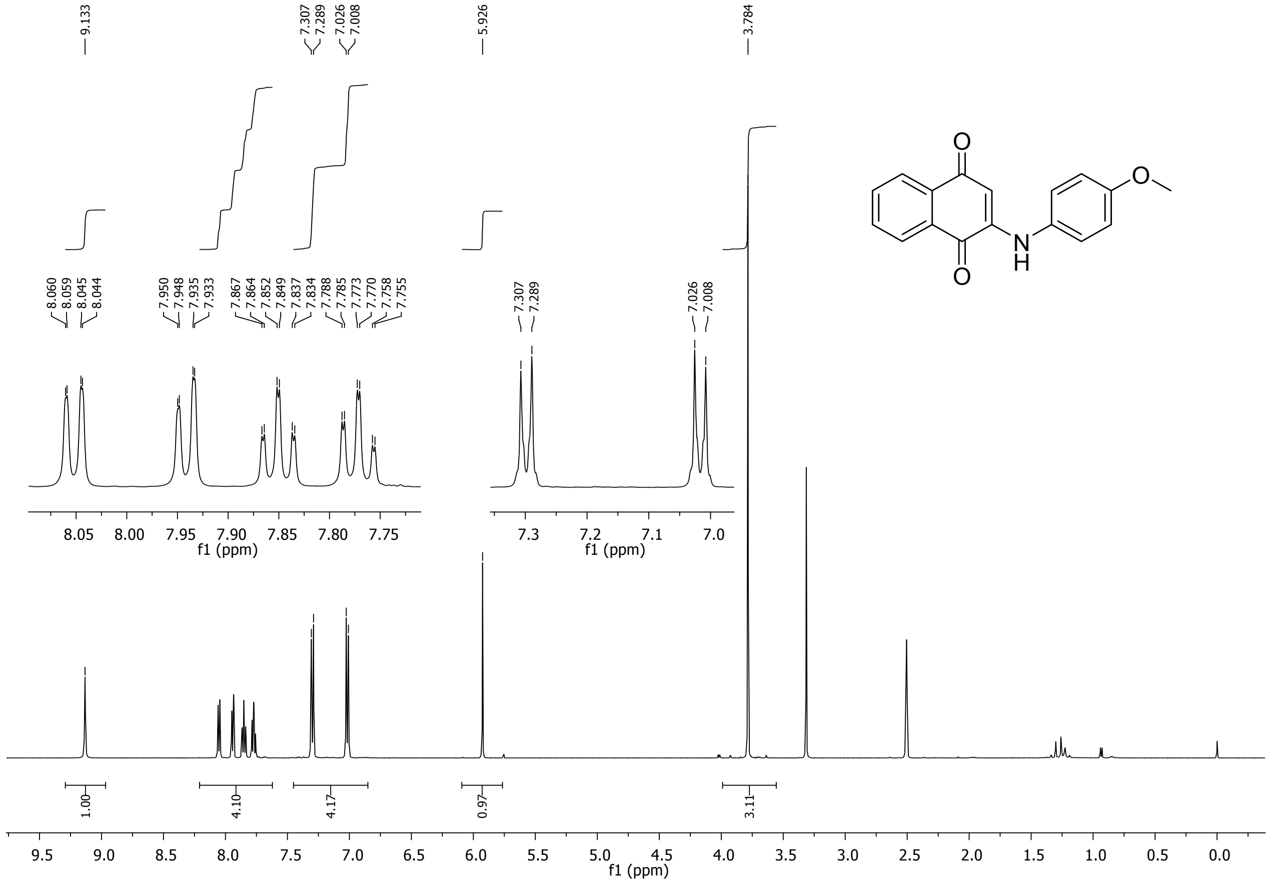

Figure S14. Full ^{1}H NMR DMSO-d_6 spectrum of **3d**

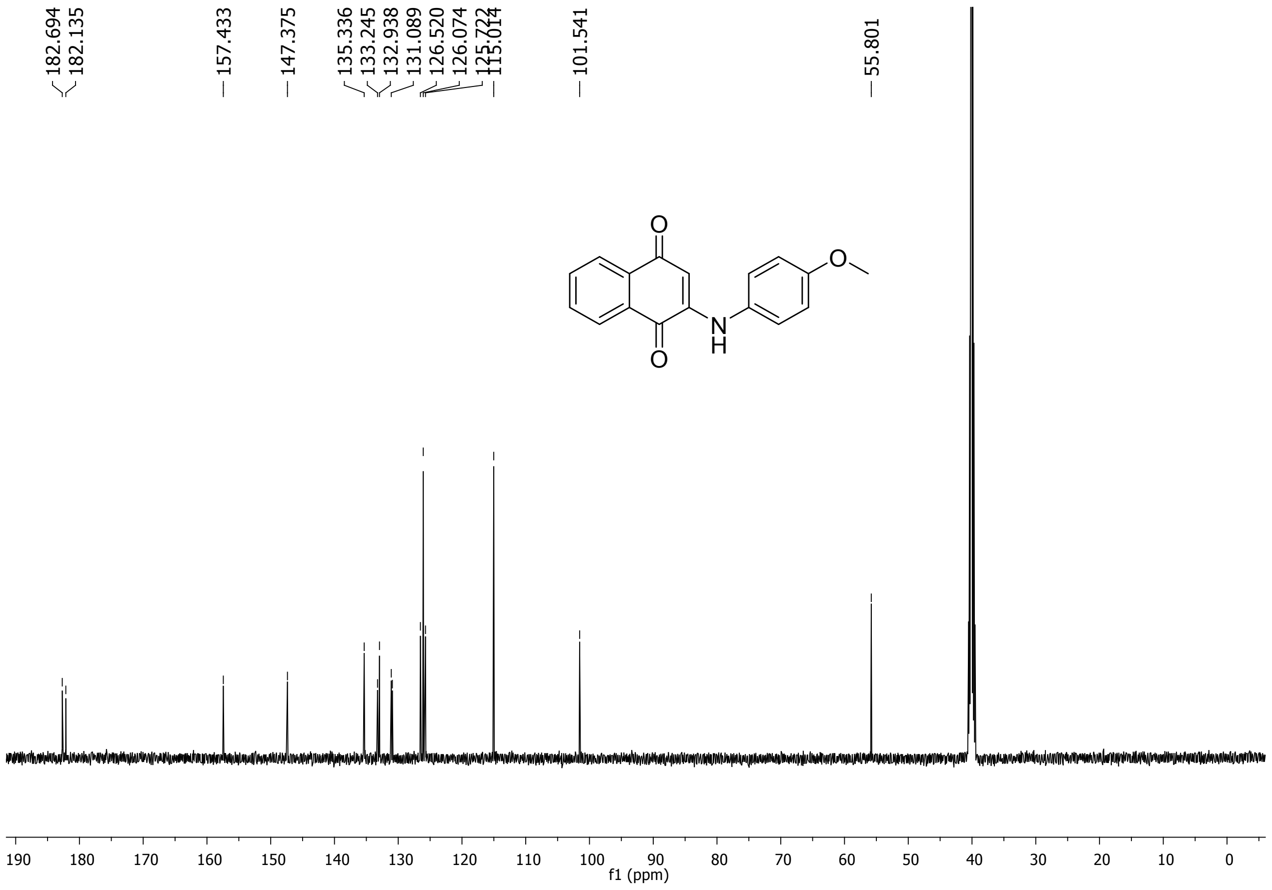

Figure S15. Full $^{13}C\{^{1}H\}$ NMR DMSO-d_6 spectrum of **3d**

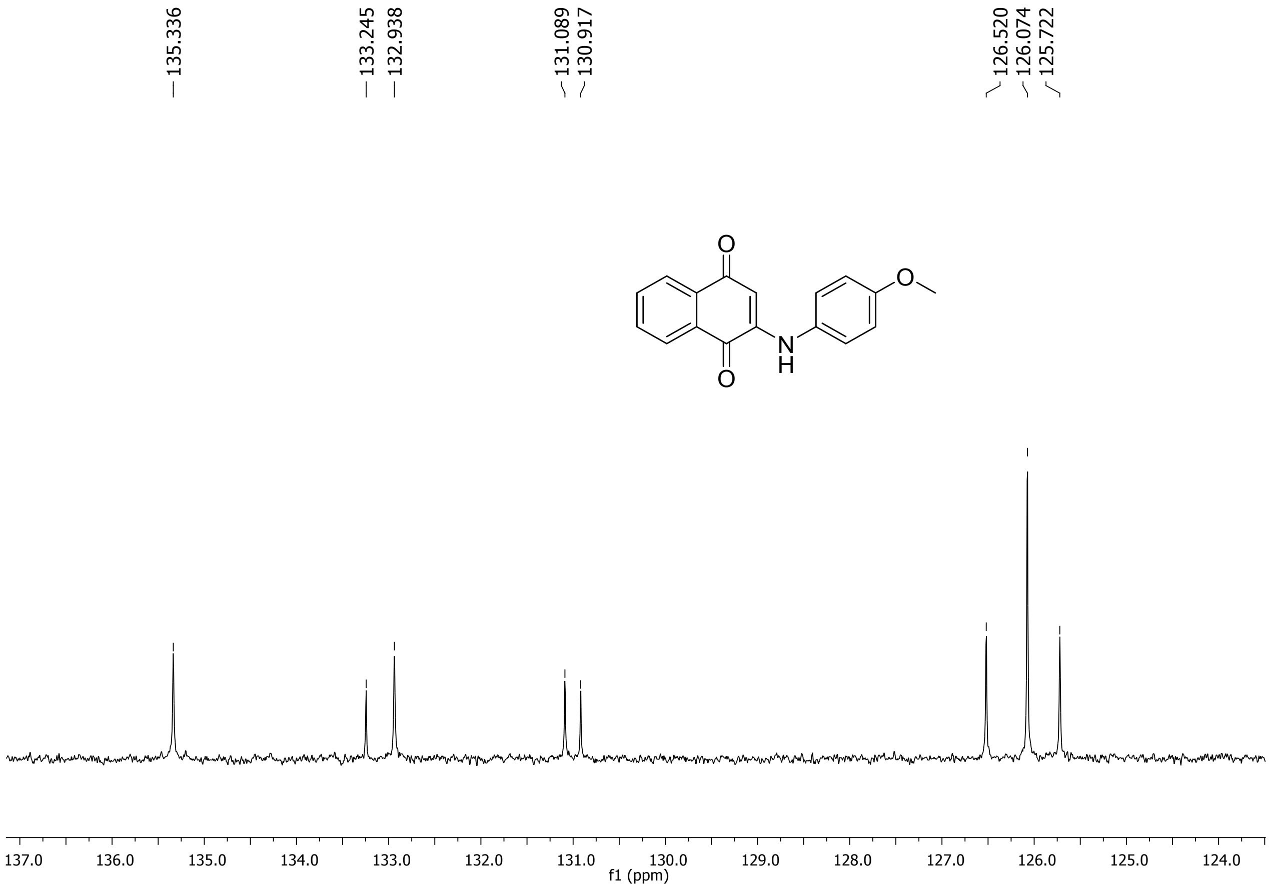

Figure S16. Expanded $^{13}C\{^{1}H\}$ NMR DMSO-d_6 spectrum of **3d**

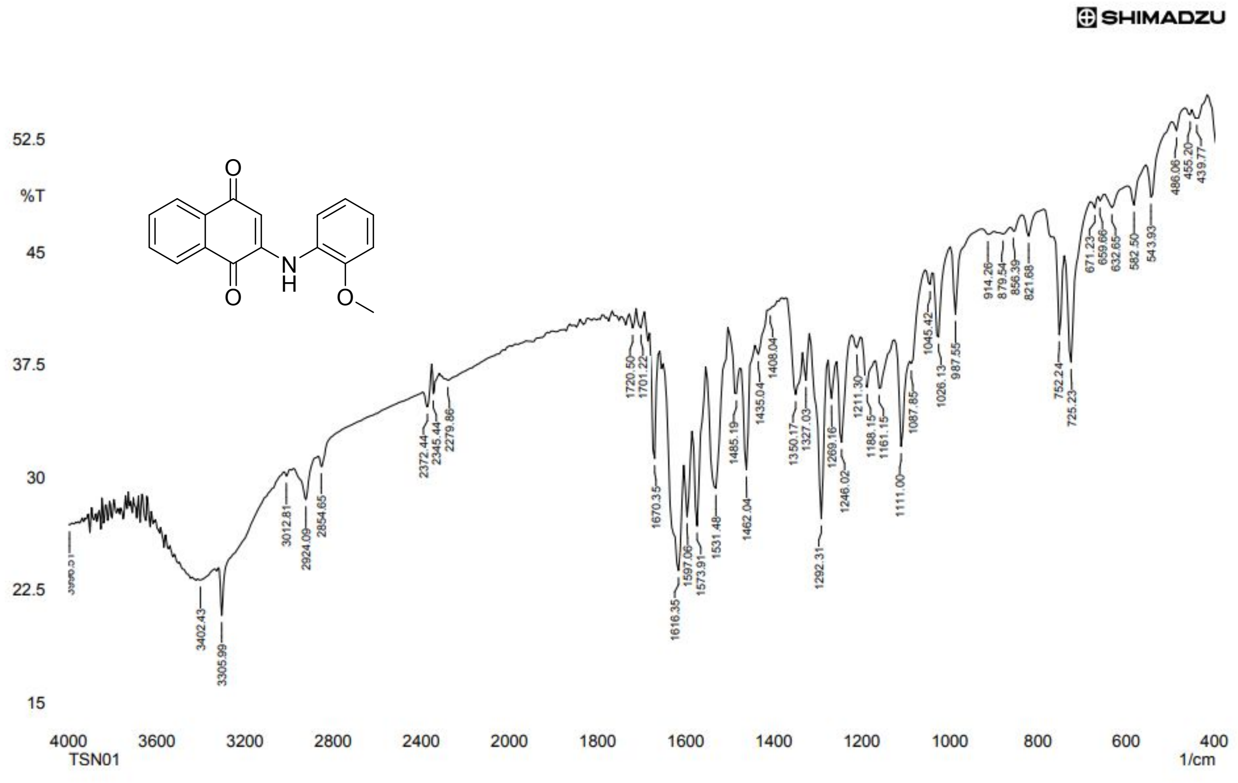

Figure S17. IR (KBr) spectrum of **3e**

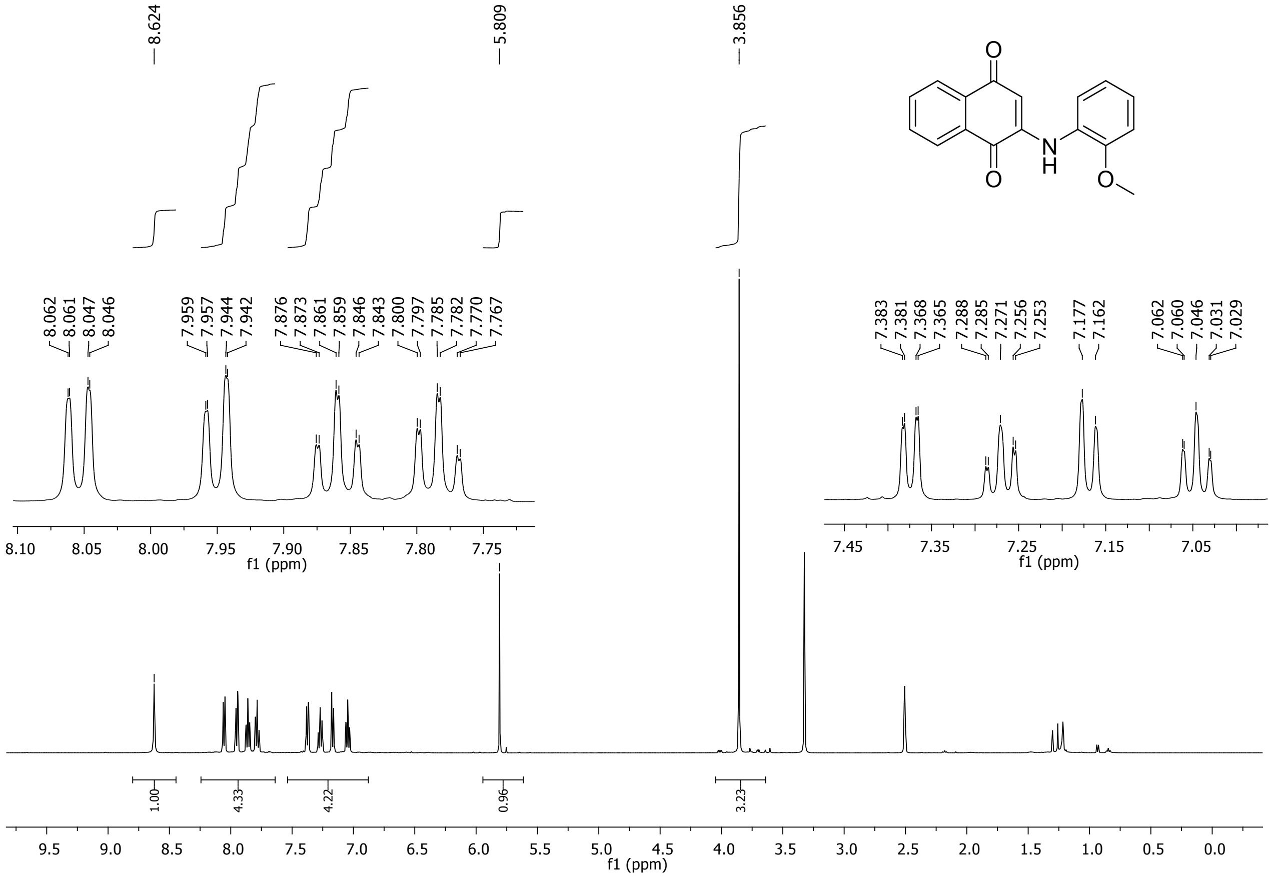

Figure S18. Full ^{1}H NMR DMSO-d_6 spectrum of **3e**

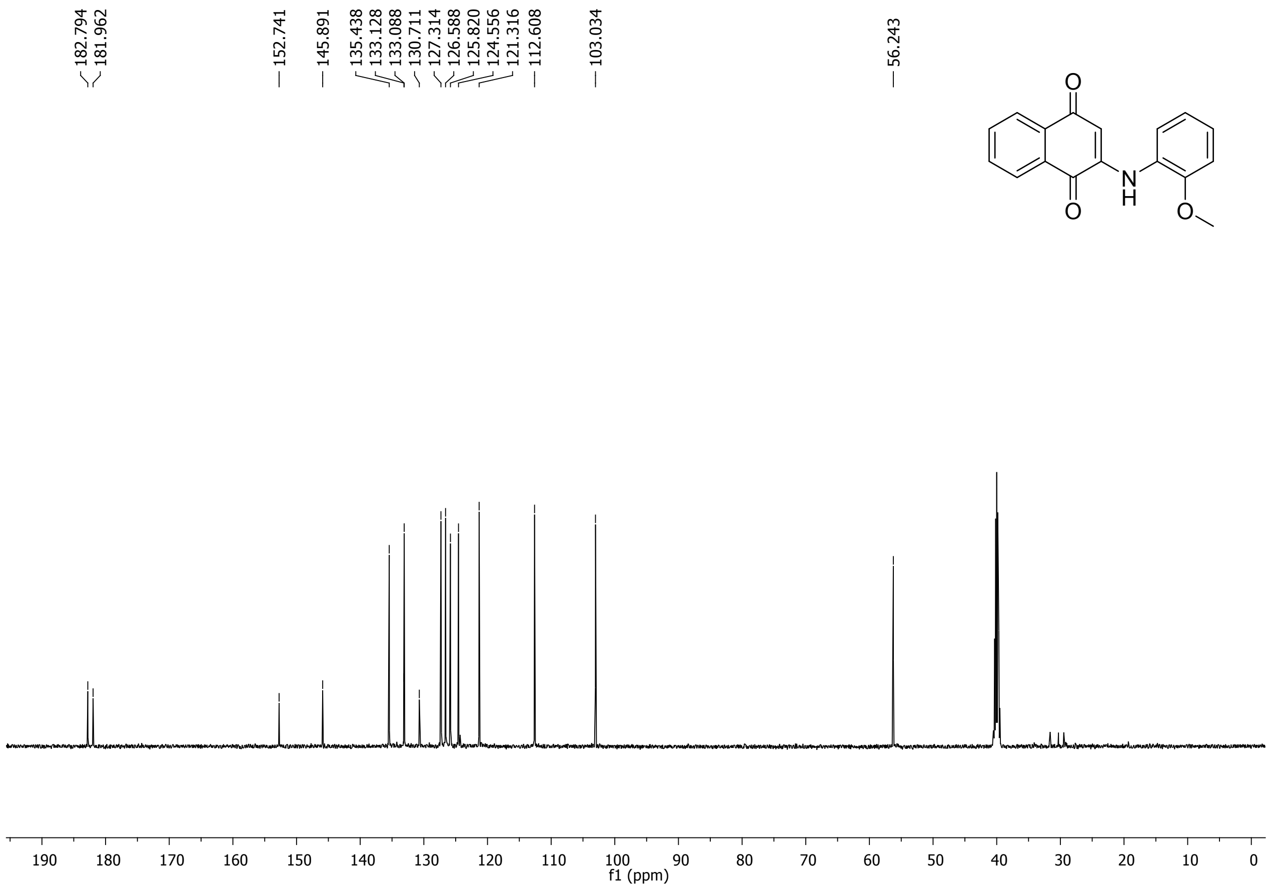

Figure S19. Full $^{13}C\{^1H\}$ NMR DMSO-d_6 spectrum of **3e**

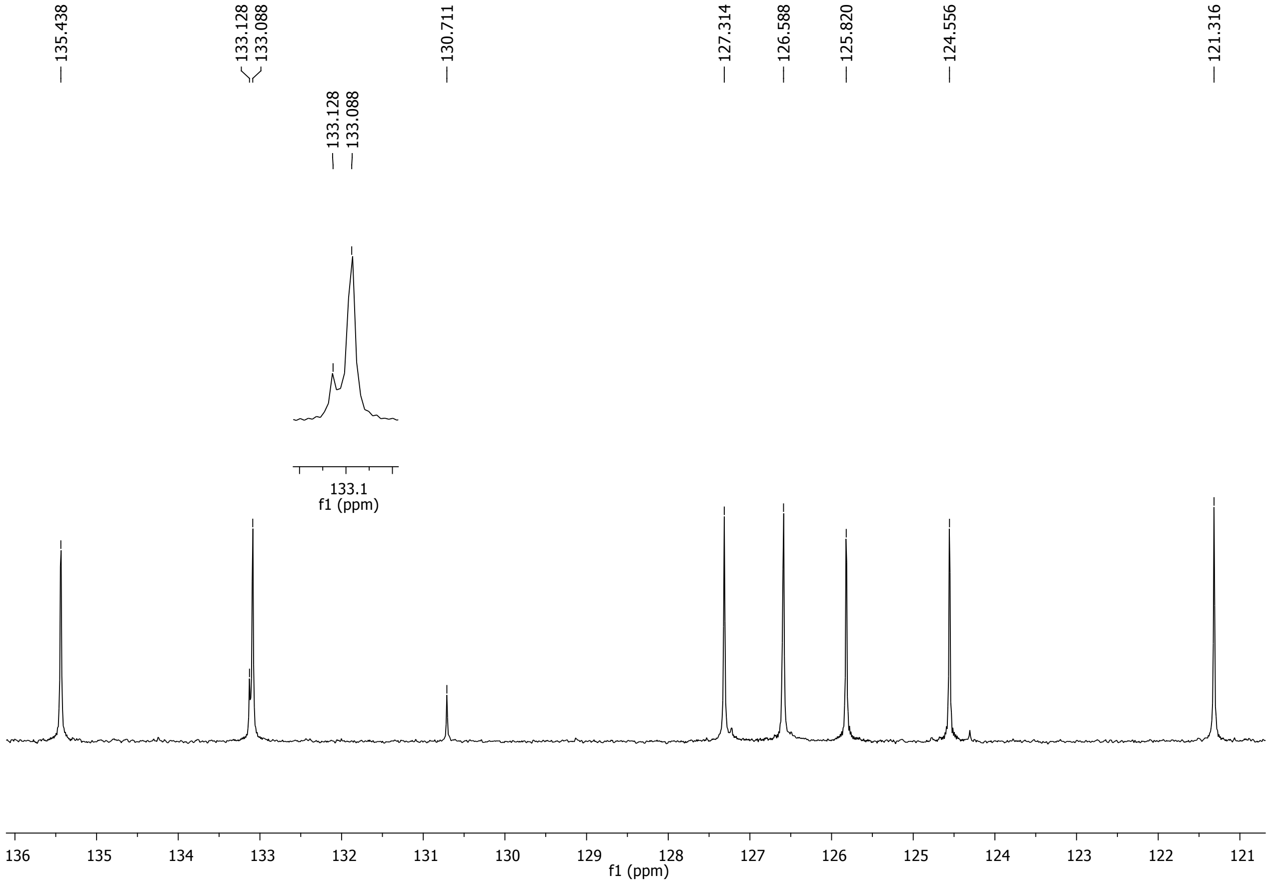

Figure S20. Expanded $^{13}C\{^1H\}$ NMR DMSO-d_6 spectrum of **3e**

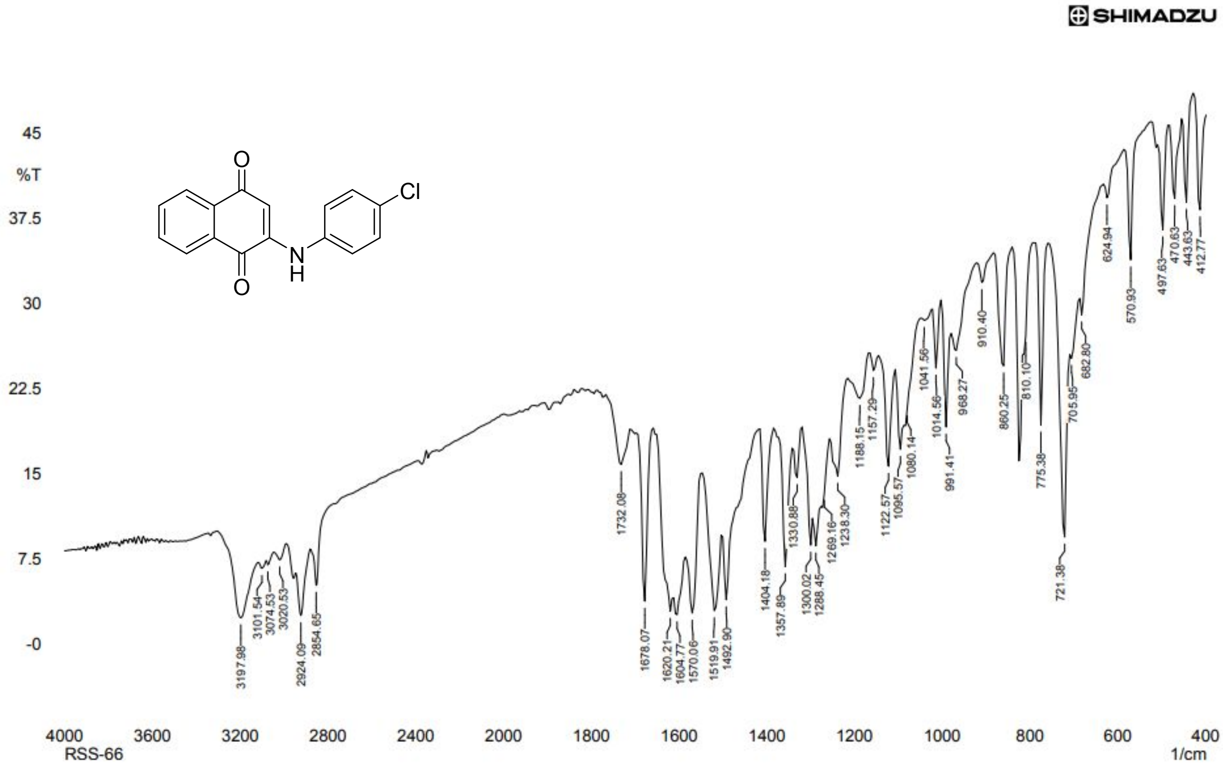

Figure S21. IR (KBr) spectrum of **3f**

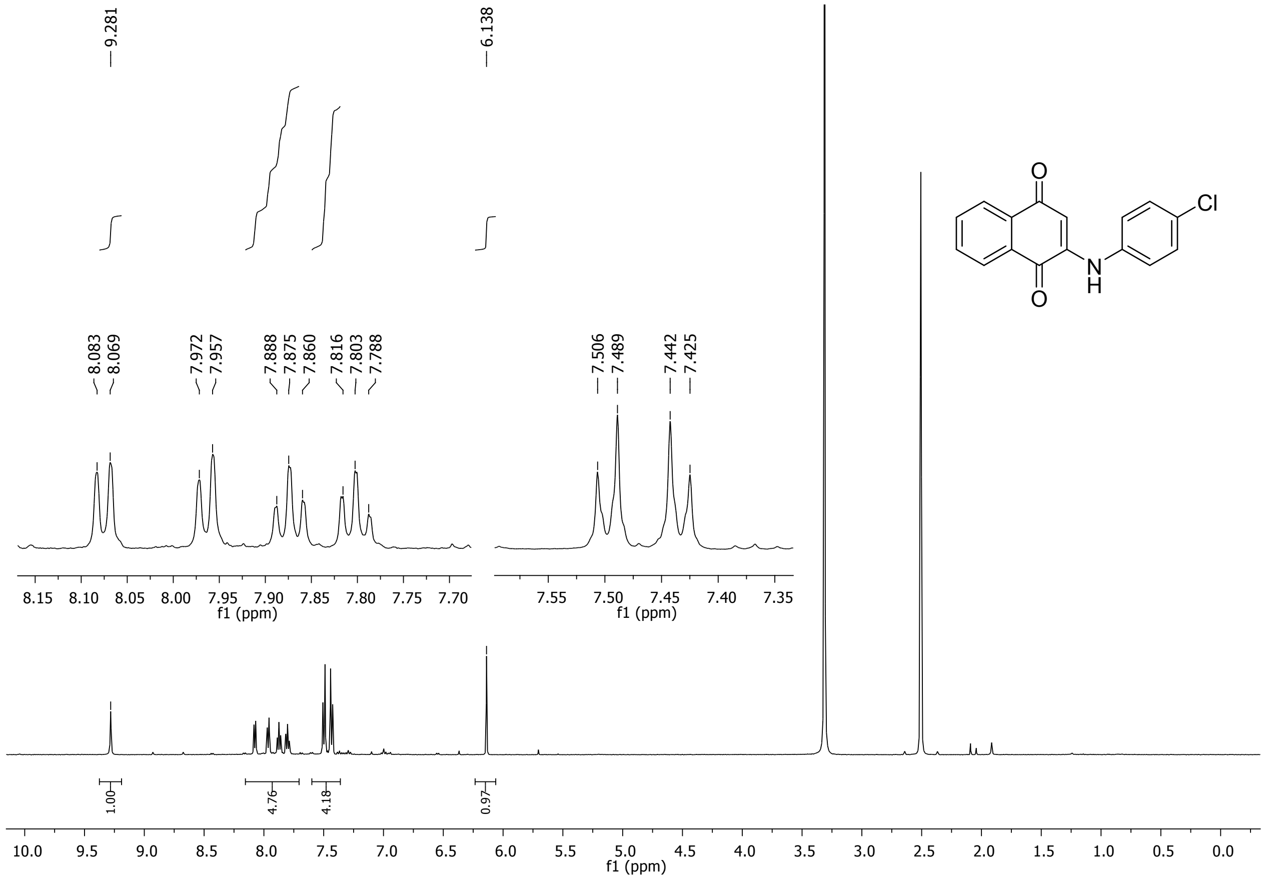

Figure S22. Full ^{1}H NMR DMSO-d_6 spectrum of **3f**

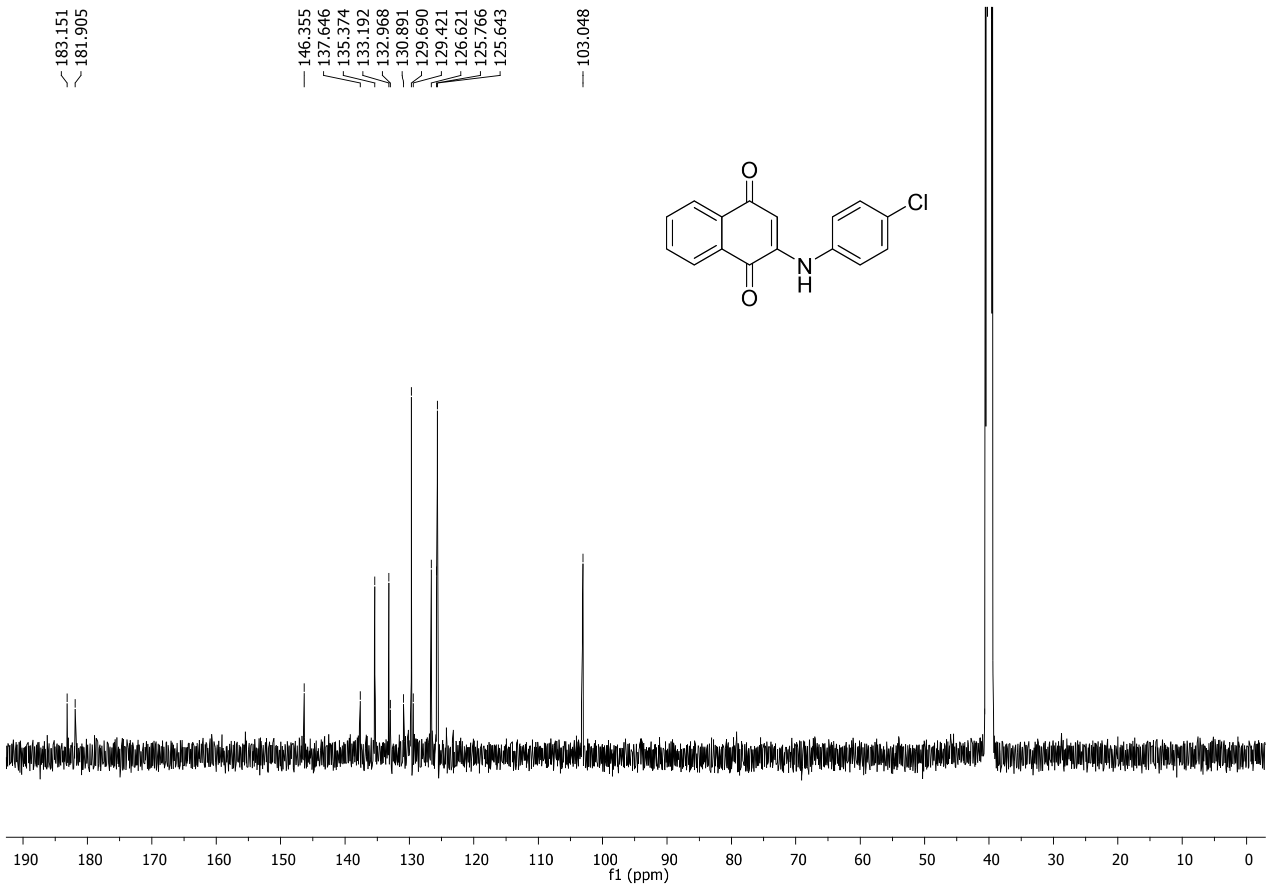

Figure S23. Full $^{13}C\{^{1}H\}$ NMR DMSO-d_6 spectrum of **3f**

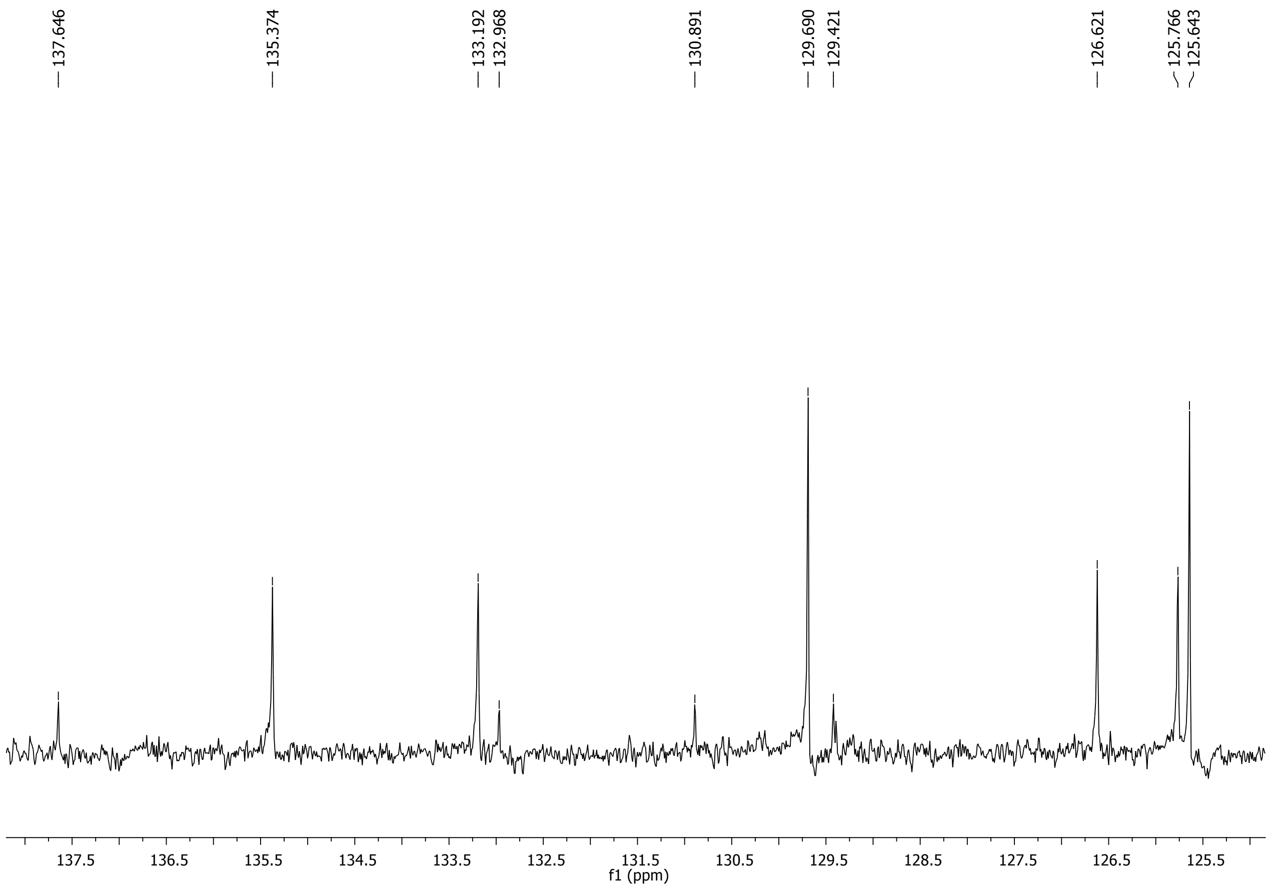

Figure S24. Expanded $^{13}C\{^{1}H\}$ NMR DMSO-d_6 spectrum of **3f**

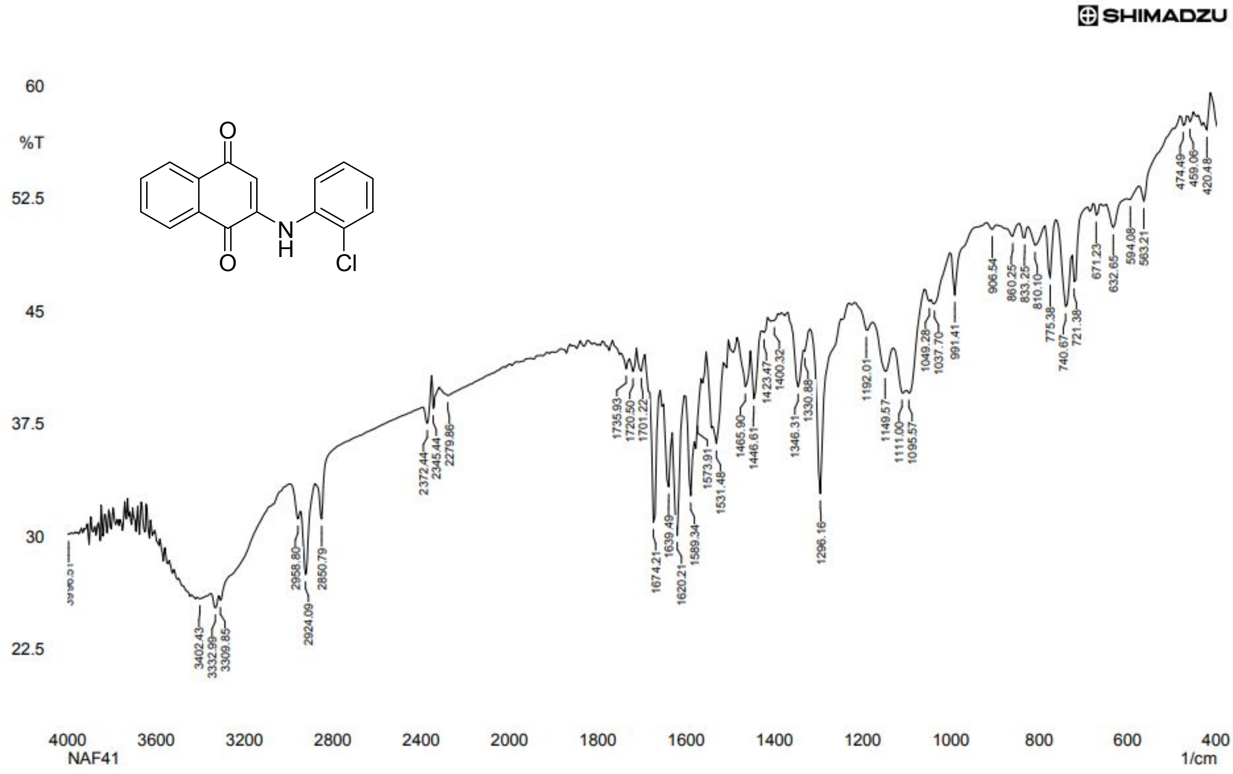

Figure S25. IR (KBr) spectrum of **3g**

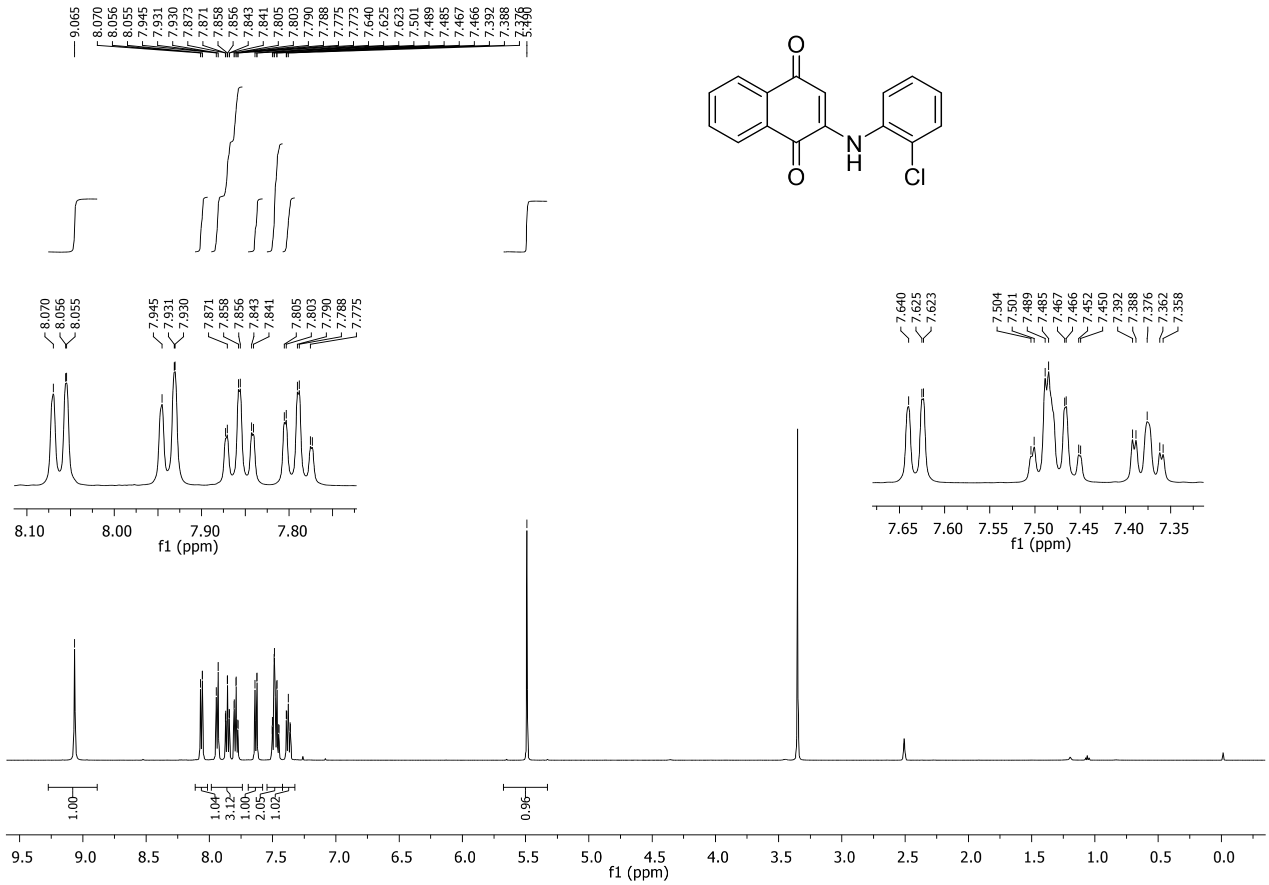

Figure S26. Full ^{1}H NMR DMSO-d_6 spectrum of **3g**

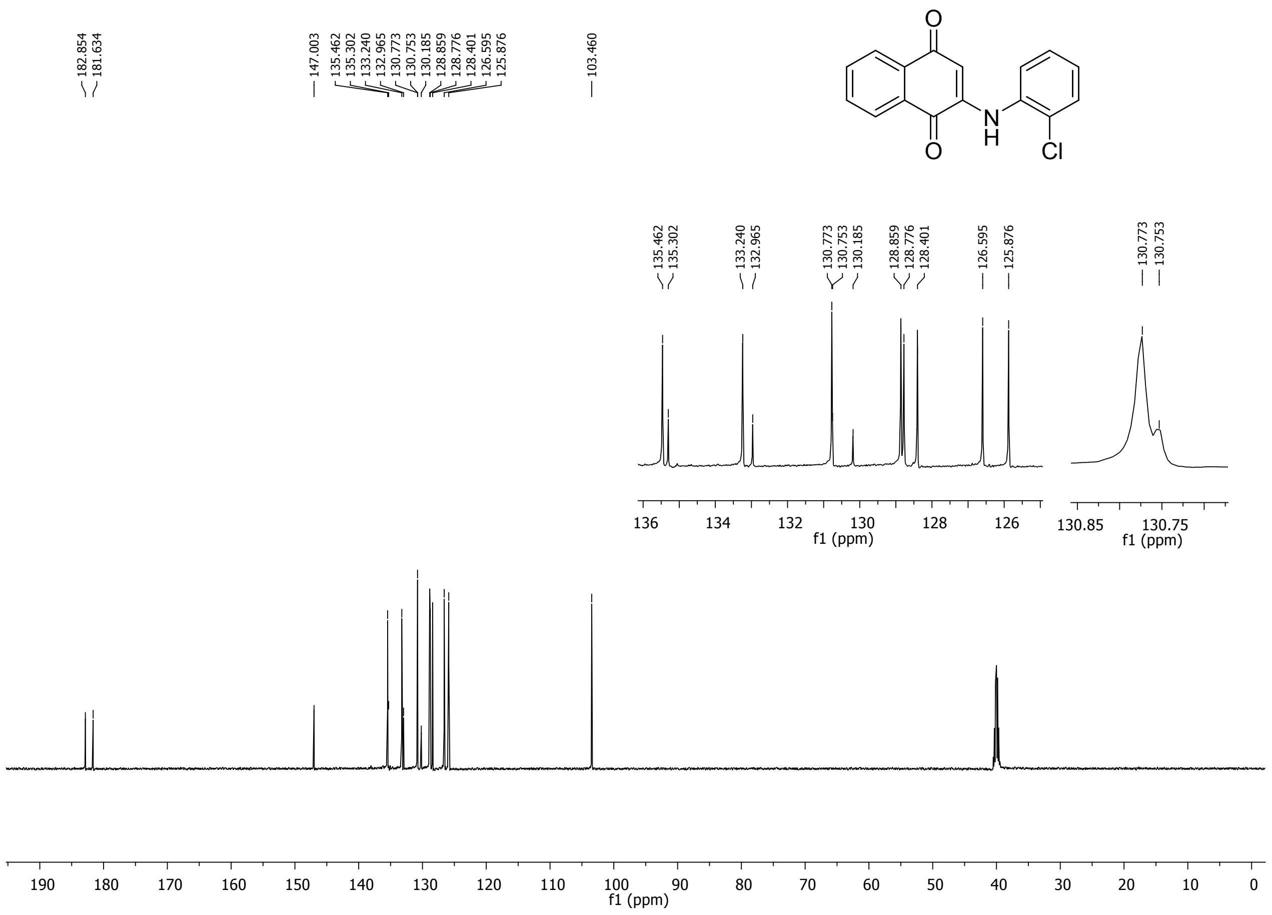

Figure S27. Full $^{13}C\{^1H\}$ NMR DMSO-d_6 spectrum of **3g**

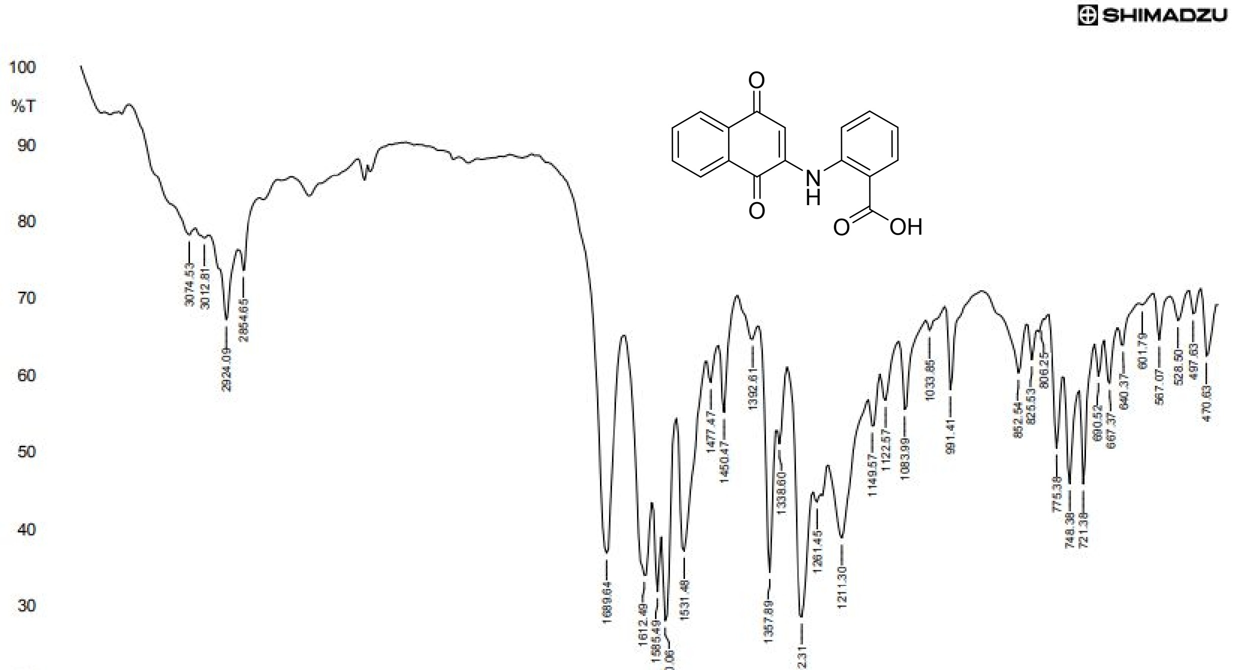

Figure S28. IR (KBr) spectrum of **3h**

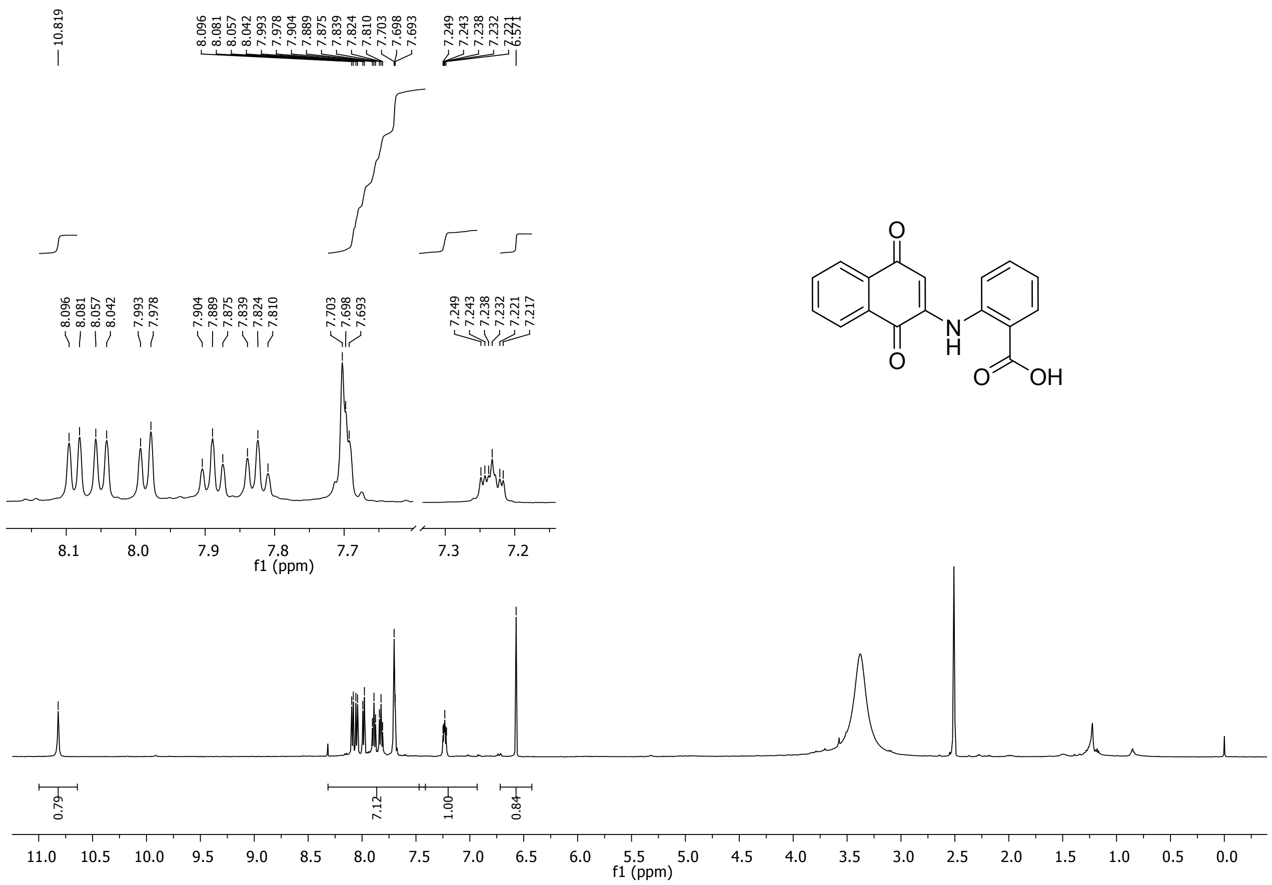

Figure S29. Full ^{1}H NMR DMSO-d_6 spectrum of **3h**

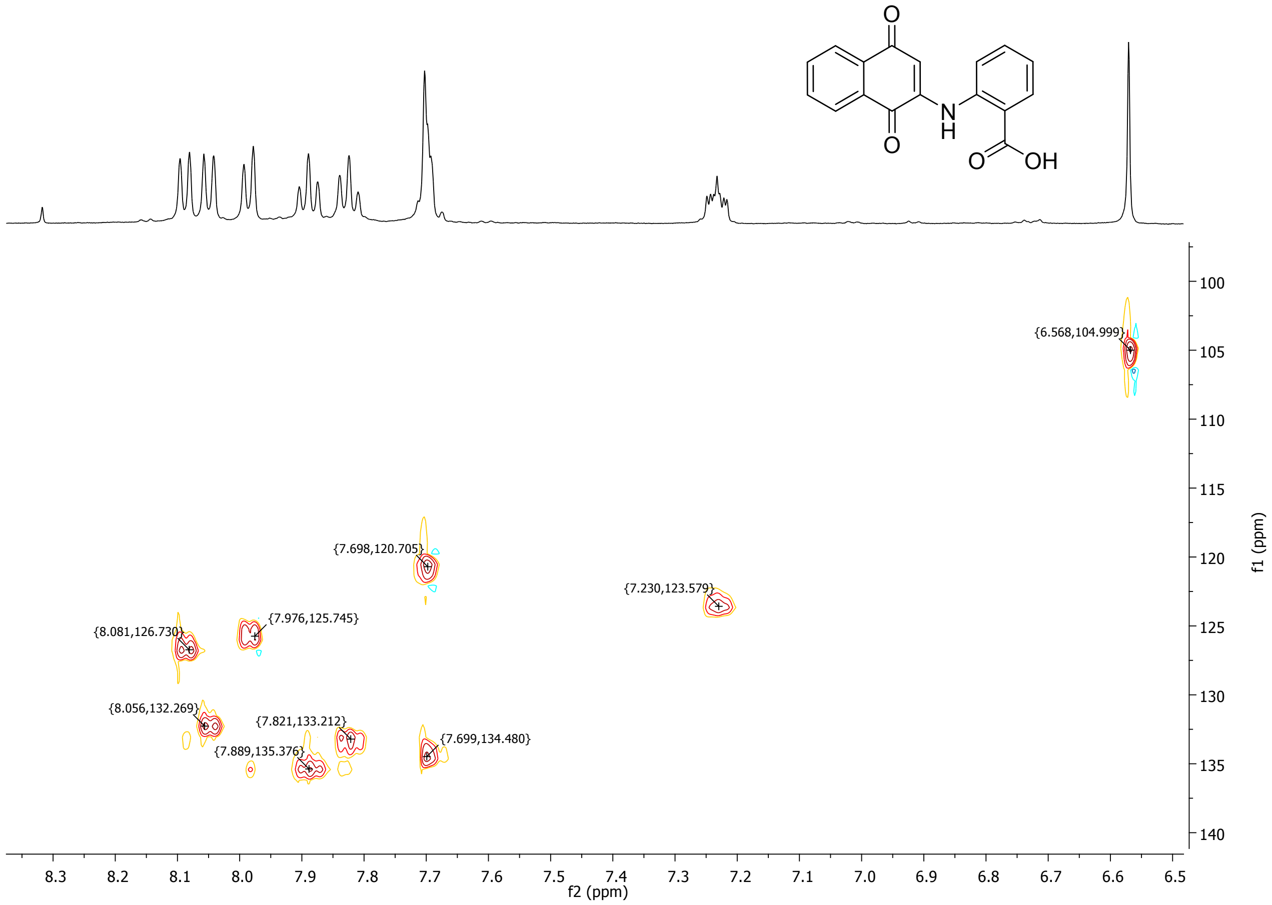

Figure S30. Expanded HSQC spectrum of **3h**

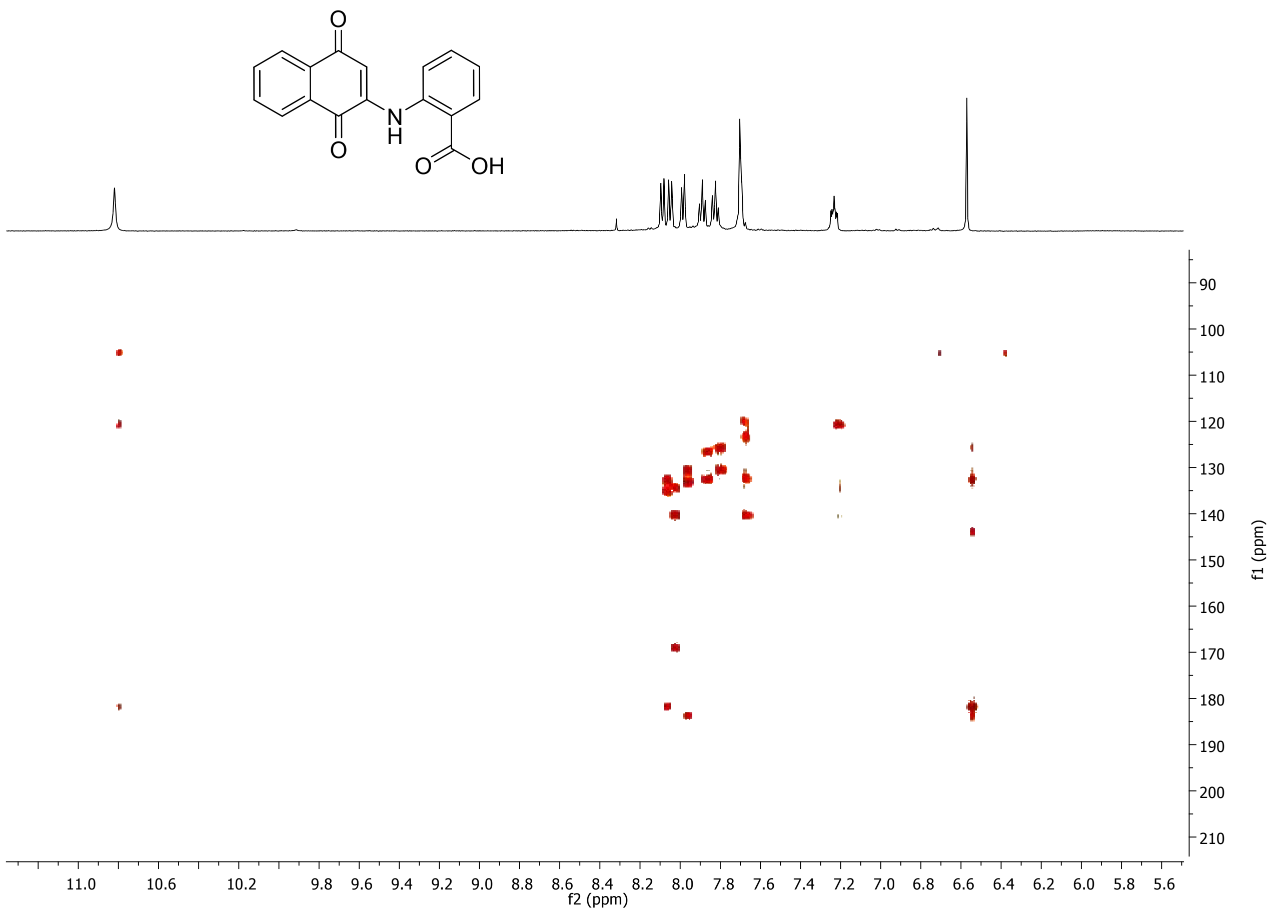

Figure S31. Expanded HMBC spectrum of **3h**

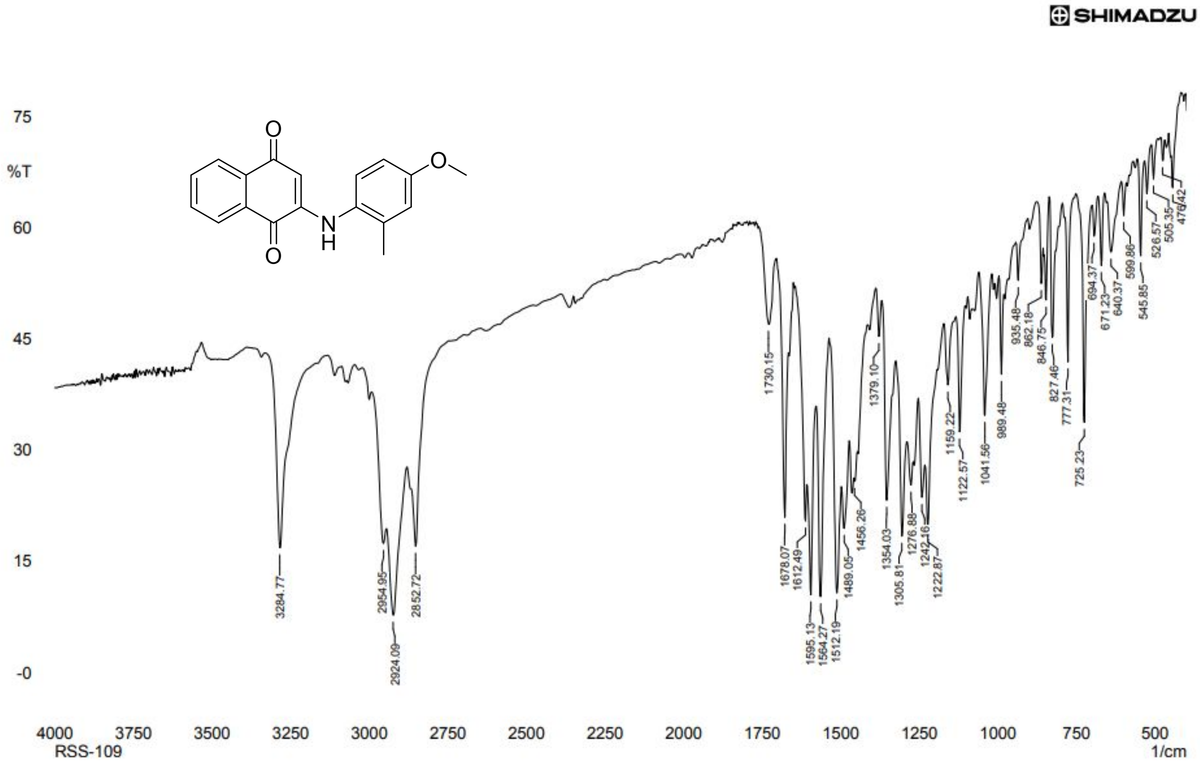

Figure S32. IR (KBr) spectrum of **3i**

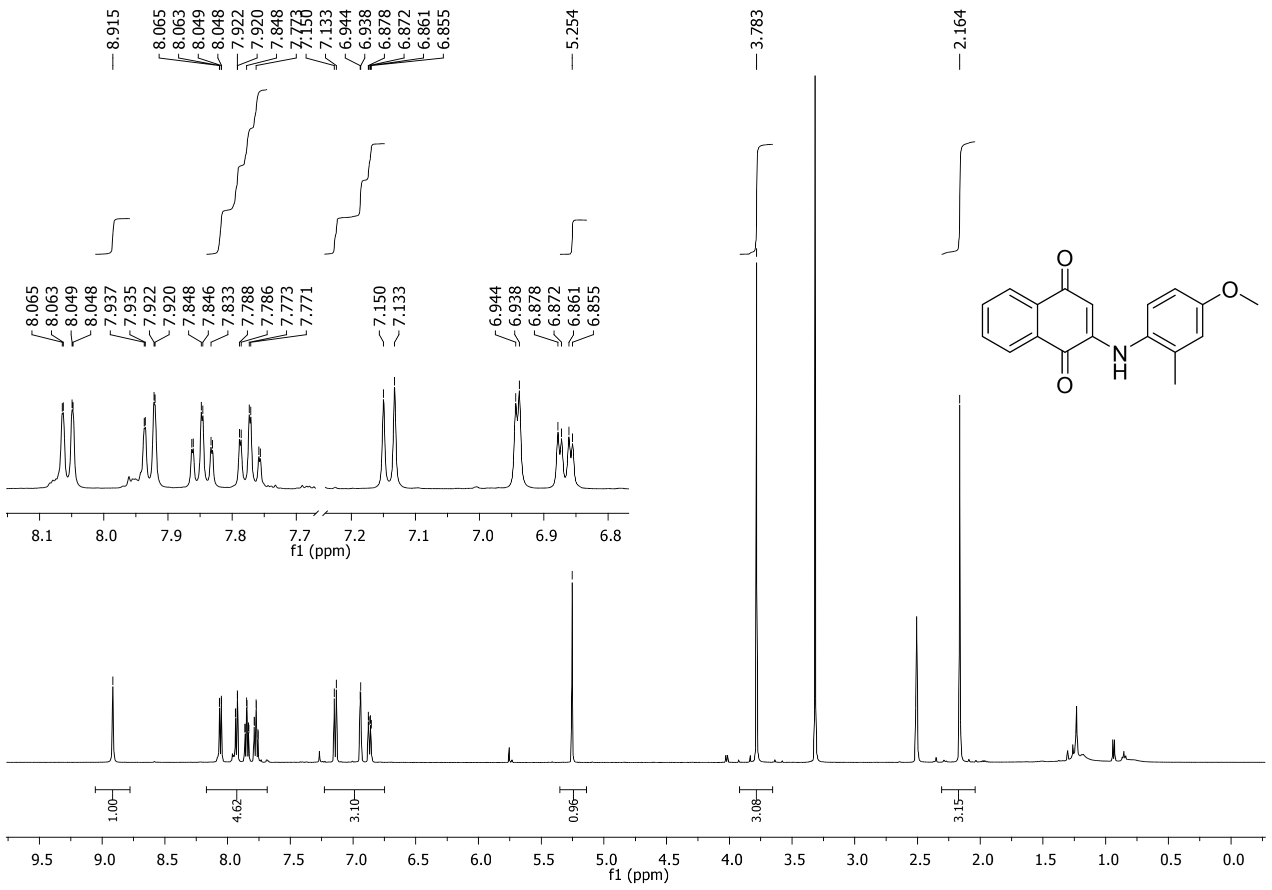

Figure S33. Full ^{1}H NMR DMSO-d_6 spectrum of **3i**

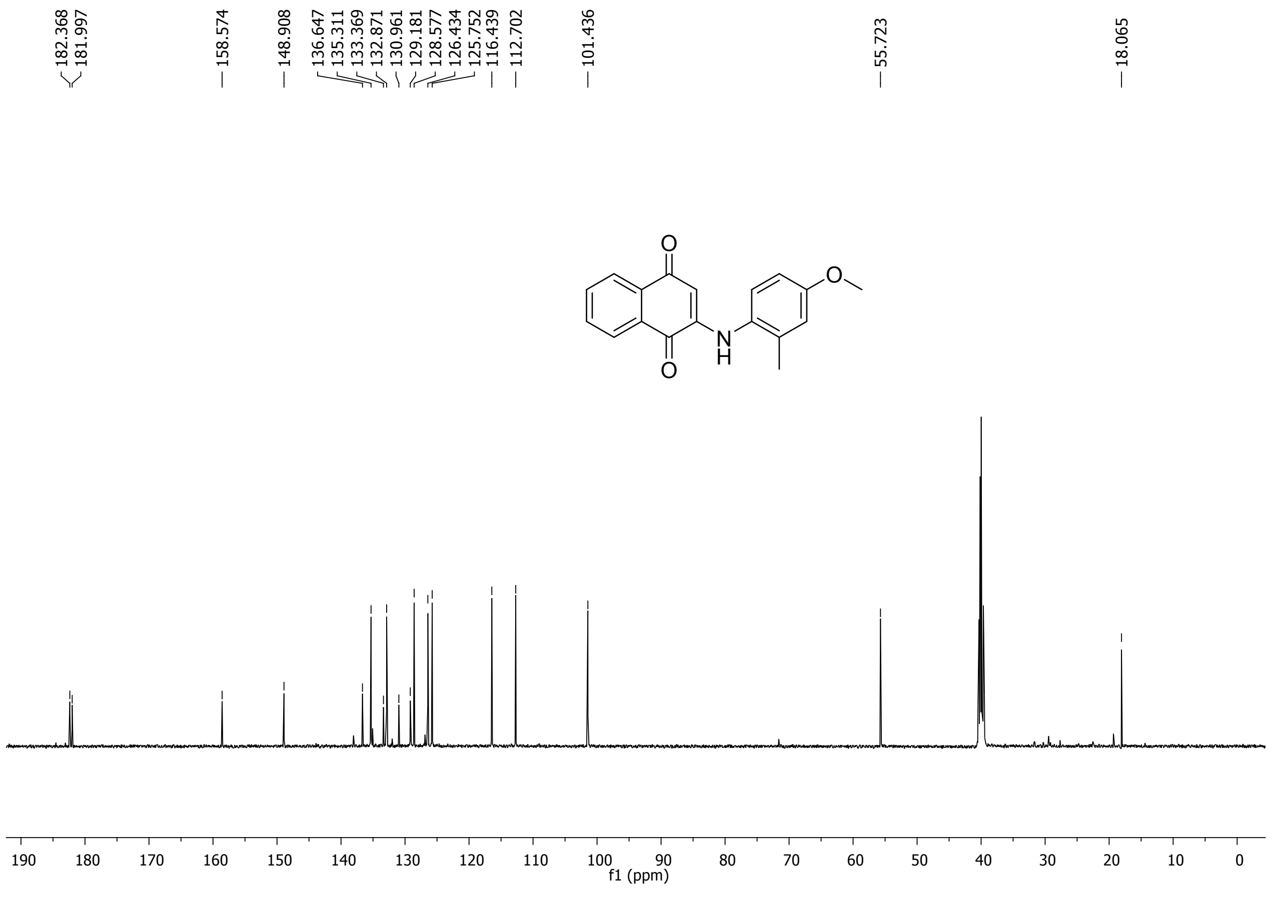

Figure S34. Full $^{13}C\{^{1}H\}$ NMR DMSO-d_6 spectrum of **3i**

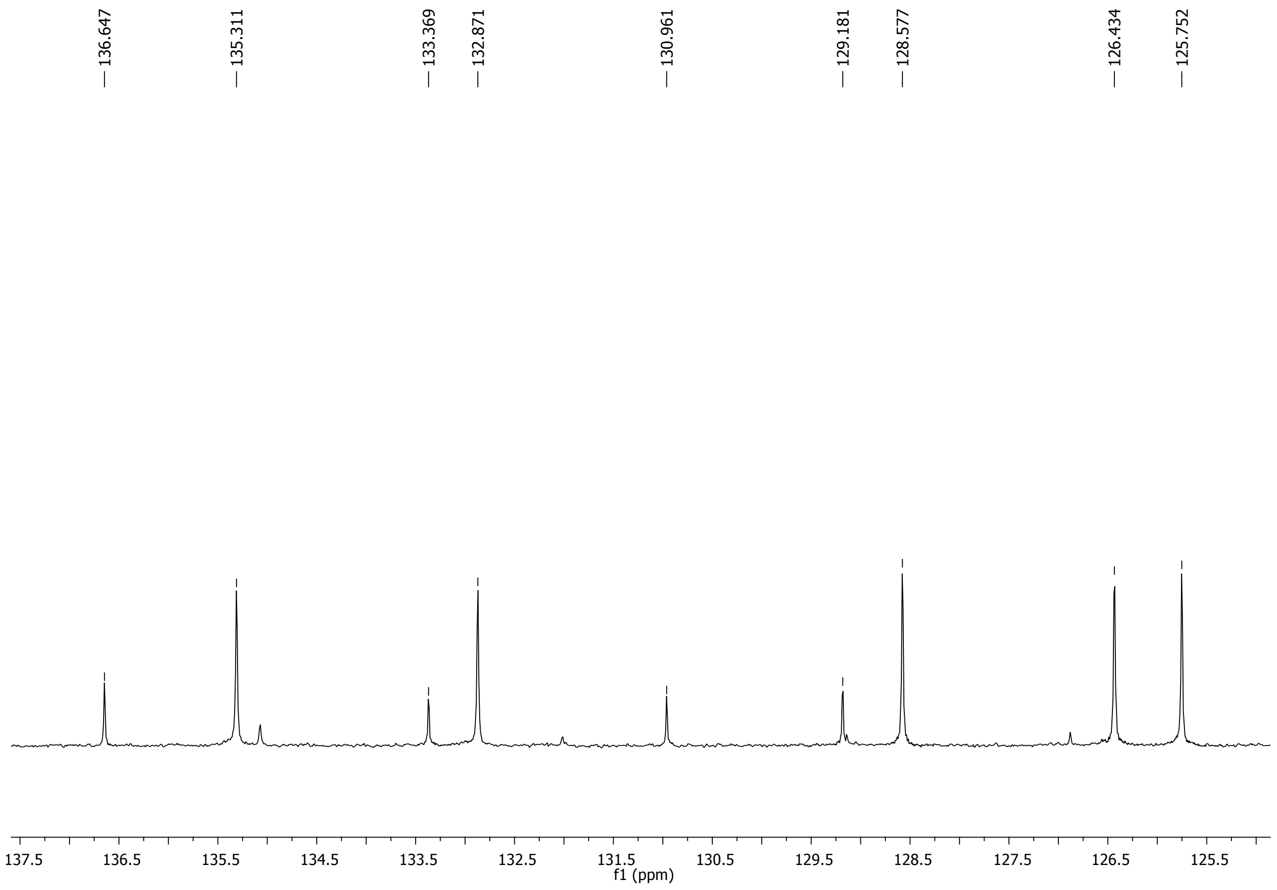

Figure S35. Expanded $^{13}C\{^{1}H\}$ NMR DMSO-d_6 spectrum of **3i**

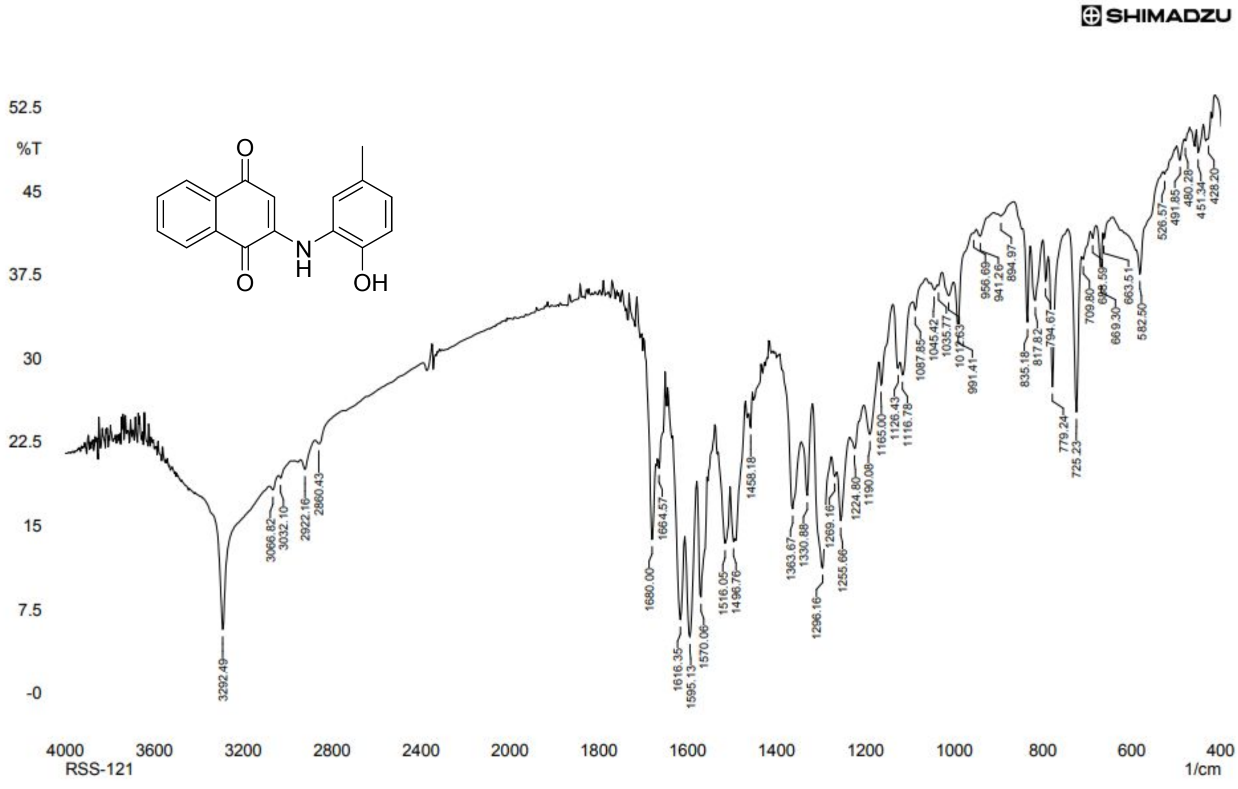

Figure S36. IR (KBr) spectrum of **3j**

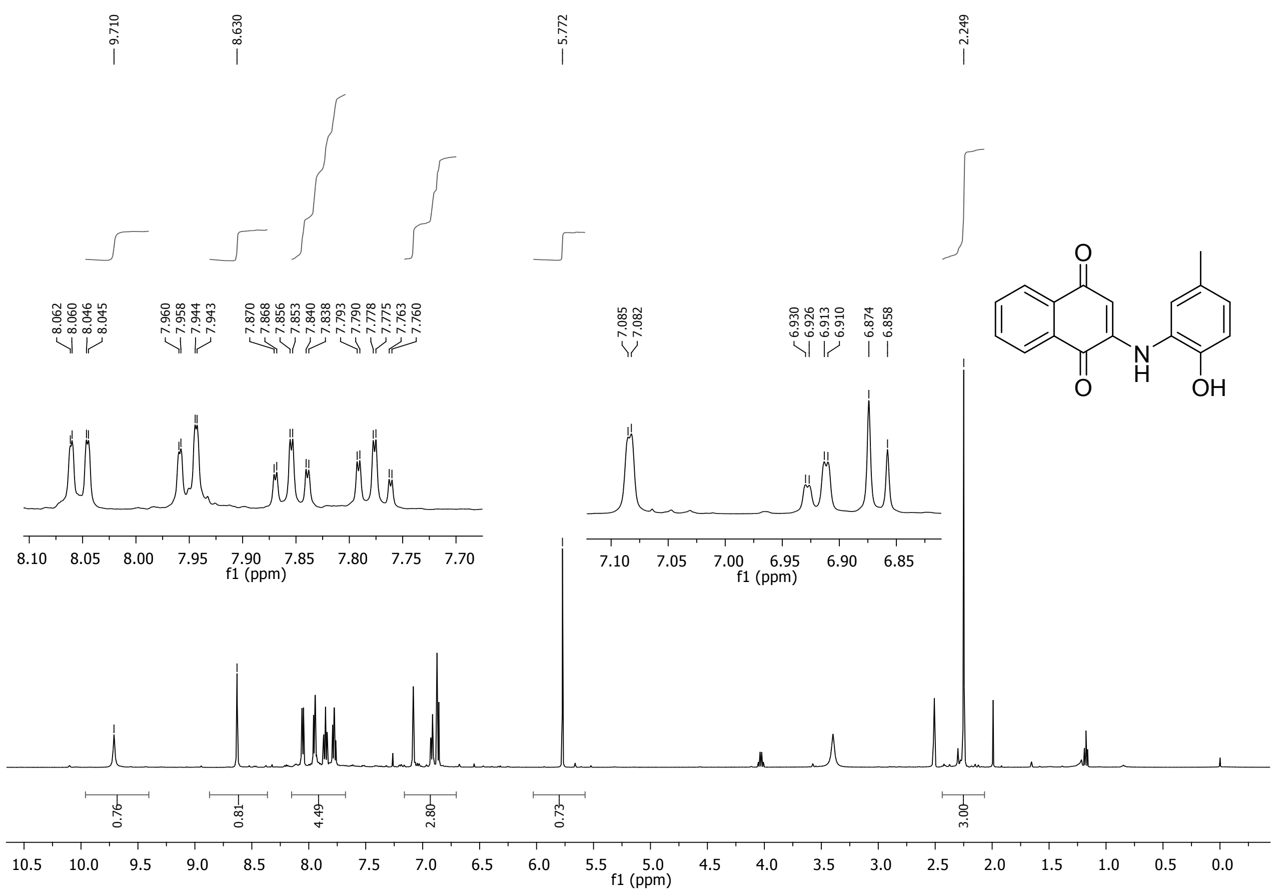

Figure S37. Full ^{1}H NMR DMSO-d$_6$ spectrum of **3j**

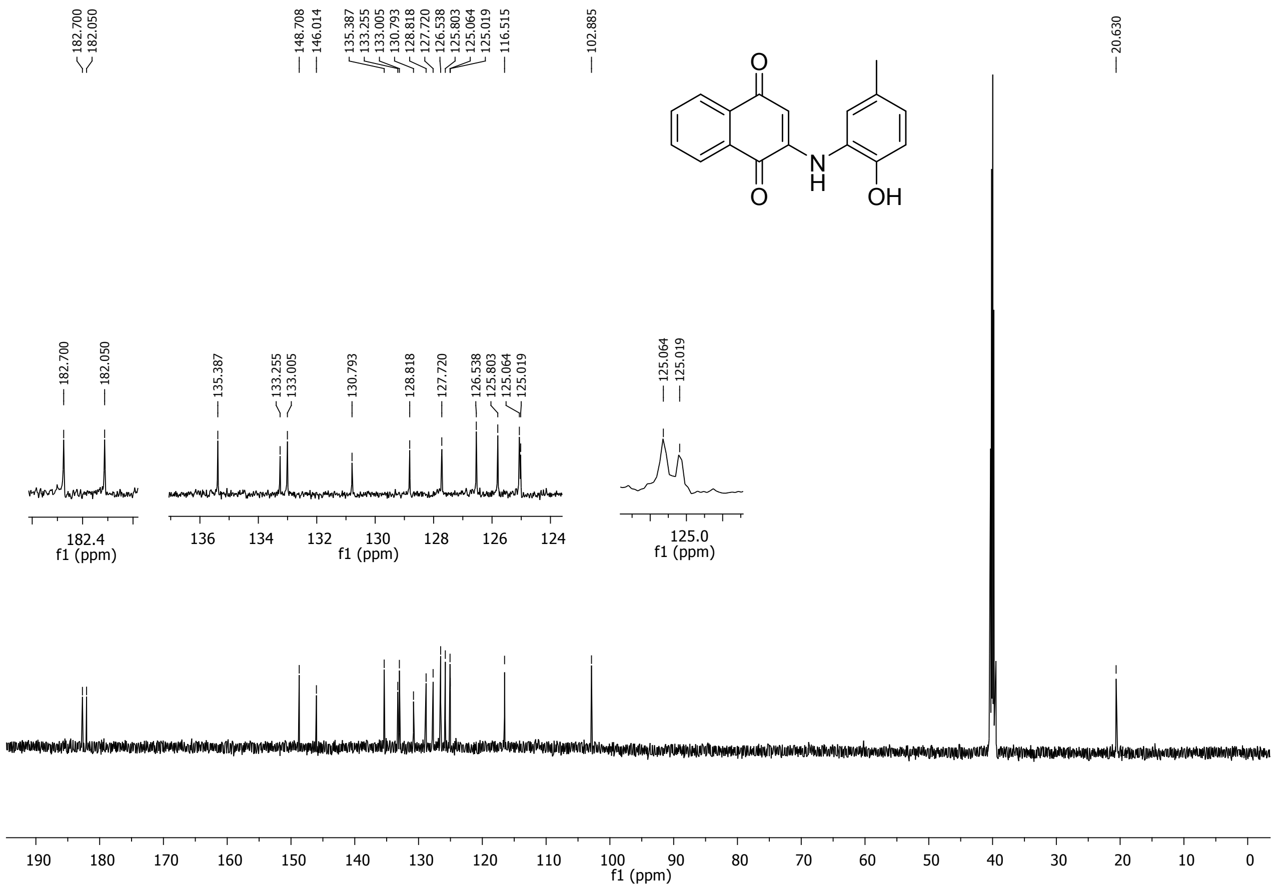

Figure S38. Full ^{13}C{^{1}H} NMR DMSO-d$_6$ spectrum of **3j**

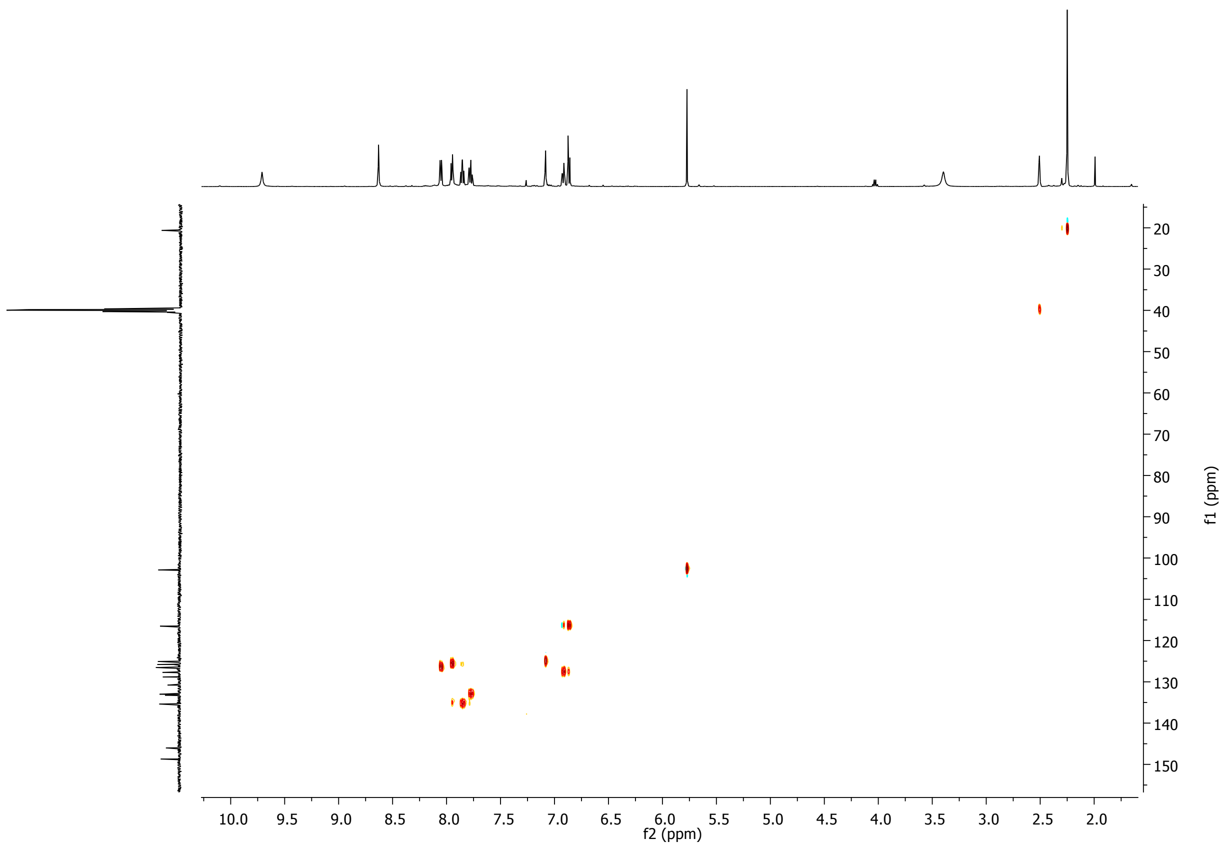

Figure S39. Full HSQC spectrum of **3j**

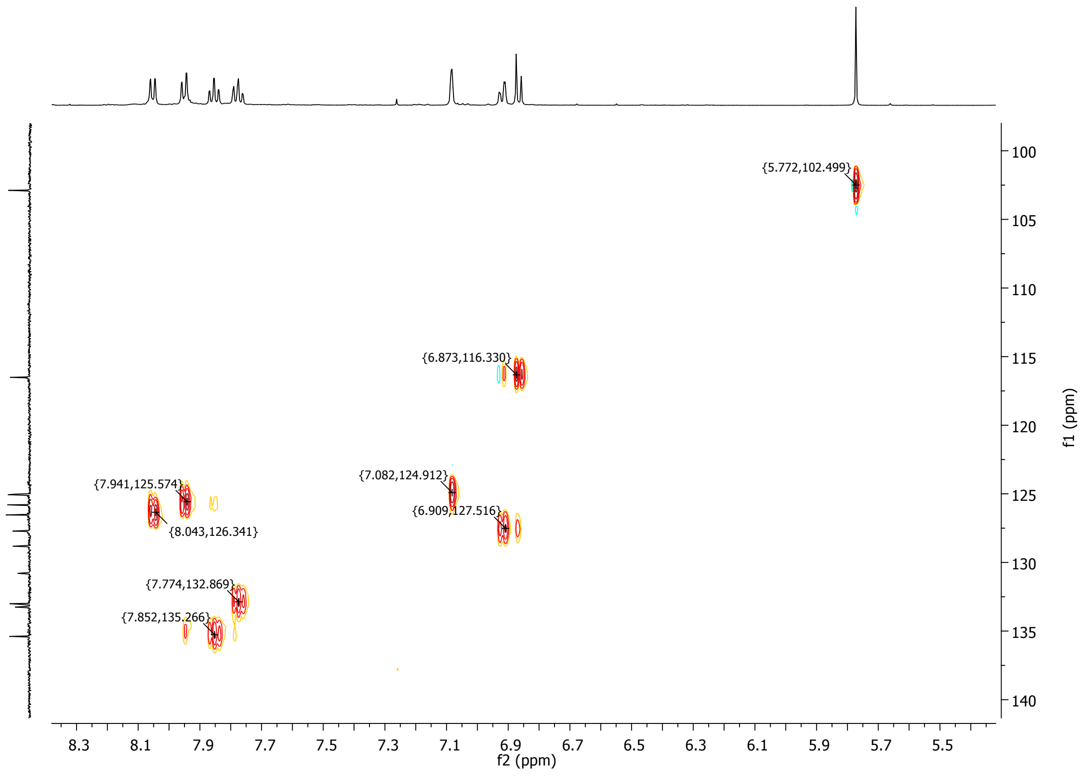

Figure S40. Expanded HSQC spectrum of **3j**

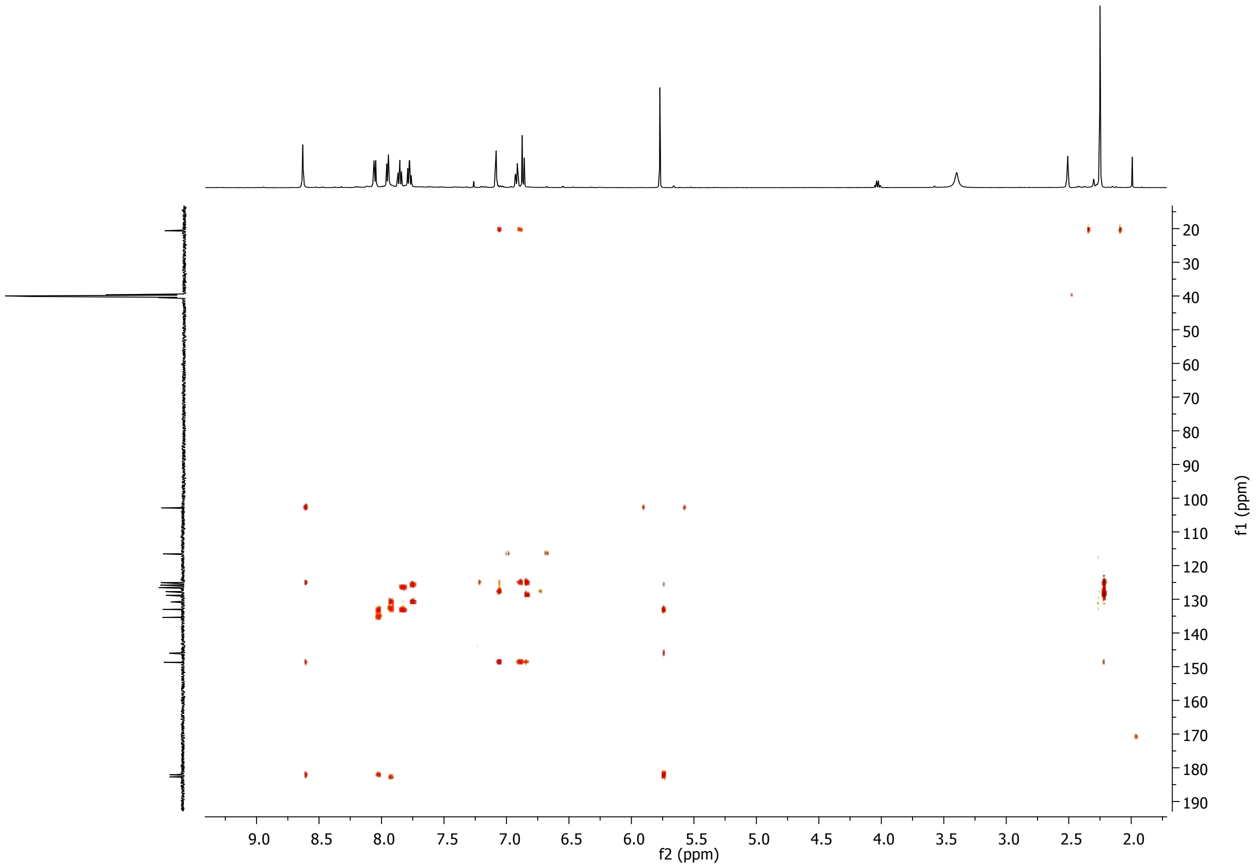

Figure S41. Full HMBC spectrum of **3j**

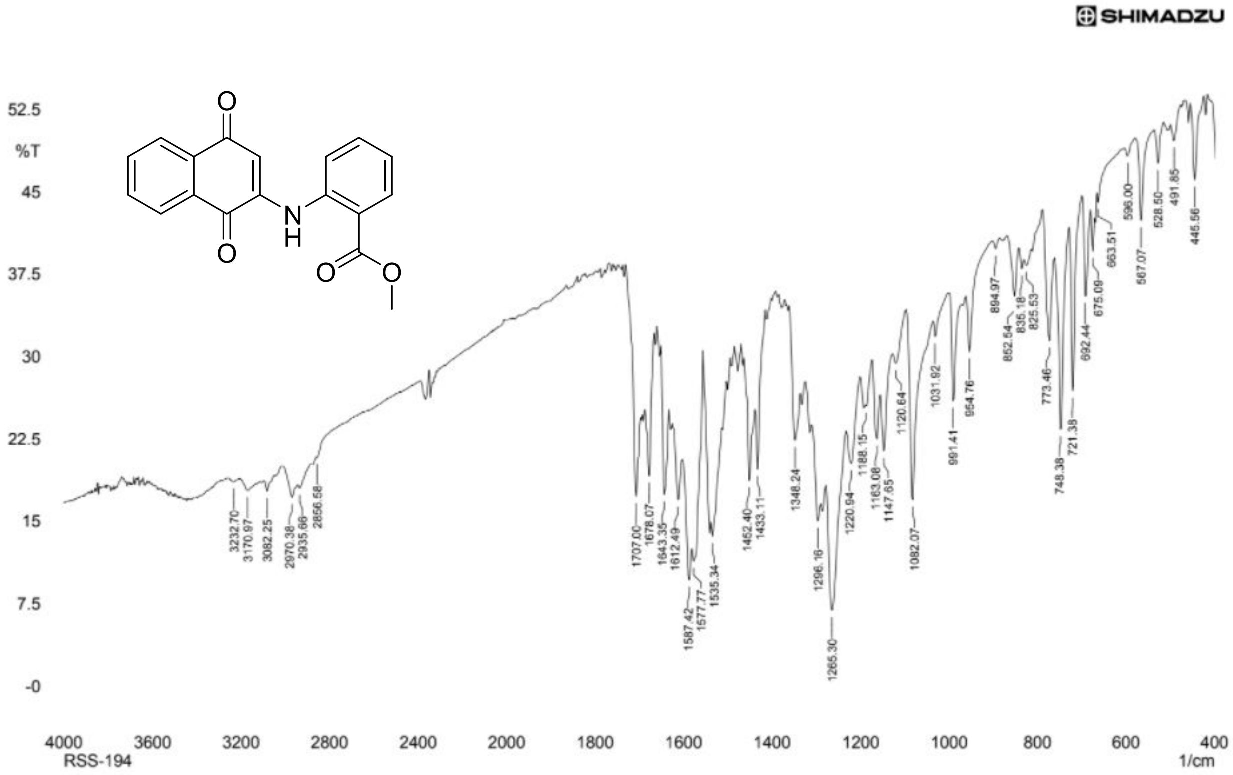

Figure S42. IR (KBr) spectrum of **3k**

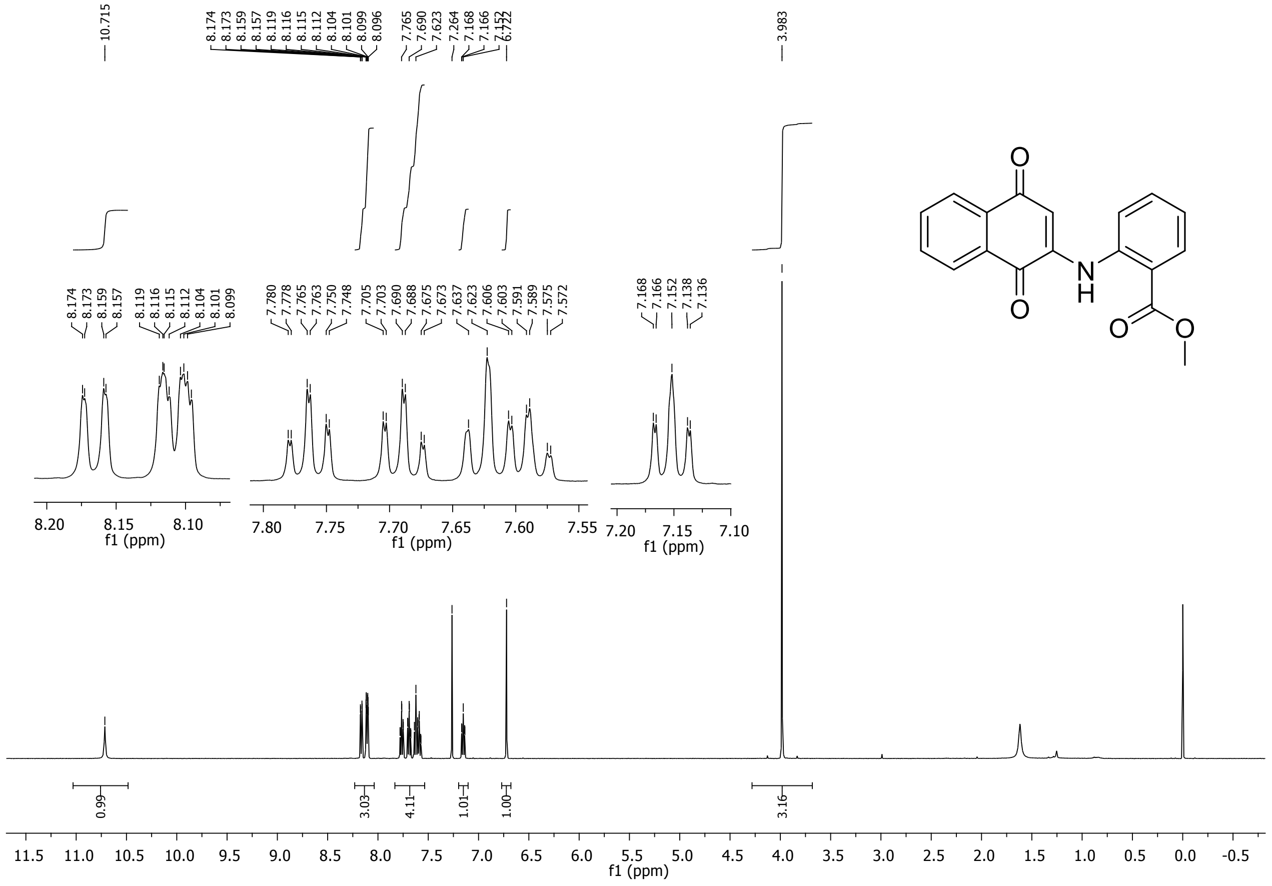

Figure S43. Full ^{1}H NMR $CDCl_3$ spectrum of **3k**

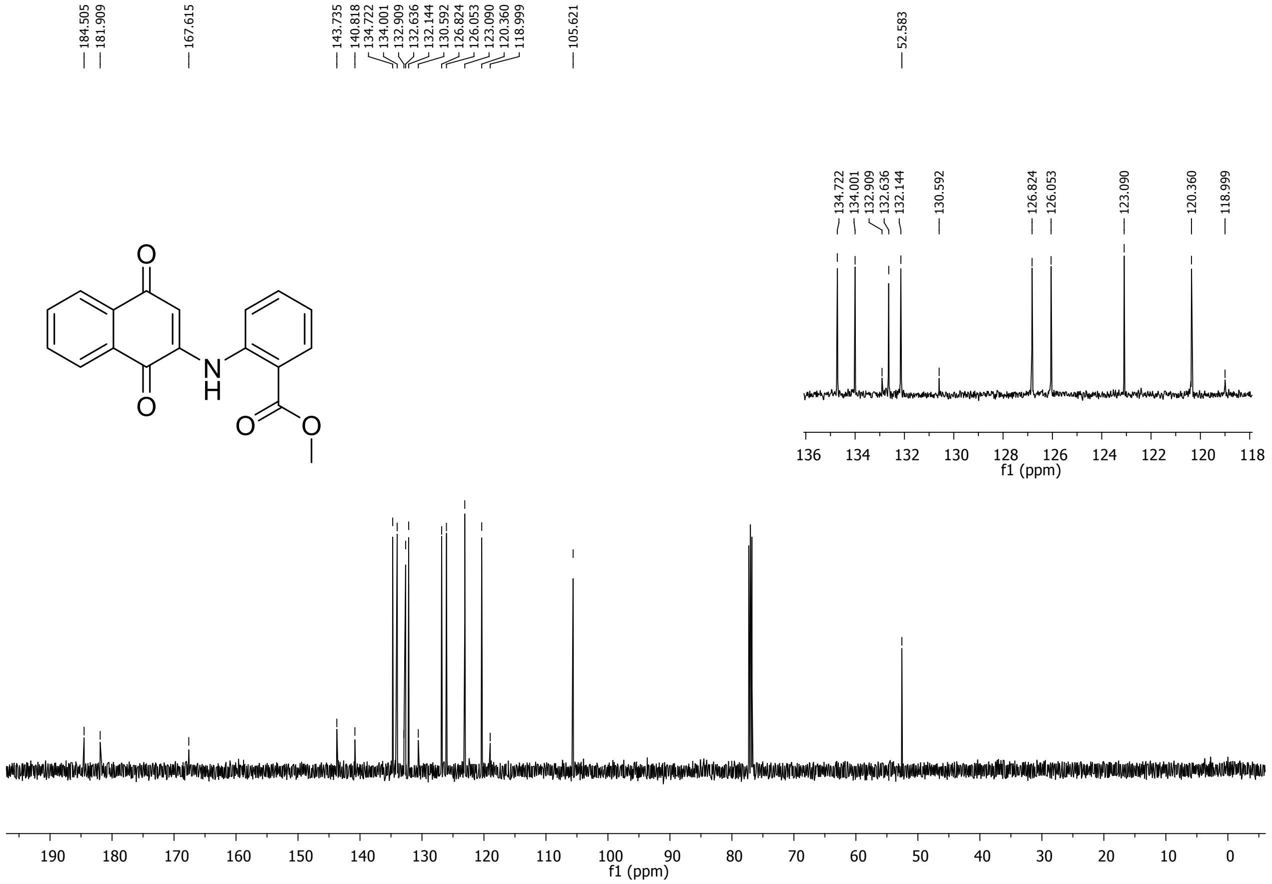

Figure S44. Full $^{13}C\{^{1}H\}$ NMR $CDCl_3$ spectrum of **3k**

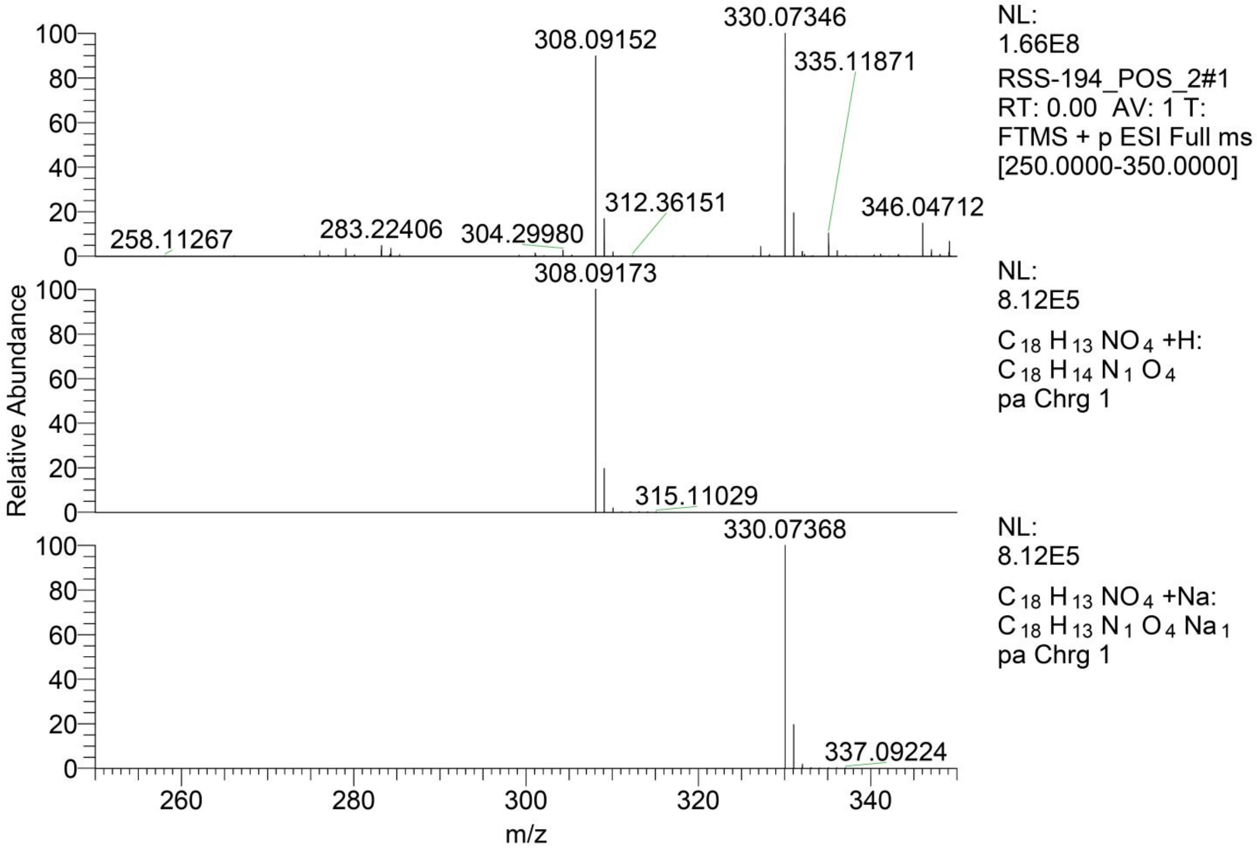

Figure S45. HRMS spectrum of **3k**

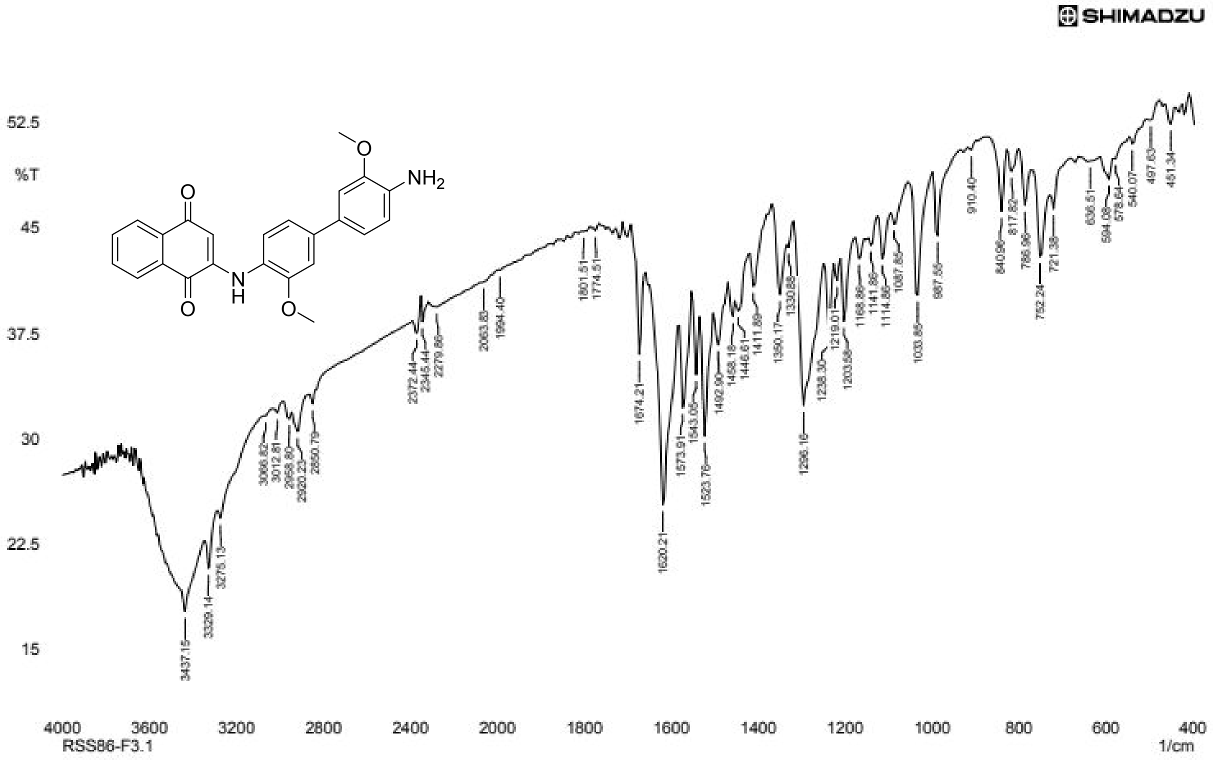

Figure S46. IR (KBr) spectrum of **3l**

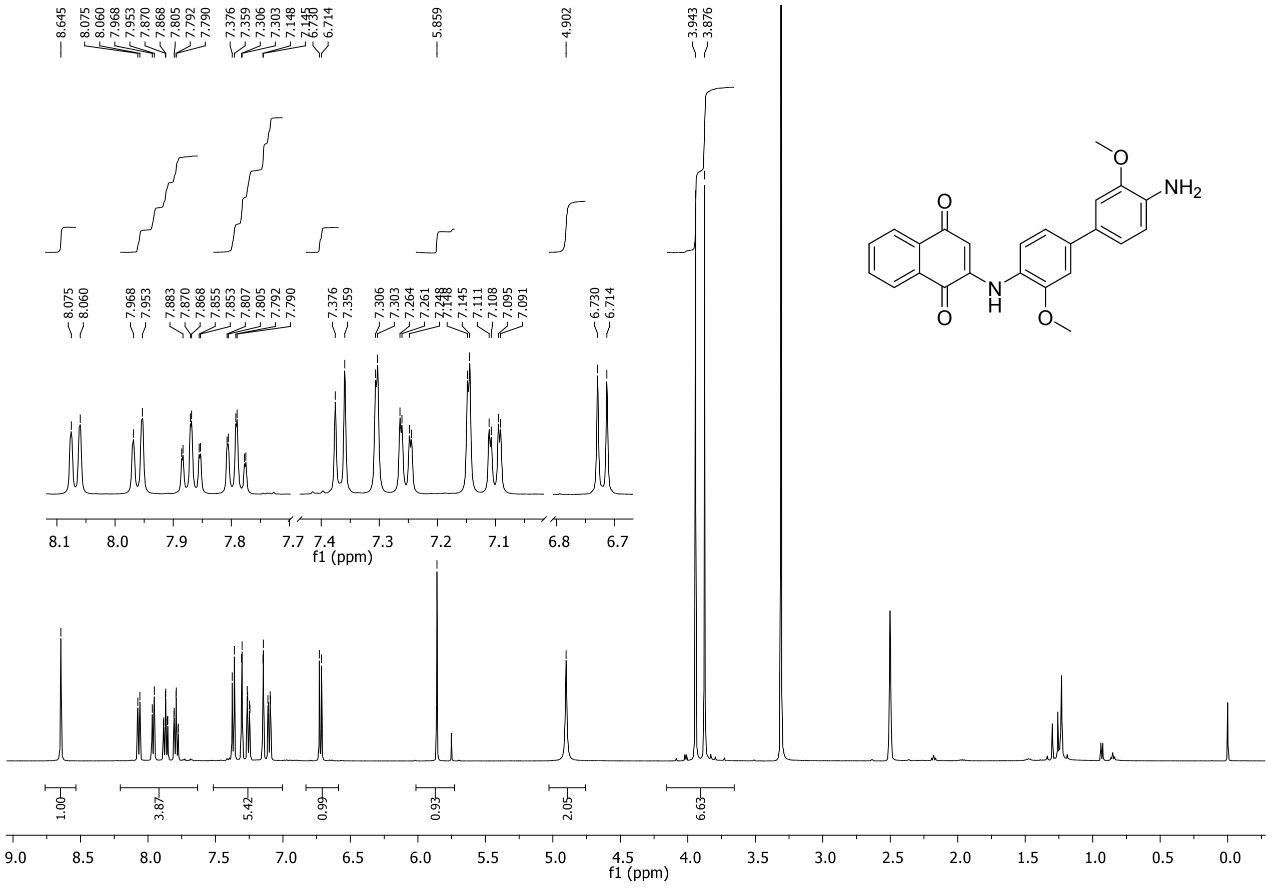

Figure S47. Full ^{1}H NMR DMSO-d_6 spectrum of **3l**

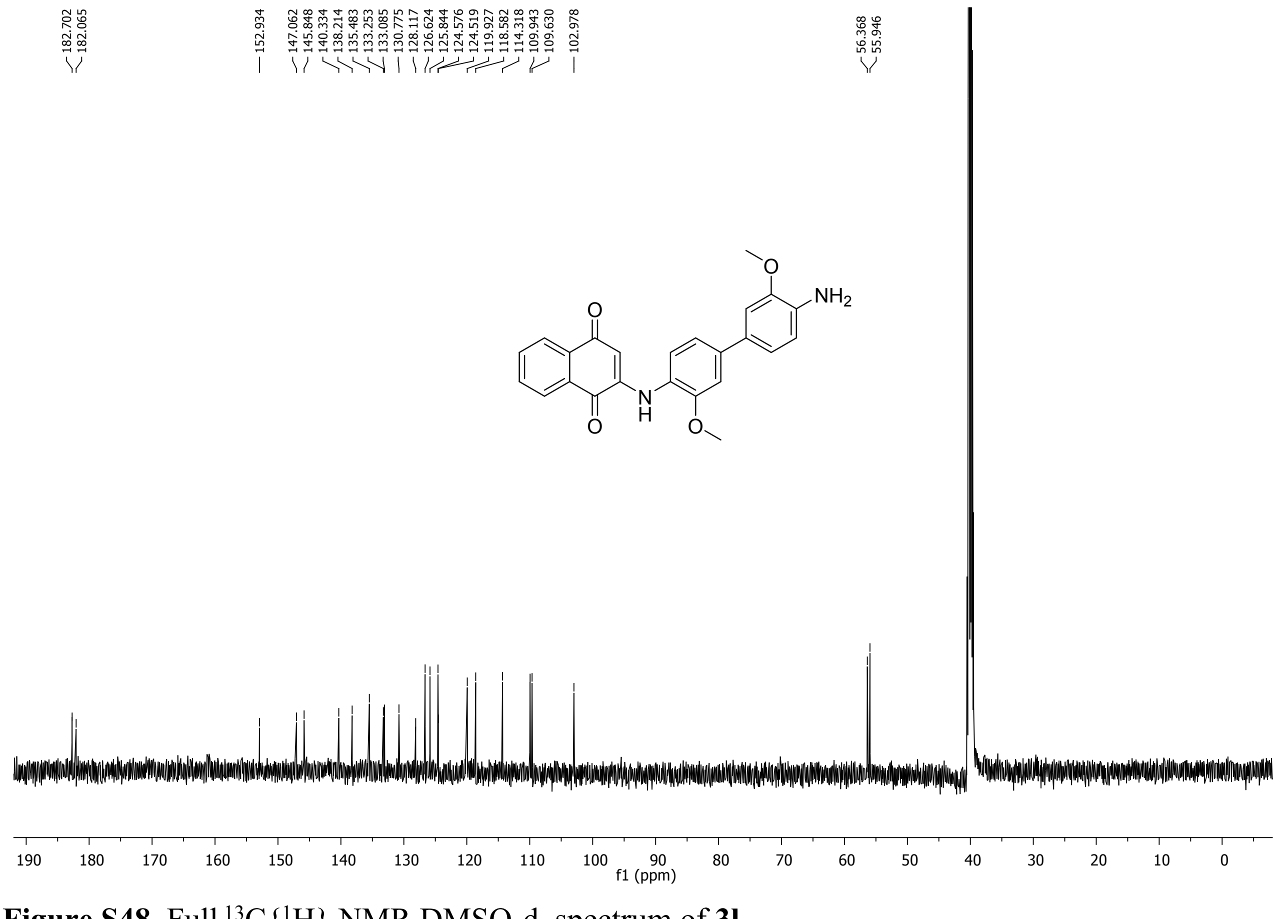

Figure S48. Full $^{13}C\{^{1}H\}$ NMR DMSO-d_6 spectrum of **3l**

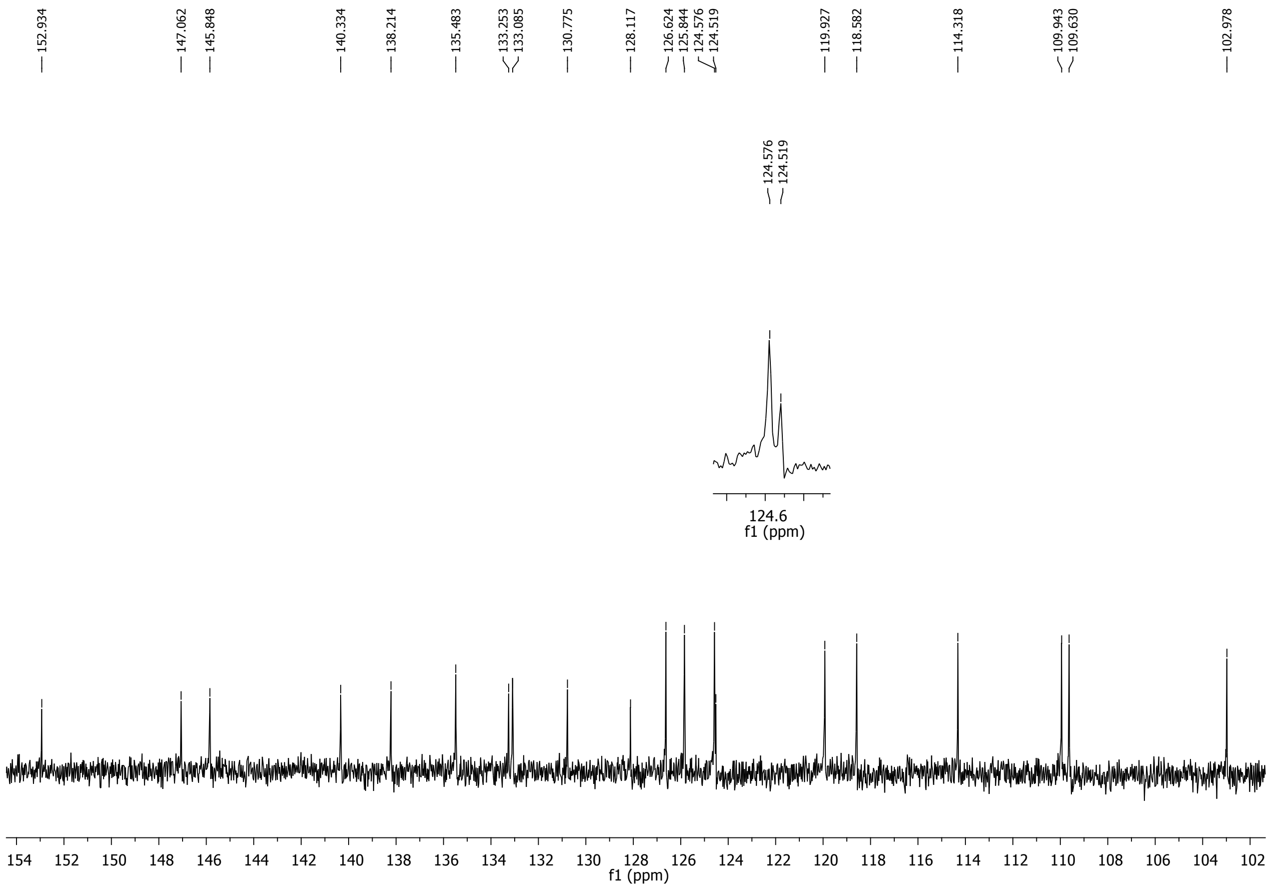

Figure S49. Expanded $^{13}C\{^{1}H\}$ NMR DMSO-d_6 spectrum of **3l**

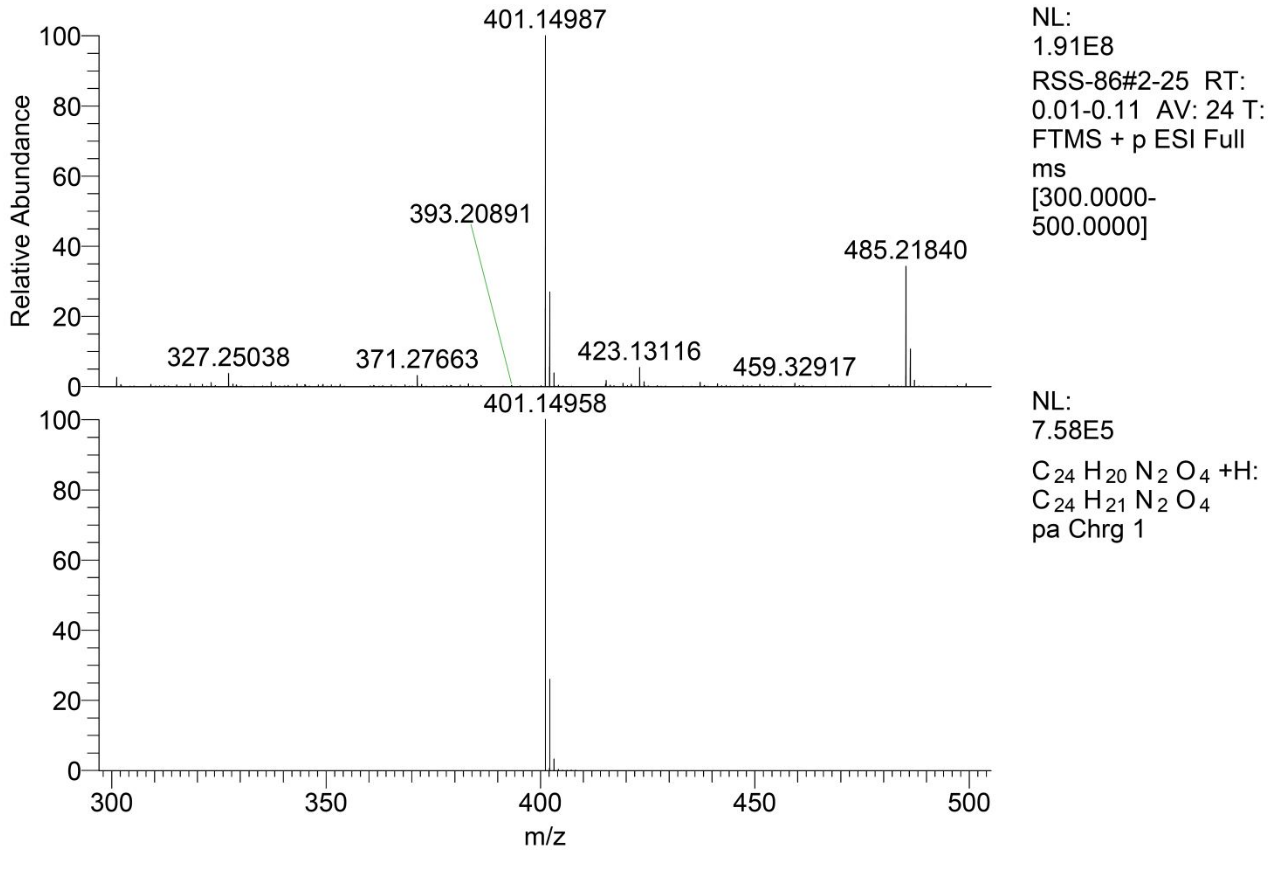

Figure S50. HRMS spectrum of **3l**

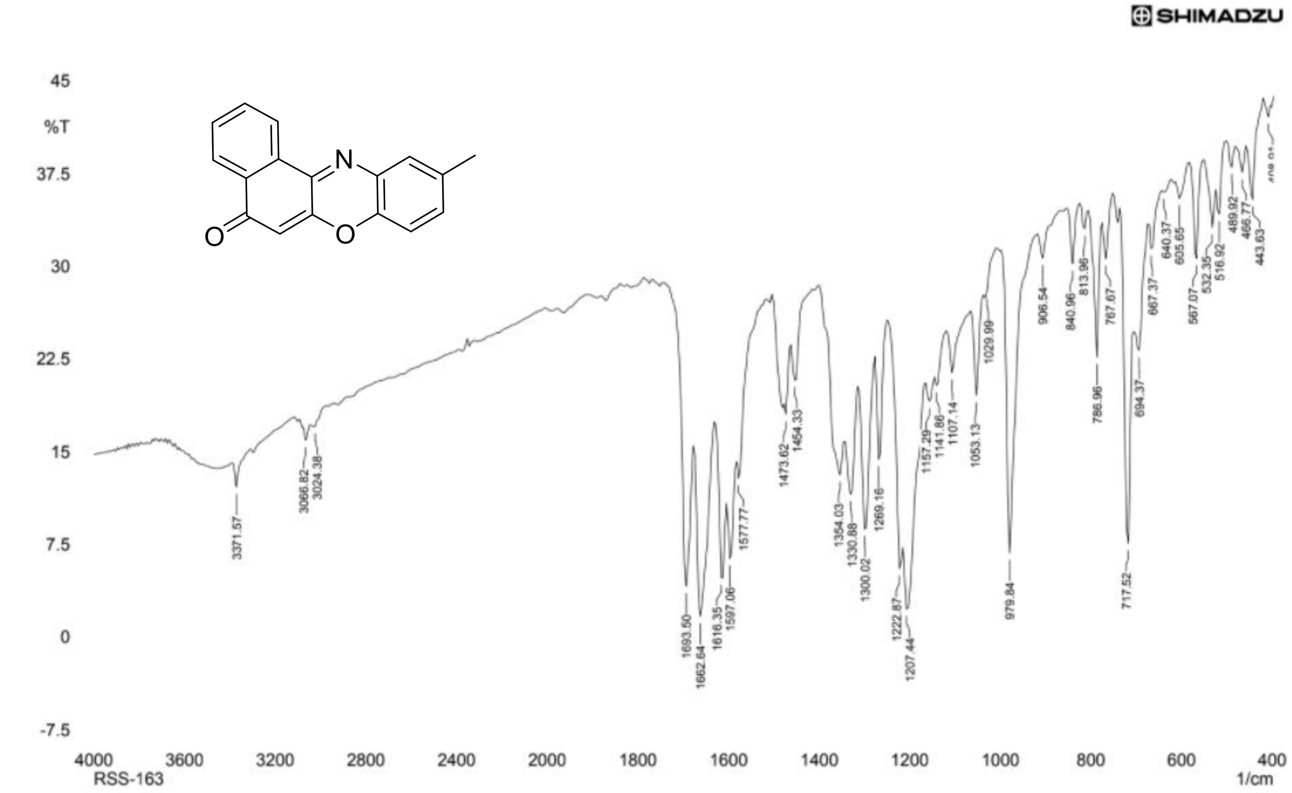

Figure S51. IR (KBr) spectrum of **6**

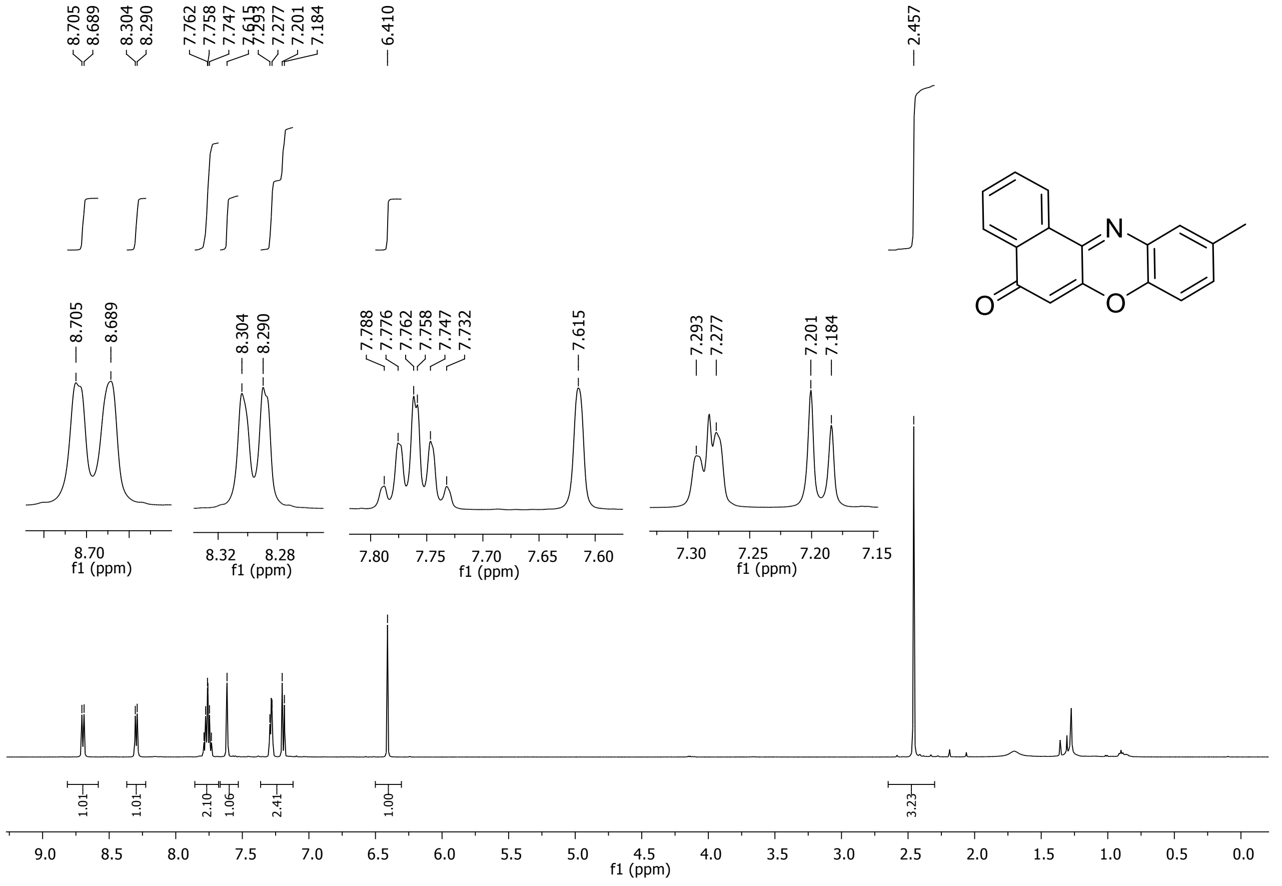

Figure S52. Full ^1H NMR $CDCl_3$ spectrum of **6**

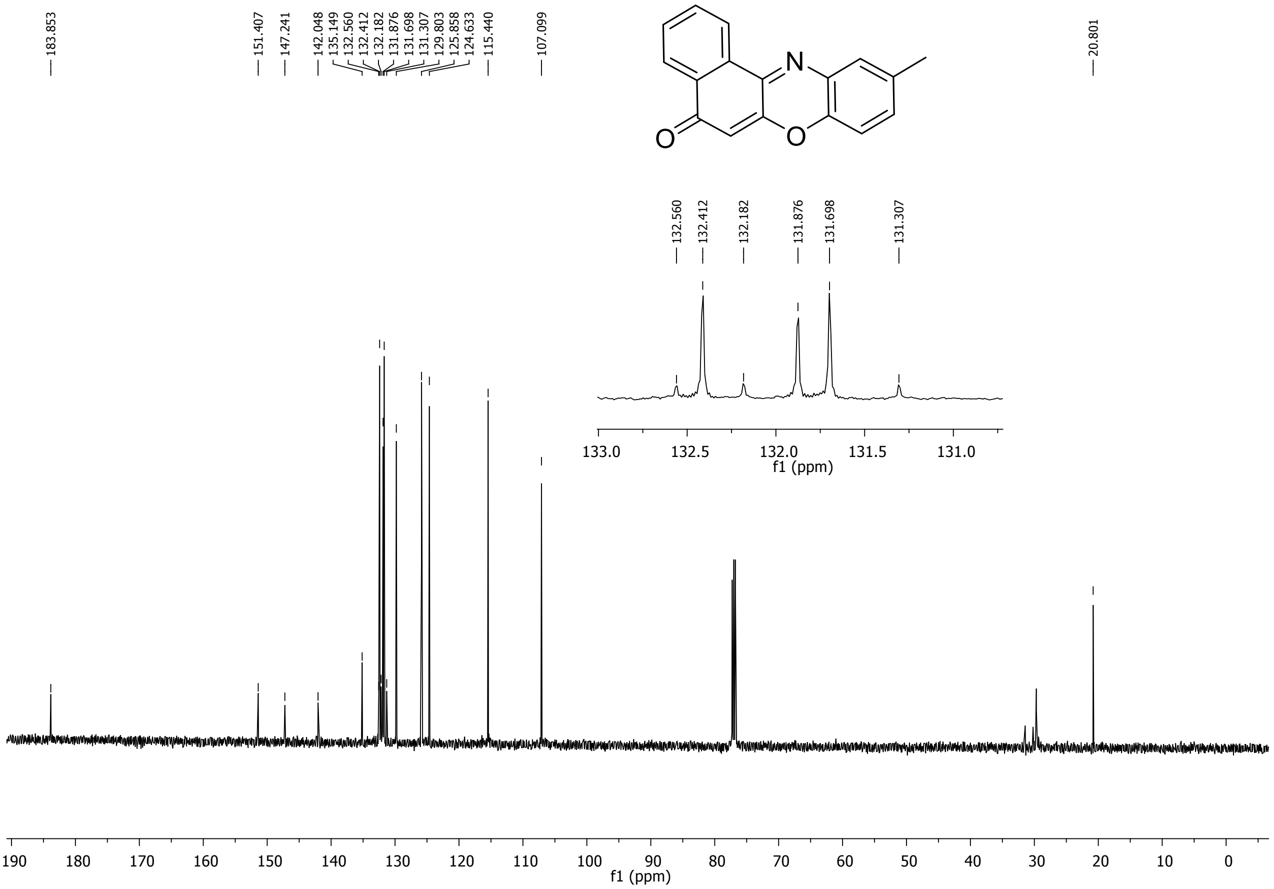

Figure S53. Full $^{13}C\{^1H\}$ NMR $CDCl_3$ spectrum of **6**

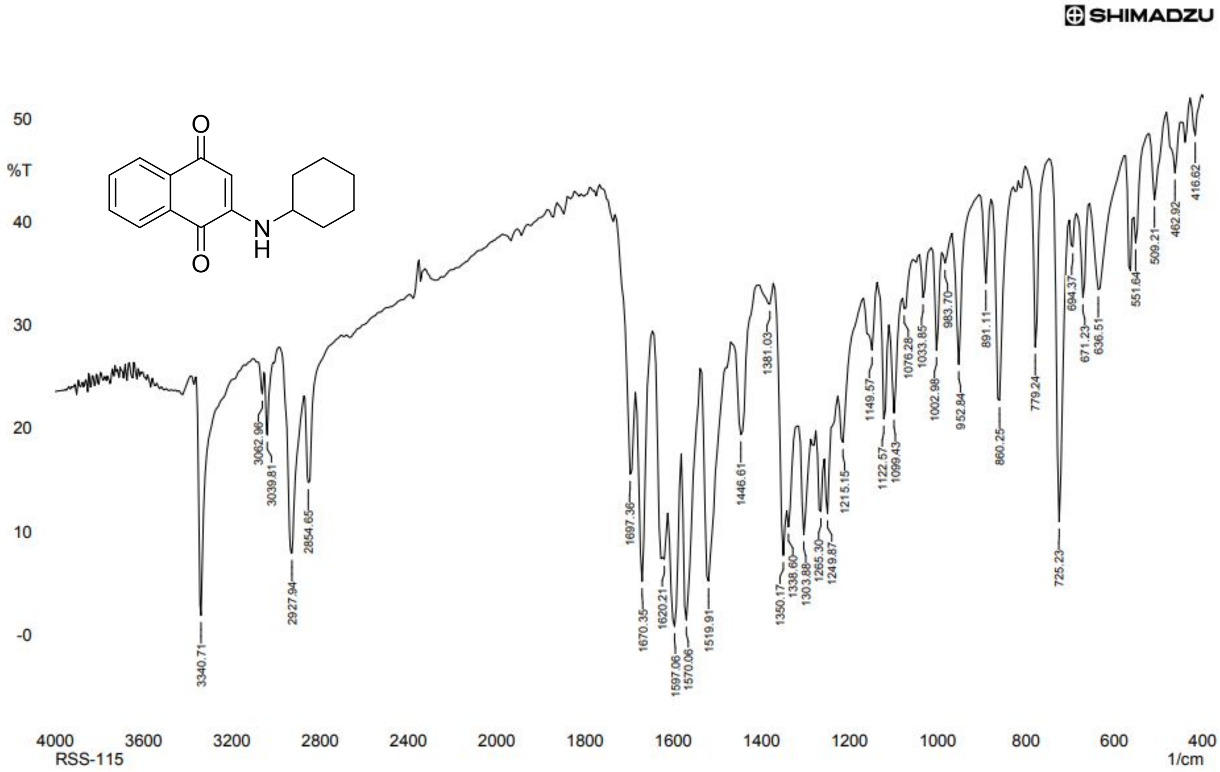

Figure S54. IR (KBr) spectrum of **3q**

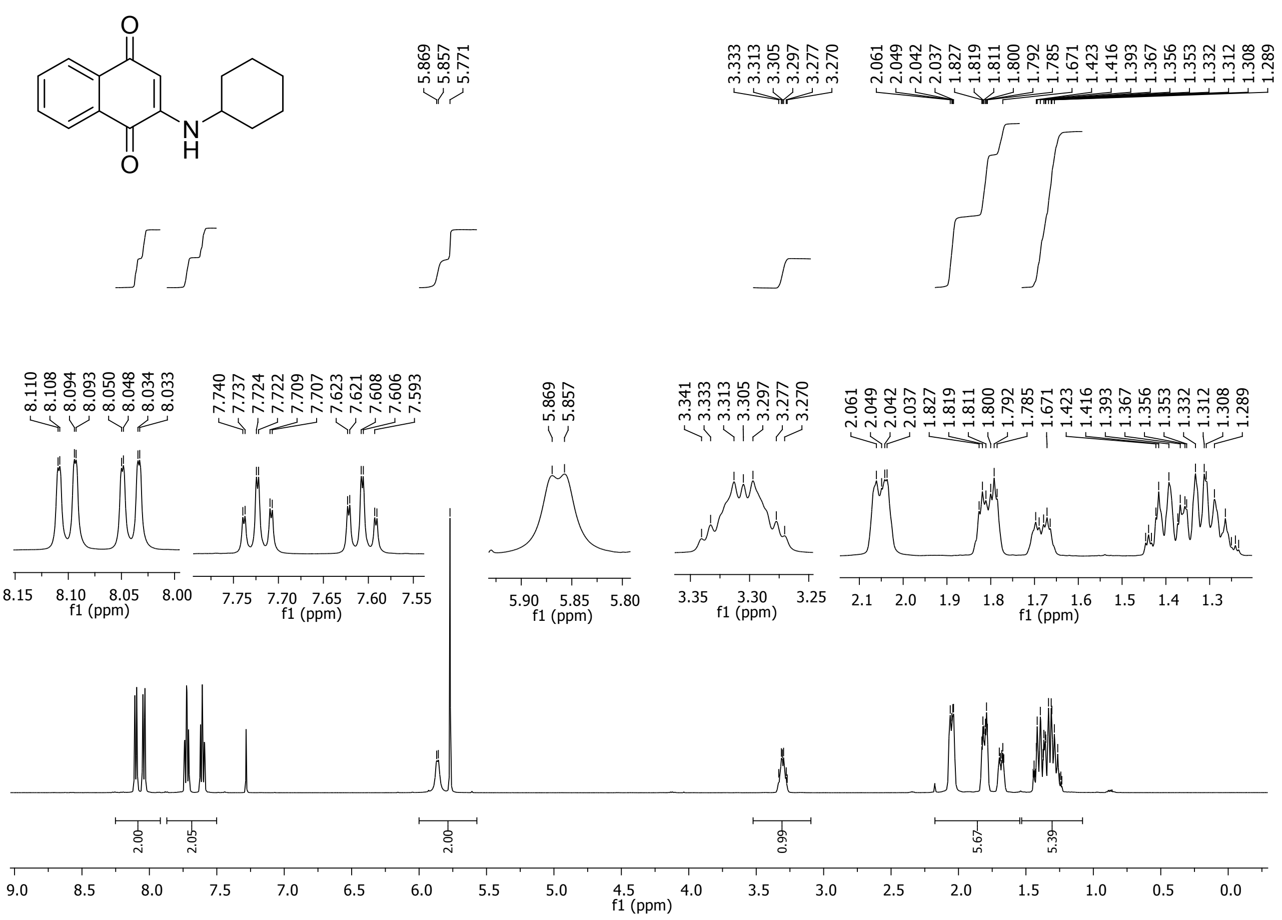

Figure S55. Full ^{1}H NMR $CDCl_3$ spectrum of **3q**

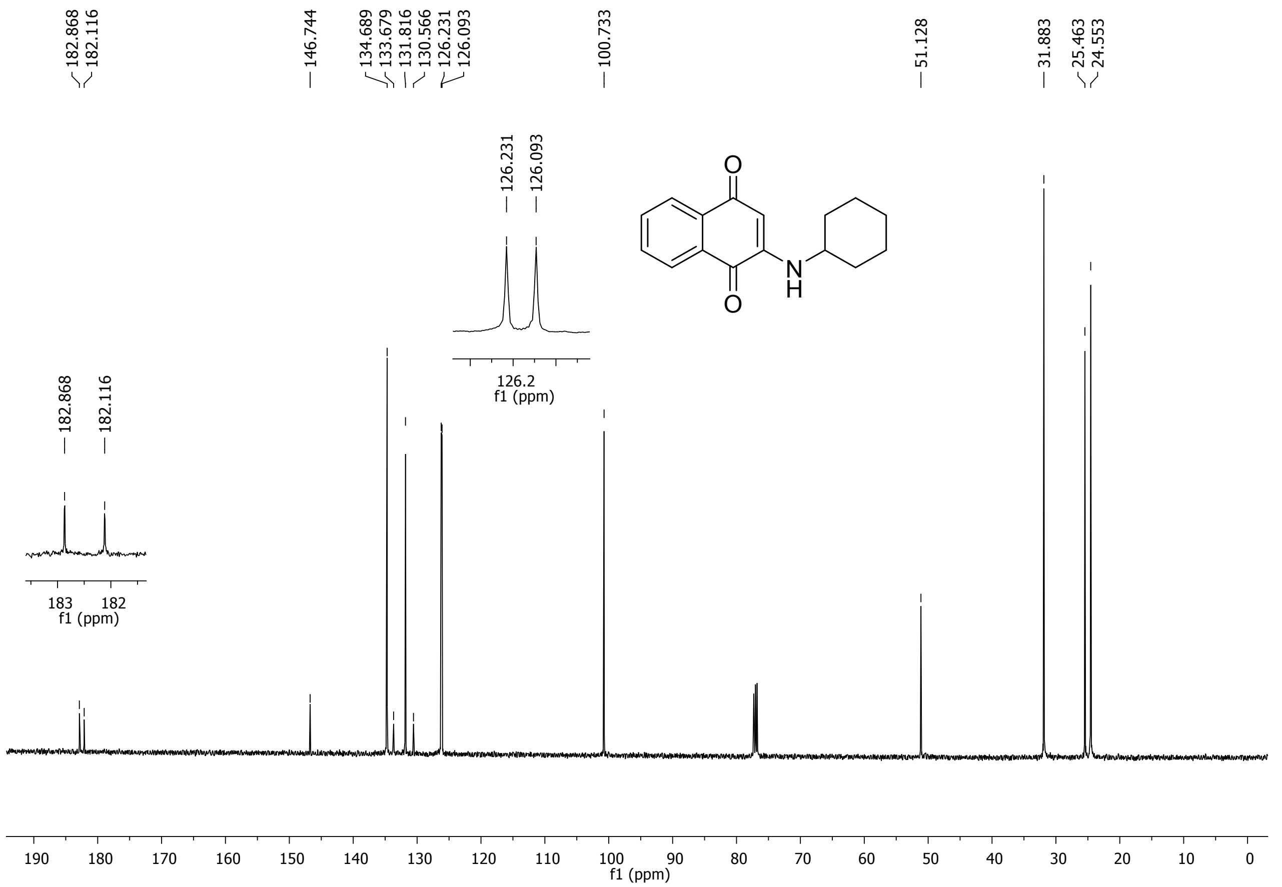

Figure S56. Full $^{13}C\{^{1}H\}$ NMR $CDCl_3$ spectrum of **3q**

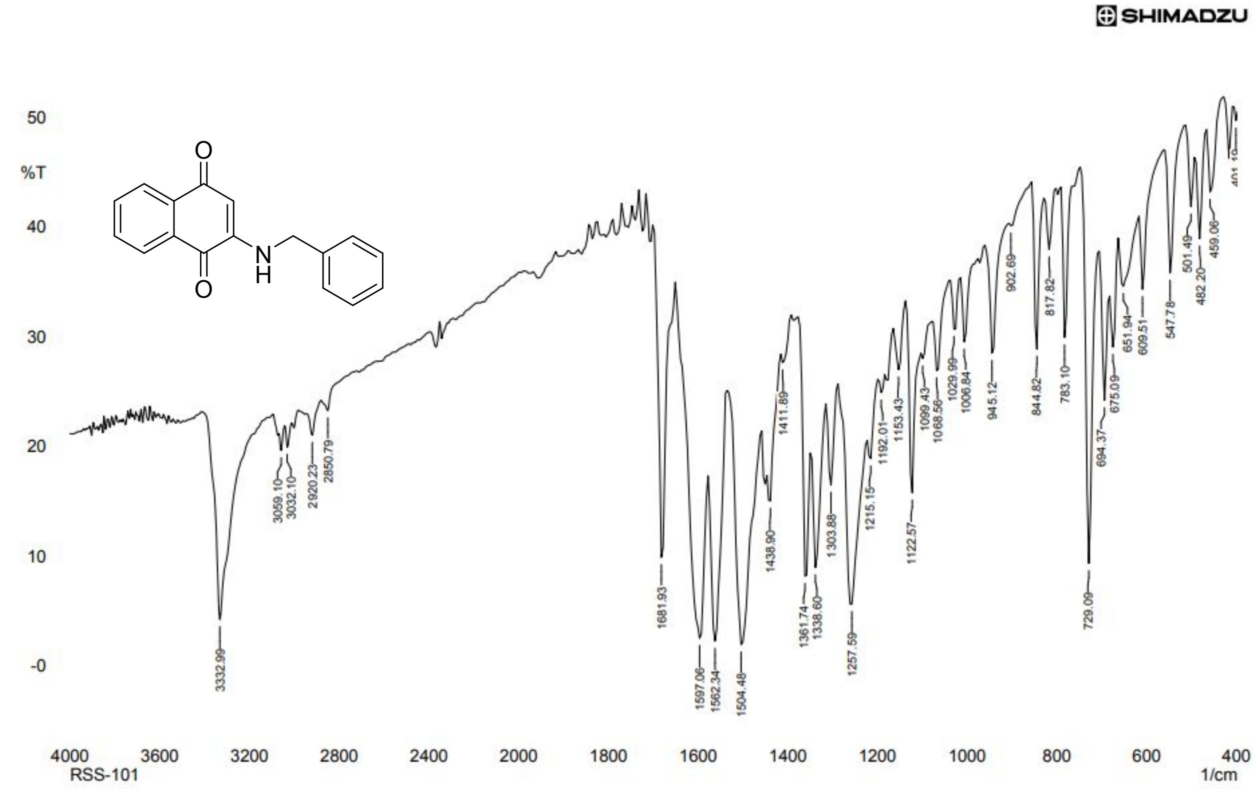

Figure S57. IR (KBr) spectrum of **3r**

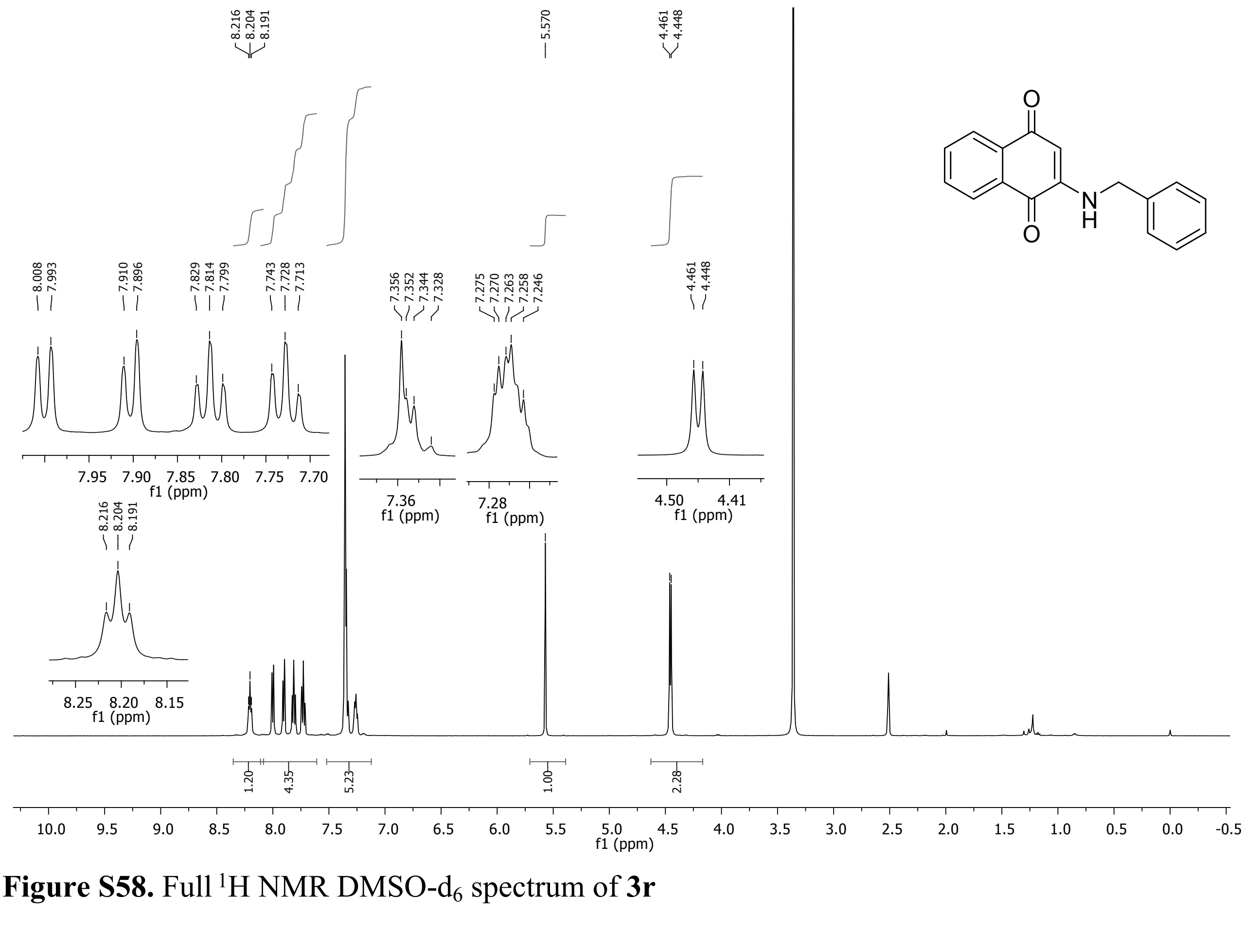

Figure S58. Full ^{1}H NMR DMSO-d_6 spectrum of **3r**

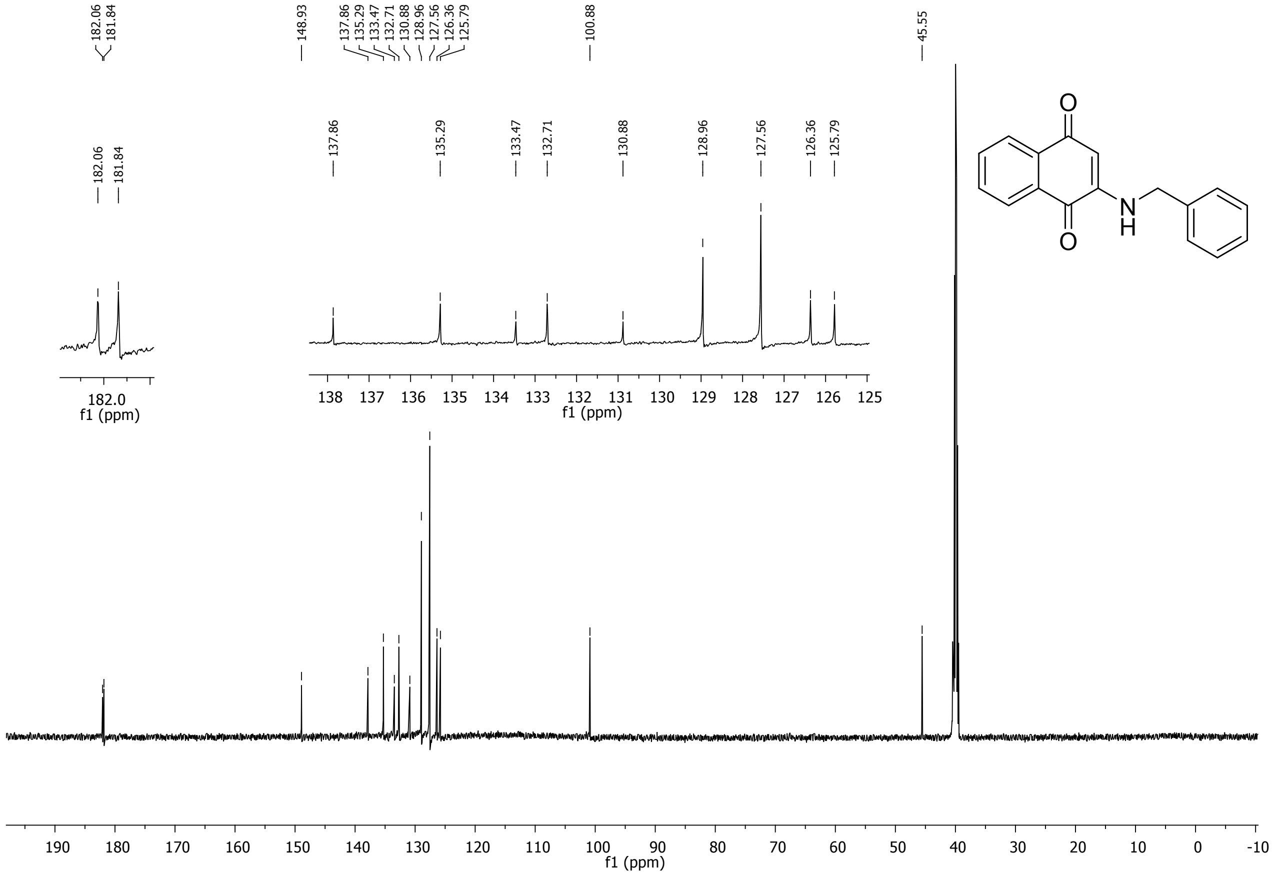

Figure S59. Full $^{13}C\{^{1}H\}$ NMR DMSO-d$_6$ spectrum of **3r**

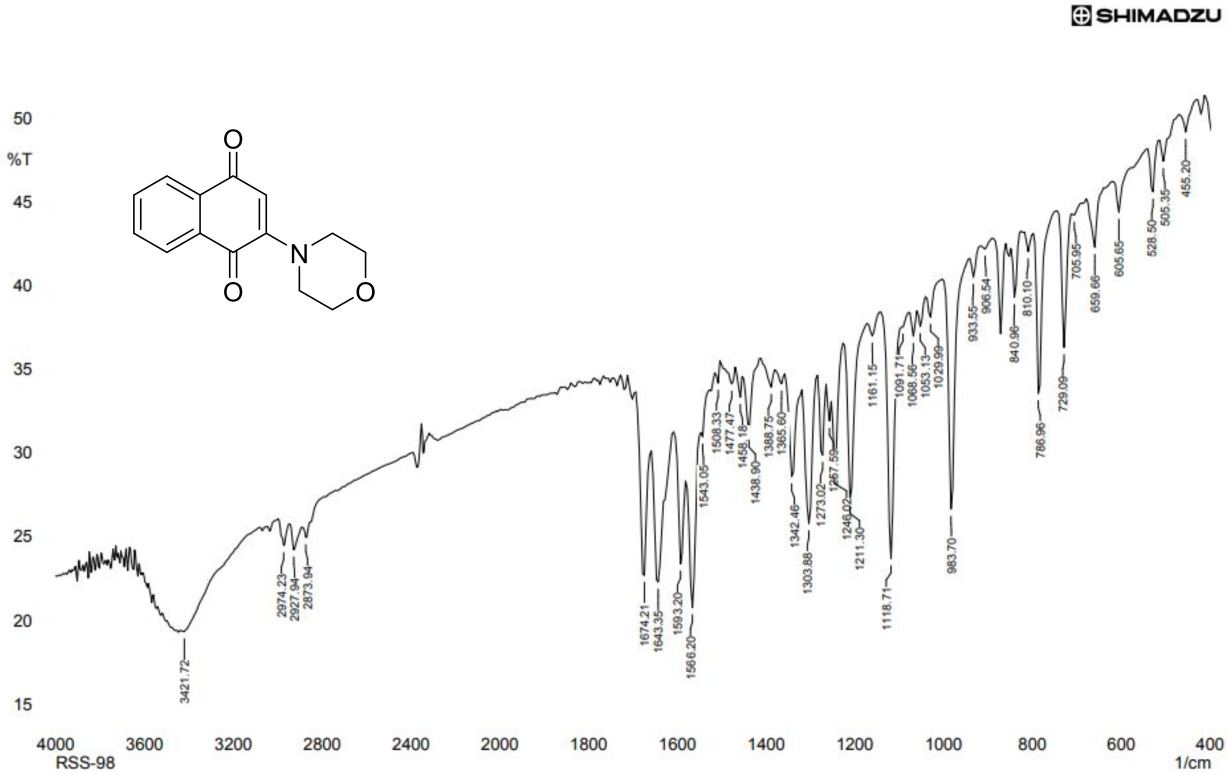

Figure S60. IR (KBr) spectrum of **3s**

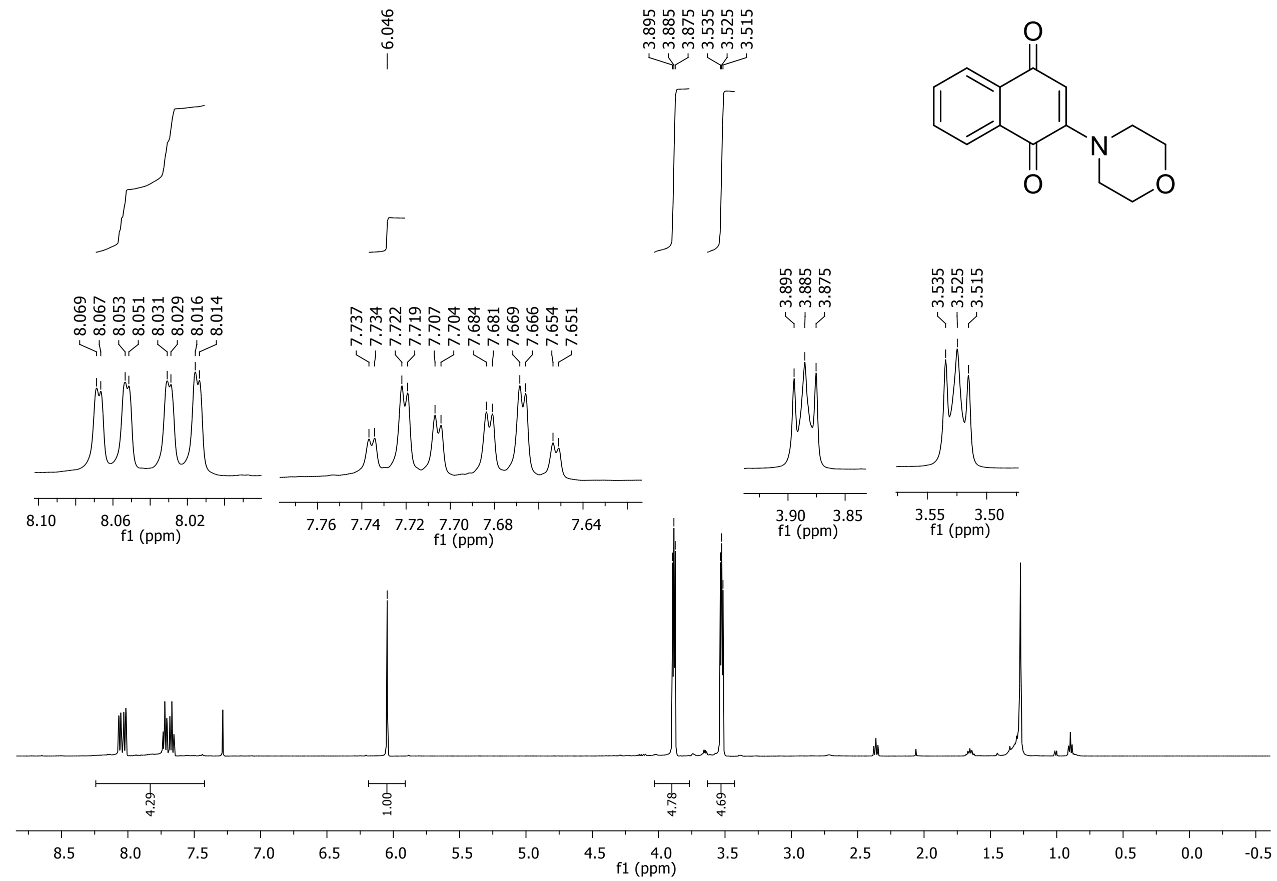

Figure S61. Full ^{1}H NMR $CDCl_3$ spectrum of **3s**

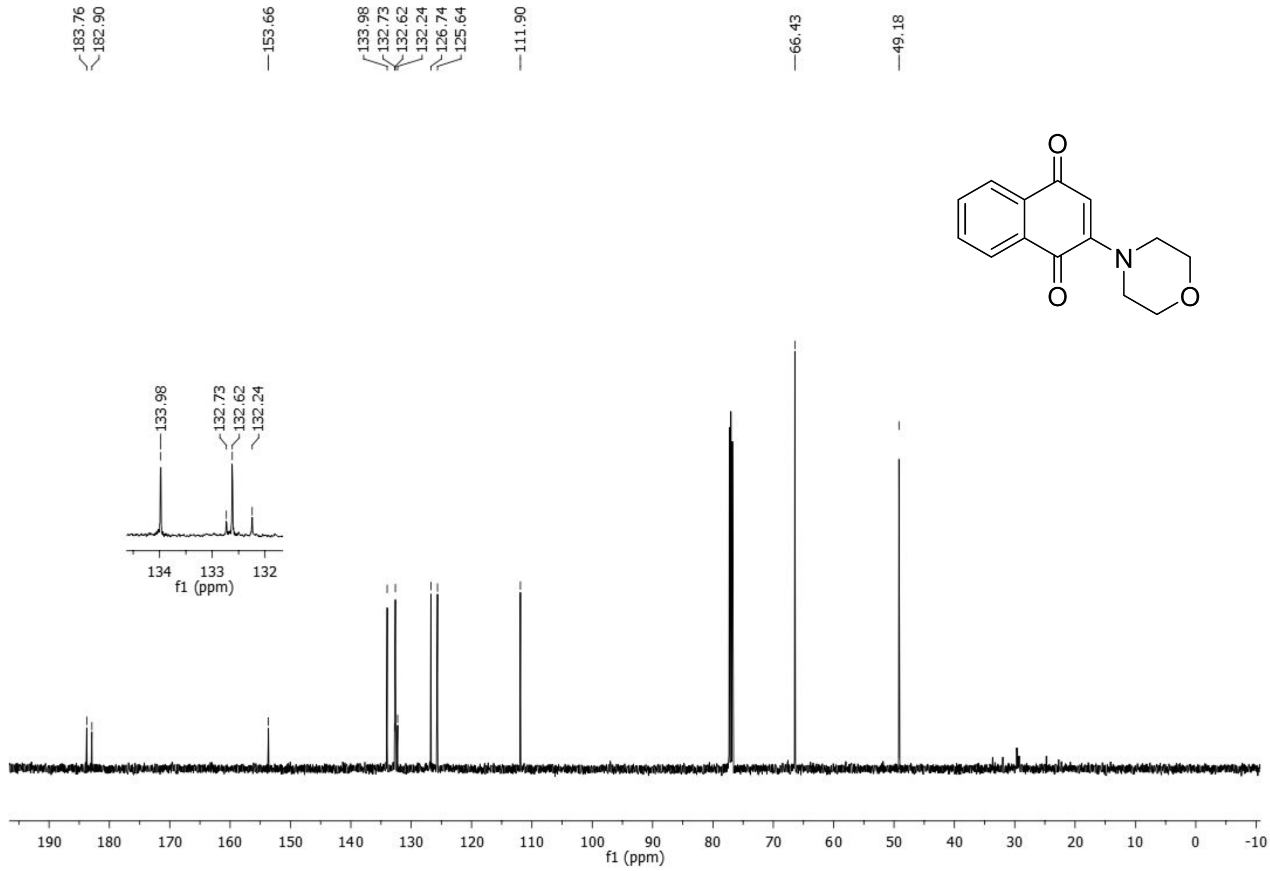

Figure S62. Full $^{13}C\{^{1}H\}$ NMR $CDCl_3$ spectrum of **3s**

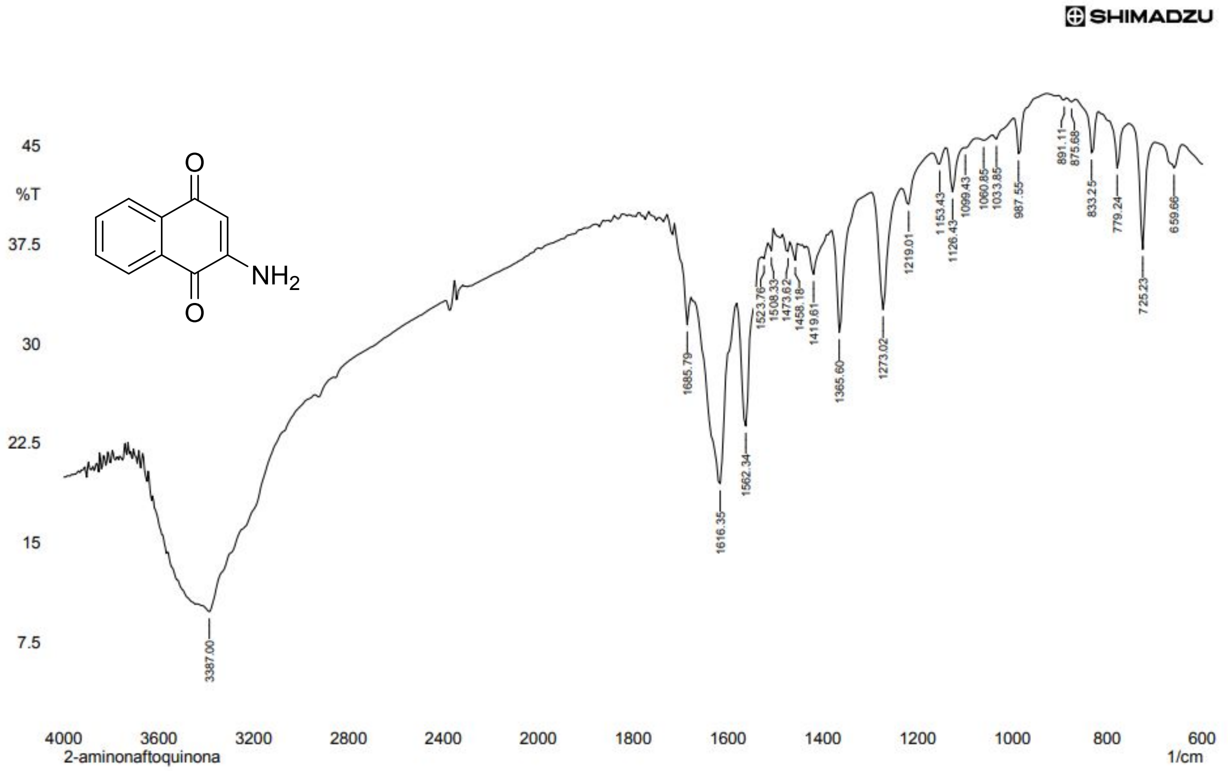

Figure S63. IR (KBr) spectrum of **7**

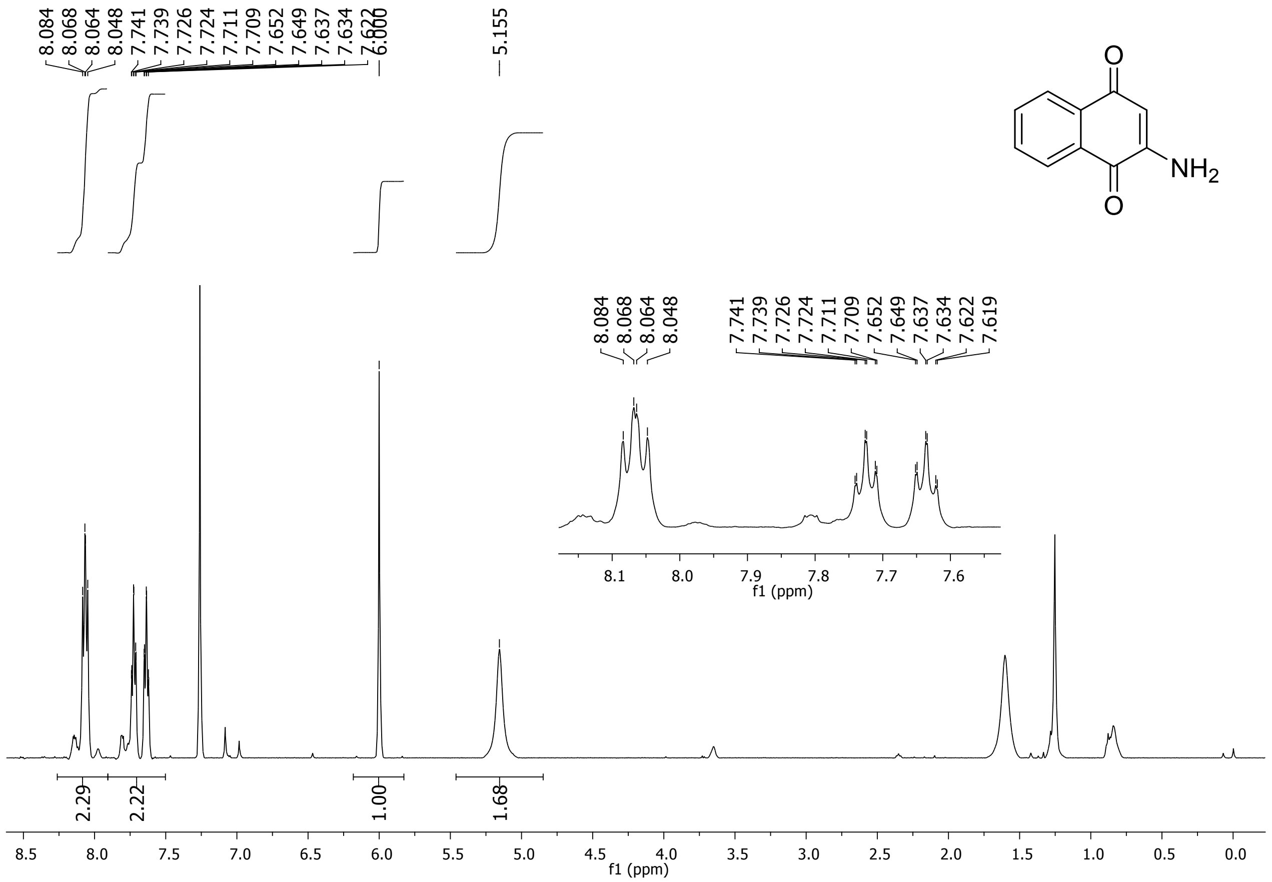

Figure S64. Full ^{1}H NMR $CDCl_3$ spectrum of **7**

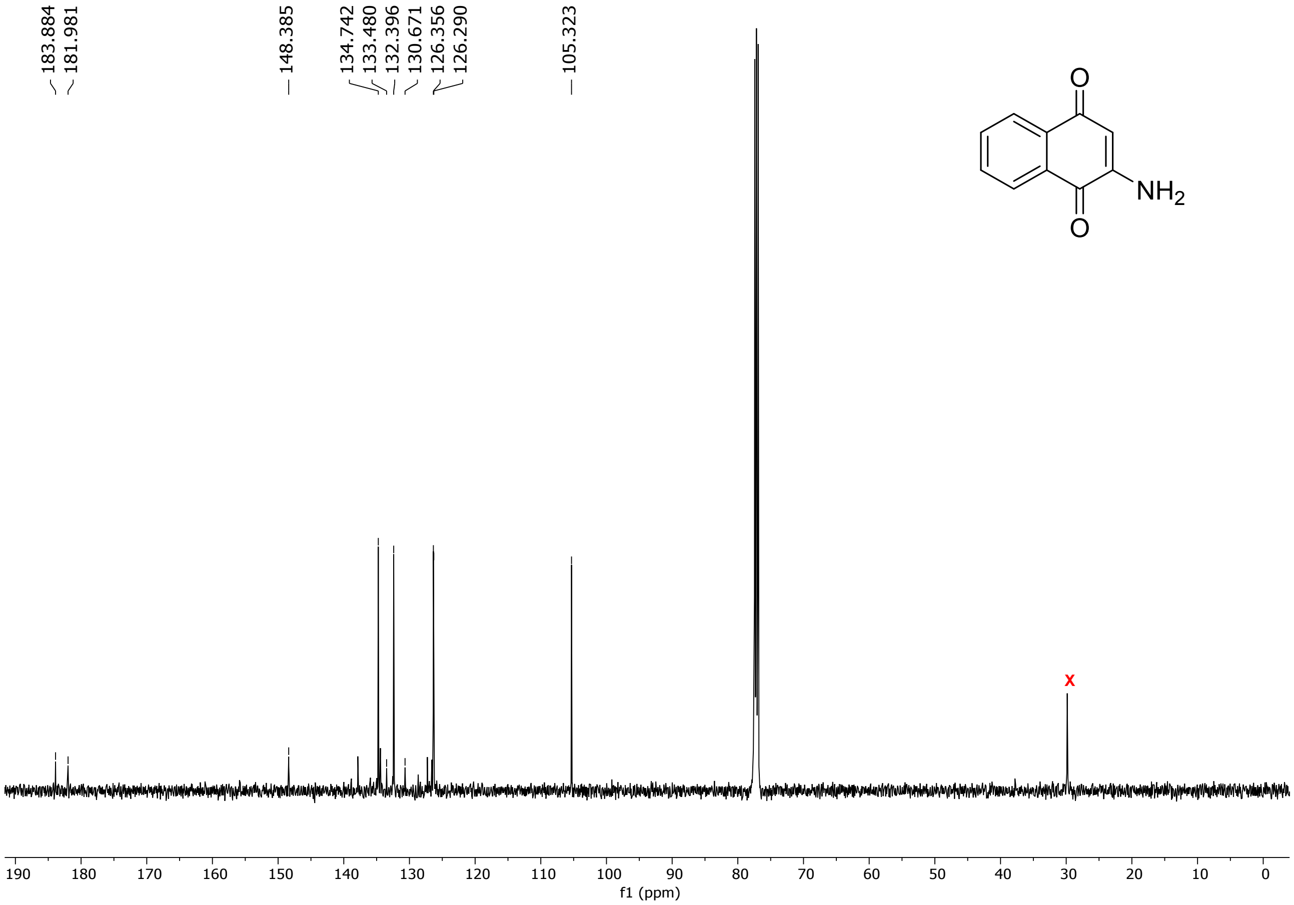

Figure S65. Full $^{13}C\{^{1}H\}$ NMR $CDCl_3$ spectrum of **7**

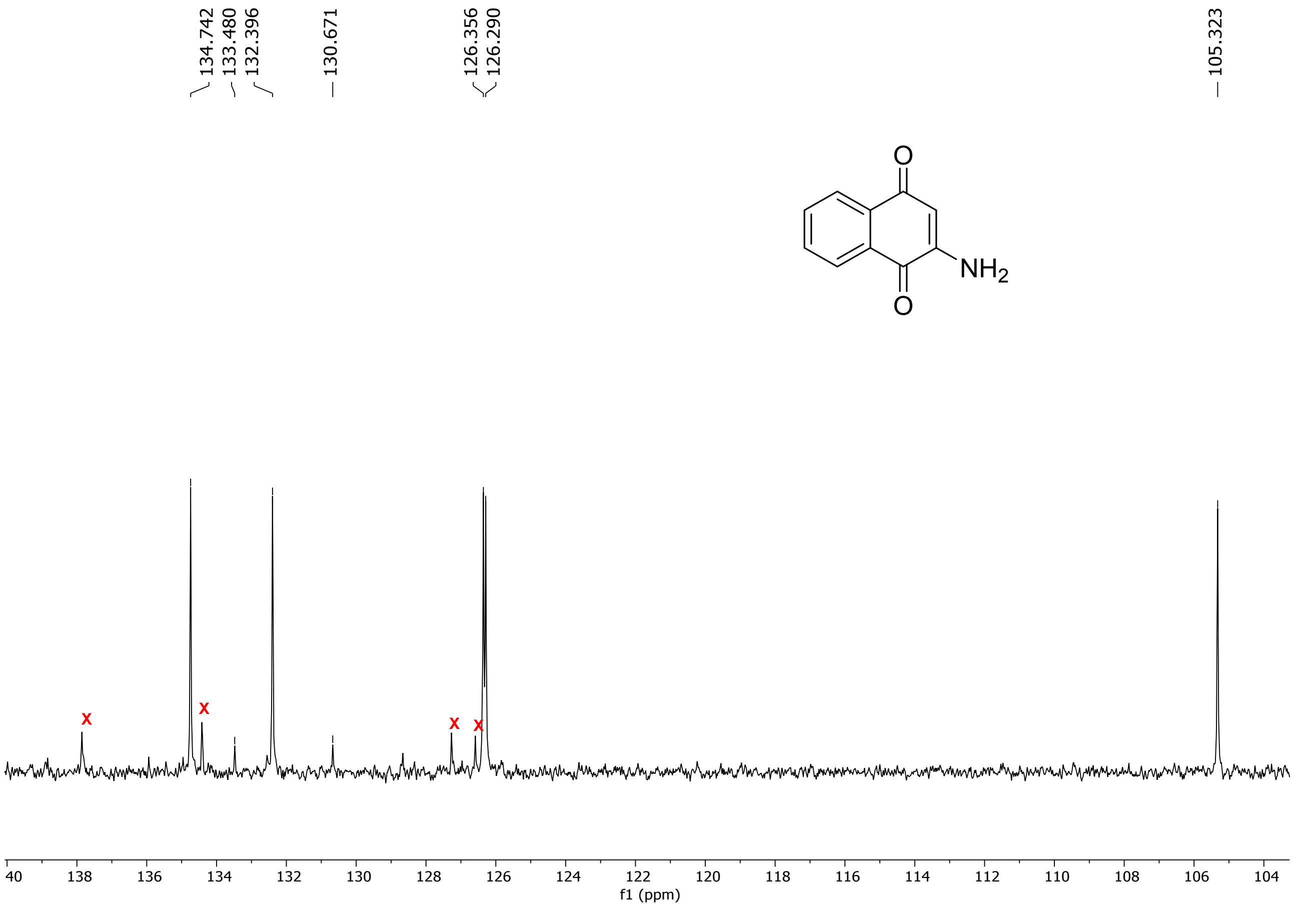

Figure S66. Expanded $^{13}C\{^{1}H\}$ NMR $CDCl_3$ spectrum of **7**

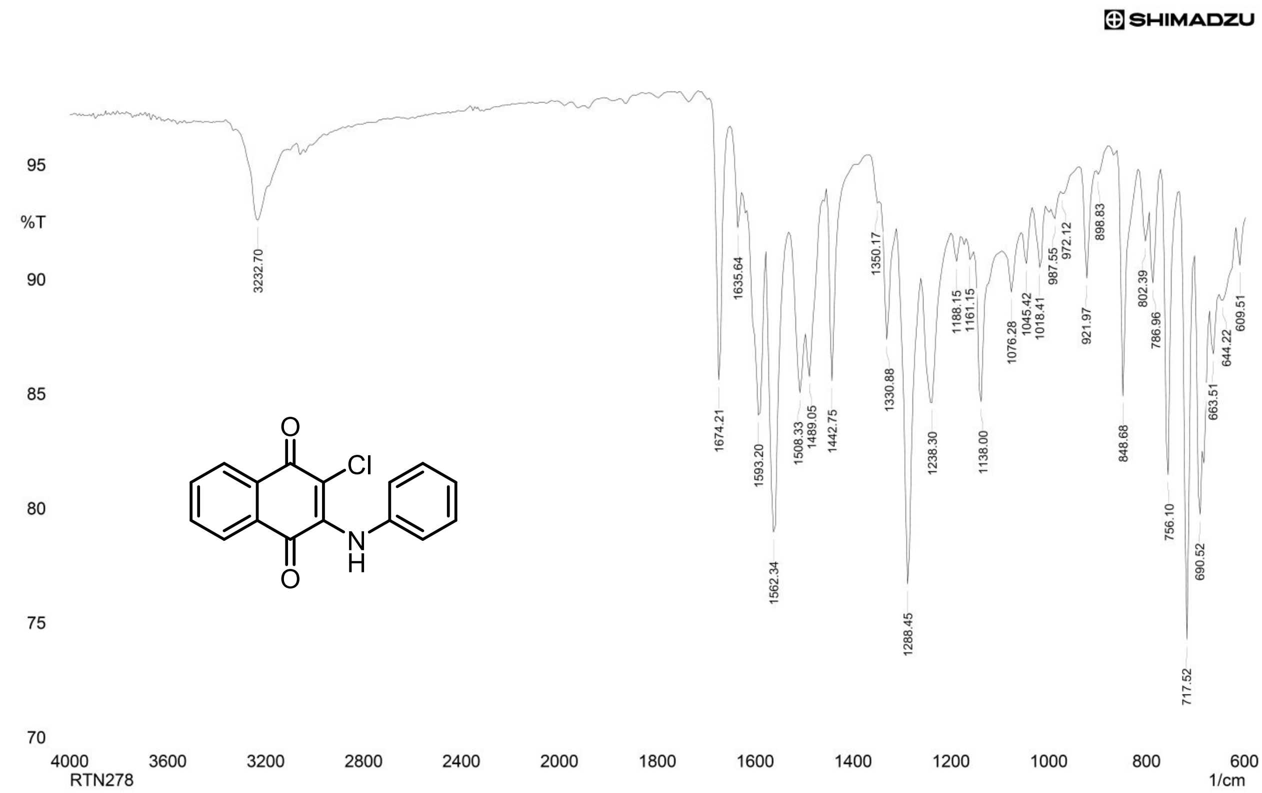

Figure S67. IR (ATR) spectrum of **3a’** (from **1b**)

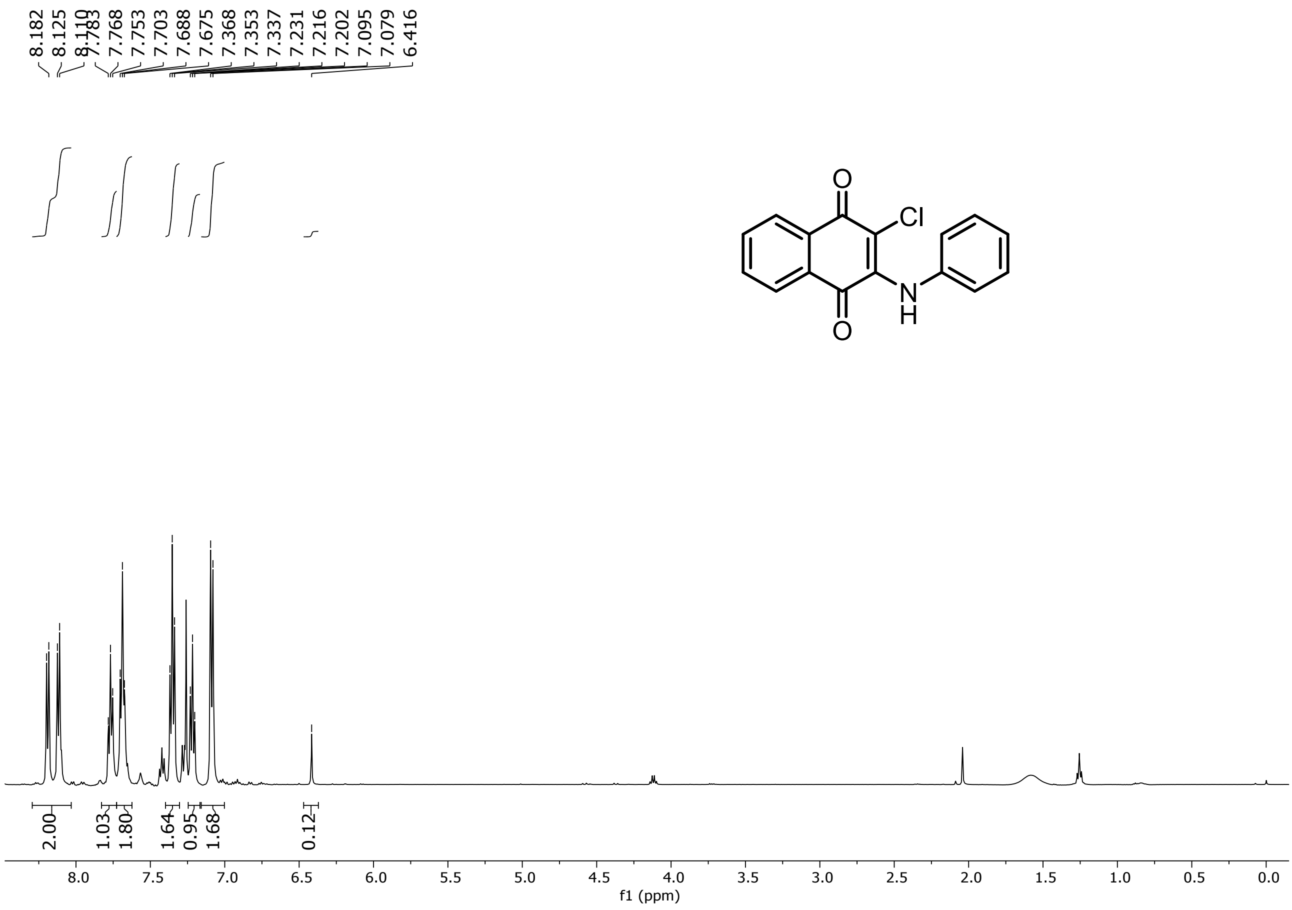

Figure S68. Full ^{1}H NMR $CDCl_3$ spectrum of **3a’** (from **1b**)

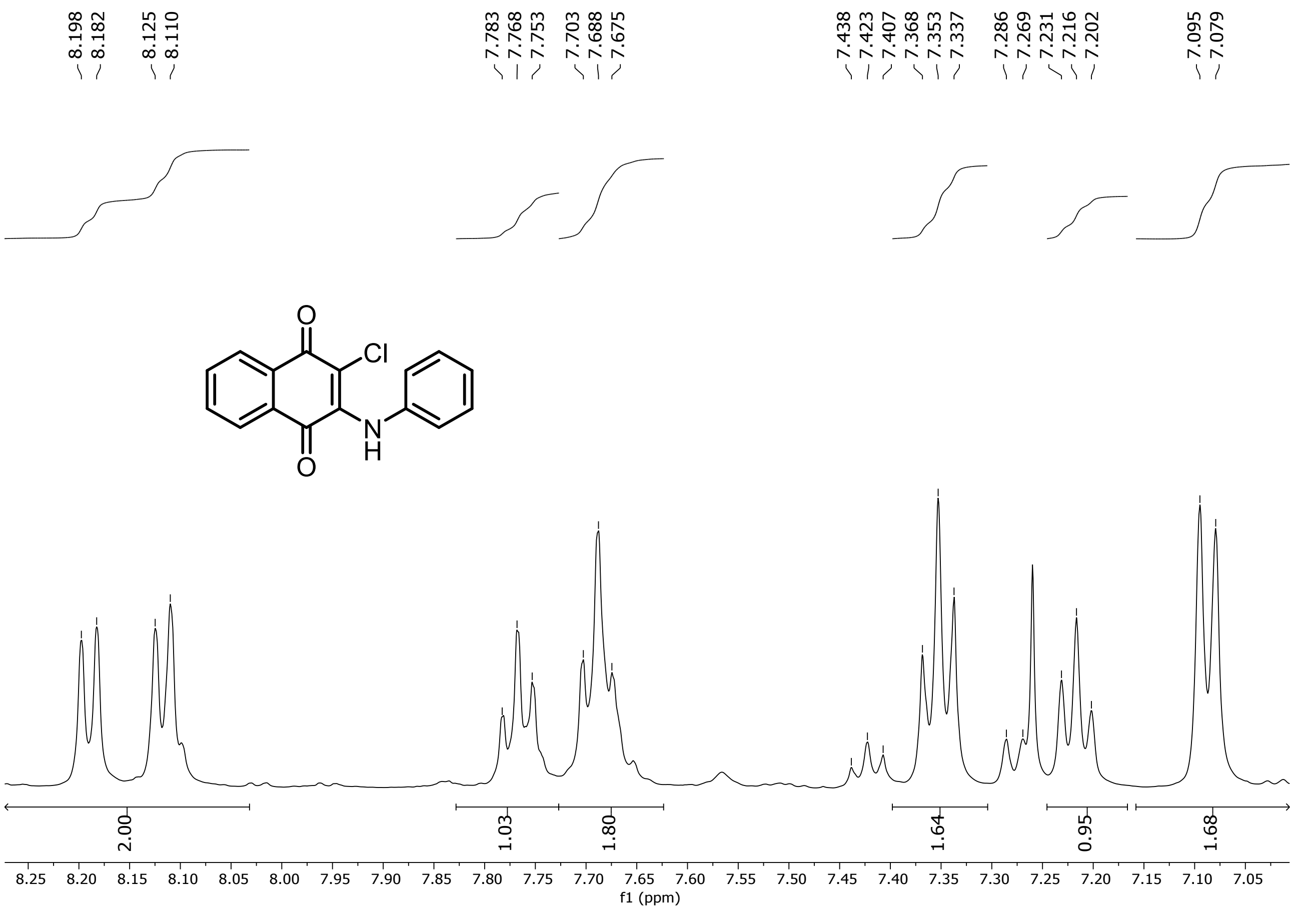

Figure S69. Expanded ^{1}H NMR $CDCl_3$ spectrum of **3a'** (from **1b**)

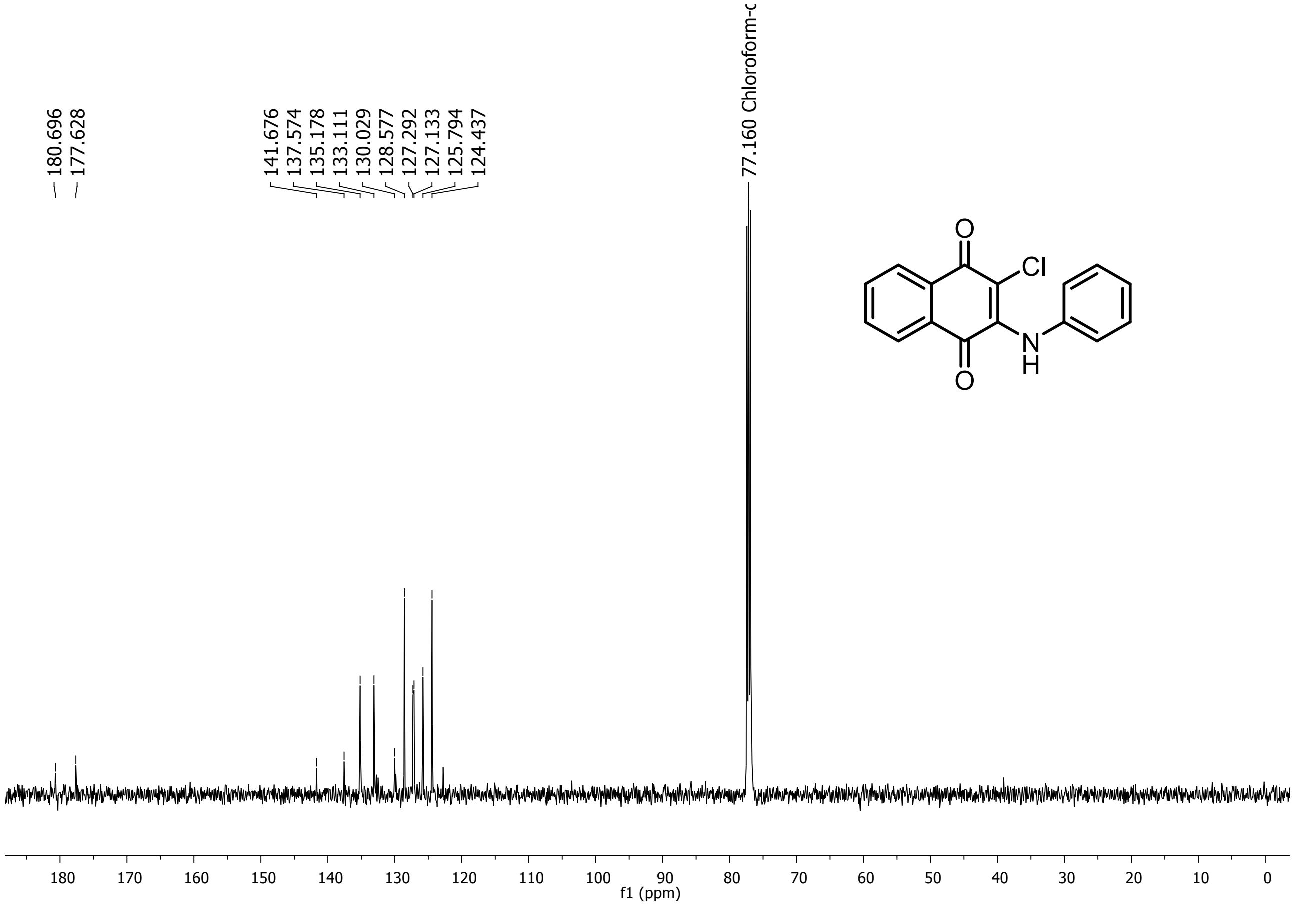

Figure S70. Full $^{13}C\{^{1}H\}$ NMR $CDCl_3$ spectrum of **3a'** (from **1b**)

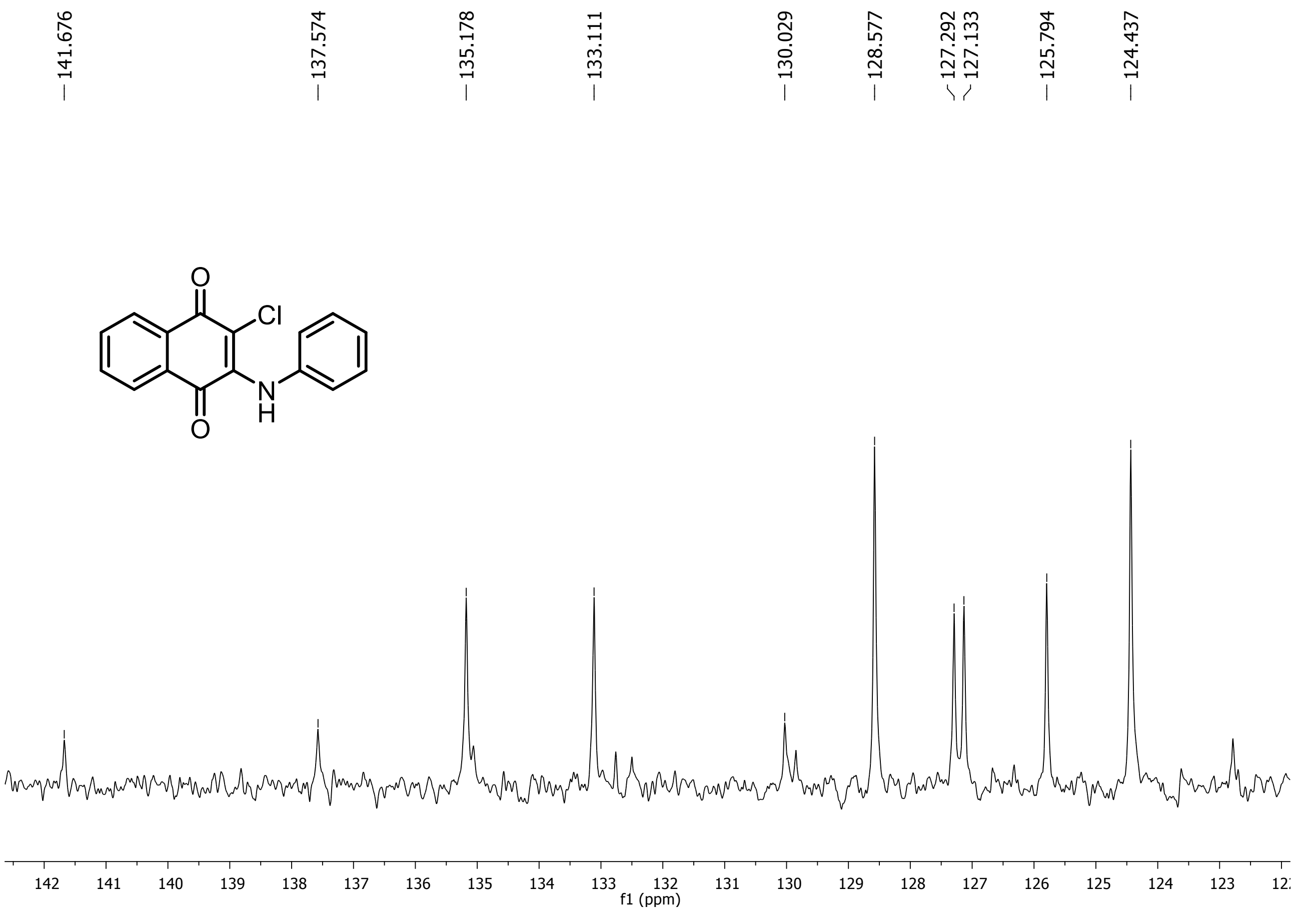

Figure S71. Expanded $^{13}C\{^{1}H\}$ NMR $CDCl_3$ spectrum of **3a'** (from **1b**)

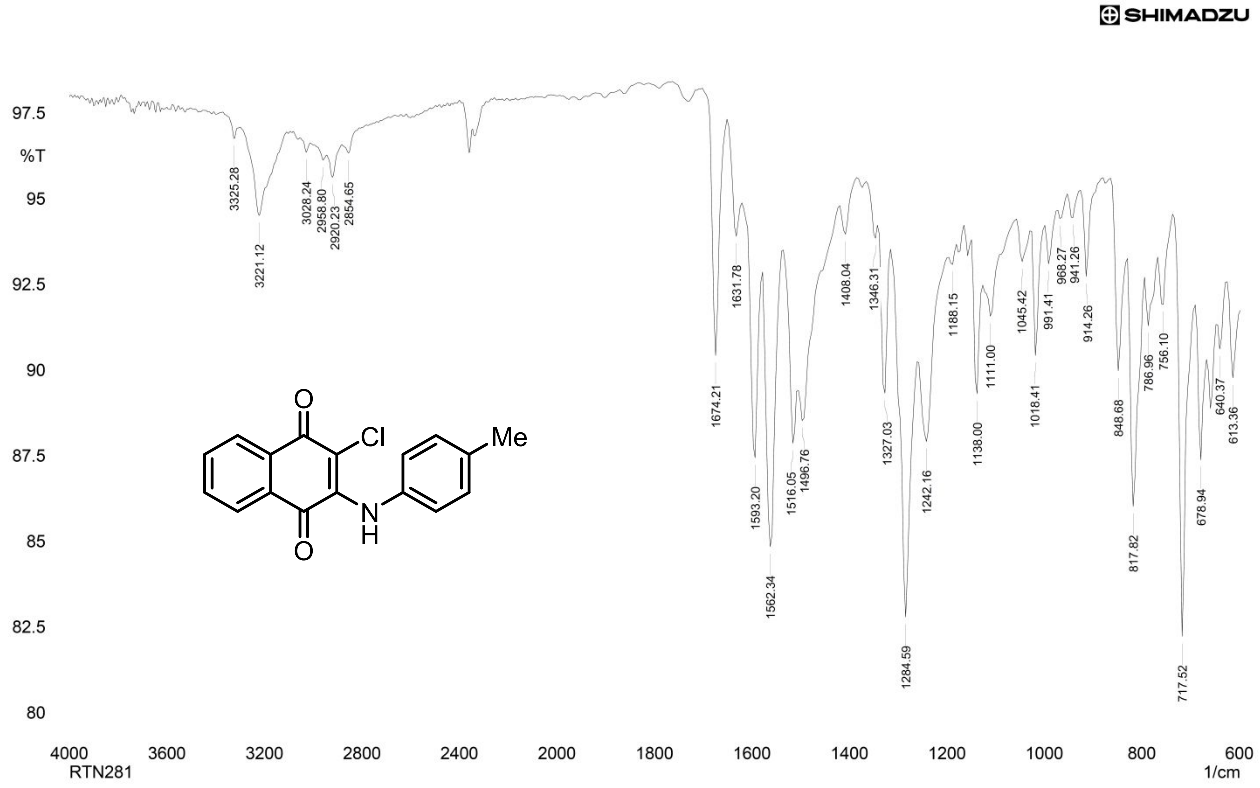

Figure S72. IR (ATR) spectrum of **3b'** (from **1b**)

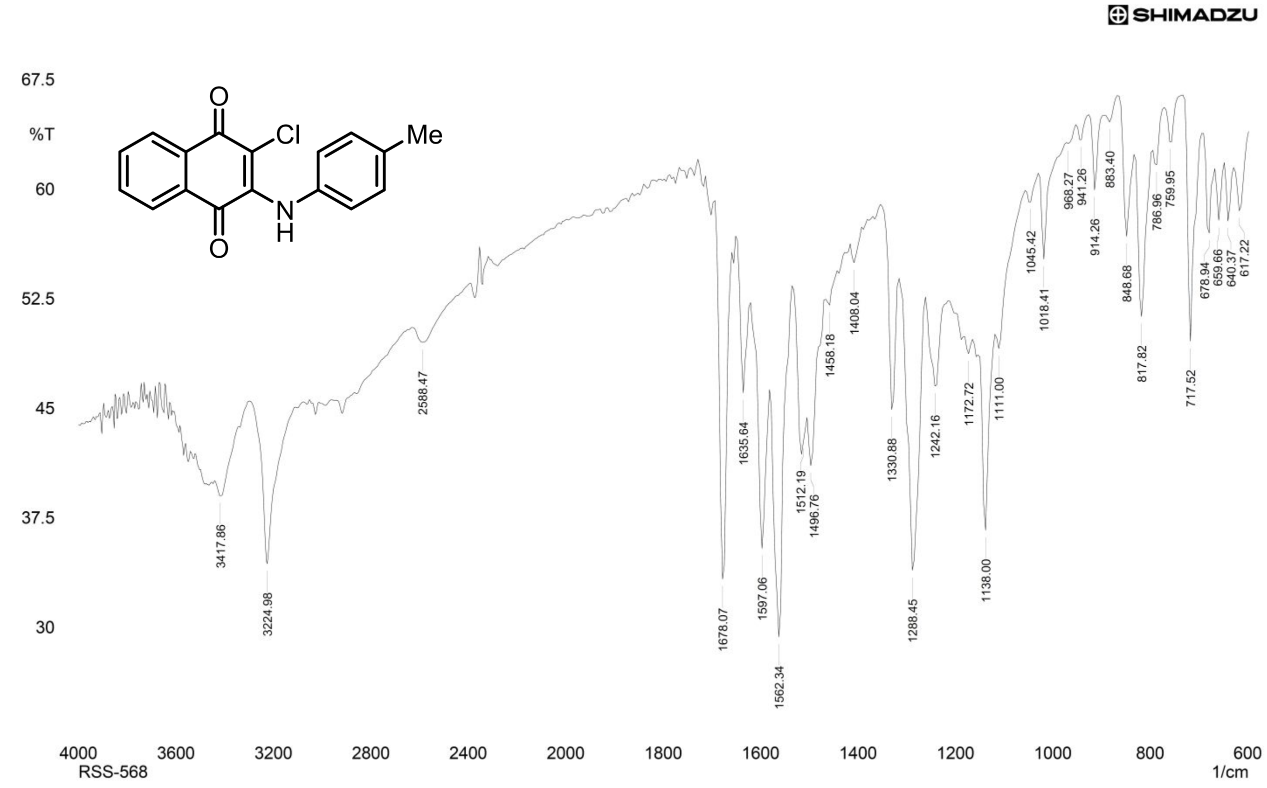

Figure S73. IR (KBr) spectrum of **3b'** (from **1c**)

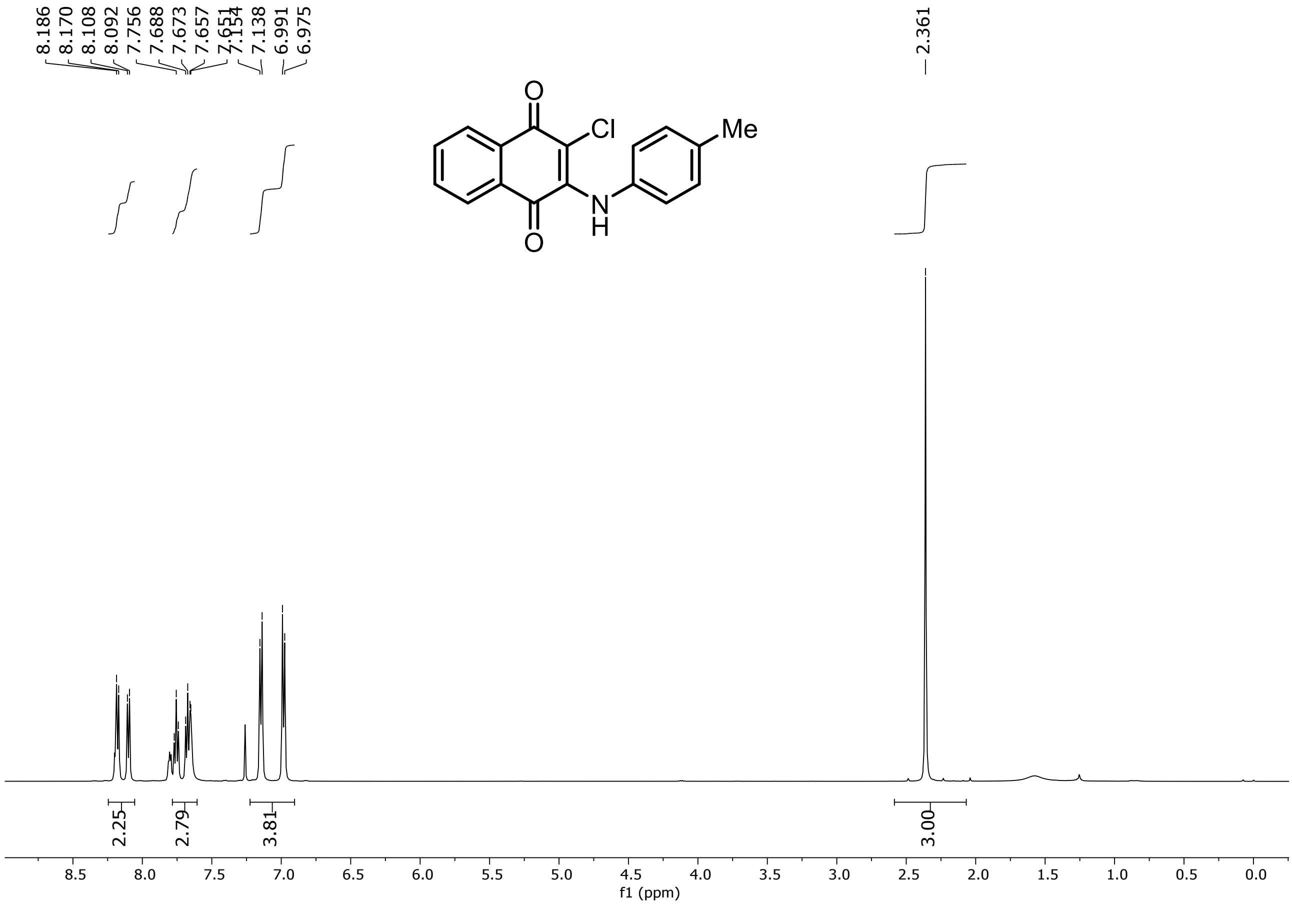

Figure S74. Full ^{1}H NMR $CDCl_3$ spectrum of **3b'** (from **1c**)

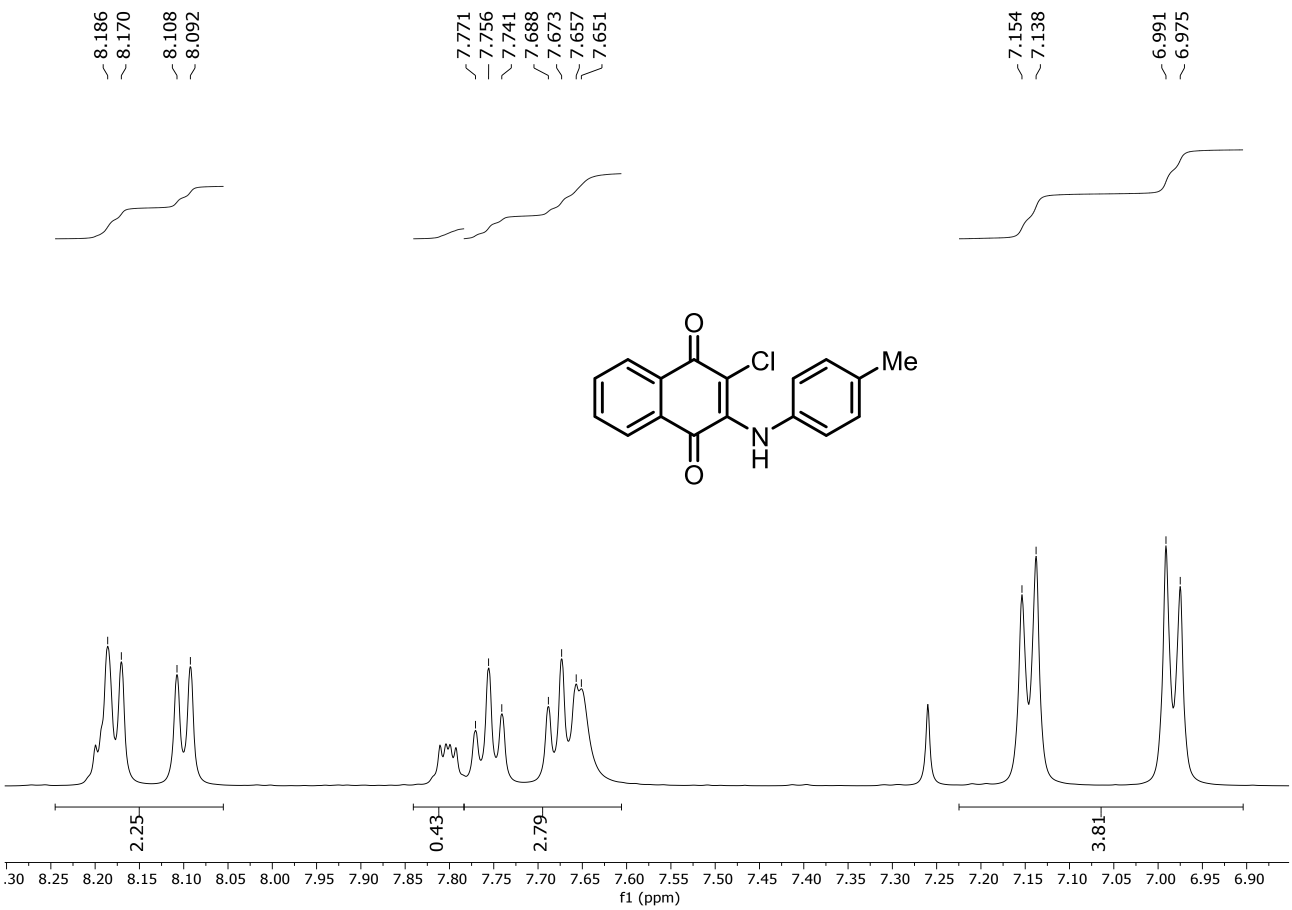

Figure S75. Expanded ^{1}H NMR $CDCl_3$ spectrum of **3b'** (from **1c**)

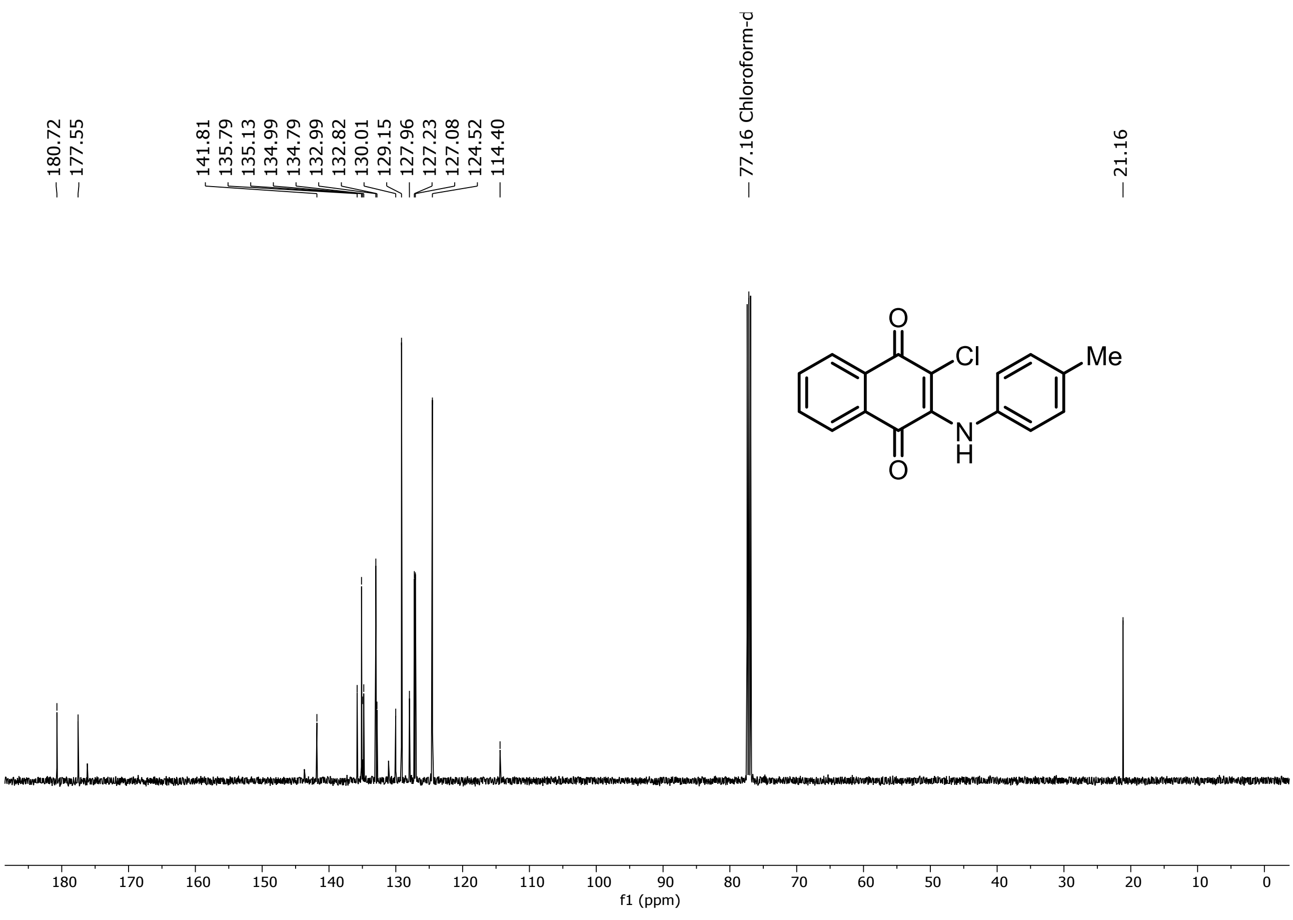

Figure S76. Full $^{13}C\{^{1}H\}$ NMR $CDCl_3$ spectrum of **3b'** (from **1c**)

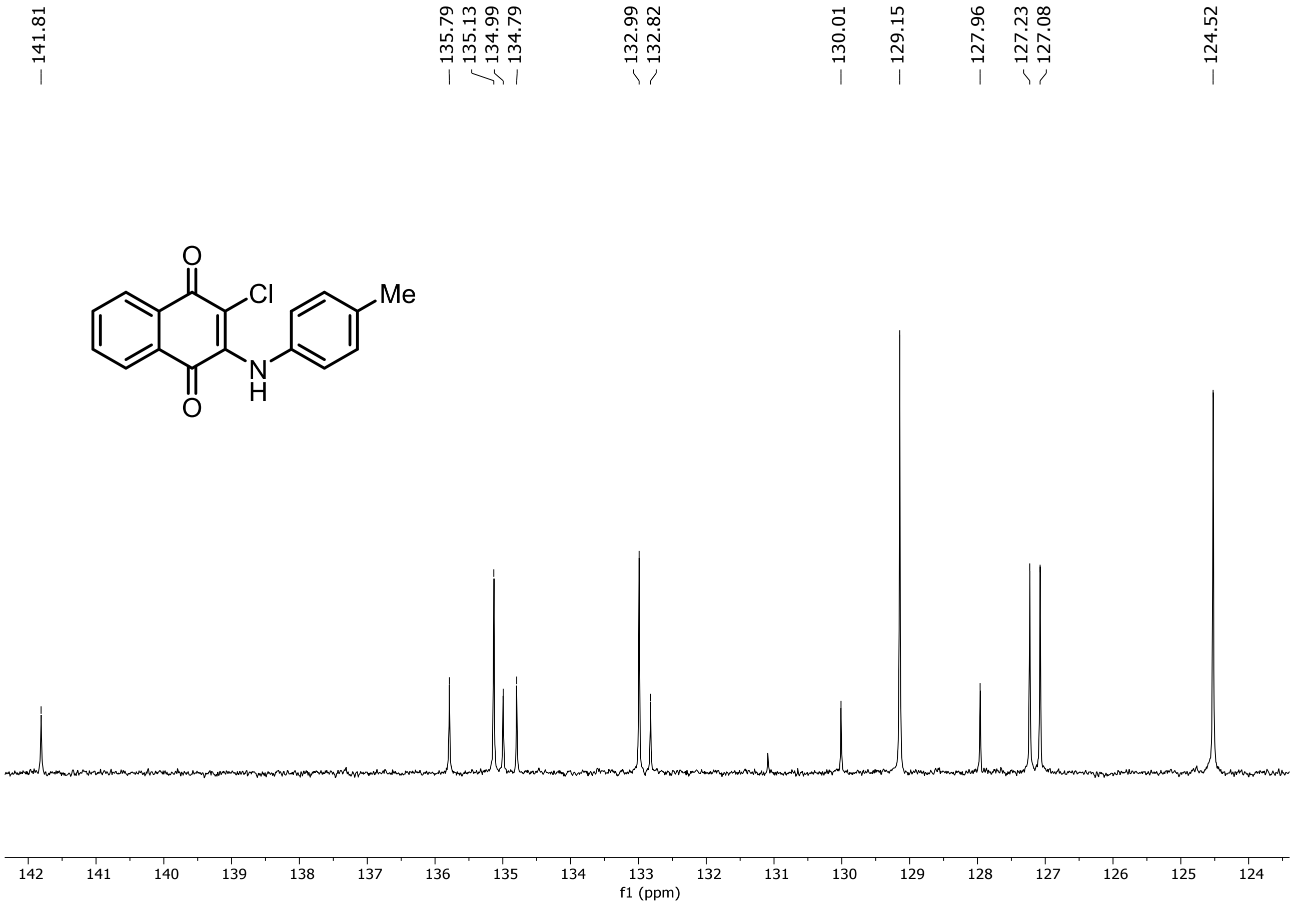

Figure S77. Expanded $^{13}C\{^{1}H\}$ NMR $CDCl_3$ spectrum of **3b'** (from **1c**)

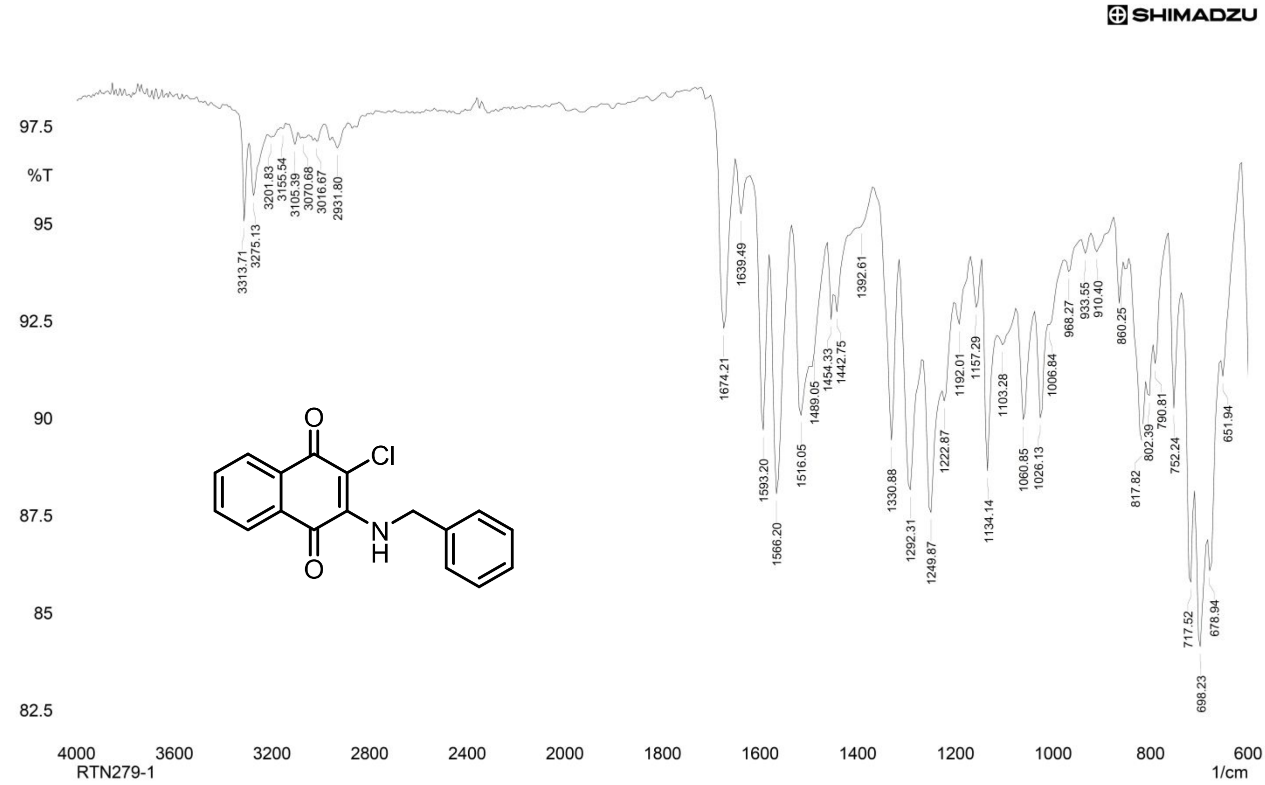

Figure S78. IR (ATR) spectrum of **3r'** (from **1b**)

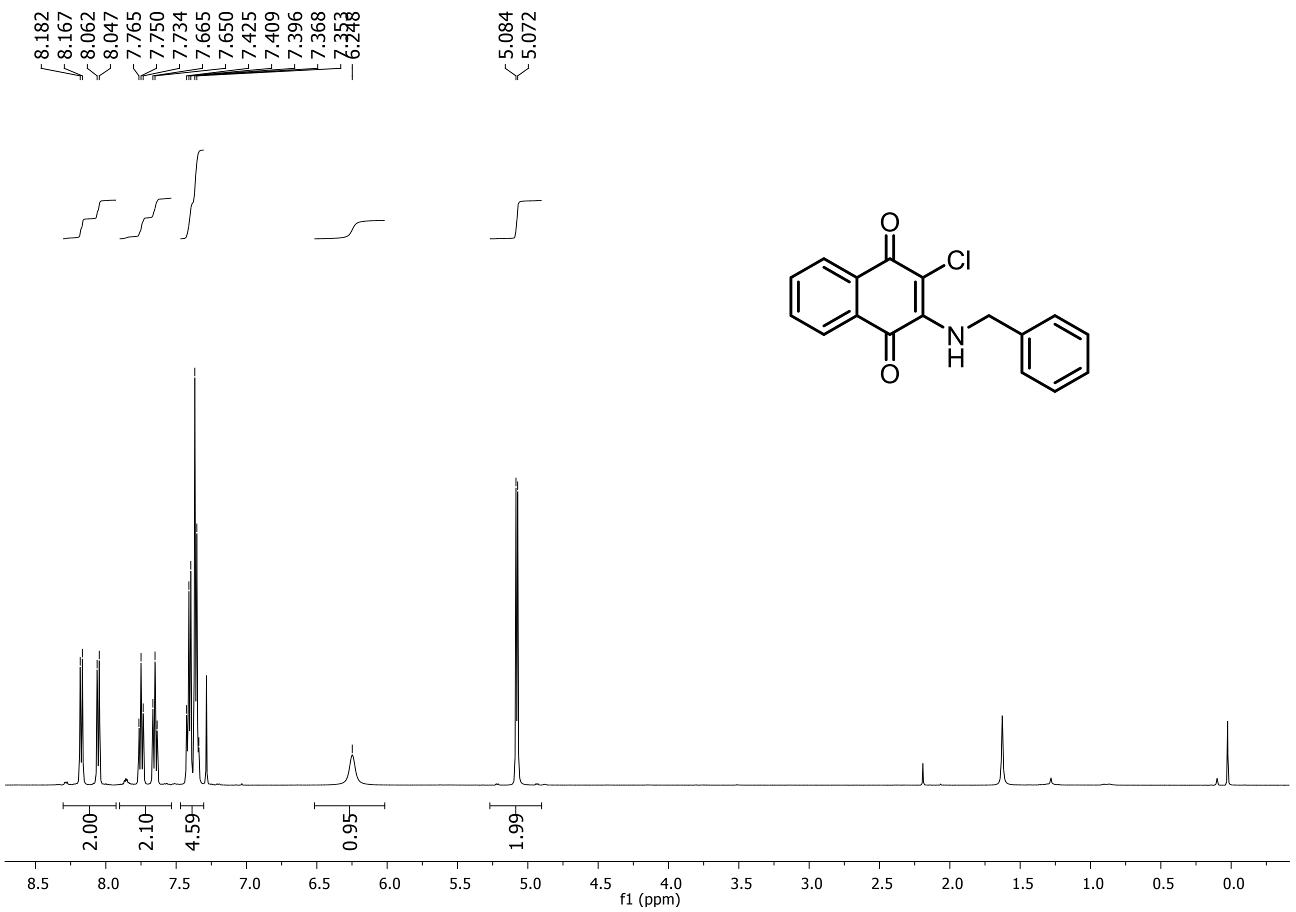

Figure S79. Full ^{1}H NMR $CDCl_3$ spectrum of **3r'** (from **1b**)

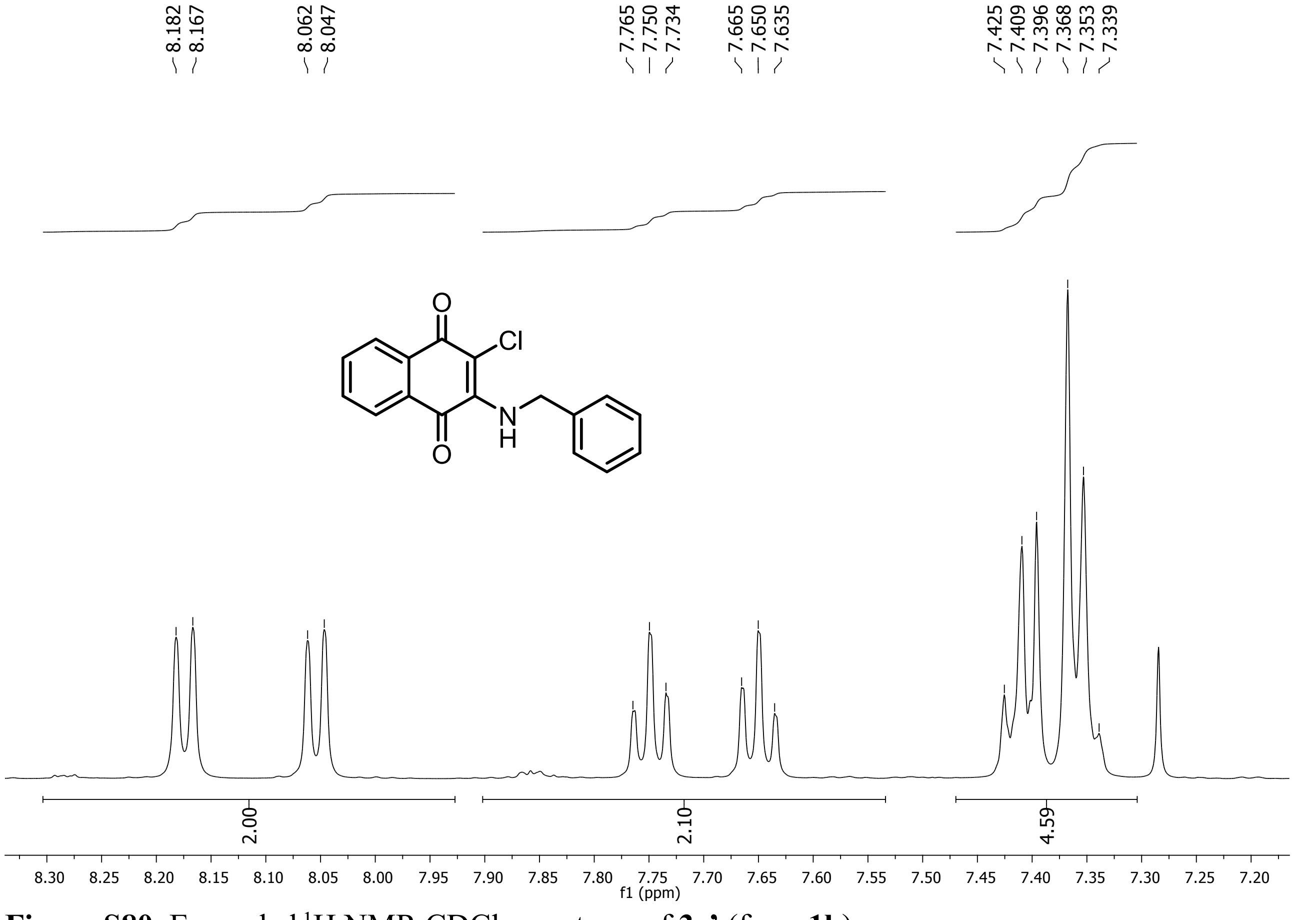

Figure S80. Expanded ^{1}H NMR $CDCl_3$ spectrum of **3r'** (from **1b**)

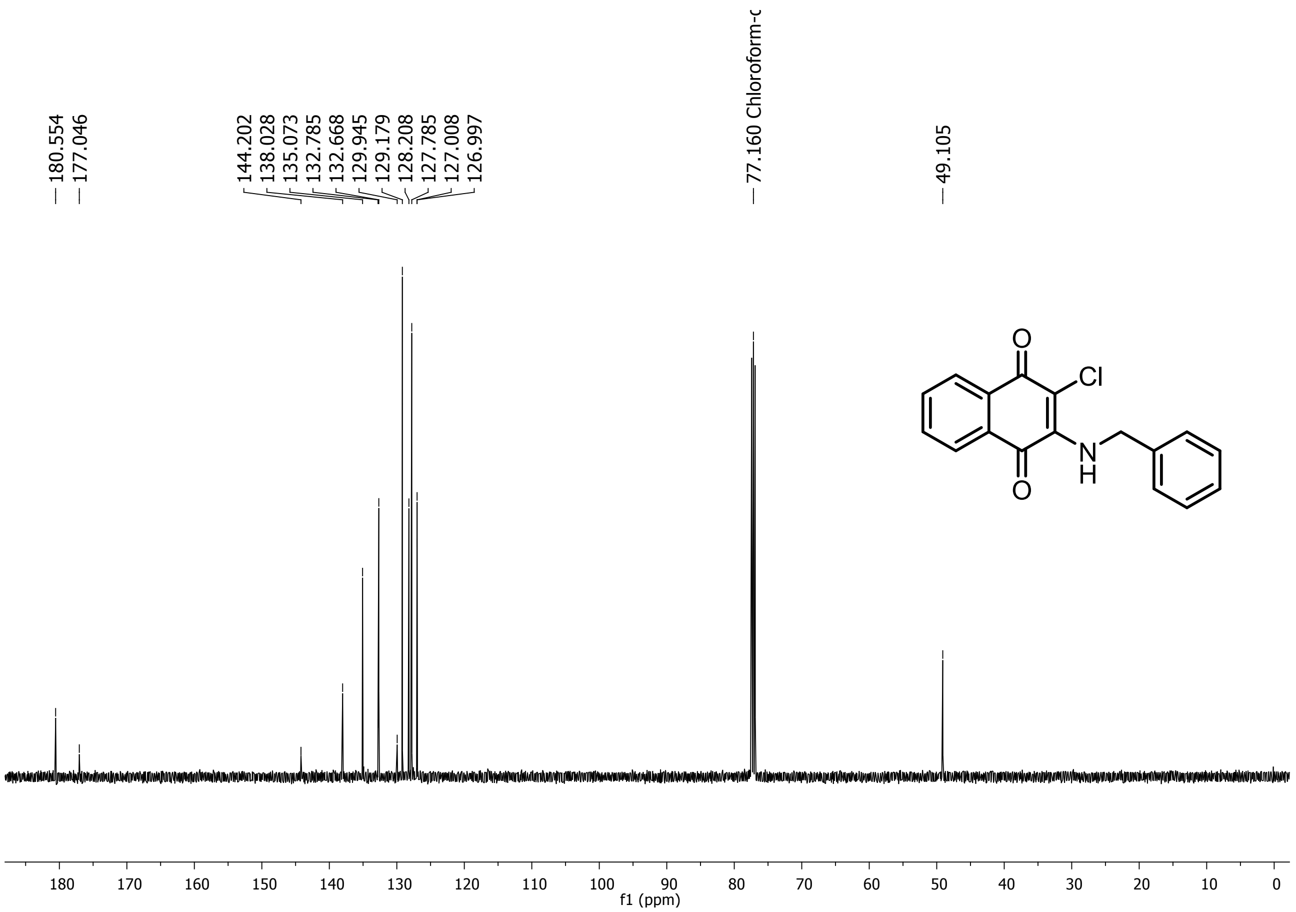

Figure S81. Full $^{13}C\{^{1}H\}$ NMR $CDCl_3$ spectrum of **3r'** (from **1b**)

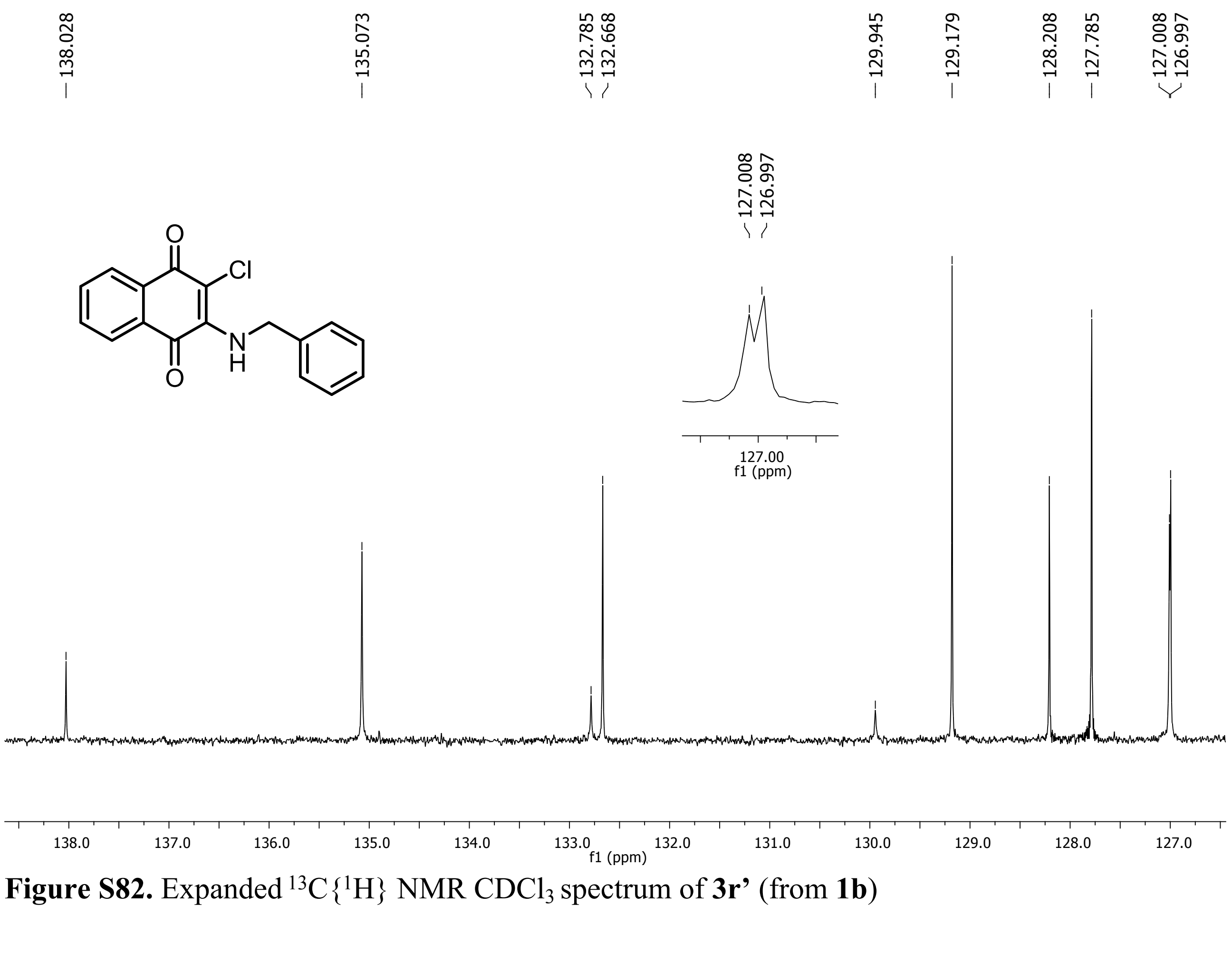

Figure S82. Expanded $^{13}C\{^{1}H\}$ NMR $CDCl_3$ spectrum of **3r'** (from **1b**)

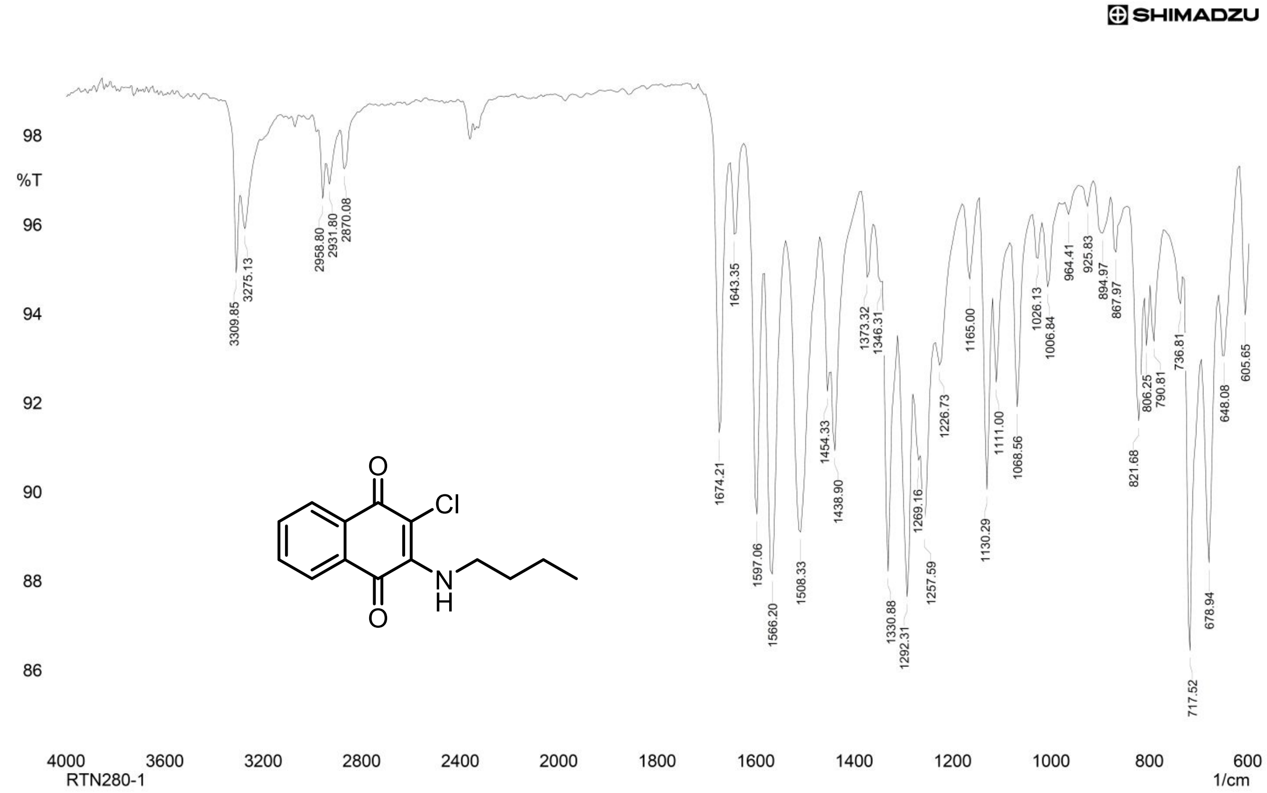

Figure S83. IR (ATR) spectrum of **3t'** (from **1b**)

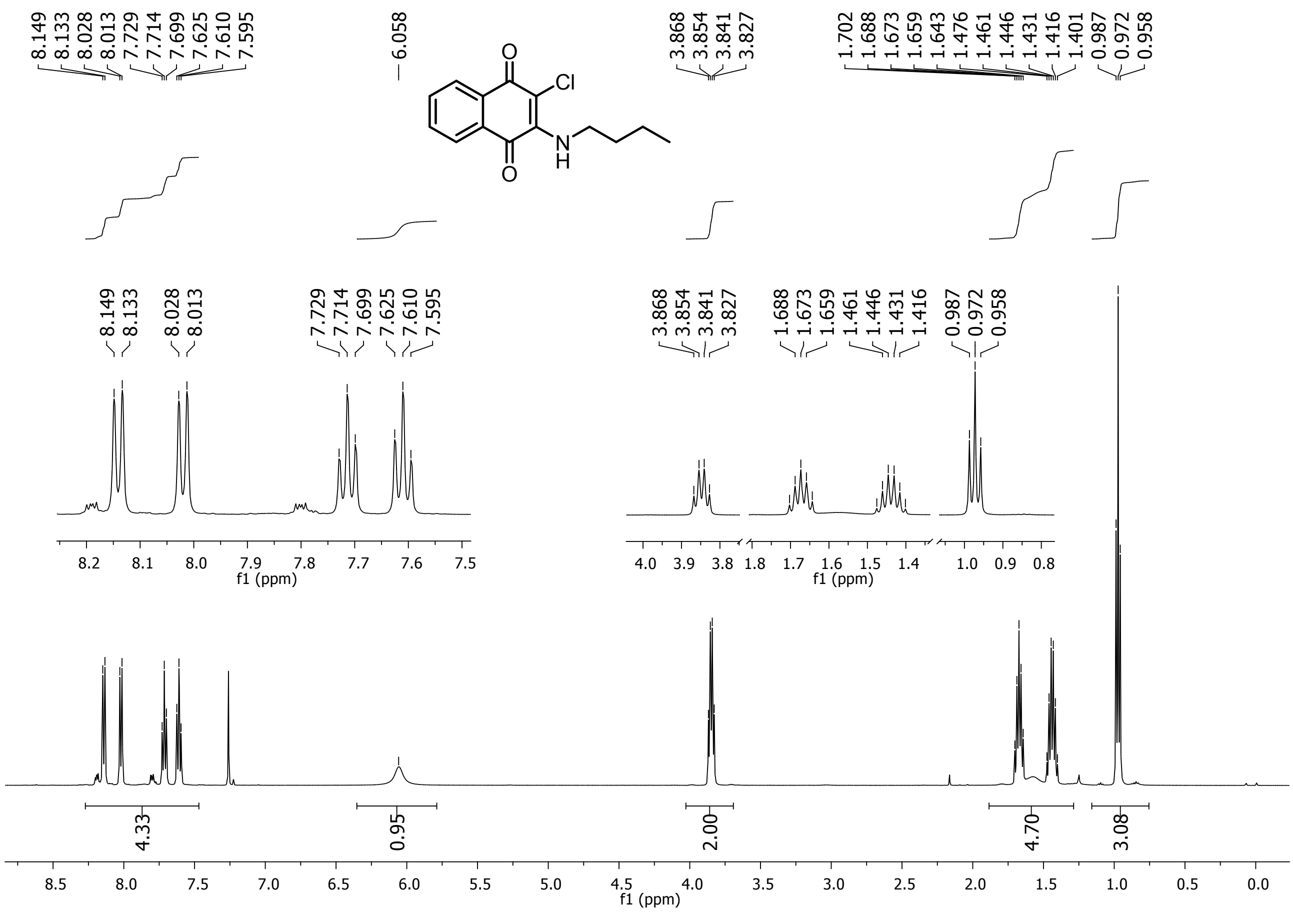

Figure S84. Full ^{1}H NMR $CDCl_3$ spectrum of **3t'** (from **1b**)

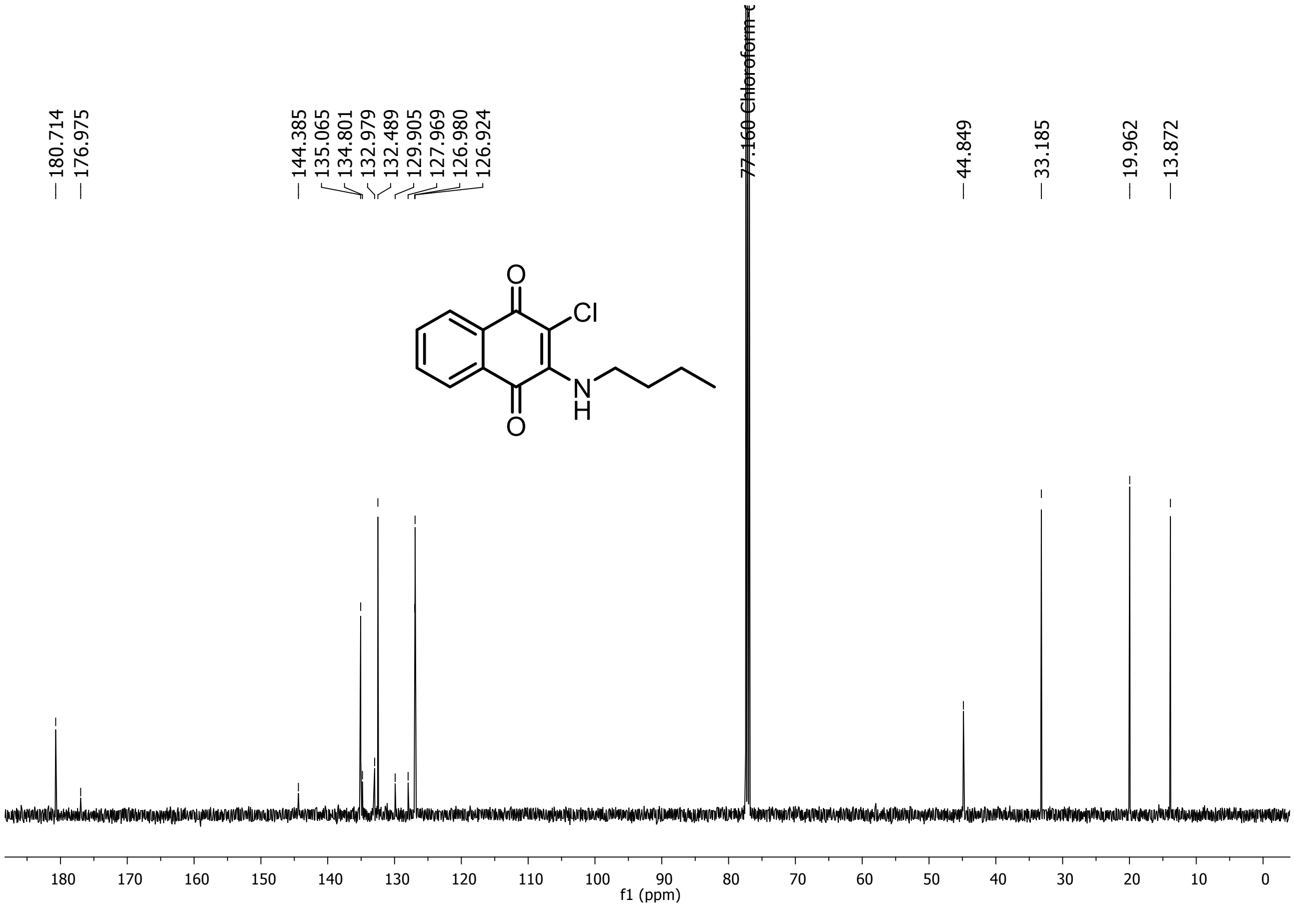

Figure S85. Full $^{13}C\{^{1}H\}$ NMR $CDCl_3$ spectrum of **3t’** (from **1b**)

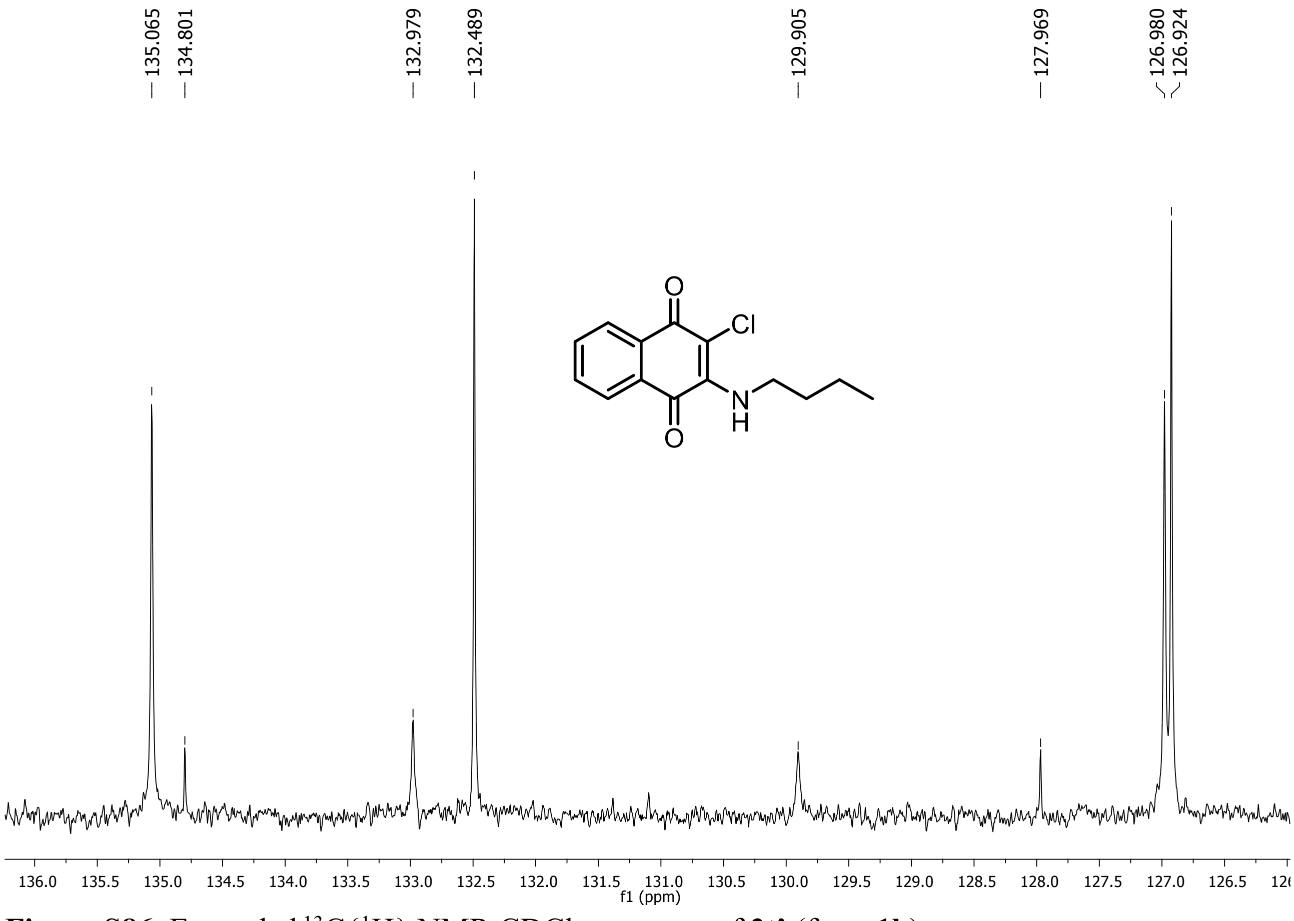

Figure S86. Expanded $^{13}C\{^{1}H\}$ NMR $CDCl_3$ spectrum of **3t’** (from **1b**)

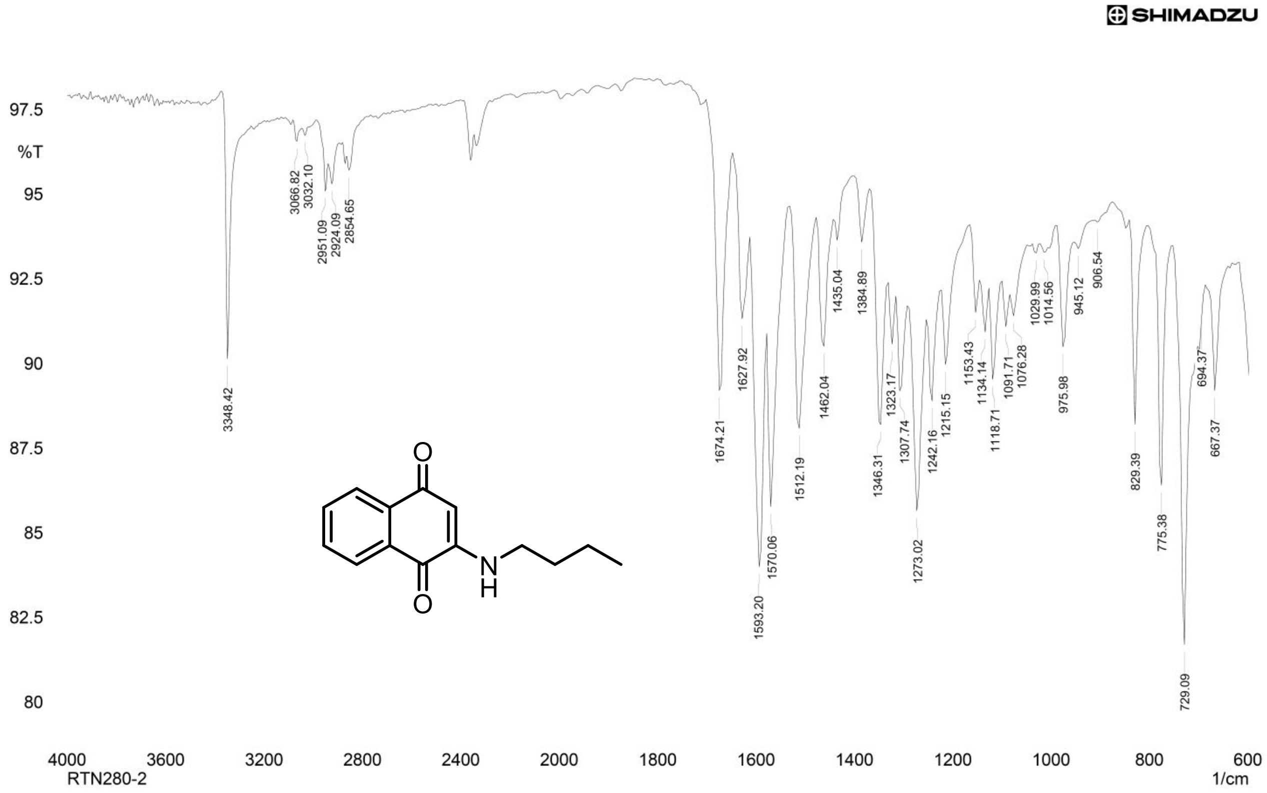

Figure S87. IR (ATR) spectrum of **3t** (from **1b**)

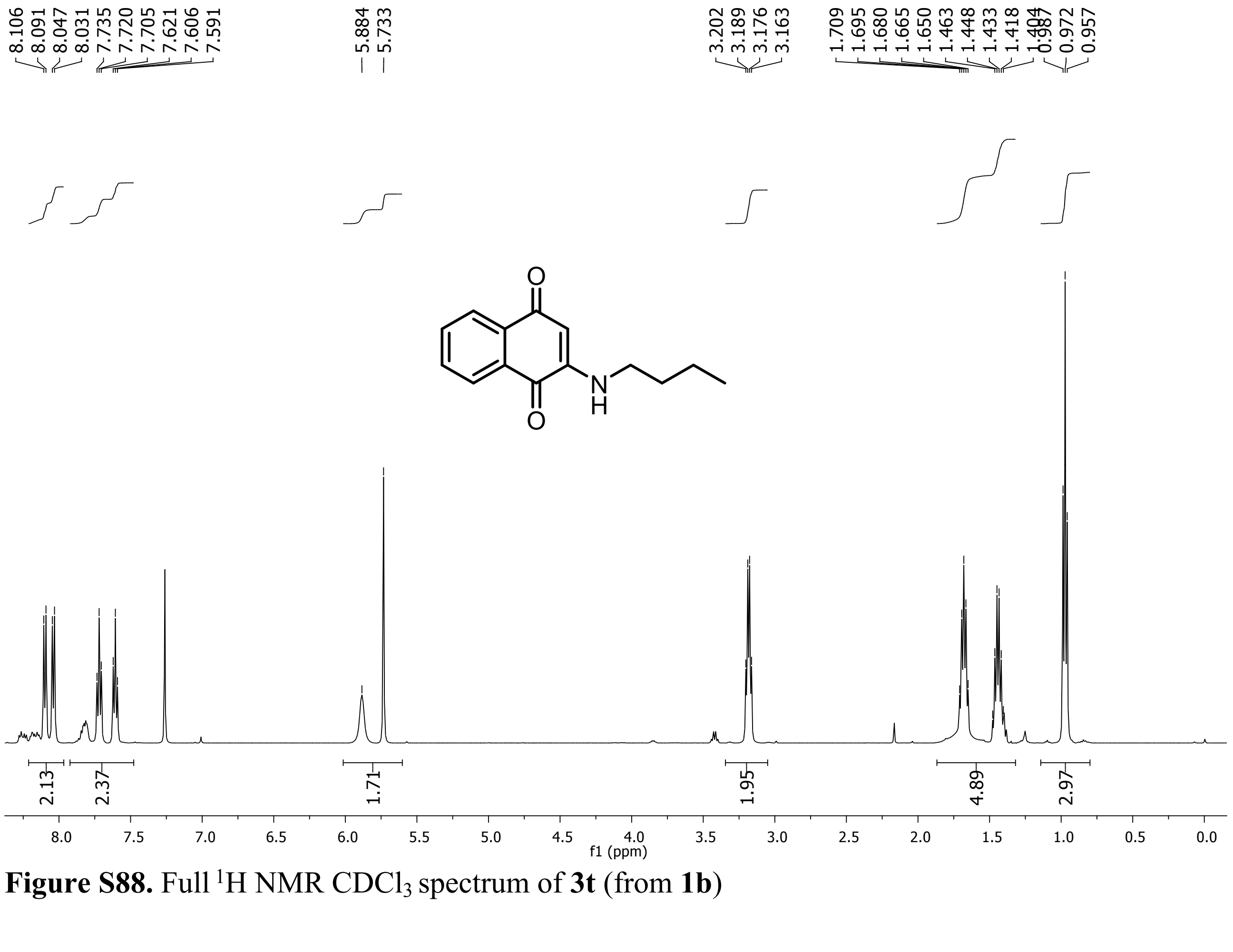

Figure S88. Full ^{1}H NMR $CDCl_3$ spectrum of **3t** (from **1b**)

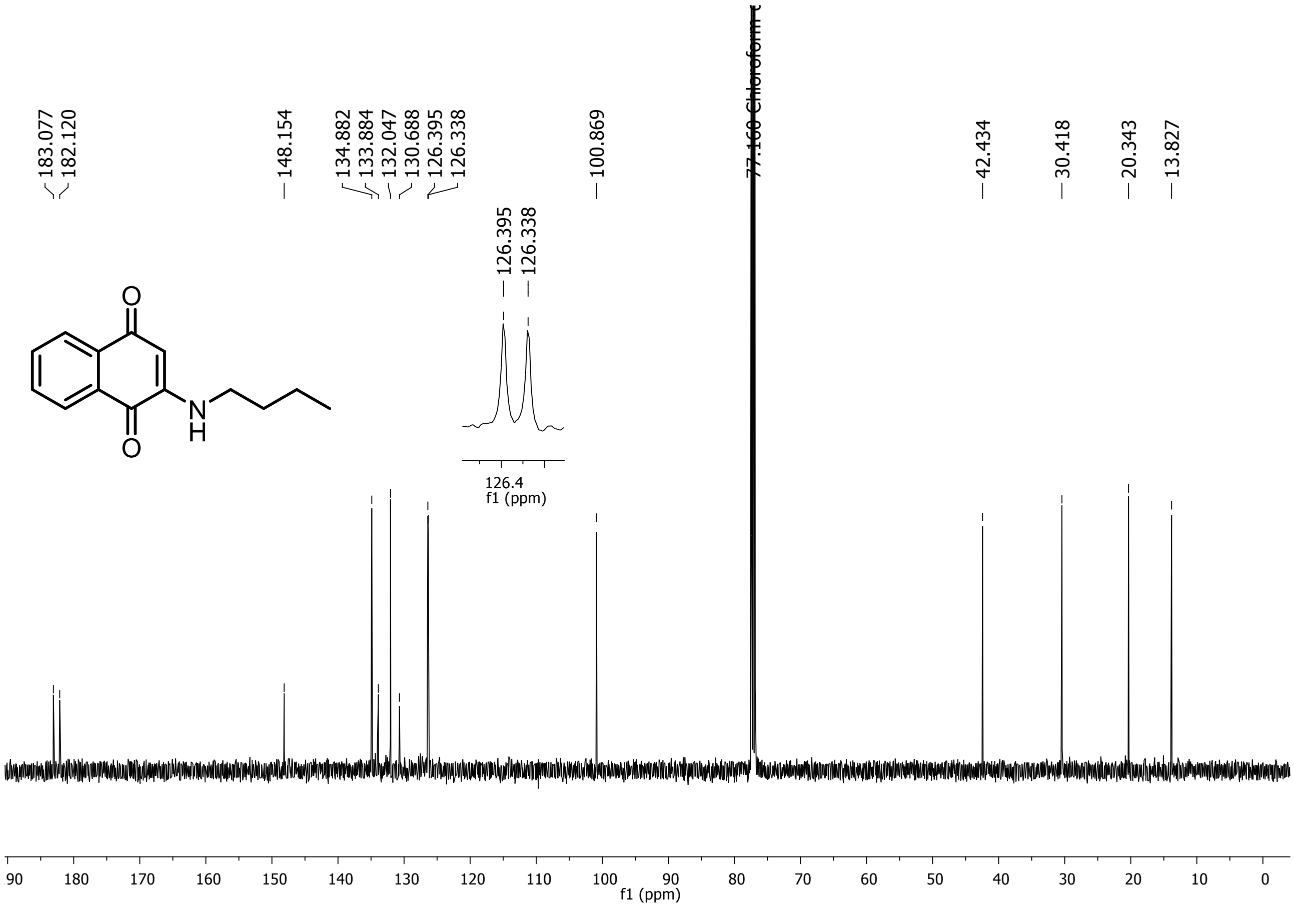

Figure S89. Full $^{13}C\{^{1}H\}$ NMR $CDCl_3$ spectrum of **3t** (from **1b**)

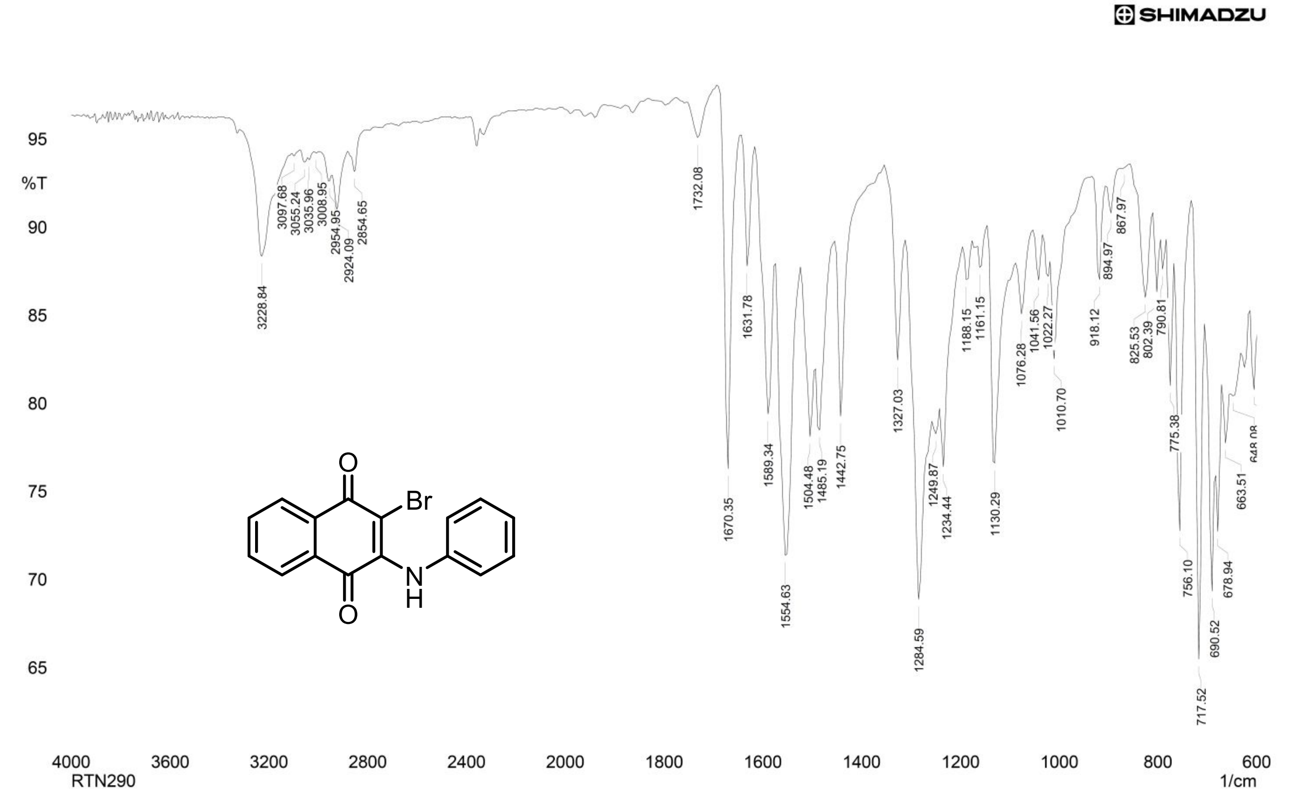

Figure S90. IR (ATR) spectrum of **3a''** (from **1d**)

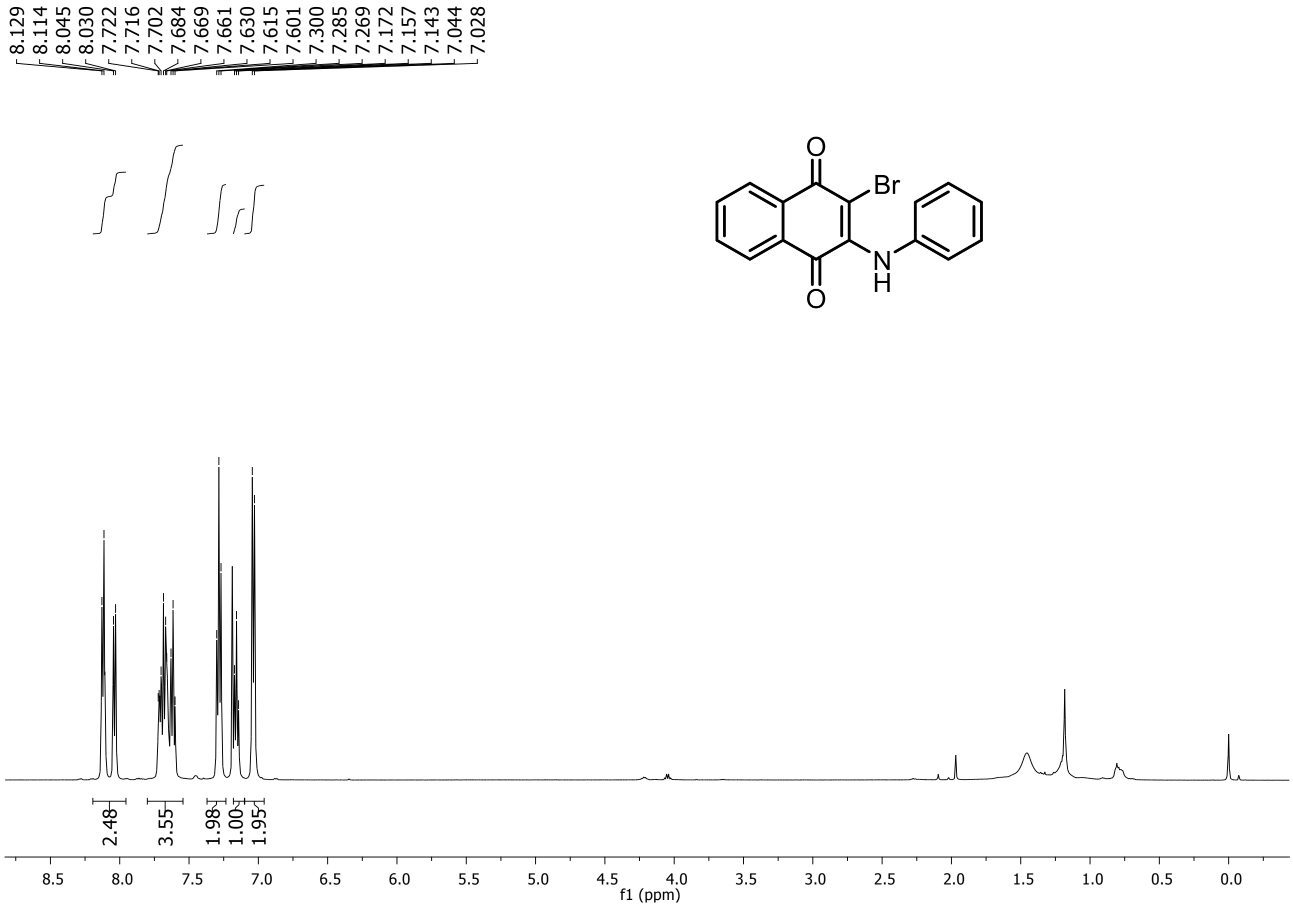

Figure S91. Full ^{1}H NMR $CDCl_3$ spectrum of **3a''** (from **1d**)

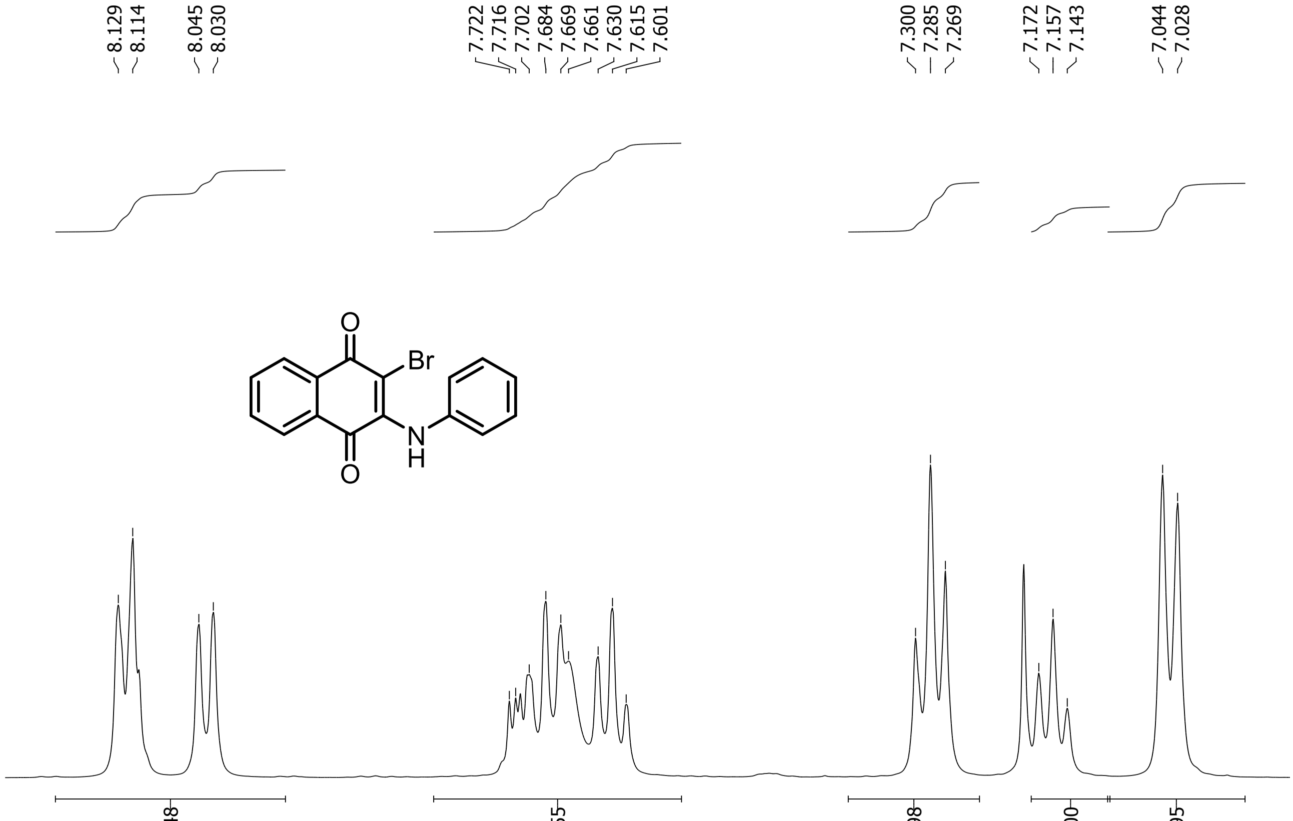

Figure S92. Expanded ^{1}H NMR $CDCl_3$ spectrum of **3a''** (from **1d**)

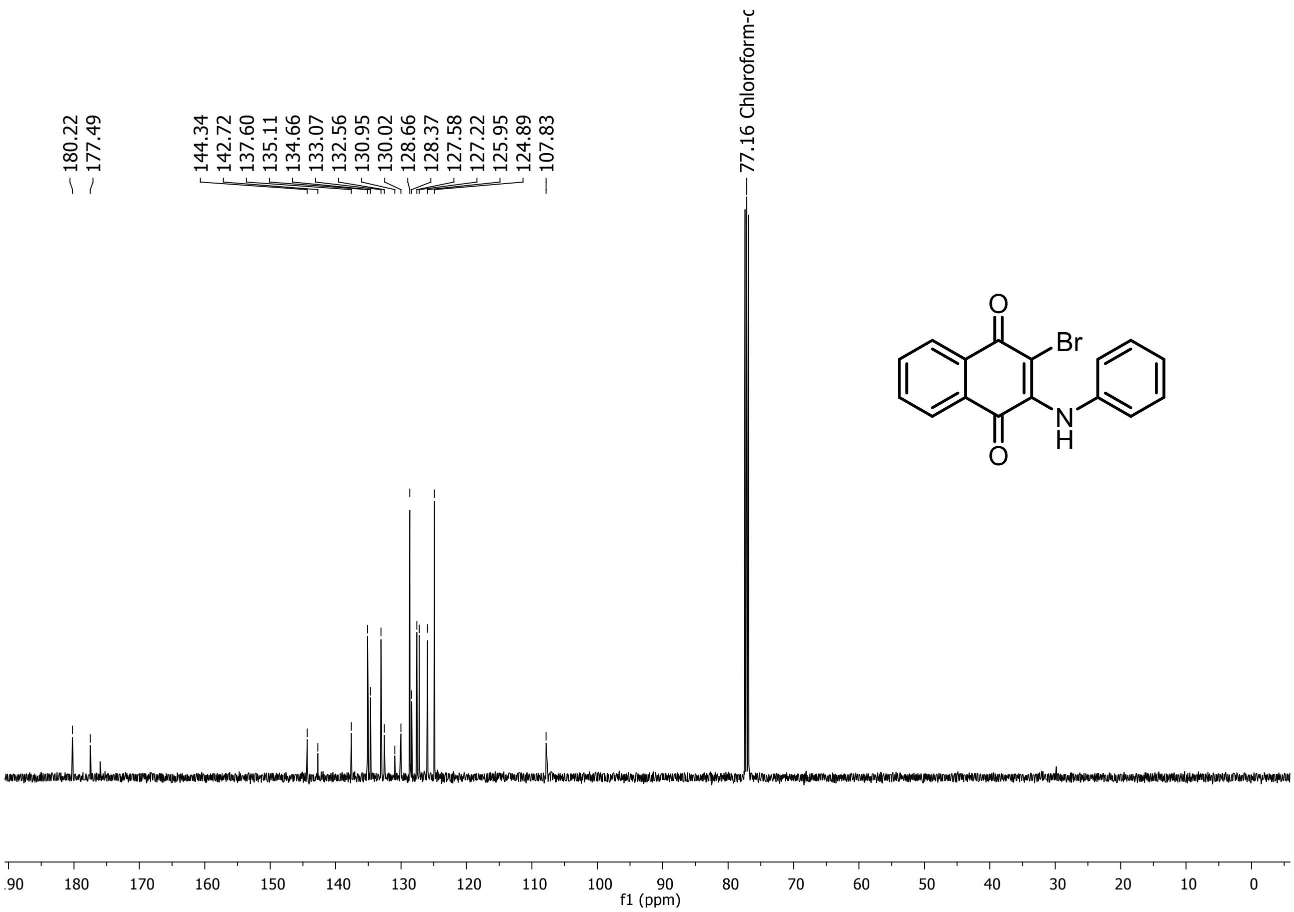

Figure S93. Full $^{13}C\{^{1}H\}$ NMR $CDCl_3$ spectrum of **3a''** (from **1d**)

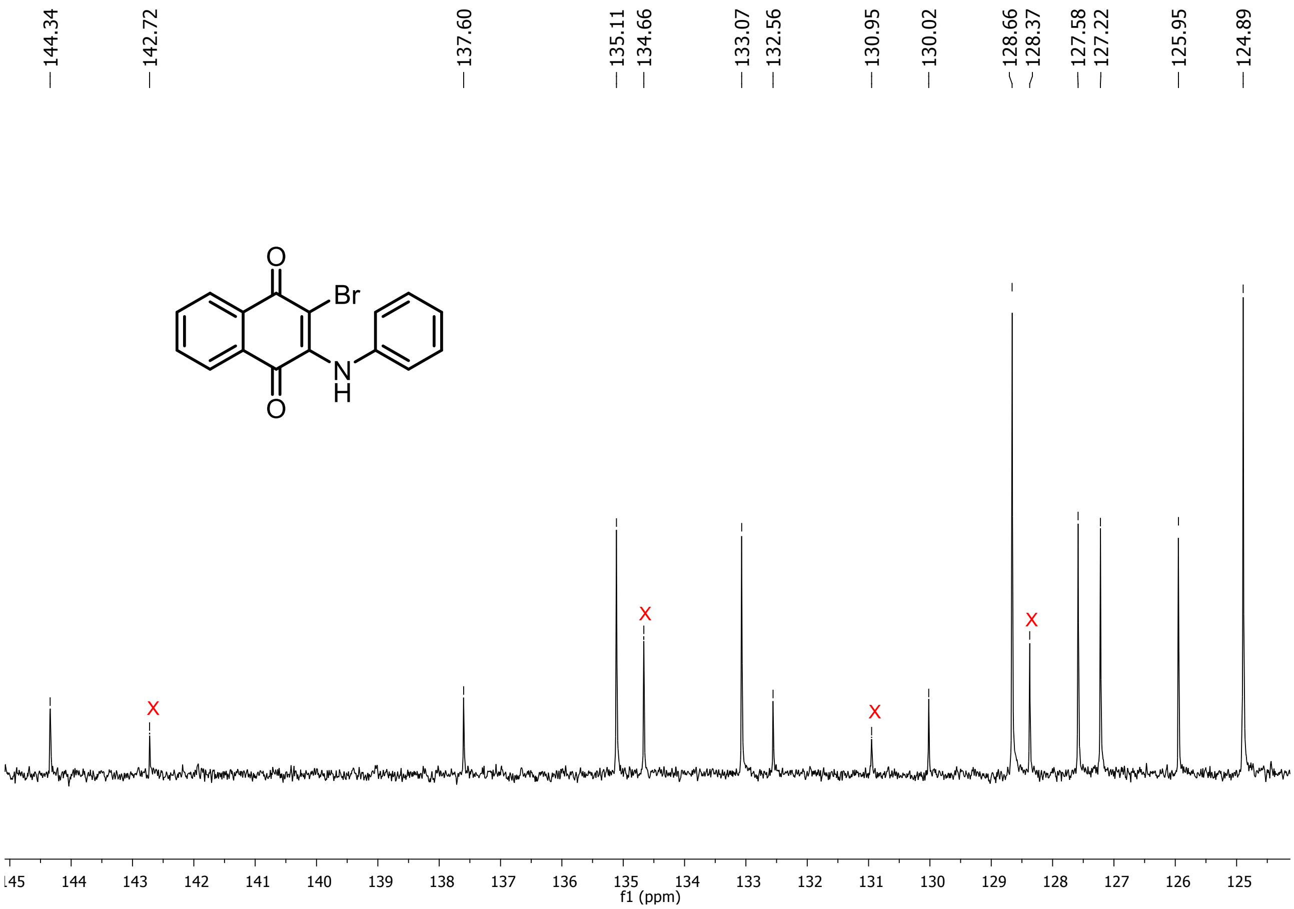

Figure S94. Expanded $^{13}C\{^{1}H\}$ NMR $CDCl_3$ spectrum of **3a''** (from **1d**)

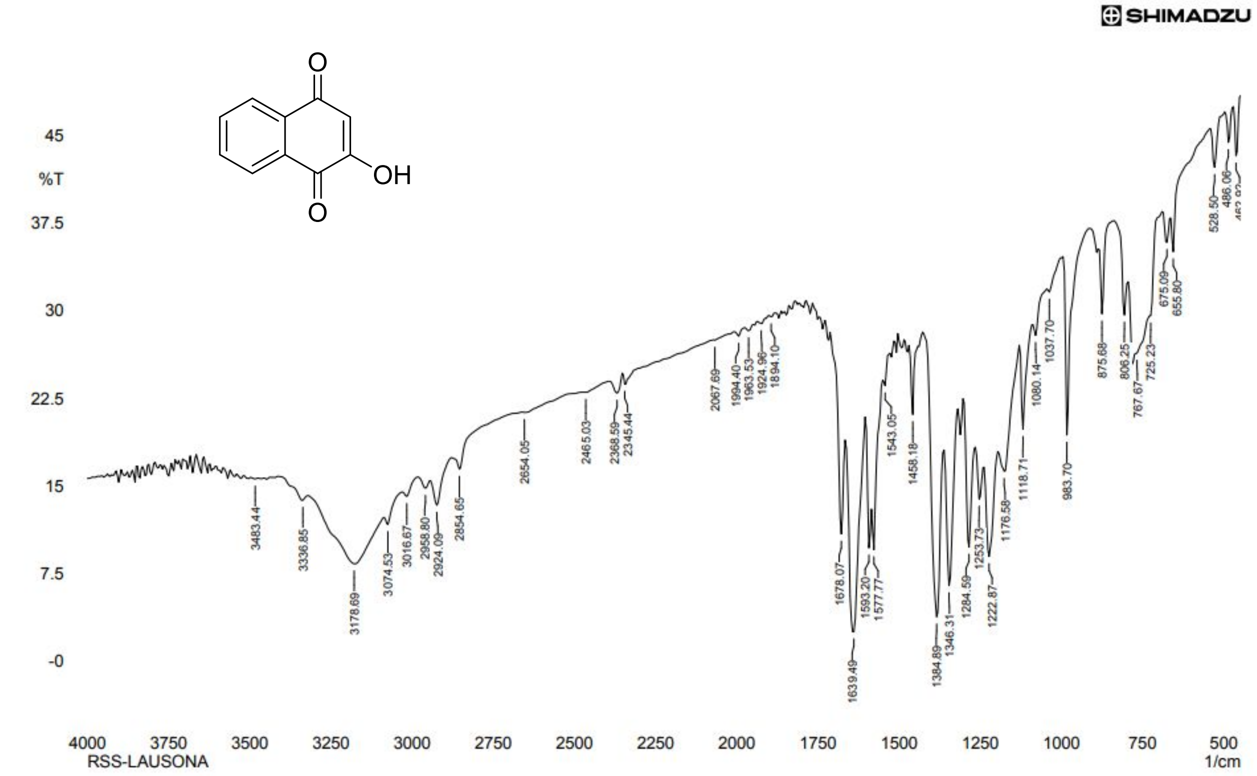

Figure S95. IR (KBr) spectrum of **8**

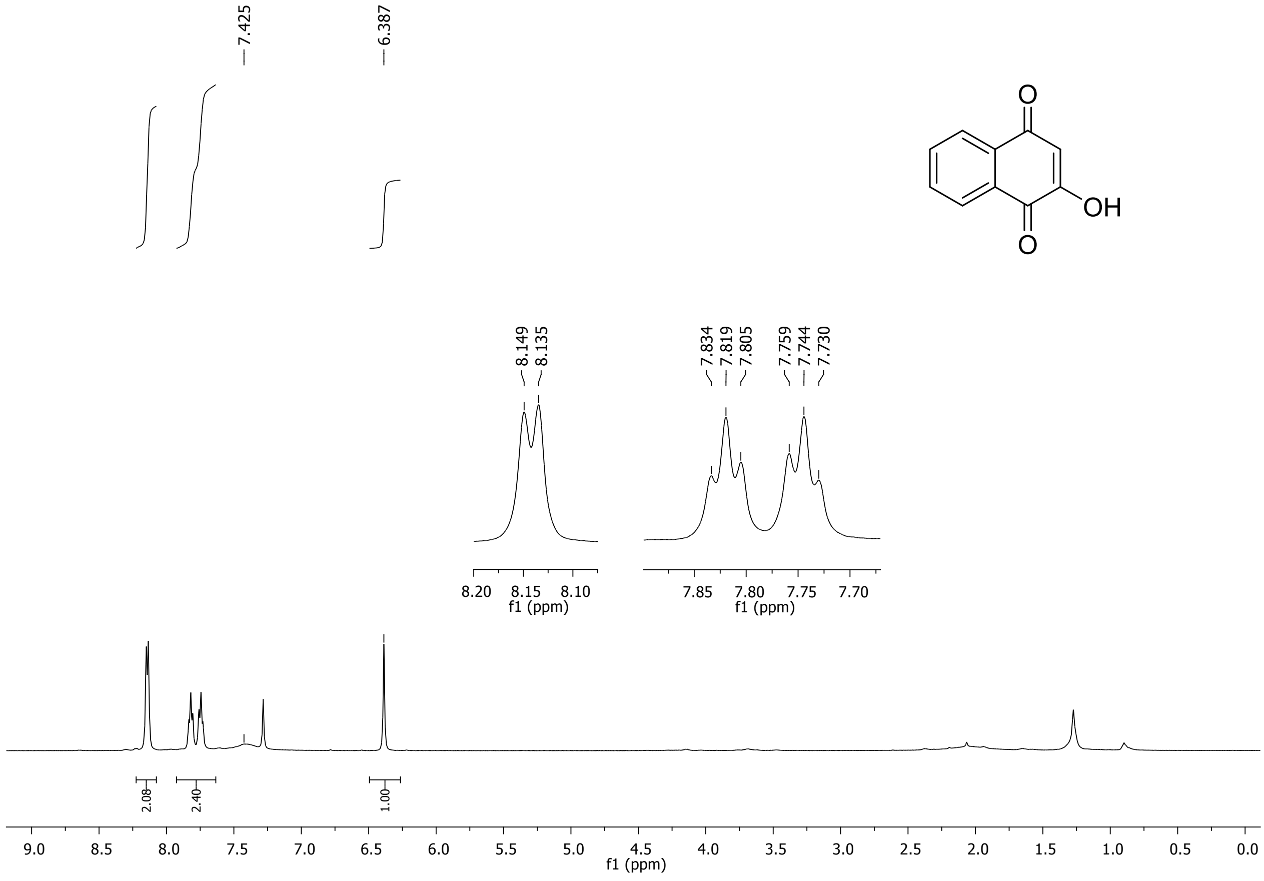

Figure S96. Full ^{1}H NMR $CDCl_3$ spectrum of **8**

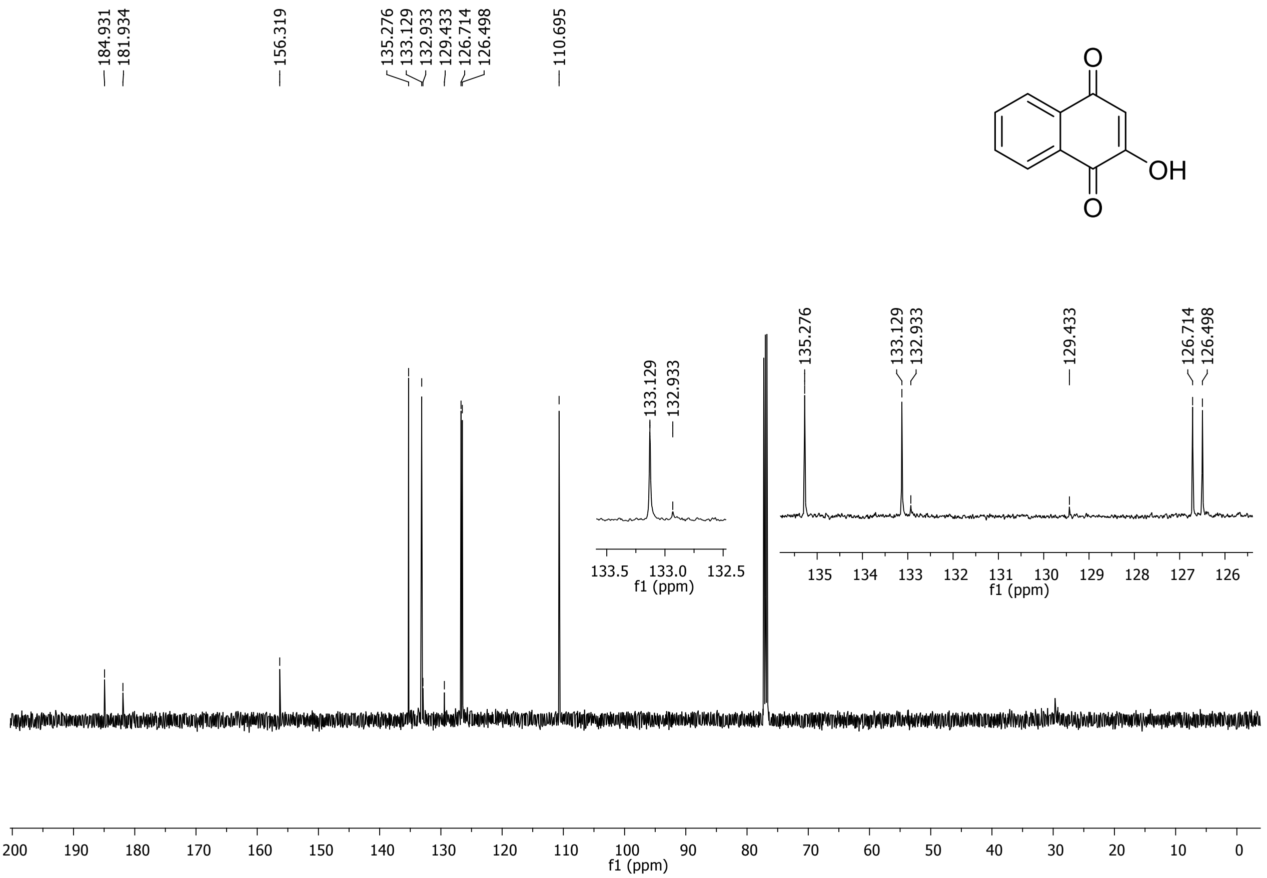

Figure S97. Full $^{13}C\{^{1}H\}$ NMR $CDCl_3$ spectrum of **8**
